# Functionalized 1,3-Thiazolidin-4-Ones from 2-Oxo-Acenaphthoquinylidene- and [2.2]Paracyclophanylidene-Thiosemicarbazones

**DOI:** 10.3390/molecules24173069

**Published:** 2019-08-23

**Authors:** Ashraf A. Aly, Nasr K. Mohamed, Alaa A. Hassan, Kamal M. El-Shaieb, Maysa M. Makhlouf, Stefan Bräse, Martin Nieger, Alan B. Brown

**Affiliations:** 1 Chemistry Department, Faculty of Science, Minia University, El-Minia 61519, Egypt; 2 Institute of Organic Chemistry, Karlsruhe Institute of Technology, Fritz-Haber-Weg 6, 76131 Karlsruhe, Germany; 3 Institute of Toxicology and Genetics (ITG) Karlsruhe Institute of Technology (KIT) Hermann-von-Helmholtz-Platz 1, D-76344 Eggenstein-Leopoldshafen, Germany; 4 Department of Chemistry, University of Helsinki, P.O. Box 55, A. I. Virtasen aukio I, 00014 Helsinki, Finland; 5 Chemistry Program, Florida Institute of Technology, Melbourne, FL 32901, USA

**Keywords:** acenaphthequinone, 4-acetyl[2.2]paracyclophane, thiosemicarbazone, dialkyl-acetylenedicarboxylates, 1,3-thiazolidinones, DMAD tetramer

## Abstract

The reactions of dialkyl acetylenedicarboxylates with various 2-oxo-acenaphthoquinylidene- and 4-acetyl[2.2]paracyclophanylidene-thiosemicarbazones were investigated. Using simple experimental procedures, 1,3-Thiazolidin-4-ones derived from acenaphthequinone or [2.2]paracyclophane were obtained as major products in good yields. In the case of allyl derivative of acenaphthoquinylidene-thiosemicarbazones, a complex structure of tetramethyl 5-(2-(((*Z*,*E*)-*N*-allyl-*N*′-(2-oxoacenaphthylen-1(2*H*)-ylidene)carbamohydrazonoyl)thio)-1,2,3-tris-(methoxycarbonyl)-cyclopropyl)-4-methoxy-7-oxabicyclo[2.2.1]hepta-2,5-diene-1,2,3,6-tetracarboxylate was formed. Single crystal X-ray analysis was used as an efficient tool to confirm the structure of the synthesized compounds as well as different spectroscopic data (^1^H-NMR, ^13^C-NMR, 2D-NMR, mass spectrometry and elemental analysis). The mechanism of the obtained products was discussed.

## 1. Introduction

Of interest due to their high biological activities, 1,3-Thiazolidin-4-one derivatives are very important core structures of many heterocyclic compounds. Compounds with a thiazolidinone structure have been reported as anti-inflammatory and analgesic [1], antioxidants [2], antitubercular [1,3], antimicrobial [2,4] and antifungal [4], antiviral (especially as anti-HIV agents [5]), anticancer [1], anticonvulsants [6], and antidiabetic agents [6,7]. Recently, various synthetic routes were used for the synthesis of different thiazolidinones from several thiosemicarbazides and thiocarbohydrazides by using different reagents, and their biological evaluation was also discussed [8,9]. Thiosemicarbazones were used as a starting material for the synthesis of different heterocyclic compounds, i.e., naphtho-thiazole and naphtho-thiadiazepines [10], 4-thiazolidinone derivatives [11,12], 1,3,4-thiadiazoles [13] which possess high biological activities, e.g., anticancer [14], antioxidants [15], antifungal [16], and antibacterial [17].

Acenaphthequinone (acenaphthylene-1,2-dione) is a quinone derivative, widely used as significant starting material for the synthesis of various heterocycles, and as a key intermediate in organic synthesis for different reactions and pharmaceutical applications [18,19,20,21]. Some derivatives of acenaphthequinone have been used as biological active compounds, dyes, pharmaceuticals, drugs, pesticides, and therapeutic agents [22,23,24]. Acenaphthylene-1,2-dione is one of the important reagents for constructing new heterocycles, e.g., spirooxadiazoline [25], spiroacenaphthyl-3,4′-pyranopyrazoles [26], fused acenaphthopyrrolizines and spiroacenaphthene pyrrole [27], dispiropyrrolidines [28,29], and polycyclic heterocycles [29,30] *via* reactions such as the Huisgen reaction, 1,3-dipolar cycloaddition, and [3+2]cycloaddition reactions. One pot synthesis of 2-oxo-1,2-dihydroacenaphthylphosphonates was obtained by using a Knoevenagel-phospha-Michael reaction of acenaphthequinone, malononitrile, and diethylphosphite catalyzed by a 1,4-diazabicyclo-[2.2.2]octane (DABCO)-based ionic liquid [31].

Recently, three component reactions were widely used as one of the most efficient routes for the synthesis of many heterocyclic rings. Consequently, three component reactions of acenaphthequinone, proline derivatives and dialkyl acetylenedicarboxylates gave azocinobenzoisoquinolone-dicarboxylates through [3+2]cycloaddition of azomethine ylides followed by ring expansion [32]. In addition, the domino three component reaction of isoquinolinium salts, acenaphthequinone and indanedione in basic media yielded 2′-acenaphthylidenespiro[indane-2,1′-pyrrolo[2,1-*a*]isoquinolones] [33]. Using a similar synthetic strategy, bis-spiro-oxoacenaphthalenes were obtained *via* the reaction of acenaphthalene-1,2-dione, active alkynes, and 4-cyanopyridine in 1,2-dimethoxyethane (DME) at room temperature [34]. The reaction of dialkyl acetylenedicarboxylate with acenaphthalenedione and CS_2_ in *t*-butylphosphine afforded oxoindolin-3-ylidene-1,3-dithioles [35]. Also, spirodihydropyridine derivatives were obtained in high yields from the reaction of acenaphthylidene malononitrile and Zwitter ionic intermediate (generated from primary amine and dimethyl acetylenedicarboxylate (DMAD) [36].

Since the maturation of the synthetic methodologies, with the possibilities for the fine-tuning of structural and functional properties, [2.2]paracyclophane (PC) chemistry has evolved—from functional molecules to functional materials [37]—and is rapidly growing from a structural and synthetic curiosity to a prevalent scaffold in asymmetric synthesis [37], π-stacked polymers, energy materials, and functional perylene coatings that find broad applications in bio-material science and as natural products [38,39,40]. Aly et al. prepared various heterocycles derived from [2.2]paracyclophane such as a five-membered ring (i.e., imidazolone and pyrrole [41,42,43,44] and six-membered ring (i.e., pyridine [45]). Additionally, we reported on the synthesis of various paracyclophanyl-substituted thiosemicarbazones, thiocarbazones and thioureas then studied their complexation towards tridentate and bidentate copper complexes [46]. Inspired by the aforementioned, we aim here to synthesize various thiazolidinone derivatives of both acenaphthequinone and [2.2]paracyclophane.

## 2. Results and Discussion

**Scheme 1:**
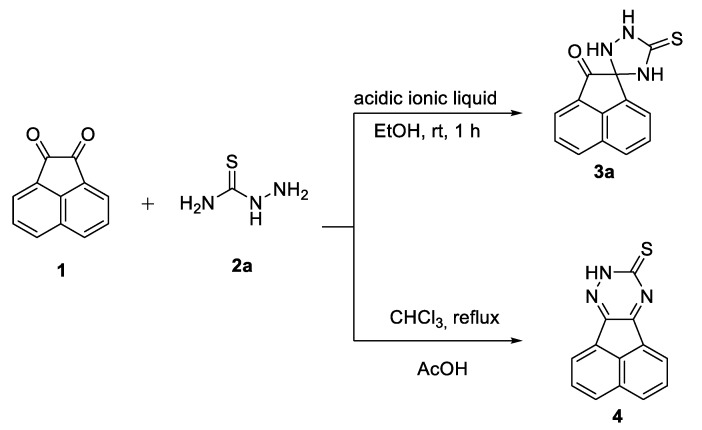
Previous work on acenaphthalene-1,2-dione **1** with thiosemicarbazide **2a**.

Acenaphthequinones conjugated with NH, SH, CH=N and NH_2_ groups are of considerable interest in organic synthesis and were produced from the condensation of thiosemicarbazide and 4-substituted thiosemicarbazides with acenaphthylene-1,2-dione [47]. A literature survey revealed, that acenaphthequinone (**1**) was reacted with thiosemicarbazide **2a** either in ethanol at room temperature and catalyzed by acidic ionic liquid furnished spiro-1,2,4-triazolidine-3-thiones **3a** [48] or in refluxing chloroform in the presence of glacial acetic acid (AcOH) yielded acenaphtho[1,2-*e*]-1,2,4-triazine-9(8*H*)-thione **4** (Scheme 1) [49].

### 2.1. Preparation of Compounds ***5a**–**e***

**Scheme 2:**
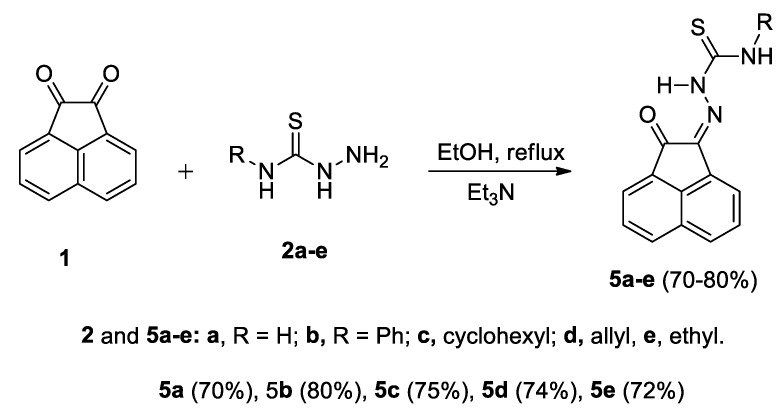
Reactions of acenaphthoqinone **1** with thiosemicarbazides **2a**–**e**.

**Figure 1:**
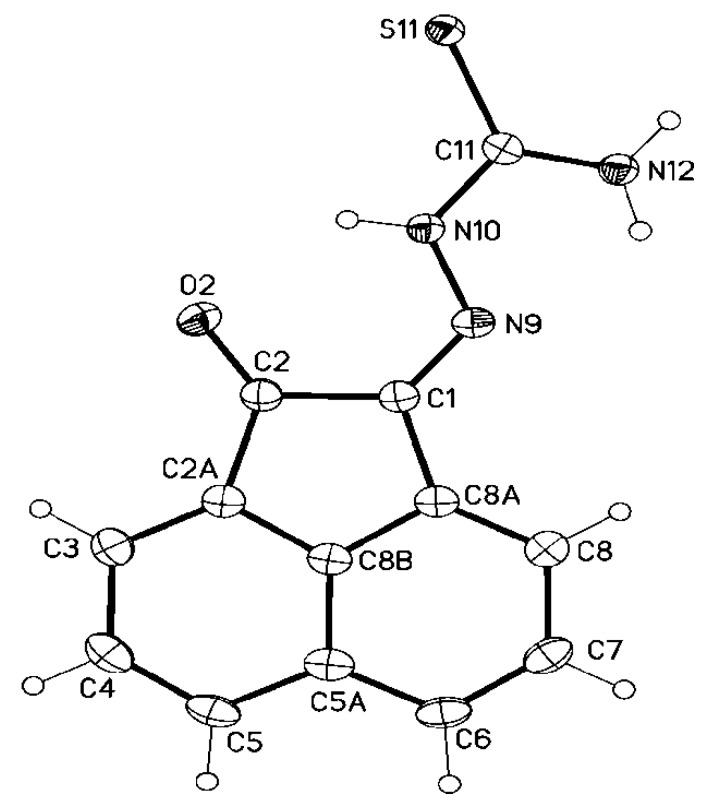
Molecular structure of one of the two crystallographic independent molecules of **5a** (displacement parameters are drawn at 50% probability level).

It was obvious that the reaction conditions, e.g., solvent, temperature and/or catalyst play a vital role on the reaction efficiency and product obtained. Various conditions were investigated and the most efficient method was the condensation of thiosemicarbazide derivatives with acenaphthalene-1,2-dione in refluxing ethanol in the presence of Et_3_N to give (*Z*)-*N*-unsubstituted/substituted-2-(2-oxoacenaphthylen-1(2*H*)-ylidene)hydrazinecarbothioamide derivatives (Scheme 2) instead of the aforementioned spiro-1,2,4-triazolidine-3-thione (**3a**) [48] and 1,2,4-triazine-3-thione **4** [49]. Although (*Z*)-2-(2-oxoacenaphthylen-1(2*H*)-ylidene)hydrazinecarbothioamide **5a** was previously reported [50], its structure was confirmed herein using single crystal X-ray analysis (Figure 1).

### 2.2. Reaction of Compounds ***5a**–**e*** with Dimethyl Acetylenedicarboxylate (DMAD, **6**)

**Scheme 3:**
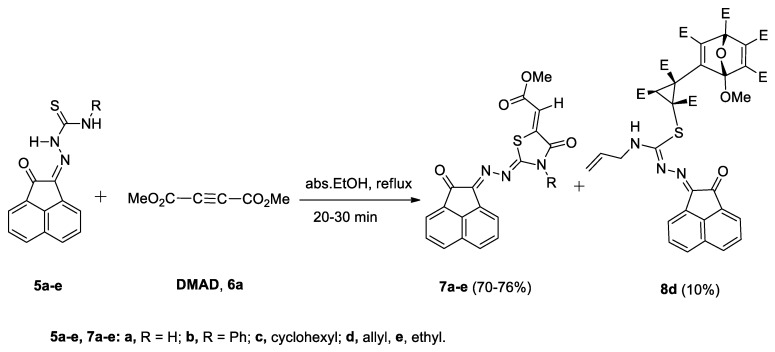
Reaction of 2-oxo-acenapthoquinylidene-thiosemicarbazones **5a**–**e** with **DMAD**, **6a**.

A mixture of dimethyl acetylenedicarboxylate (**DMAD**, **6a**) and (*Z*)-*N*-unsubstituted/substituted-2-(2-oxoacenaphthylen-1-(2*H*)-ylidene)thiosemicarbazones **5a**–**e**, was refluxed in absolute EtOH for 20–30 min. The reaction revealed the complete disappearance of compounds **5a**–**e** and **6a** to give compounds **7a**–**e** in 70–76% yields (Scheme 3). Surprisingly, only in the case of allyl derivative **5d**, an unexpected compound **8d** was obtained in 10% yield (Scheme 3). Different spectroscopic data were insufficient to determine completely the correct structure of this compound.

**Scheme 4:**
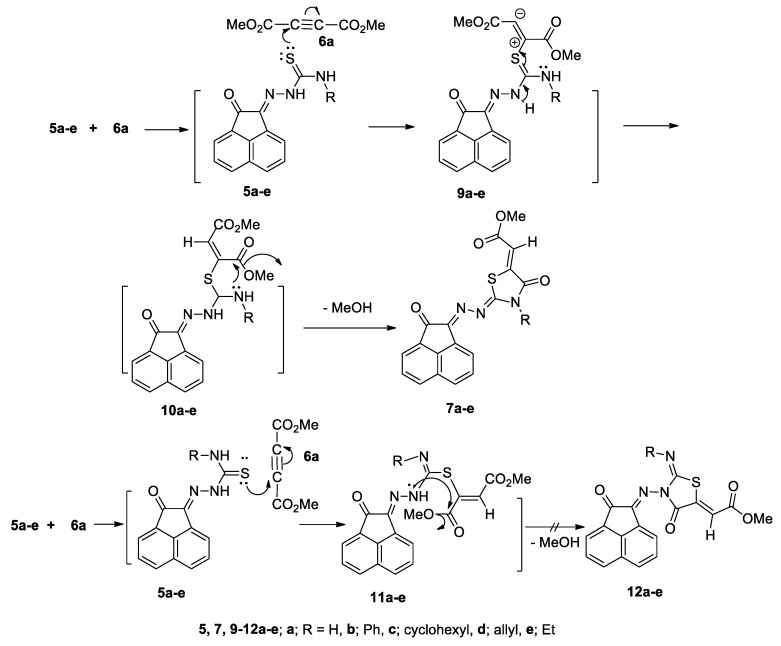
The mechanism describes the formation of compounds **7a**–**e** and **12a**–**e**.

Different nucleophilic sites on thiosemicarbazone derivatives **5a**–**e** were expected to participate in the reaction and formation of the product. Nucleophilic attack of lone pair of electrons of the sulfur atom in the thione group in **5a**–**e** to the acetylenic carbon in **6a** would give the Zwitter ions **9a**–**e**. Neutralization of **9a**–**e** would give the intermediate **10a**–**e**. Subsequently another nucleophilic attack from a lone pair of electrons of ^2^NH or ^4^NH on CO_2_Me; would yield the corresponding 4-thiazolidinones **7a**–**e** and/or **12a**–**e** *via* the loss of methanol (Scheme 4). The ^1^H and ^13^C-NMR spectral data (see details in Supplementary Materials) were used as an important tool for excluding some alternative structures. Furthermore, in the case of **7a**–**e** and **12a**–**e**, X-ray analyses were used as a useful tool to determine the correct structure and to prove that the 1,3-thiazolidine-4-ones **7a**–**e** were formed rather than **12a**–**e**.

**Figure 2:**
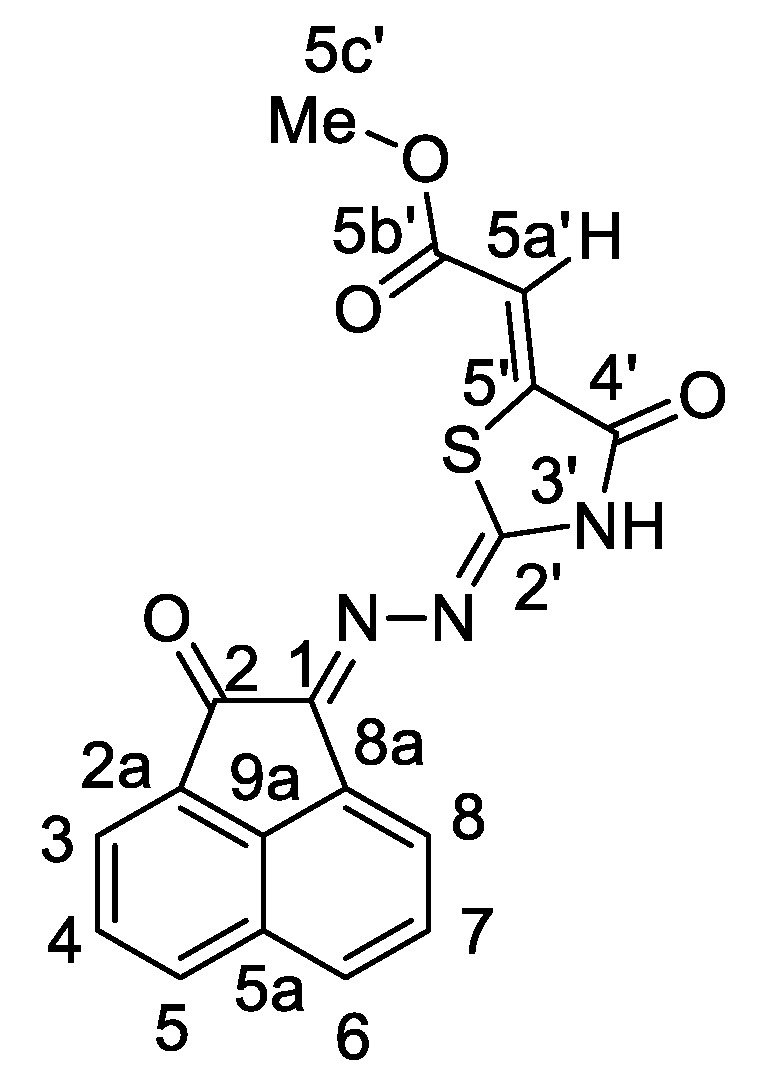
The distinctive carbons in compound **7a.**

For example, the IR spectrum of **7a** showed absorption bands at ν = 3489 cm^−1^ for the NH group, 1715, 1696 cm^−1^ for C=O as well as 1623 and 1609 cm^−1^ for C=N and Ar-C=C groups. The ^1^H-NMR spectrum of **7a** clearly proved that there are two singlets one at δ = 3.81 ppm with 3H integral corresponding to one methoxy group, the other at δ = 6.73 ppm with 1H integral relating to vinyl-CH group. Acenaphthequinone protons resonated at δ = 7.85–8.35 ppm due to six aromatic protons. A broad NH group also resonated as a singlet at 13.46 ppm. Further, ^13^C-NMR of **7a** showed further signals at δ = 52.49, 115.27, 142.07, (151.66 and 154.23), 165.78, 166.01 and 183.77 corresponding to OCH_3_, -CH=, C5-thiazole, (C2-thiazole and C=N), cyclic-C=O, CO_2_Me, C=O. The 2D-NMR further exhibited different correlations between C, H, N (Table 1 and Figure 2). The elemental analysis of **7a** suggests that it has C_18_H_11_N_3_O_4_S formula. This was further investigated from mass spectrum giving a molecular ion at *m*/*z* = 365 (10%).

**Figure 3:**
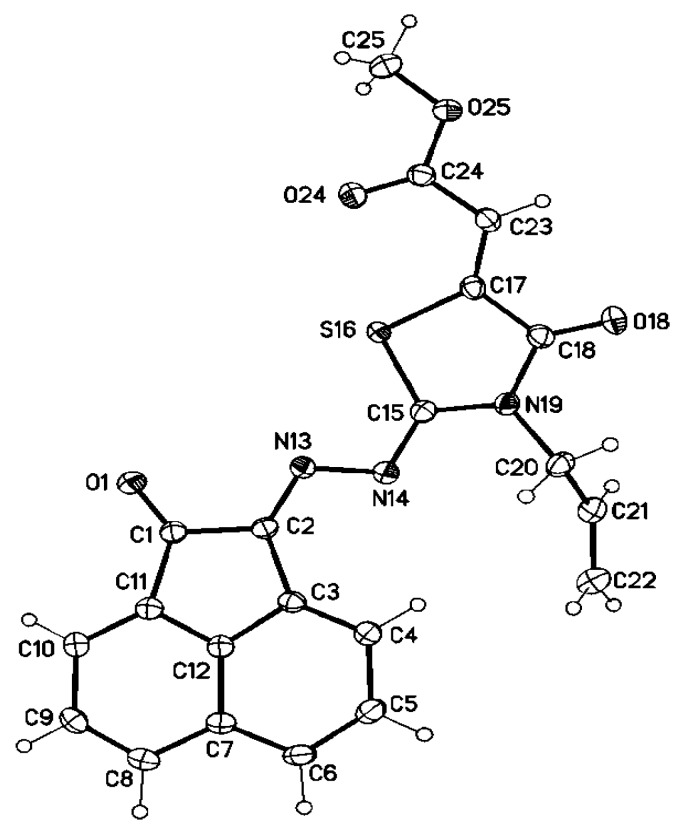
Molecular structure of **7d** (displacement parameters are drawn at 50% probability level).

Additionally, the structure of (*Z*)-methyl 2-((*Z*)-3-allyl-4-oxo-2-((*E*)-(2-oxoacenaphthylen-1(2*H*)-ylidene)hydrazono)thiazolidin-5-ylidene)acetate (**7d**) has been unambiguously confirmed *via* a single-crystal X-ray structure analysis (Figure 3). The characteristic properties of compound **7d** are that the C2-N13 and C15-N14, double bonds (note that the crystallographic numbering does not correspond to the systematic IUPAC numbering rules) exhibit bond lengths of 1.2912 (16) Å and 1.2892 (16) Å, respectively, that are a little shorter than C = N δ bonds due to high resonance in **7d**. The dihedral angle of N19-C15-N14-N13 is 178.82 (9)°, showing that it has a trans configuration relating to N19. Bond lengths C1-C2 and N13-N14 are 1.5345 (16) and 1.3950 (14), respectively, which are shorter than the corresponding C-C and N-N single bonds due to high resonance in this compound. From X-ray analysis, it was also observed that the thiazolidine C = O has a cisoid geometry regarding to vinyl CH [torsion angle O18–C18–C17–C23 −2.2 (2)°].

A literature survey revealed that, the tetramerization reaction is one of the most fascinating reactions of both dialkyl acetylenedicarboxylic acid derivatives occurring at 25 °C for several days or by heating it alone or in solution for several hours [51,52,53]. Furthermore, trimer [54] and dimer [55] forms were reported for compound **6a**. Recently, Huang et al. [56] have reported the reaction of 2,2′-bis(azaphosphindole) with four equivalents of **6a** in THF at room temperature to afford a tetramer complex of **6a** with 2,2′-bis(azaphosphindole).

**Figure 4:**
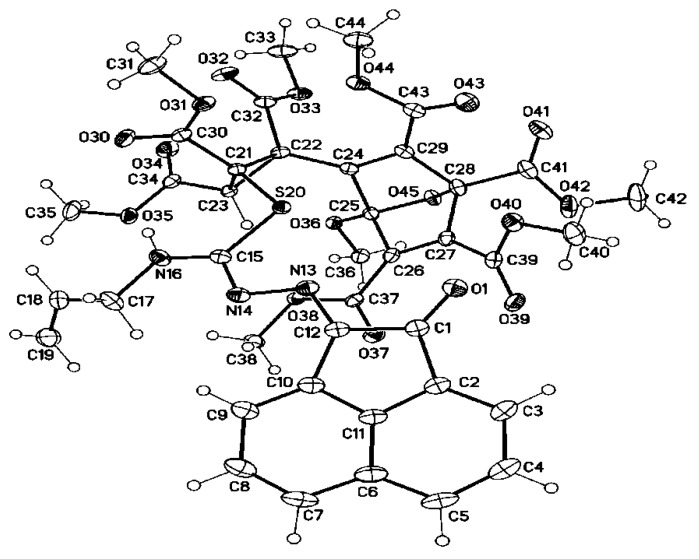
Molecular structure of one of the two crystallographic independent molecules of **8d** (displacement parameters are drawn at 30% probability level).

The structure of **8d** was resolved by using single crystal X-ray analysis and it was found that it is tetramethyl 5-(2-(((*Z*,*E*)-*N*-allyl-*N*′-(2-oxoacenaphthylen-1(2*H*)-ylidene)carbamo-hydrazonoyl)-thio)-1,2,3-tris(methoxycarbonyl)cyclopropyl)-4-methoxy-7-oxabicyclo-[2.2.1]hepta-2,5-diene-1,2,3,6-tetracarboxylate (**8d**) (Figure 4). To our best knowledge, it would be the first X-ray structure of that tetramer of **6a**. It is interesting to note that only one diastereomer was isolated (at least being isolated).

**Scheme 5:**
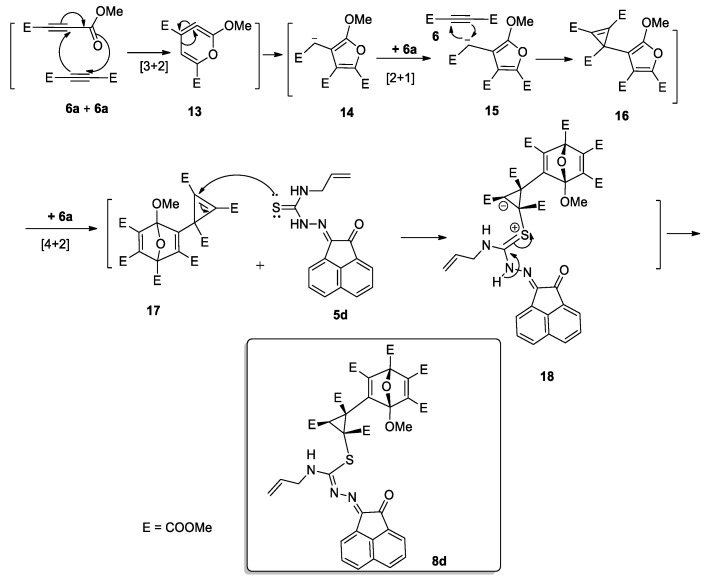
Plausible mechanism for the synthesis of compound **8d**.

The unusual reactivity of **6a** towards allyl derivative of thiosemicarbazones **5d** obviously involves four molecules of **6** reacting with one molecule of **5d**. The mechanism presumably begins *via* dimerization of **6a** (acting as dienophile and as 1,3-dipole) to afford **13**, which then rearranged to give **14**. Addition of the third molecule of **6a** to the intermediate **14** would give the intermediate **15**, which would be neutralized to give cyclopropene **16** (Scheme 5). Diels-Alder reaction of **16** and the fourth molecule of **6a**, yield the complex **17** [56] (Scheme 5). Subsequently, a nucleophilic attack of thione-lone pair of thiosemicarbazone **5d** to the double bond of the cyclopropene ring would give the Zwitter ion **18**, which would be neutralized to give compound **8d** (Scheme 5).

**Scheme 6:**
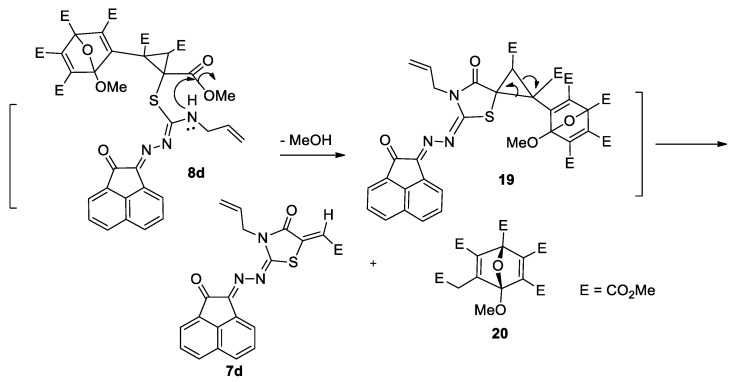
Formation of thiazolidinone **7d** from compound **8d**.

The formation of a complex structure may be further used as a Supplementary tool for the formation of 1,3-thiazolidinone derivative **7d**, which might be attributed to the instability of **8d** during the course of a reaction. It can be also suggested that another nucleophilic attack of NH lone-pair of compound **8d** to one of the carbonyl ester group of the cyclopropane ring afforded the intermediate **19** that would be rearranged giving the 4-thiazolidinone **7d** and **20** (Scheme 6). However, compound **20** was unfortunately not isolated.

On the other hand, the synthesis of 4-acetyl[2.2]paracyclophanylidene thiosemicarbazone (**22a**) was established from the reaction between 4-acetyl[2.2]paracyclophane (**21**) and thiosemicarbazide (**2a**) and by applying the procedure described by Aly et al. [46].

**Scheme 7:**
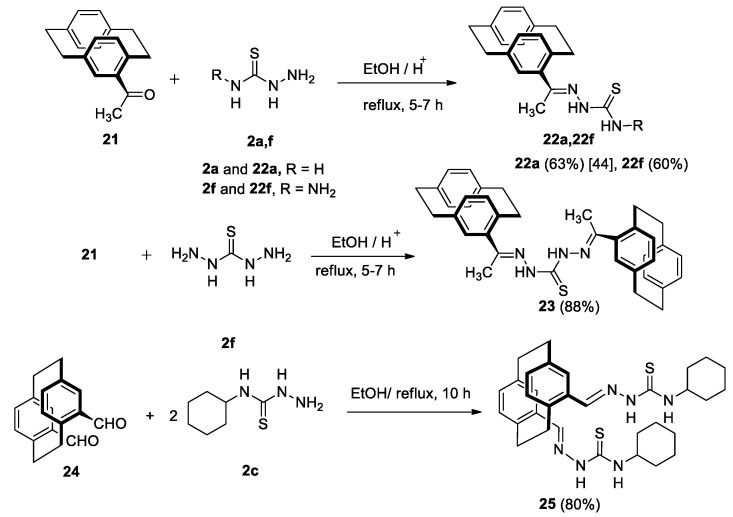
Synthesis of paracyclophane thiosemicarbazone derivatives **22a,b**, **23** and **25**.

**Figure 5:**
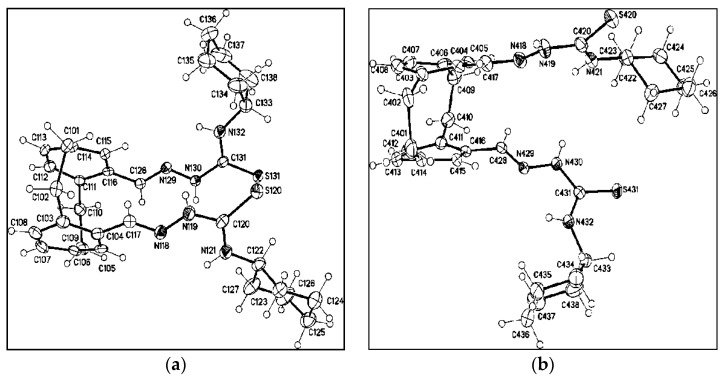
Molecular structure of two of the four crystallographic independent molecules of **25** showed the two different conformations in the crystal (displacement parameters, are drawn at 30% probability level).

Similarly, the reaction of **21** with **2f** gave **22f** in 60% yield (Scheme 7). If two equivalents of **21** were reacted with one equivalent of thiocarbohydrazide (**2f**), the reaction gave bis-4-acetyl-[2.2]paracyclophanylidene-hydrazine-1-carbothiohydrazone (**23**) in 88% yield. In addition, refluxing of the two equivalents of *N*-cyclohexylhydrazinecarbothioamide (**2c**) with 4,15-diformyl-2.2]paracyclophane (**24**) yielded the desired 2,2′-(1,4(1,4)-dibenzen-acyclohexaphane-1^2^,4^3^-diylbis(methane-ylylidene))bis-(*N*-cyclohexylhydrazine-1-carbothioamide (**25**) in 80% yield (Scheme 7). The X-ray structure analysis of compound **25** is as shown in Figure 5.

**Scheme 8:**
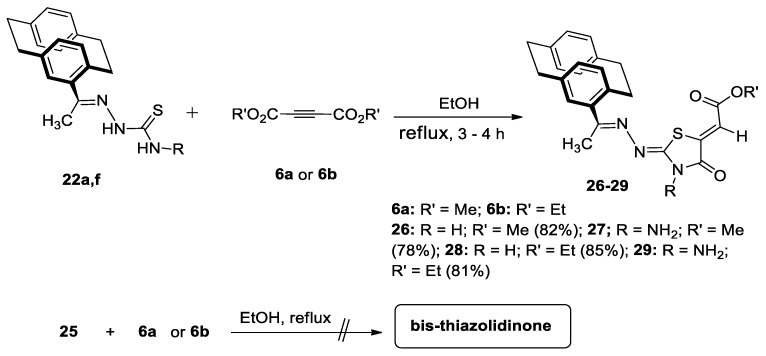
Reaction of [2.2]paracyclophanylidene thiosemicarbazones **22a**,**f** with **6a** and **6b**.

**Figure 6:**
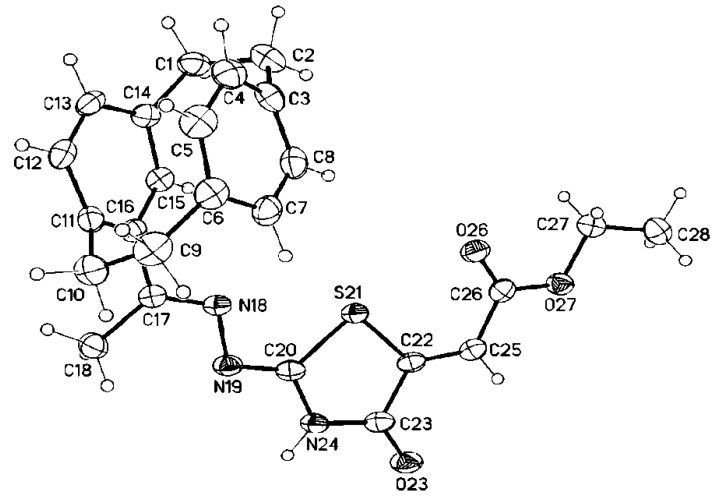
Molecular structure of **28** (displacement parameters are drawn at 30% probability level).

We aimed to synthesize thiazolidinones bearing a paracyclophanyl moiety; accordingly, we reacted 4-acetyl[2.2]paracyclophanylidene-thiosemicarbazone derivatives **22a** and **22f** with **DMAD**, **6a** and **DEAD**, **6b** (Scheme 8). By adaptation of the previously mentioned procedure, the expected 1,3-thiazolidinones **26**–**29** were obtained in good yields (Scheme 8). Depending on ^1^H-NMR and ^13^C-NMR, various alternative structures were ruled out. In order to distinguish between the expected 1,3-thiazolidinone derivatives, a single crystal of **28** was obtained as ethyl 2-((*Z*)-2-(((*E*)-1-(1,4(1,4)-dibenzenacyclohexaphane-1^2^-yl)ethylidene)hydrazine-ylidene)-4-oxothiazolidin-5-yl)acetate (Figure 6).

Prolonged heating of **25** with either **6a** or **6b** under the aforementioned conditions failed to give either a mono- or a bis-thiazolidinone structure. Either the sensitivity of such layered molecules towards light and heat to undergo dimerization and/or steric factors might cause the reaction not to occur.

**Scheme 9:**
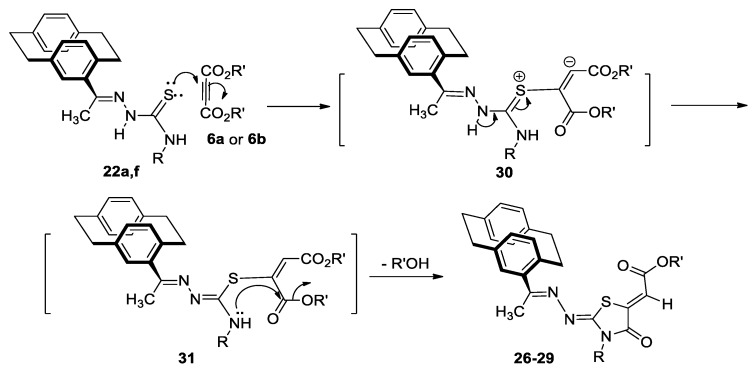
Mechanism described the formation of compounds **26**–**29**.

By analogy, the mechanism of formation of compounds **7a**–**e** would be similar to that for the formation of compounds **26**–**29** (Scheme 9). Nucleophilic attack from sulfur lone pair in **22a**,**f** to the triple bond of either **6a** or **6b** would give the intermediate **30,** which would be neutralized as described before, to give **31**. The intermediate **31** would be easily cyclized to give the corresponding thiazolidinones **26**–**29** (Scheme 8), *via* another nucleophilic attack from NH in the formed intermediate **32** to the ester-C=O and liberation of methanol or ethanol (Scheme 9).

## 3. Material and Methods

### 3.1. Chemistry

Melting points (mp’s) were recorded on a Gallenkamp melting point apparatus (Gallenkamp, UK) using open capillaries and were uncorrected. NMR data were recorded on Bruker AM 400 or AV400 spectrometers (Bruker, Rheinstetten, Germany), at 400 MHz for ^1^H and 100 MHz for ^13^C. Chemical shifts were reported in ppm from tetramethylsilane using solvent resonance in CDCl_3_ or DMSO-*d*_6_ solutions as the internal standard. The ^13^C-NMR signals were assigned on the basis of DEPT 135/90 spectra. The mass spectra were obtained on Finnigan MAT 312) (Germany) instrument using electron impact ionization (70 eV). The IR spectra were recorded on Bruker Alpha FT-IR instrument (Germany) with samples prepared as potassium bromide pellets. Thin-layer chromatography (TLC) was performed on precoated plates (silica gel 60 PF_254_), and zones were visualized with ultraviolet (UV) light. Elemental analyses for C, H, N were carried out with Elementar 306.

#### 3.1.1. Starting Materials: Acenaphthequinone, 1 Was Bought and Bought from Aldrich, whereas [2.2]Paracyclophane Was Commercially Available

##### Preparation of 2-Oxoacenaphthylidene Thiosemicarbazones **5a**–**e**

Compounds **5a**–**e** were synthesized by refluxing solutions of acenaphthequinone **1** (1.82 g, 10 mmol) in absolute EtOH (100 mL) containing triethylamine (0.5 mL) and different solutions of thiosemicarbazide derivatives **2a**–**e** for 3–5 h. The products were left to stand, and then collected by filtration after precipitation with ethanol. The resulting solid was recrystallized from the stated solvents to give yellow to orange crystals.

*(Z)-2-(2-Oxoacenaphthylen-1-(2H)-ylidene)hydrazinecarbothioamide* (**5a**). Yield: (1.58 g, 70%); orange crystals (EtOH); m.p.: 214–216 °C [50].

*(Z)-2-(2-Oxoacenaphthylen-1-(2H)-ylidene)-N-phenylhydrazinecarbothioamide* (**5b**). Yield: (2.65 g, 80%); yellow crystals (MeOH); m.p.: 208–210 °C [47].

*(Z)-N-Cyclohexyl-2-(2-oxoacenaphthylen-1-(2H)-ylidene)hydrazinecarbothioamide* (**5c**). Yield: (2.80 g, 75%); yellow crystals (CH_3_CN); m.p.: 202–204 °C. IR (KBr): *ν* = 3295 (NH’s), 2931 (Ali-CH), 1681 (CO), 1620 (C=N), 1609 (Ar-C=C) cm^−1^. ^1^H-NMR (400 MHz, DMSO-*d*_6_): *δ* = 1.17 (m, 1H, cyclic-CH), 1.33 (m, 2H, cyclic-CH_2_), 1.53 (m, 2H, cyclic-CH_2_), 1.66 (m, 1H, cyclic-CH), 1.79 (m, 2H, cyclic-CH_2_), 1.95 (m, 2H, cyclic-CH_2_), 4.23 (m, 1H, cyclic-CH), 7.85 (*t*, 1H, Ar-H, *J* = 7.9 Hz), 7.89 (*t*, 1H, Ar-H, *J* = 8.1 Hz), 8.09 (*d*, 1H, Ar-H, *J* = 8.2 Hz), 8.10 (*d*, 1H, Ar-H, *J* = 8.2 Hz), 8.15 (*d*, 1H, Ar-H, *J* = 8.3 Hz), 8.38 (d, 1H, Ar-H, *J* = 8.2 Hz), 8.97 (*d*, 1H, cyclohexyl-NH), 12.62 (s, 1H, = N-NH). ^13^C-NMR: (100 MHz, DMSO-*d*_6_): *δ* = 24.87, 25.05, 31.44 (cyclic-CH_2_), 53.84 (cyclic-CH), 118.61, 122.43, 127.05, 128.54, 128.79, 132.76 (Ar-CH), 129.88, 129.99, 130.43, 139.09 (Ar-C), 137.24 (C=N), 176.04 (C=S), 188.51 (C=O). MS: *m*/*z* (%) = 387 (M^+^, 100), 180 (53), 154 (59), 136 (40), 107 (13). Anal. Calcd. for C_19_H_19_N_3_OS (337.44); C, 67.63; H, 5.68; N, 12.45; S, 9.50. Found: C, 67.55; H, 5.75; N, 12.35; S, 9.57.

*(Z)-N-Allyl-2-(2-oxoacenaphthylen-1-(2H)-ylidene)hydrazinecarbothioamide* (**5d**). Yield: (2.18 g, 74%); light orange crystals (EtOH), m.p.: 190–191 °C [57].

*(Z)-N-Ethyl-2-(2-oxoacenaphthylen-1(2H)-ylidene)hydrazinecarbothioamide* (**5e**). Yield: (2.03 g, 72%); yellow crystals (EtOH), m.p.: 195–196 °C; [47].

##### Reactions of 2-Oxoacenaphthylidene Thiosemicarbazone Derivatives with **6a**

A mixture of 2-oxoacenaphthylidene thiosemicarbazones (**5a**–**e**, 1 mmol) in absolute ethanol (50 mL) was refluxed with **6a** for 20–30 min and the reaction was monitored by TLC analysis. A yellow precipitate of **7a**–**e** was formed, and then the reaction mixture was filtered and washed with a small amount of ethanol. The obtained precipitates were crystalized in ethanol to give yellow crystals of 1,3-thiazolidin-4-ones **7a**–**d**. In addition, the filtrate was left to stand at room temperature after separation of the precipitates **7a**–**e** and, in the case of **7d**, fine crystals of **8d** were collected after filtration).

*(Z)-Methyl 2-((Z)-4-oxo-2-((E)-(2-oxoacenaphthylen-1-(2H)-ylidene)hydrazono)thiazolidin-5-ylidene)acetate* (**7a**). Yield: (0.277 g, 76%); yellow crystals (EtOH); m.p.: 252–254 °C. IR (KBr): *ν* = 3489 (NH), 3150 (Ar-CH), 2960 (Ali-CH), 1715, 1696 (CO), 1623 (C=N), 1604 (Ar-C=C) cm^−1^. NMR see Table 1. MS, *m*/*z* (%) = 365 (M^+^, 10), 307 (35), 154 (100), 137 (65), 107 (17). Anal. Calcd. for C_18_H_11_N_3_O_4_S (365.36); C, 59.17; H, 3.03; N, 11.50; S, 8.78. Found: C, 59.26; H, 3.07; N, 11.57; S, 8.67.

*(Z)-Methyl 2-((Z)-4-oxo-2-((E)-(2-oxoacenaphthylen-1-(2H)-ylidene)hydrazono)-3-phenyl-thiazolidin-5-ylidene)acetate* (**7b**). Yield: (0.321 g, 73%); yellow crystals (EtOH); m.p.: 266–268 °C. IR (KBr): *ν* = 3120 (Ar-CH), 2952 (Ali-CH), 1713, 1690 (CO), 1630 (C=N), 1589 (Ar-C=C) cm^−1^. ^1^H-NMR (400 MHz, DMSO-*d*_6_): *δ* = 3.87 (s, 3H, OCH_3_), 6.95 (s, 1H, vinyl-CH), 7.49 (*t*, 1H, Ar-H, *J* = 7.7 Hz), 7.66 (m, 3H, Ar-H), 7.69 (m, 2H, Ar-H), 7.72 (*d*, 1H, Ar-H, *J* = 7.0 Hz), 7.86 (*t*, 1H, Ar-H, *J* = 8.0 Hz), 8.07 (*d*, 1H, Ar-H, *J* = 6.9 Hz), 8.13 (*d*, 1H, Ar-H, *J* = 8.3 Hz), 8.32 (*d*, 1H, Ar-H, *J* = 8.2 Hz). ^13^C-NMR (100 MHz, DMSO-*d*_6_): *δ* = 52.72 (OCH_3_), 116.42 (vinyl-CH), 125.72, 127.19, 128.08, 128.21, 128.29, 128.74, 129.15, 129.20, 129.41 (Ar-CH), 130.06, 130.86, 132.14, 134.13, 140.69 (Ar-C), 140.77 (thiazole-C5), 155.55, 163.52 (thiazole-C2, C = N), 165.77 (cyclic-C = O), 167.12 (ester C = O), 188.41 (C = O). MS, *m*/*z* (%) = 441 (M^+^, 30), 306 (35), 180 (17), 154 (100), 136 (63), 107 (20). Anal. Calcd. for C_24_H_15_N_3_O_4_S (441.46); C, 65.30; H, 3.42; N, 9.52; S, 7.26. Found: C, 65.23; H, 3.38; N, 9.57; S, 7.37.

*(Z)-Methyl 2-((Z)-3-cyclohexyl-4-oxo-2-((E)-(2-oxoacenaphthylen-1(2H)-ylidene)-hydrazono)-thiazolidin-5-ylidene)acetate* (**7c**). Yield: (0.322 g, 72%); yellow crystals (EtOH); m.p.: 284–286 °C. IR (KBr): *ν* = 3110 (Ar-CH), 2955 (Ali-CH), 1709, 1683 (CO), 1618 (C=N), 1591 (Ar-C=C) cm^−1^. ^1^H-NMR (400 MHz, DMSO-*d*_6_): *δ* = 1.35–1.37 (m, 2H, cyclic-CH_2_), 1.72–1.75 (m, 4H, cyclic-CH_2_), 1.86–1.88 (m, 4H, cyclic-CH_2_), 2.32 (m, 1H, cyclic-CH), 3.83 (s, 3H, OCH_3_), 6.83 (s, 1H, vinyl-CH), 7.85 (*t*, 1H, Ar-H, *J* = 8.0 Hz), 7.88 (*t*, 1H, Ar-H, *J* = 7.8 Hz), 7.93 (*d*, 1H, Ar-H, *J* = 7.1 Hz), 8.05 (*d*, 1H, Ar-H, *J* = 7.8 Hz), 8.13 (*d*, 1H, Ar-H, *J* = 8.3 Hz), 8.26 (*d*, 1H, Ar-H, *J* = 8.3 Hz). ^13^C-NMR (100 MHz, DMSO-*d*_6_): *δ* = 27.61, 30.72, 35.93 (cyclic-CH_2_), 53.07 (OCH_3_), 59.92 (cyclic-CH), 116.42 (vinyl-CH), 119.03, 121.72, 128.33, 128.44, 128.92, 132.10 (Ar-CH), 131.23, 133.44, 131.93, 139.50 (Ar-C), 142.19 (thiazole-C5), 152.18, 155.56 (thiazole-C2, C=N), 166.37 (cyclic-C=O), 166.69 (ester-C=O), 183.87 (C=O). MS, *m*/*z* (%) = 447 (M^+^, 5), 306 (35), 154 (100) 137 (67), 107 (17). Anal. Calcd. for C_24_H_21_N_3_O_4_S (447.51); C, 64.41; H, 4.73; N, 9.39; S, 7.17. Found: C, 64.32; H, 4.80; N, 9.34; S, 7.09.

*(Z)-Methyl 2-((Z)-3-allyl-4-oxo-2-((E)-(2-oxoacenaphthylen-1-(2H)-ylidene)hydrazono)-thiazolidin-5-ylidene)acetate* (**7d**). Yield: (0.303 g, 75%); yellow crystals (EtOH); m.p.: 218–220 °C. IR (KBr): *ν* = 3085 (Ar-CH), 2970 (Ali-CH), 1710, 1695 (CO), 1620 (C=N), 1609 (Ar-C=C) cm^−1^. ^1^H-NMR (400 MHz, DMSO-*d*_6_): *δ* = 3.86 (s, 3H, OCH_3_), 4.75 (m, 2H, allyl-CH_2_N), 5.34 (m, 2H, allyl-CH_2_=), 6.06 (m, 1H, allyl-CH=), 6.89 (s, 1H, vinyl-CH), 7.78 (*t*, 1H, Ar-H, *J* = 8.2 Hz), 7.81 (*t*, 1H, Ar-H, *J* = 7.9 Hz), 7.98 (*d*, 1H, Ar-H, *J* = 7.1 Hz), 8.04 (*d*, 1H, Ar-H, *J* = 7.8 Hz), 8.12 (*d*, 1H, Ar-H, *J* = 8.1 Hz), 8.58 (*d*, 1H, Ar-H, *J* = 8.1 Hz). ^13^C-NMR (100 MHz, DMSO-*d*_6_): *δ* = 47.72 (allyl-CH_2_N) 52.61 (OCH_3_), 115.28 (vinyl-CH), 118.58 (allyl-CH_2_=), 118.90, 121.62, 128.35, 128.70, 129.06, 131.91 (Ar-CH), 131.21, 131.63, 132.40, 138.9 (Ar-C), 134.85 (allyl-CH=), 142.23 (thiazole-C5), 151.91, 155.36 (thiazole-C2, C = N) 165.41 (cyclic-C=O), 166.54 (ester C=O), 183.93 (C=O). MS, *m*/*z* (%) = 405 (M^+^, 10), 306 (40), 289 (16), 154 (100), 137 (65), 107 (15). Anal. Calcd. for C_21_H_15_N_3_O_4_S (405.43); C, 62.21; H, 3.73; N, 10.36; S, 7.91. Found: C, 62.25; H, 3.68; N, 10.45; S, 7.97.

*(Z)-Methyl 2-((Z)-3-ethyl-4-oxo-2-((E)-(2-oxoacenaphthylen-1-(2H)-ylidene)hydrazono)-thiazolidin-5-ylidene)acetate* (**7e**). Yield: (0.290 g, 74%); yellow crystals (EtOH); m.p.: 200–202 °C. IR (KBr): *ν* = 3143 (Ar-CH), 2944 (Ali-CH), 1714, 1698 (CO), 1607 (C=N), 1590 (Ar-C=C) cm^−1^. ^1^H-NMR (400 MHz, DMSO-*d*_6_): *δ* = 1.36 (*t*, 3H, CH_3_, *J* = 6.72 Hz), 3.75 (*q*, 2H, CH_2_, *J* = 6.72 Hz), 3.82 (s, 3H, OCH_3_), 6.86 (s, 1H, vinyl-CH), 7.82 (*t*, 1H, Ar-H, *J* = 8.1 Hz), 8.01 (*d*, 1H, Ar-H, *J* = 7.2 Hz), 8.30 (*t*, 1H, Ar-H, *J* = 7.9 Hz), 8.45 (*d*, 1H, Ar-H, *J* = 7.8 Hz), 8.65 (*d*, 1H, Ar-H, *J* = 8.2 Hz), 8.36 (*d*, 1H, Ar-H, *J* = 8.0 Hz). ^13^C-NMR (100 MHz, DMSO-*d*_6_): *δ* = 12.29 (CH_3_), 31.5 (CH_2_), 52.52 (OCH_3_), 116.37 (vinyl-CH), 126.02, 127.46, 128.85, 129.66, 130.03, 131.30 (Ar-CH), 132.37, 135.30, 139.91, 140.74 (Ar-C), 144.24 (thiazole-C5), 154.92, 160.66 (thiazole-C2, C=N), 164.13 (cyclic-C=O), 165.66 (ester C=O), 187.49 (C=O). MS, *m*/*z* (%) = 393 (M^+^, 20), 306 (40), 153 (100), 136 (60), 107 (17). Anal. Calcd. for C_20_H_15_N_3_O_4_S (393.42); C, 61.06; H, 3.84; N, 10.68; S, 8.15. Found: C, 61.16; H, 3.81; N, 10.75; S, 8.20.

*Tetramethyl 5-(2-(((Z)-N-allyl-N′-((E)-2-oxoacenaphthylen-1-(2H)-ylidene)carbamo-hydrazonoyl)thio)-1,2,3-tris(methoxycarbonyl)cyclopropyl)-4-methoxy-7-oxabicyclo-[2.2.1]hepta-2,5-diene-1,2,3,6-tetracarboxylate* (**8d**). Yield: (0.086 g, 10%); orange crystals (EtOH); m.p.: 224–226 °C. IR (KBr): *ν* = 3401 (NH), 3100 (Ar-CH), 2953 (Ali-CH), 1717, 1698 (CO), 1623 (C=N), 1590 (Ar-C=C) cm^−1^. ^1^H-NMR (400 MHz, DMSO-*d*_6_): *δ* = 3.13 (s, 1H, cyclopropane-H), 3.40 (s, 3H, OCH_3_), 3.46, 3.55, 3.65, 3.73, 3.80, 3.85, 3.95 (s, 3H, OCH_3_), 4.40 (m, 2H, allyl-CH_2_N), 5.36 (m, 2H, allyl-CH_2_=), 6.08 (m, 1H, allyl-CH=), 7.77 (*t*, 1H, Ar-H, *J* = 8.2 Hz), 7.81 (*t*, 1H, Ar-H, *J* = 7.91 Hz), 8.00 (*d*, 1H, Ar-H, *J* = 7.1 Hz), 8.05 (*d*, 1H, Ar-H, *J* = 7.7 Hz), 8.11 (*d*, 1H, Ar-H, *J* = 8.3 Hz), 8.24 (*d*, 1H, Ar-H, *J* = 8.1 Hz), 9.40 (s, IH, NH-allyl). ^13^C-NMR (100 MHz, DMSO-*d*_6_): *δ* = 22.23, 24.90 (cyclopropane-C), 25.60 (cyclopropane-CH), 46.81 (allyl-CH_2_N), 52.00, 52.20, 52.60, 53.07, 53.19, 53.53, 53.65, 54.17 (OCH_3_), 118.19 (allyl-CH_2_=), 134.22 (allyl-CH=), 119.91, 121.80, 128.52, 128.29, 130.00, 132.32 (Ar-CH), 94.66, 121.66, 131.44, 134.58, 136.84, 138.01, 138.22, 138.93, 140.41, 142.82 (Ar-C), 152.63, 154.82 (C=N), 165.21, 167.53, 168.91, 171.68, 172.00, 173.51, 175.44 (ester-C=O), 185.16 (C=O). MS, *m*/*z* (%) = 863 (M^+^, 10), 805 (5), 612 (20), 307 (35), 154 (100), 137 (67), 107 (18). Anal. Calcd. for C_40_H_37_N_3_O_17_S (863.80); C, 55.62; H, 4.32; N, 4.86; S, 3.71. Found: C, 55.69; H, 4.27; N, 4.79; S, 3.61.

##### Preparation of 4-Acetyl-[2.2]Paracyclophanylidene-Thiosemicarbazones **22a,f**

4-Acetyl[2.2]paracyclophanylidene-thiosemicarbazones **22a,f** were synthesized via refluxing solutions of 4-acetyl[2.2]paracyclophane (**21**) (2.50 g, 10 mmol) in absolute ethanol (100 mL) containing glacial acetic acid (0.5 mL) and thiosemicarbazide **2a** (0.91 g, 10 mmol) or (1.06 g, 10 mmol) of thiocarbohydrazide **2f** for 6 h. Compounds **22a,f** were precipitated, then filtered and washed with dry ethanol to get the crude as colorless crystals. On the other hand, Bis-4-acetyl[2.2]-paracyclophanylidene-hydrazine-1-carbothiohydrazide (**23**) was obtained efficiently after treating **21** (2.50 g, 10 mmol) with **2f** (0.53 g, 5 mmol) in absolute EtOH (50 mL) under the same condition mentioned before (Scheme 7).

*(E)-2-(4-([2.2]Paracyclophanylethylidene)-1-carbothiohydrazide* (**22f**). Yield: (2.03 g, 60%); colorless crystals (EtOH/DMF); m.p.: 188–189 °C. IR (KBr): *ν* = 3450–3344 (NH), 2926–2853 (Ali-CH), 1642 (C=N), 1617 (Ar-C=C), 1354 (C=S) cm^−1^. ^1^H-NMR (400 MHz, DMSO-*d*_6_): *δ* = 2.28 (s, 3H, CH_3_), 2.79 (m, 2H, PC-CH_2_), 2.98 (m, 4H, 2 PC-CH_2_), 3.40 (m, 2H, PC-CH_2_), 4.99 (b, 2H, NH_2_), 6.49 (m, 6H, PC-H), 6.86 (s, 1H, PC-H), 9.37 (s, 1H, NH), 10.10 (s, 1H, = N-NH). ^13^C-NMR (100 MHz, DMSO-*d*_6_): *δ* = 18.04 (CH_3_), 34.38, 34.71, 34.84 (Pc-CH_2_), 130.56, 131.62, 132.23, 132.92, 132.50, 132.41, 135.61 (Pc-*C*H), 137.89, 138.97, 139.15, 139.45 (Pc-*C*), 150.68 (C = N), 177.17 (C = S). MS: *m*/*z* (%) = 338 [M^+^, 50], 306 (30), 153 (100), 135 (75), 106 (26). Anal. Calcd. for C_19_H_22_N_4_S (338.47); C, 67.42; H, 6.55; N, 16.55; S, 9.47. Found: C, 67.46; H, 6.50; N, 16.49; S, 9.53.

*Bis-4-acetyl[2.2]paracyclophanylidene-hydrazine-1-carbothiohydrazide* (**23**). Yield: (4.14 g, 88%); colorless crystals (EtOH/ DMF); m.p.: 224–226 °C. IR (KBr): *ν* = 3450–3344 (NH), 2926–2853 (Ali-CH), 1642 (C=N), 1617 (Ar-C=C), 1354 (C=S) cm^−1^. ^1^H-NMR (400 MHz, DMSO-*d*_6_): *δ* = 2.29 (s, 6H, 2CH_3_), 2.35 (m, 4H, PC-CH_2_), 2.97 (m, 8H, PC-CH_2_), 3.10 (m, 4H, PC-CH_2_), 6.48–6.56 (m, 4H, PC-H), 6.82 (s, 2H, PC-H), 7.08–7.09 (m, 8H, PC-H), 10.25 (s, 2H, 2=N-NH). ^13^C-NMR (100 MHz, DMSO-*d*_6_): *δ* = 18.56, 18.69 (CH_3_), 34.38, 34.59, 34.71, 34.83 (PC-CH_2_), 130.57, 131.60, 131.90, 132.23, 132.61, 133.20, 133.40 (PC-CH), 135.61, 135.75, 138.23, 139.15, 139.24 (PC-C), 150.93 (C=N), 177.32 (C = S). MS: *m*/*z* (%) = 570 [M^+^, 20], 363 (15), 351 (25), 307 (34), 154 (100), 106 (20), 98 (63). Anal. Calcd. for C_37_H_38_N_4_S (570.79); C, 77.86; H, 6.71; N, 9.82; S, 5.62. Found: C, 77.97; H, 6.63; N, 9.94; S, 5.75.

*2,2′-(1,4(1,4)-Dibenzenacyclohexaphane-1^2^,4^3^-diylbis(methane-ylylidene))bis-(N-cyclohexyl-hydrazine-1-carbothioamide)* (**25**). Yield: (4.59 g, 80%); colorless crystals (CH_3_CN); m.p.:142–143 °C. IR (KBr): *ν* = 3440 (NH), 2953 (Ali-CH), 1637 (C=N), 1610 (Ar-C=C), 1350 (C=S) cm^−1^. ^1^H-NMR (400 MHz, DMSO-*d*_6_): *δ* = 1.39 (m, 4H, cyclohexyl-CH_2_), 1.75 (m, 8H, cyclohexyl-CH_2_), 1.92 (m, 8H, cyclohexyl-CH_2_), 2.55 (m, 2H, cyclohexyl-CH), 2.90 (m, 4H, 2 PC-*C*H_2_), 3.23 (m, 2H, PC-*C*H_2_), 3.55 (m, 2H, PC-*C*H_2_), 6.52 (m, 4H, PC-H), 6.75 (s, 2H, PC-H), 7.55 (d, 2H, NH-cyclohexyl), 8.15 (s, 2H, CH=N), 11.20 (s, 2H, =N-NH). ^13^C-NMR (100 MHz, DMSO-*d*_6_): *δ* = 25.92, 32.50, 34.60 (cyclic-CH_2_), 32.58, 34.65, 34.88, (PC-CH_2_), 130.07, 132.40, 133.24 (PC-CH), 135.52, 138.14, 138.80, 139.90 (PC-C), 142.50 (CH=N), 175.06 (C = S). MS: *m*/*z* (%) = 574 [M^+^, 3], 417 (4), 261 (13), 258 (18), 157 (4), 141 (29), 129 (98), 83 (33), 56 (100). Anal. Calcd. for C_32_H_42_N_6_S_2_ (574.29); C, 66.86; H, 7.36; N, 14.62; S, 11.16. Found: C, 66.90; H, 7.42; N, 14.64; S, 11.10.

##### Reactions of 4-Acetyl[2.2]Paracyclophanylidene-Thiosemicarbazones, **22a**,**f** with **6a** and **6b**

A mixture of **22a,f** (1 mmol) in absolute ethanol (50 mL) was refluxed with either **6a** or **6b** for 3 h and the reaction was monitored by TLC. The precipitate of **26**–**29** was formed after concentration the solvent under vacuum, was filtered and washed with cold ethanol (15 mL) and recrystallized from methanol.

*Methyl 2-((Z)-2-(((E)-1-(1,4(1,4)-dibenzenacyclohexaphane-1^2^-yl)ethylidene)hydrazine-ylidene)-4-oxothiazolidin-5-yl)acetate* (**26**). Yield: (0.355 g, 82%); yellow crystals; m.p.: 256–258 °C. IR (KBr) *ν* = 3260 (NH), 3110 (Ar-CH), 2925 (Ali-CH), 1713, 1699 (CO), 1626 (C=N), 1600 (Ar-C=C) cm^−1^. ^1^H-NMR (400 MHz, DMSO-*d*_6_): *δ* = 2.37 (s, 3H, CH_3_), 2.89 (m, 8H, PC-CH_2_), 3.77 (s, 3H, OCH_3_), 6.54 (s, 1H, PC-H), 6.68 (m, 6H, PC-H), 6.74 (s, 1H, vinyl-CH), 12.89 (s, 1H, NH-thiazole). ^13^C-NMR (100 MHz, DMSO-*d*_6_): δ = 18.33 (CH_3_), 34.54, 34.60, 34.76, 35.02 (PC-CH_2_), 52.38 (OCH_3_), 113.92 (vinyl-CH), 130.81, 132.08, 132.37, 132.58, 132.70, 133.63, 135.82 (PC-CH), 138.23, 138.54, 138.97, 139.22 (PC-C), 143.54 (thiazole-C5), 159.05, 165.09 (thiazole-C2, C=N) 165.92 (cyclic-C=O), 165.99 (ester C=O). MS, *m*/*z* (%) = 433 (M^+^, 43), 306 (40), 289 (17), 153 (100) 136 (65), 106 (15). Anal. Calcd. for C_24_H_23_N_3_O_3_S (433.52); C, 66.49; H, 5.35; N, 9.69; S, 7.40. Found: C, 66.52; H, 5.46; N, 9.57; S, 7.33.

*Methyl 2-((Z)-3-amino-2-(((E)-1-(1,4(1,4)-dibenzenacyclohexaphane-1^2^-yl)ethylidene)-hydrazineylidene)-4-oxothiazolidin-5-yl)acetate* (**27**). Yield: (0.349 g, 78%); yellow crystals (CH_3_OH); m.p.: 260–262 °C. IR (KBr) *ν* = 3337 (NH_2_), 3050 (Ar-CH), 2923 (Ali-CH), 1737, 1700 (CO), 1630 (C=N), 1603 (Ar-C=C) cm^−1^. ^1^H-NMR (400 MHz, DMSO-*d*_6_): *δ* = 2.35 (s, 3H, CH_3_), 2.90 (m, 8H, PC-CH_2_), 3.80 (s, 3H, OCH_3_), 6.65 (m, 6H, PC-H), 6.55 (s, 1H, PC-H), 6.75 (s, 1H, vinyl-CH), 6.90 (br, s, 2H, NH_2_). ^13^C-NMR (100 MHz, DMSO-*d*_6_): *δ* = 18.61 (CH_3_), 34.51, 34.57, 34.75, 34.89 (PC-CH_2_), 52.61 (OCH_3_), 114.19 (vinyl-CH), 130.48, 131.52, 132.24, 132.38, 132.58, 133.34, 135.68 (PC-CH), 138.11, 138.86, 139.97, 139.17 (PC-C), 144.51 (thiazole-C5), 152.36, 161.06 (thiazole-C2) 165.90 (cyclic-C=O), 166.77 (ester C = O). MS, *m*/*z* (%) = 448 (M+, 7), 306 (30), 289 (15), 153 (100) 136 (64), 106 (20). Anal. Calcd. for C_24_H_24_N_4_O_3_S (448.54); C, 64.27; H, 5.39; N, 12.49; S, 7.15. Found: C, 64.39; H, 5.48; N, 12.37; S, 7.31.

*Ethyl 2-((Z)-2-(((E)-1-(1,4(1,4)-dibenzenacyclohexaphane-1^2^-yl)ethylidene)hydrazine-ylidene)-4-oxothiazolidin-5-yl)acetate* (**28**). Yield: (0.380, 85%); yellow plates (CH_3_OH); m.p. 200–202 °C. IR (KBr) *ν* = 3240 (NH), 3120 (Ar-CH), 2919 (Ali-CH), 1714, 1695 (CO), 1624 (C=N), 1592 (Ar-C=C) cm^−1^. ^1^H-NMR (DMSO-*d*_6_): *δ* = 1.27 (*t*, 3H, CH_3_, *J* = 7.1 Hz), 2.37 (s, 3H, CH_3_), 2.90–3.14 (m, 8H, PC-CH_2_), 4.24 (*q*, 2H, OCH_2_, *J* = 7.1 Hz), 6.56–6.61 (m, 6H, PC-H), 6.54 (s, 1H, PC-H), 6.70 (s, 1H, vinyl-CH), 12.90 (s, 1H, NH-thiazole). ^13^C-NMR (100 MHz, DMSO-*d*_6_): *δ* = 14.01, 18.36 (CH_3_), 34.53, 34.61, 34.75, 35.03 (PC-CH_2_), 61.25 (OCH_2_), 114.56 (vinyl-CH), 130.79, 132.09, 132.36, 132.58, 132.70, 133.70, 135.82 (PC-CH), 138.17, 138.55, 138.96, 139.22 (PC-C), 142.90 (thiazole-C5), 158.45, 165.27 (thiazole-C2, C=N), 165.32 (cyclic-C=O), 165.62 (ester C=O). MS, *m*/*z* (%) = 447 (M^+^, 62), 306 (32), 153 (100), 135 (65), 106 (20). Anal. Calcd. For C_25_H_25_N_3_O_3_S (447.55): C, 67.09; H, 5.63; N, 9.39; S, 7.16. Found: C, 67.12; H, 5.66; N, 9.45; S, 7.28.

*Ethyl 2-((Z)-3-Amino-2-(((E)-1-(1,4(1,4)-dibenzenacyclohexaphane-1^2^-yl)ethylidene)-hydrazine-ylidene)-4-oxothiazolidin-5-yl)acetate* (**29**). Yield: (0.374 g, 81%); yellow crystals (CH_3_OH); m.p. 174–175 °C. IR (KBr): *ν* = 3344 (NH), 3100 (Ar-CH), 2924 (Ali-CH), 1735, 1720 (CO), 1622 (C=N), 1607 (Ar-C=C) cm^−1^. ^1^H-NMR (400 MHz, DMSO-*d*_6_): *δ* = 1.28 (*t*, 3H, CH_3_, *J* = 7.2 Hz), 2.33 (s, 3H, CH_3_), 2.92–3.04 (m, 8H, PC-CH_2_), 4.27 (*q*, 2H, OCH_2_, *J* = 7.2 Hz), 6.68 (m, 6H, PC-H), 6.57 (s, 1H, PC-H), 6.72 (s, 1H, vinyl-CH), 10.09 (b, s, 2H, NH_2_). ^13^C-NMR (100 MHz, DMSO-*d*_6_): *δ* = 14.38 (CH_3_), 18.62 (CH_3_), 34.57, 34.66, 34.76, 35.12, (PC-CH_2_), 54.40 (OCH_3_), 62.0 (OCH_2_), 115.46 (vinyl-CH), 131.81, 132.18, 132.40, 132.60, 132.73, 135.72, 134.86 (PC-CH), 138.27, 138.60, 138.99, 139.25 (PC-C), 143.56 (thiazole-C5), 159.17, 166.02 (thiazole-C2, C=N), 165.95 (cyclic-C=O), 176.02 (ester C=O). MS, *m*/*z* (%) = 462 (M^+^, 6), 307 (25), 248 (40), 155 (27), 154 (95), 143 (100), 135 (65), 106 (20). Anal. Calcd. for C_25_H_26_N_4_O_3_S (462.56): C, 64.91; H, 5.67; N, 12.11; S, 6.93. Found: C, 65.01; H, 5.75; N, 12.16; S, 7.02.

### 3.2. Single Crystal X-ray Structure Determination of ***5a***, ***7d***, ***8d***, ***25*** and ***28***

A single crystal of **5a** was obtained by recrystallization from C_2_H_5_OH (ethanol), a single crystal of **7d** was obtained by recrystallization from C_2_H_5_OH (ethanol), a single crystal of **8d** was obtained from C_2_H_5_OH (ethanol), **25** from CH_3_CN (acetonitrile), and a single crystal of **28** was obtained acenaphthequinone recrystallization from CH_3_OH (methanol). The single-crystal X-ray analyses were carried out on a Bruker D8 Venture diffractometer with Photon100 or Photon II CPAD detector at 123K using Cu-Kα radiation (*λ* = 1.54178 Å). Direct Methods (SHELXS [58] or Dual Space Methods (SHELXT [59]) were used for structure solution and refinement was carried out using SHELXL-2014 (full-matrix least-squares on *F*^2^) [59]. Hydrogen atoms were refined using a riding model (H (N, O) free). Semi-empirical absorption corrections were applied.

**Compound 5a (SB1184_HY):** C_13_H_9_N_3_OS, Mr = 255.29 g mol^−1^, orange blocks, size 0.30 × 0.25 × 0.20 mm, monoclinic, space group P2_1_/c (no.14), *a* = 6.3357 (2) Ǻ, *b* = 18.2558 (7) Ǻ, *c* = 20.0737 (7) Ǻ, β = 97.327 (1)°, V = 2302.83 (14) Ǻ^3^, Z = 8, D_calcd_ = 1.473 Mg m^−3^, F (000) = 1056, μ (Cu-Kα) = 2.42 mm^−1^, Τ = 123 K, 19236 measured reflections (2θ_max_ = 144.2 °), 4510 independent reflections [R_int_ = 0.023], 343 parameters, 6 restraints, R_1_ [for 4468 I › 2σ (I)] = 0.030, wR^2^ (for all data) = 0.079, S = 1.06, largest diff. peak and hole = 0.37 e Ǻ^−3^/−0.31 e Ǻ^−3^. It is a polymorph of ***EBINOV*** (2-(2-oxoacenaphthylen-1(2*H*)-ylidene)hydrazinecarbothioamide methanol solvate) [60].

**Compound 7d (SB1186_HY):** C_21_H_15_N_3_O_4_S, Mr = 405.42 g mol^−1^, yellow blocks, size 0.12 × 0.06 × 0.04 mm, monoclinic, space group P2_1_/n (no.14), *a* = 7.0903 (2) Ǻ, *b* = 15.6914 (5) Ǻ, *c* = 16.4943 (5) Ǻ, β = 98.917 (1)°, V = 1812.92 (9) Ǻ^3^, Z = 4, D_calcd_ = 1.485 Mg m^−3^, F (000) = 840, μ (Cu-Kα) = 1.90 mm^−1^, Τ = 123 K, 18041 measured reflections (2θ_max_ = 144.4°), 3575 independent reflections [R_int_ = 0.024], 263 parameters, R_1_ [for 3402 I › 2σ (I)] = 0.029, w*R*^2^ (for all data) = 0.077, S = 1.06, largest diff. peak and hole = 0.28 e Ǻ^−3^/−0.30 e Ǻ^−3^.

**Compound 8d (SB1185_HY):** C_40_H_37_N_3_O_17_S, Mr = 863.78 g mol^−1^, orange plates, size 0.09 × 0.06 × 0.03 mm, triclinic, space group *P*-1 (no. 2), *a* = 12.8863 (5) Ǻ, *b* = 15.1023 (6) Ǻ, *c* = 22.3027 (8) Ǻ, α = 100.800 (2)°, β = 100.850 (2)°, γ = 104.460 (2)°, V = 4000.4 (3) Ǻ^3^, Z = 4, D_calcd_ = 1.434 Mg m^−3^, F (000) = 1800, μ (Cu-Kα) = 1.43 mm^−1^, Τ = 123 K, 71781 measured reflections (2θ_max_ = 144.4°), 15705 independent reflections [R_int_ = 0.039], 1113 parameters, 986 restraints, R_1_ [for 14,020 I › 2σ (I)] = 0.059, w*R*^2^ (for all data) = 0.148, S = 1.10, largest diff. peak and hole = 1.52 e Ǻ^−3^/−0.59 e Ǻ^−3^. In the 2nd molecule the vinyl moiety is disordered, disordered atoms refined isotropically. In the second molecule the oxacenaphthylene moiety shows high Uij-values, probably disordered, but the disorder is not resolved. Use of constraints (EADP) and restraints (SADI) for the refinement as well as a general RIGU restraint.

**Compound 25 (SB1014_HY):** C_32_H_42_N_6_S_2_ ∙ 0.5 C_2_H_5_N, Mr = 595.36 g mol^−1^, colorless blocks, size 0.40 × 0.18 × 0.06 mm, triclinic, space group *P*-1 (no.2), *a* = 13.7588 (6) Ǻ, *b* = 23.3483 (9) Ǻ, *c* = 23.3963 (9) Ǻ, α = 60.101 (2)°, β = 84.894 (3)°, γ = 84.516 (3)°, V = 6478.3 (5) Ǻ^3^, Z = 8, D_calcd_ = 1.221 Mg m^−3^, F (000) = 2552, μ (Cu-Kα) = 1.74 mm^−1^, Τ = 123 K, 67389 measured reflections (2θ_max_ = 145.4°), 24776 independent reflections [R_int_ = 0.067], 1485 parameters, 1254 restraints, R_1_ [for 16,926 I › 2σ (I)] = 0.115, w*R*^2^ (for all data) = 0.321, S = 1.01, largest diff. peak and hole = 1.33 e Ǻ^−3^/−0.79 e Ǻ^−3^. The compound was refined as a two-component pseudo merohedral twin. A general RIGU restraint is used for the refinement due to the bad quality of the data. Two solvent molecules acetonitrile per asymmetric unit disordered, the disordered atoms refined isotropically.

**Compound 28 (SB1183_HY):** C_25_H_25_N_3_O_3_S ∙ CH_3_OH, Mr = 479.58 g mol^−1^, yellow plates, size 0.16 × 0.14 × 0.02 mm, monoclinic, space group *P*2_1_/c (no,.14), *a* = 19.5995 (5) Ǻ, *b* = 7.4181 (2) Ǻ, *c* = 18.2170 (5) Ǻ, β = 112.833 (2)°, V = 2441.04 (12) Ǻ^3^, Z = 4, D_calcd_ = 1.305 Mg m^−3^, F (000) = 1016, μ (Cu-Kα) = 1.49 mm^−1^, Τ = 123 K, 25915 measured reflections (2θ_max_ = 144.6°), 4808 independent reflections [R_int_ = 0.052], 315 parameters, two restraints, *R*_1_ [for 4146 I › 2σ (I)] = 0.064, w*R*^2^ (for all data) = 0.155, S = 1.17, largest diff. peak and hole = 0.46 e Ǻ^−3^/−0.32 e Ǻ^−3^.

CCDC 1937480 (**5a**–**sb1184_hy**), 1937481 (**7d**–**sb1186_hy**), 1937482 (**8d**–**sb1185_hy**), 1937483 (**25**–**sb1014_hy**), and 1937484 (**28**–**sb1183_hy**) contain the Supplementary crystallographic data for this paper. These data can be obtained free of charge from The Cambridge Crystallographic Data Centre *via* www.ccdc.cam.ac.uk/data_request/cif (details in Supplementary Materials).

## 4. Conclusions

In conclusion, we prepared various derivatives of thiazolodinone derived by acenaphthequinone and [2.2]paracyclophane. We are able here to prove the structures of the obtained products via X-ray structure analysis. We could also, for the first time, get the X-ray structure analysis of the tetramer form resulting from DMAD.

## Figures and Tables

**Table 1:** NMR spectroscopic data of **7a**.

**^1^H-NMR:**	**^1^H-^1^H COSY:**		**Assignment:**
13.46 (b; 1H)			NH-3′
8.35 (d, *J* = 8.2; 1H)	7.86		H-5
8.20 (d, *J* = 8.2; 1H)	7.85		H-6
8.01 (d, *J* = 7.8; 1H)	7.86		H-3
7.99 (d, *J* = 7.0; 1H)	7.85		H-8
7.86 (“t”, *J* = 7.9; 1H)8.35, 8.01	H-4		
7.85 (“t”, *J* = 8.0; 1H)	8.20, 7.99		H-7
6.73 (s; 1H)			H-5a′
3.81 (s; 3H)			H-5c′
**^13^C NMR:**	**^1^H-^13^C HSQC: H-^13^C HMBC:**		**Assignment:**
183.77		8.01, *7.86*	C-2
166.01			C-4
165.78		6.73, 3.81	C-5′
154.23			C-2′
151.66		7.99	C-1
142.07		6.73	C-5′
139.71		8.35, 8.20, 8.01, 7.86, 7.85	C-9a
131.86	8.35	8.20, 8.01, 7.86	C-5
131.15, 131.05, 130.06		8.35, 8.20, 8.01, 7.86, 7.85	C-2a, 5a, 9
128.95, 128.59	7.86, 7.85	8.35, 7.99	C-4, 7
128.20	8.20	8.20	C-6
121.52	8.01	8.35	C-3
118.82	7.99	8.20	C-8
115.27	6.73	6.73, 3.81	C-5a′
52.49	3.81	3.81	C-5c′

## Supplementary Materials

The following are available online.
