# Supplementary material for: Functionalized 1,3-Thiazolidin-4-Ones from 2-Oxo-Acenaphthoquinylidene- and [2.2]Paracyclophanylidene-Thiosemicarbazones
Source: Molecules. 2019 Aug 23;24(17):3069. doi: 10.3390/molecules24173069 (PMC6749376; doi:10.3390/molecules24173069)
Supplement: Supplementary file 1 [file molecules-24-03069-s001.pdf]

molecules

MDPI

Functionalized 1,3-Thiazolidin-4-Ones from 2-Oxo-Acenaphthoquinylidene- and [2.2]Paracyclophanylidene-Thiosemicarbazones

Ashraf A. Aly, [1,*] Nasr K. Mohamed [1], Alaa A. Hassan [1], Kamal M. El-Shaieb [1], Maysa M. Makhlouf [1], Stefan Bräse [2,3,*], Martin Nieger [4] and Alan B. Brown [5]

[1] Chemistry Department, Faculty of Science, Minia University, El-Minia 61519, Egypt
[2] Institute of Organic Chemistry, Karlsruhe Institute of Technology, Fritz-Haber-Weg 6, 76131 Karlsruhe, Germany
[3] Institute of Toxicology and Genetics (ITG) Karlsruhe Institute of Technology (KIT) Hermann-von-Helmholtz-Platz 1, D-76344 Eggenstein-Leopoldshafen, Germany
[4] Department of Chemistry, University of Helsinki, P.O. Box 55, A. I. Virtasen aukio I, 00014 Helsinki, Finland
[5] Chemistry Program, Florida Institute of Technology, Melbourne, FL 32901, USA
* Correspondence: ashrafaly63@yahoo.com (A.A.A.); braese@kit.edu (S.B.)

SINGLE CRYSTAL X-RAY STRUCTURE DETERMINATION OF 5a, 7d, 18, 26 and 28

A single crystal of **5a** was obtained by recrystallization from C_2H_5OH (ethanol), a single crystal of **7d** was obtained by recrystallization from C_2H_5OH (ethanol), a single crystal of **18** was obtained from C_2H_5OH (ethanol), **26** from CH_3CN (acetonitrile) and a single crystal of **29** was obtained *via* recrystallization from CH_3OH (methanol). The single-crystal X-ray analysis were carried out on a Bruker D8 Venture diffractometer with Photon100 or Photon II CPAD detector at 123K using Cu-K radiation (= 1.54178 Å). Direct Methods (SHELXS [44] or Dual Space Methods (SHELXT [45]) were used for structure solution and refinement was carried out using SHELXL-2014 (full-matrix least-squares on F^2) [45]. Hydrogen atoms were refined using a riding model (H (N, O) free). Semi-empirical absorption corrections were applied.

Compound 5a (SB1184_HY, MY125): $C_{13}H_9N_3OS$, Mr = 255.29 g mol^{-1}, orange blocks, size 0.30 × 0.25 × 0.20 mm, monoclinic, space group P2$_1$/c (no.14), a = 6.3357 (2) Å, b = 18.2558 (7) Å, c = 20.0737 (7) Å, β = 97.327 (1)°, V = 2302.83 (14) Å^3, Z = 8, D_{calcd} = 1.473 Mg m^{-3}, F (000) = 1056, μ (Cu-Kα) = 2.42 mm^{-1}, T = 123 K, 19236 measured reflections ($2\theta_{max}$= 144.2 °), 4510 independent reflections [R_{int} = 0.023], 343 parameters, 6 restraints, R_1 [for 4468 I › 2σ (I)] = 0.030, wR^2 (for all data) = 0.079, S = 1.06, largest diff. peak and hole = 0.37 e Å^{-3}/- 0.31 e Å^{-3}. It is a polymorph of ***EBINOV*** (2-(2-oxoacenaphthylen-1(2H)-ylidene)hydrazinecarbothioamide methanol solvate) [46].

Compound 7d (SB1186_HY): $C_{21}H_{15}N_3O_4S$, Mr = 405.42 g mol^{-1}, yellow blocks, size 0.12 × 0.06 × 0.04 mm, monoclinic, space group P2$_1$/n (no.14), a = 7.0903 (2) Å, b = 15.6914 (5) Å, c = 16.4943 (5) Å, β = 98.917 (1)°, V = 1812.92 (9) Å^3, Z = 4, D_{calcd} = 1.485 Mg m^{-3}, F (000) = 840, μ (Cu-Kα) = 1.90 mm^{-1}, T = 123 K, 18041 measured reflections ($2\theta_{max}$= 144.4 °), 3575 independent reflections [R_{int} = 0.024], 263 parameters, R_1 [for 3402 I › 2σ (I)] = 0.029, wR^2 (for all data) = 0.077, S = 1.06, largest diff. peak and hole = 0.28 e Å^{-3}/- 0.30 e Å^{-3}.

Compound 8d (SB1185_HY): $C_{40}H_{37}N_3O_{17}S$, Mr = 863.78 g mol^{-1}, orange plates, size 0.09 × 0.06 × 0.03 mm, triclinic, space group *P*-1 (no.2), a = 12.8863 (5) Å, b = 15.1023 (6) Å, c = 22.3027 (8) Å, α = 100.800 (2)°, β = 100.850 (2)°, γ = 104.460 (2)°, V = 4000.4 (3) Å^3, Z = 4, D_{calcd} = 1.434 Mg m^{-3}, F (000) = 1800, μ (Cu-Kα) = 1.43 mm^{-1}, T = 123 K, 71781 measured reflections ($2\theta_{max}$= 144.4 °), 15705 independent reflections [R_{int} = 0.039], 1113 parameters, 986 restraints, R_1 [for 14020 I › 2σ (I)] = 0.059, wR^2 (for all data) = 0.148, S = 1.10, largest diff. peak and hole = 1.52 e Å^{-3}/- 0.59 e Å^{-3}. In the 2nd molecule the vinyl moiety is disordered, disordered atoms refined isotropically. In the 2nd molecule the oxacenaphthylene moiety shows high Uij-values, probably disordered, but the disorder is not resolved. Use of constraints (EADP) and restraints (SADI) for the refinement as well as a general RIGU restraint.

Compound 25 (SB1014_HY): $C_{32}H_{42}N_6S_2 \cdot 0.5\ C_2H_5N$, Mr = 595.36 g mol^{-1}, colourless blocks, size 0.40 × 0.18 × 0.06 mm, triclinic, space group *P*-1 (no.2), a = 13.7588 (6) Å, b = 23.3483 (9) Å, c = 23.3963 (9) Å, α = 60.101 (2)°, β = 84.894

(3)°, γ = 84.516 (3)°, V = 6478.3 (5) Å^3, Z = 8, D_{calcd} = 1.221 Mg m^{-3}, F (000) = 2552, μ (Cu-Kα) = 1.74 mm^{-1}, T = 123 K, 67389 measured reflections ($2\theta_{max}$= 145.4 °), 24776 independent reflections [R_{int} = 0.067], 1485 parameters, 1254 restraints, R_1 [for 16926 I › 2σ (I)] = 0.115, wR^2 (for all data) = 0.321, S = 1.01, largest diff. peak and hole = 1.33 e Å^{-3}/- 0.79 e Å^{-3}. Refined as a 2-component pseudo merohedral twin. A general RIGU restraint is used for the refinement due to the bad quality of the data. Two solvent molecules acetonitrile per asymmetric unit disordered, the disordered atoms refined isotropically.

Compound 28 (SB1183_HY): $C_{25}H_{25}N_3O_3S \cdot CH_3OH$, Mr = 479.58 g mol^{-1}, yellow plates, size 0.16 × 0.14 × 0.02 mm, monoclinic, space group *P*2$_1$/c (no,.14), a = 19.5995 (5) Å, b = 7.4181 (2) Å, c = 18.2170 (5) Å, β = 112.833 (2)°, V = 2441.04 (12) Å^3, Z = 4, D_{calcd} = 1.305 Mg m^{-3}, F (000) = 1016, μ (Cu-Kα) = 1.49 mm^{-1}, T = 123 K, 25915 measured reflections ($2\theta_{max}$= 144.6 °), 4808 independent reflections [R_{int} = 0.052], 315 parameters, 2 restraints, R_1 [for 4146 I › 2σ (I)] = 0.064, wR^2 (for all data) = 0.155, S = 1.17, largest diff. peak and hole = 0.46 e Å^{-3}/- 0.32 e Å^{-3}.

CCDC 1937480 (**5a – sb1184_hy**), 1937481 (**7d – sb1186_hy**), 1937482 (**8d – sb1185_hy**), 1937483 (**25 – sb1014_hy**), and 1937484 (**28 – sb1183_hy**) contain the supplementary crystallographic data for this paper. These data can be obtained free of charge from The Cambridge Crystallographic Data Centre via www.ccdc.cam.ac.uk/data_request/cif.

-3.

SUPPLEMENTARY DATA

CCDC 1937480 (**5a – sb1184_hy**), 1937481 (**7d – sb1186_hy**), 1937482 (**8d – sb1185_hy**), 1937483 (**25 – sb1014_hy**), and 1937484 (**28 – sb1183_hy**) contain the supplementary crystallographic data for this paper. These data can be obtained free of charge from The Cambridge Crystallographic Data Centre via www.ccdc.cam.ac.uk/data_request/cif.

Table. S1. Crystal data of **5a**.

$C_{13}H_9N_3OS$	*F*(000) = 1056
M_r = 255.29	D_x = 1.473 Mg m^{-3}
Monoclinic, *P*2$_1$/*c (no.14)*	Cu *K* radiation, = 1.54178 Å
a = 6.3357 (2) Å	Cell parameters from 9865 reflections
b = 18.2558 (7) Å	= 2.4–72.0°
c = 20.0737 (7) Å	= 2.42 mm^{-1}
= 97.327 (1)°	*T* = 123 K
V = 2302.83 (14) Å^3	Blocks, orange
Z = 8	0.30 × 0.25 × 0.20 mm

Table. S2. Data collection of **5a**.

Bruker D8 VENTURE diffractometer with PhotonII CPAD detector	4468 reflections with *I* > 2 (*I*)
Radiation source: INCOATEC microfocus sealed tube	R_{int} = 0.023
rotation in and , 1°, shutterless scans	$_{max}$ = 72.1°, $_{min}$ = 3.3°
Absorption correction: multi-scan *SADABS* (Sheldrick, 2014)	*h* = -7 7
T_{min} = 0.530, T_{max} = 0.638	*k* = -22 22
19236 measured reflections	*l* = -24 21
4510 independent reflections	

Table. S3. Refinement of **5a**.

Refinement on F^2	Primary atom site location: dual
Least-squares matrix: full	Secondary atom site location: difference Fourier map
$R[F^2 > 2\ (F^2)] = 0.030$	Hydrogen site location: difference Fourier map
$wR(F^2) = 0.079$	H atoms treated by a mixture of independent and constrained refinement
$S = 1.06$	$w = 1/[\ ^2(F_o^2) + (0.0375P)^2 + 1.2283P]$ where $P = (F_o^2 + 2F_c^2)/3$
4510 reflections	$(\ /\ )_{max} = 0.001$
343 parameters	$_{max} = 0.37$ e Å^{-3}
6 restraints	$_{min} = -0.31$ e Å^{-3}

Table. S4. Fractional atomic coordinates and isotropic or equivalent isotropic displacement parameters (Å^2) for (**5a**)

	x	y	z	U_{iso}*/U_{eq}
C1	0.7222 (2)	0.33454 (7)	0.49881 (6)	0.0177 (3)
C2	0.6352 (2)	0.29728 (7)	0.43321 (7)	0.0200 (3)
O2	0.71311 (15)	0.30201 (6)	0.38066 (5)	0.0263 (2)
C2A	0.4425 (2)	0.25714 (7)	0.44740 (7)	0.0201 (3)
C3	0.2991 (2)	0.21325 (8)	0.40886 (7)	0.0236 (3)
H3	0.3164	0.2019	0.3637	0.028*
C4	0.1244 (2)	0.18534 (8)	0.43870 (8)	0.0267 (3)
H4	0.0242	0.1546	0.4128	0.032*
C5	0.0950 (2)	0.20133 (7)	0.50415 (8)	0.0250 (3)
H5	-0.0245	0.1817	0.5222	0.030*
C5A	0.2410 (2)	0.24670 (7)	0.54487 (7)	0.0214 (3)
C6	0.2332 (2)	0.26934 (8)	0.61204 (7)	0.0247 (3)
H6	0.1171	0.2548	0.6347	0.030*
C7	0.3926 (2)	0.31218 (8)	0.64469 (7)	0.0246 (3)
H7	0.3842	0.3259	0.6899	0.030*
C8	0.5694 (2)	0.33679 (7)	0.61376 (7)	0.0219 (3)
H8	0.6784	0.3657	0.6377	0.026*
C8A	0.5772 (2)	0.31726 (7)	0.54797 (7)	0.0186 (3)
C8B	0.4149 (2)	0.27251 (7)	0.51454 (6)	0.0184 (3)
N9	0.88824 (17)	0.37528 (6)	0.51153 (5)	0.0184 (2)
N10	1.00624 (17)	0.38788 (6)	0.46058 (6)	0.0185 (2)
H10	0.974 (3)	0.3669 (9)	0.4215 (7)	0.022*
C11	1.18358 (19)	0.43087 (7)	0.47315 (6)	0.0174 (2)

S11	1.32420 (5)	0.44988 (2)	0.40919 (2)	0.01974 (9)
N12	1.23111 (18)	0.45515 (6)	0.53488 (6)	0.0199 (2)
H12A	1.346 (2)	0.4813 (9)	0.5447 (9)	0.030*
H12B	1.155 (3)	0.4414 (10)	0.5651 (8)	0.030*
C21	0.6772 (2)	0.54045 (7)	0.79929 (6)	0.0186 (3)
C22	0.7741 (2)	0.49843 (7)	0.86125 (6)	0.0198 (3)
O22	0.68972 (15)	0.44764 (5)	0.88733 (5)	0.0247 (2)
C22A	0.9837 (2)	0.53389 (7)	0.88213 (6)	0.0195 (3)
C23	1.1461 (2)	0.52085 (8)	0.93313 (7)	0.0227 (3)
H23	1.1366	0.4822	0.9643	0.027*
C24	1.3279 (2)	0.56673 (8)	0.93772 (7)	0.0249 (3)
H24	1.4389	0.5593	0.9736	0.030*
C25	1.3491 (2)	0.62170 (8)	0.89201 (7)	0.0240 (3)
H25	1.4750	0.6505	0.8964	0.029*
C25A	1.1853 (2)	0.63577 (7)	0.83856 (7)	0.0207 (3)
C26	1.1838 (2)	0.68911 (7)	0.78680 (7)	0.0239 (3)
H26	1.3049	0.7193	0.7847	0.029*
C27	1.0075 (2)	0.69706 (7)	0.73972 (7)	0.0236 (3)
H27	1.0091	0.7338	0.7063	0.028*
C28	0.8239 (2)	0.65284 (7)	0.73903 (7)	0.0209 (3)
H28	0.7030	0.6603	0.7066	0.025*
C28A	0.8255 (2)	0.59862 (7)	0.78693 (6)	0.0188 (3)
C28B	1.0042 (2)	0.59145 (7)	0.83629 (6)	0.0187 (3)
N29	0.49979 (17)	0.52922 (6)	0.76146 (5)	0.0197 (2)
N30	0.37572 (17)	0.47290 (6)	0.77707 (6)	0.0203 (2)
H30	0.413 (3)	0.4453 (9)	0.8126 (8)	0.024*
C31	0.1819 (2)	0.46333 (7)	0.73904 (7)	0.0197 (3)
S31	0.02017 (5)	0.39655 (2)	0.75854 (2)	0.02380 (10)
N32	0.13964 (19)	0.50915 (7)	0.68753 (6)	0.0244 (2)
H32A	0.011 (2)	0.5118 (11)	0.6693 (9)	0.037*
H32B	0.224 (3)	0.5465 (9)	0.6845 (10)	0.037*

Tabe S5. Atomic displacement parameters (Å^2) for (**5a**)

	U^{11}	U^{22}	U^{33}	U^{12}	U^{13}	U^{23}
C1	0.0152 (6)	0.0183 (6)	0.0195 (6)	0.0032 (5)	0.0016 (5)	0.0024 (5)
C2	0.0170 (6)	0.0209 (6)	0.0222 (6)	0.0022 (5)	0.0024 (5)	0.0008 (5)
O2	0.0229 (5)	0.0358 (6)	0.0212 (5)	-0.0020 (4)	0.0073 (4)	-0.0022 (4)
C2A	0.0187 (6)	0.0190 (6)	0.0227 (6)	0.0024 (5)	0.0025 (5)	0.0021 (5)
C3	0.0231 (7)	0.0217 (7)	0.0253 (7)	0.0004 (5)	0.0001 (5)	0.0000 (5)
C4	0.0224 (7)	0.0202 (6)	0.0359 (8)	-0.0025 (5)	-0.0022 (6)	0.0019 (6)
C5	0.0176 (6)	0.0210 (6)	0.0364 (8)	-0.0008 (5)	0.0034 (5)	0.0093 (6)
C5A	0.0180 (6)	0.0192 (6)	0.0273 (7)	0.0037 (5)	0.0037 (5)	0.0085 (5)
C6	0.0217 (7)	0.0258 (7)	0.0281 (7)	0.0051 (5)	0.0089 (5)	0.0105 (6)
C7	0.0281 (7)	0.0269 (7)	0.0199 (6)	0.0082 (6)	0.0072 (5)	0.0054 (5)
C8	0.0224 (6)	0.0214 (6)	0.0217 (7)	0.0038 (5)	0.0020 (5)	0.0024 (5)
C8A	0.0166 (6)	0.0177 (6)	0.0217 (6)	0.0036 (5)	0.0028 (5)	0.0041 (5)
C8B	0.0163 (6)	0.0161 (6)	0.0225 (6)	0.0035 (5)	0.0016 (5)	0.0051 (5)
N9	0.0157 (5)	0.0185 (5)	0.0212 (5)	0.0027 (4)	0.0036 (4)	0.0034 (4)
N10	0.0168 (5)	0.0208 (5)	0.0181 (5)	-0.0013 (4)	0.0034 (4)	-0.0003 (4)
C11	0.0143 (6)	0.0156 (6)	0.0217 (6)	0.0035 (5)	0.0006 (5)	0.0023 (5)
S11	0.01645 (16)	0.02434 (17)	0.01841 (16)	-0.00321 (11)	0.00219 (11)	0.00023 (11)
N12	0.0178 (5)	0.0226 (6)	0.0193 (5)	-0.0016 (4)	0.0028 (4)	0.0005 (4)
C21	0.0177 (6)	0.0194 (6)	0.0189 (6)	0.0020 (5)	0.0036 (5)	-0.0018 (5)
C22	0.0181 (6)	0.0221 (6)	0.0194 (6)	0.0026 (5)	0.0035 (5)	-0.0012 (5)
O22	0.0217 (5)	0.0262 (5)	0.0266 (5)	-0.0011 (4)	0.0041 (4)	0.0055 (4)
C22A	0.0187 (6)	0.0216 (6)	0.0188 (6)	0.0019 (5)	0.0043 (5)	-0.0030 (5)
C23	0.0219 (7)	0.0251 (7)	0.0209 (6)	0.0038 (5)	0.0023 (5)	-0.0009 (5)
C24	0.0194 (6)	0.0304 (7)	0.0239 (7)	0.0041 (5)	-0.0012 (5)	-0.0062 (6)
C25	0.0193 (6)	0.0257 (7)	0.0273 (7)	-0.0017 (5)	0.0033 (5)	-0.0089 (6)
C25A	0.0203 (6)	0.0205 (6)	0.0222 (6)	0.0003 (5)	0.0055 (5)	-0.0067 (5)
C26	0.0255 (7)	0.0206 (6)	0.0270 (7)	-0.0040 (5)	0.0090 (5)	-0.0055 (5)
C27	0.0315 (7)	0.0194 (6)	0.0215 (7)	0.0000 (5)	0.0097 (5)	-0.0019 (5)
C28	0.0243 (7)	0.0198 (6)	0.0189 (6)	0.0020 (5)	0.0033 (5)	-0.0027 (5)
C28A	0.0192 (6)	0.0200 (6)	0.0176 (6)	0.0010 (5)	0.0041 (5)	-0.0039 (5)
C28B	0.0188 (6)	0.0191 (6)	0.0186 (6)	0.0020 (5)	0.0046 (5)	-0.0046 (5)
N29	0.0183 (5)	0.0207 (5)	0.0206 (5)	0.0004 (4)	0.0042 (4)	-0.0014 (4)
N30	0.0176 (5)	0.0215 (5)	0.0212 (6)	-0.0009 (4)	0.0005 (4)	0.0021 (4)
C31	0.0178 (6)	0.0221 (6)	0.0196 (6)	0.0028 (5)	0.0035 (5)	-0.0051 (5)

S31	0.02086 (17)	0.02359 (18)	0.02657 (18)	-0.00380 (12)	0.00158 (13)	-0.00154 (12)
N32	0.0191 (6)	0.0302 (6)	0.0230 (6)	-0.0004 (5)	-0.0007 (4)	0.0033 (5)

Table.S6. Geometric parameters (Å, º) for (**5a**)

C1—N9	1.2873 (17)	C21—N29	1.2900 (17)
C1—C8A	1.4656 (17)	C21—C28A	1.4602 (18)
C1—C2	1.5216 (18)	C21—C22	1.5224 (18)
C2—O2	1.2234 (16)	C22—O22	1.2211 (17)
C2—C2A	1.4823 (18)	C22—C22A	1.4888 (18)
C2A—C3	1.3736 (19)	C22A—C23	1.3764 (19)
C2A—C8B	1.4092 (18)	C22A—C28B	1.4134 (19)
C3—C4	1.419 (2)	C23—C24	1.418 (2)
C3—H3	0.9500	C23—H23	0.9500
C4—C5	1.382 (2)	C24—C25	1.378 (2)
C4—H4	0.9500	C24—H24	0.9500
C5—C5A	1.420 (2)	C25—C25A	1.418 (2)
C5—H5	0.9500	C25—H25	0.9500
C5A—C8B	1.4063 (18)	C25A—C28B	1.4004 (19)
C5A—C6	1.417 (2)	C25A—C26	1.423 (2)
C6—C7	1.376 (2)	C26—C27	1.376 (2)
C6—H6	0.9500	C26—H26	0.9500
C7—C8	1.4211 (19)	C27—C28	1.415 (2)
C7—H7	0.9500	C27—H27	0.9500
C8—C8A	1.3750 (19)	C28—C28A	1.3791 (19)
C8—H8	0.9500	C28—H28	0.9500
C8A—C8B	1.4136 (18)	C28A—C28B	1.4120 (18)
N9—N10	1.3612 (15)	N29—N30	1.3549 (16)
N10—C11	1.3674 (17)	N30—C31	1.3719 (17)
N10—H10	0.873 (13)	N30—H30	0.880 (14)
C11—N12	1.3143 (17)	C31—N32	1.3302 (18)
C11—S11	1.6895 (13)	C31—S31	1.6710 (14)
N12—H12A	0.869 (14)	N32—H32A	0.853 (15)
N12—H12B	0.859 (14)	N32—H32B	0.873 (15)
N9—C1—C8A	123.88 (12)	N29—C21—C28A	123.16 (12)
N9—C1—C2	128.80 (12)	N29—C21—C22	129.14 (12)
C8A—C1—C2	107.31 (11)	C28A—C21—C22	107.69 (11)
O2—C2—C2A	129.30 (12)	O22—C22—C22A	129.28 (12)
O2—C2—C1	124.98 (12)	O22—C22—C21	125.44 (12)

C2A—C2—C1	105.72 (11)	C22A—C22—C21	105.26 (11)
C3—C2A—C8B	120.06 (12)	C23—C22A—C28B	119.41 (13)
C3—C2A—C2	133.00 (13)	C23—C22A—C22	133.58 (13)
C8B—C2A—C2	106.91 (11)	C28B—C22A—C22	107.01 (11)
C2A—C3—C4	117.87 (13)	C22A—C23—C24	118.04 (13)
C2A—C3—H3	121.1	C22A—C23—H23	121.0
C4—C3—H3	121.1	C24—C23—H23	121.0
C5—C4—C3	122.18 (13)	C25—C24—C23	122.32 (13)
C5—C4—H4	118.9	C25—C24—H24	118.8
C3—C4—H4	118.9	C23—C24—H24	118.8
C4—C5—C5A	120.90 (13)	C24—C25—C25A	120.77 (13)
C4—C5—H5	119.5	C24—C25—H25	119.6
C5A—C5—H5	119.5	C25A—C25—H25	119.6
C8B—C5A—C6	116.01 (13)	C28B—C25A—C25	115.96 (12)
C8B—C5A—C5	115.79 (13)	C28B—C25A—C26	116.26 (12)
C6—C5A—C5	128.19 (13)	C25—C25A—C26	127.78 (13)
C7—C6—C5A	120.43 (12)	C27—C26—C25A	120.19 (13)
C7—C6—H6	119.8	C27—C26—H26	119.9
C5A—C6—H6	119.8	C25A—C26—H26	119.9
C6—C7—C8	122.91 (13)	C26—C27—C28	122.77 (13)
C6—C7—H7	118.5	C26—C27—H27	118.6
C8—C7—H7	118.5	C28—C27—H27	118.6
C8A—C8—C7	117.67 (13)	C28A—C28—C27	118.00 (13)
C8A—C8—H8	121.2	C28A—C28—H28	121.0
C7—C8—H8	121.2	C27—C28—H28	121.0
C8—C8A—C8B	119.53 (12)	C28—C28A—C28B	119.34 (12)
C8—C8A—C1	133.88 (13)	C28—C28A—C21	133.83 (12)
C8B—C8A—C1	106.56 (11)	C28B—C28A—C21	106.81 (11)
C5A—C8B—C2A	123.15 (12)	C25A—C28B—C28A	123.34 (12)
C5A—C8B—C8A	123.41 (12)	C25A—C28B—C22A	123.45 (12)
C2A—C8B—C8A	113.43 (11)	C28A—C28B—C22A	113.19 (12)
C1—N9—N10	117.51 (11)	C21—N29—N30	118.17 (11)
N9—N10—C11	118.39 (11)	N29—N30—C31	118.19 (11)
N9—N10—H10	120.9 (11)	N29—N30—H30	121.1 (11)
C11—N10—H10	120.6 (11)	C31—N30—H30	120.5 (11)
N12—C11—N10	116.85 (12)	N32—C31—N30	115.45 (12)
N12—C11—S11	124.59 (10)	N32—C31—S31	124.90 (11)

N10—C11—S11	118.56 (10)	N30—C31—S31	119.65 (10)
C11—N12—H12A	118.9 (12)	C31—N32—H32A	117.1 (13)
C11—N12—H12B	119.1 (12)	C31—N32—H32B	118.7 (13)
H12A—N12—H12B	121.8 (17)	H32A—N32—H32B	119.2 (18)
N9—C1—C2—O2	-1.2 (2)	N29—C21—C22—O22	-3.8 (2)
C8A—C1—C2—O2	177.59 (13)	C28A—C21—C22—O22	177.35 (12)
N9—C1—C2—C2A	179.59 (13)	N29—C21—C22—C22A	177.53 (13)
C8A—C1—C2—C2A	-1.61 (13)	C28A—C21—C22—C22A	-1.32 (13)
O2—C2—C2A—C3	1.7 (3)	O22—C22—C22A—C23	2.5 (2)
C1—C2—C2A—C3	-179.19 (14)	C21—C22—C22A—C23	-178.94 (14)
O2—C2—C2A—C8B	-176.78 (13)	O22—C22—C22A—C28B	-178.29 (13)
C1—C2—C2A—C8B	2.37 (14)	C21—C22—C22A—C28B	0.31 (13)
C8B—C2A—C3—C4	0.71 (19)	C28B—C22A—C23—C24	0.52 (19)
C2—C2A—C3—C4	-177.56 (13)	C22—C22A—C23—C24	179.69 (13)
C2A—C3—C4—C5	0.4 (2)	C22A—C23—C24—C25	-2.1 (2)
C3—C4—C5—C5A	-0.3 (2)	C23—C24—C25—C25A	1.4 (2)
C4—C5—C5A—C8B	-0.89 (19)	C24—C25—C25A—C28B	0.76 (19)
C4—C5—C5A—C6	178.93 (13)	C24—C25—C25A—C26	-178.74 (13)
C8B—C5A—C6—C7	-1.90 (19)	C28B—C25A—C26—C27	2.63 (18)
C5—C5A—C6—C7	178.28 (13)	C25—C25A—C26—C27	-177.87 (13)
C5A—C6—C7—C8	0.9 (2)	C25A—C26—C27—C28	-1.4 (2)
C6—C7—C8—C8A	1.0 (2)	C26—C27—C28—C28A	-1.5 (2)
C7—C8—C8A—	-1.68 (19)	C27—C28—C28A—	2.93 (18)

C8B		C28B	
C7—C8—C8A—C1	176.12 (13)	C27—C28—C28A—C21	-175.06 (13)
N9—C1—C8A—C8	1.1 (2)	N29—C21—C28A—C28	1.1 (2)
C2—C1—C8A—C8	-177.75 (14)	C22—C21—C28A—C28	180.00 (14)
N9—C1—C8A—C8B	179.11 (12)	N29—C21—C28A—C28B	-177.11 (12)
C2—C1—C8A—C8B	0.25 (13)	C22—C21—C28A—C28B	1.83 (14)
C6—C5A—C8B—C2A	-177.79 (12)	C25—C25A—C28B—C28A	179.29 (12)
C5—C5A—C8B—C2A	2.05 (19)	C26—C25A—C28B—C28A	-1.15 (19)
C6—C5A—C8B—C8A	1.17 (18)	C25—C25A—C28B—C22A	-2.33 (19)
C5—C5A—C8B—C8A	-178.98 (12)	C26—C25A—C28B—C22A	177.23 (12)
C3—C2A—C8B—C5A	-2.0 (2)	C28—C28A—C28B—C25A	-1.67 (19)
C2—C2A—C8B—C5A	176.66 (12)	C21—C28A—C28B—C25A	176.81 (11)
C3—C2A—C8B—C8A	178.92 (12)	C28—C28A—C28B—C22A	179.80 (11)
C2—C2A—C8B—C8A	-2.40 (15)	C21—C28A—C28B—C22A	-1.72 (15)
C8—C8A—C8B—C5A	0.64 (19)	C23—C22A—C28B—C25A	1.71 (19)
C1—C8A—C8B—C5A	-177.70 (11)	C22—C22A—C28B—C25A	-177.66 (12)
C8—C8A—C8B—C2A	179.70 (12)	C23—C22A—C28B—C28A	-179.75 (12)
C1—C8A—C8B—C2A	1.35 (15)	C22—C22A—C28B—C28A	0.87 (15)
C8A—C1—N9—N10	-178.71 (11)	C28A—C21—N29—N30	179.01 (11)
C2—C1—N9—N10	-0.10 (19)	C22—C21—N29—N30	0.3 (2)
C1—N9—N10—C11	-179.83 (11)	C21—N29—N30—C31	176.57 (11)
N9—N10—C11—N12	2.17 (17)	N29—N30—C31—N32	3.30 (17)

N9—N10—C11—S11	-177.22 (9)	N29—N30—C31—S31	-177.61 (9)

Table. S7. Hydrogen-bond geometry (Å, º) for (**5a**)

D—H···*A*	*D*—H	H···*A*	*D*···*A*	*D*—H···*A*
N10—H10···O2	0.87 (1)	2.11 (2)	2.7785 (15)	133 (1)
N12—H12*B*···N9	0.86 (1)	2.24 (2)	2.6086 (16)	106 (1)
N12—H12*A*···S11^{i}	0.87 (1)	2.51 (2)	3.3760 (12)	171 (2)
N30—H30···O22	0.88 (1)	2.16 (2)	2.8189 (15)	132 (1)
N32—H32*B*···N29	0.87 (2)	2.20 (2)	2.5844 (16)	106 (1)
N32—H32*A*···S11^{ii}	0.85 (2)	2.57 (2)	3.3927 (12)	161 (2)

Symmetry codes: (i) -*x*+3, -*y*+1, -*z*+1; (ii) -*x*+1, -*y*+1, -*z*+1.

Table. S8. Crystal data of **7d**.

$C_{21}H_{15}N_3O_4S$	*F*(000) = 840
M_r = 405.42	D_x = 1.485 Mg m^{-3}
Monoclinic, *P*2$_1$/*n (no.14)*	Cu *K* radiation, = 1.54178 Å
a = 7.0903 (2) Å	Cell parameters from 9959 reflections
b = 15.6914 (5) Å	= 3.9–72.1°
c = 16.4943 (5) Å	= 1.90 mm^{-1}
= 98.917 (1)°	*T* = 123 K
V = 1812.92 (9) Å^3	Blocks, yellow
Z = 4	0.12 × 0.06 × 0.04 mm

Table S9. Data collection of **7d**.

Bruker D8 VENTURE diffractometer with PhotonII CPAD detector	3402 reflections with *I* > 2 (*I*)
Radiation source: INCOATEC microfocus sealed tube	R_{int} = 0.024
rotation in and , 1°, shutterless scans	$_{max}$ = 72.2°, $_{min}$ = 3.9°
Absorption correction: multi-scan *SADABS* (Sheldrick, 2014)	*h* = -8 8
T_{min} = 0.852, T_{max} = 0.929	*k* = -18 19
18041 measured reflections	*l* = -20 20
3575 independent reflections	

Table S10. Refinement of **7d**.

Refinement on F^2	Primary atom site location: dual
Least-squares matrix: full	Secondary atom site location: difference Fourier map
$R[F^2 > 2\ (F^2)] = 0.029$	Hydrogen site location: difference Fourier map
$wR(F^2) = 0.077$	H-atom parameters constrained
$S = 1.06$	$w = 1/[\ ^2(F_o^2) + (0.0384P)^2 + 0.7452P]$ where $P = (F_o^2 + 2F_c^2)/3$
3575 reflections	$(\ /\ )_{max} = 0.001$
263 parameters	$_{max} = 0.28$ e Å^{-3}
0 restraints	$_{min} = -0.30$ e Å^{-3}

Table S11. Fractional atomic coordinates and isotropic or equivalent isotropic displacement parameters (Å^2) for (**7d**)

	x	*y*	*z*	U_{iso}*/U_{eq}
C1	0.70416 (16)	0.65573 (8)	0.30930 (7)	0.0180 (2)
O1	0.64725 (13)	0.60857 (6)	0.25234 (5)	0.0250 (2)
C2	0.74693 (16)	0.62987 (7)	0.40001 (7)	0.0171 (2)
C3	0.81358 (16)	0.70651 (7)	0.44723 (7)	0.0175 (2)
C4	0.86958 (17)	0.72403 (8)	0.52939 (7)	0.0210 (2)
H4	0.8702	0.6806	0.5695	0.025*
C5	0.92619 (19)	0.80823 (8)	0.55267 (8)	0.0251 (3)
H5	0.9657	0.8205	0.6091	0.030*
C6	0.92587 (19)	0.87284 (8)	0.49621 (8)	0.0253 (3)
H6	0.9654	0.9284	0.5143	0.030*
C7	0.86702 (17)	0.85737 (8)	0.41112 (8)	0.0202 (2)
C8	0.85792 (17)	0.91639 (8)	0.34555 (8)	0.0219 (3)
H8	0.8933	0.9741	0.3568	0.026*
C9	0.79830 (17)	0.89110 (8)	0.26542 (8)	0.0211 (3)
H9	0.7936	0.9320	0.2227	0.025*
C10	0.74407 (16)	0.80607 (8)	0.24507 (7)	0.0193 (2)
H10	0.7046	0.7896	0.1896	0.023*
C11	0.75003 (16)	0.74793 (8)	0.30771 (7)	0.0175 (2)
C12	0.81150 (16)	0.77348 (7)	0.38949 (7)	0.0172 (2)
N13	0.72606 (14)	0.55155 (6)	0.42093 (6)	0.0183 (2)
N14	0.77915 (14)	0.53895 (6)	0.50510 (6)	0.0186 (2)
C15	0.75204 (16)	0.46094 (7)	0.52497 (7)	0.0169 (2)
S16	0.65737 (4)	0.37838 (2)	0.45817 (2)	0.01713 (9)
C17	0.68350 (16)	0.30689 (8)	0.54051 (7)	0.0186 (2)
C18	0.76070 (17)	0.35003 (8)	0.61963 (7)	0.0201 (2)

O18	0.78913 (15)	0.31661 (6)	0.68652 (5)	0.0304 (2)
N19	0.79839 (14)	0.43453 (6)	0.60489 (6)	0.0182 (2)
C20	0.88356 (18)	0.49155 (8)	0.67152 (7)	0.0215 (3)
H20A	0.9712	0.5318	0.6499	0.026*
H20B	0.9598	0.4572	0.7152	0.026*
C21	0.73680 (18)	0.54129 (8)	0.70801 (7)	0.0221 (3)
H21	0.6255	0.5124	0.7187	0.027*
C22	0.75430 (19)	0.62300 (8)	0.72599 (8)	0.0258 (3)
H22A	0.8642	0.6534	0.7159	0.031*
H22B	0.6570	0.6515	0.7490	0.031*
C23	0.64373 (17)	0.22373 (8)	0.53725 (7)	0.0212 (3)
H23	0.6617	0.1908	0.5862	0.025*
C24	0.57213 (18)	0.18290 (8)	0.45819 (8)	0.0217 (3)
O24	0.54571 (14)	0.22057 (6)	0.39356 (5)	0.0270 (2)
O25	0.54143 (15)	0.09940 (6)	0.46641 (6)	0.0294 (2)
C25	0.4707 (3)	0.05586 (9)	0.39007 (9)	0.0377 (4)
H25A	0.3516	0.0827	0.3643	0.057*
H25B	0.4470	-0.0042	0.4014	0.057*
H25C	0.5659	0.0597	0.3530	0.057*

Table. S12. Atomic displacement parameters (Å²) for (**7d**)

	U^{11}	U^{22}	U^{33}	U^{12}	U^{13}	U^{23}
C1	0.0175 (5)	0.0179 (6)	0.0189 (6)	0.0020 (4)	0.0035 (4)	-0.0002 (4)
O1	0.0337 (5)	0.0204 (4)	0.0200 (4)	-0.0027 (4)	0.0008 (4)	-0.0030 (3)
C2	0.0168 (5)	0.0166 (6)	0.0184 (6)	0.0017 (4)	0.0036 (4)	-0.0015 (4)
C3	0.0175 (5)	0.0149 (5)	0.0205 (6)	0.0017 (4)	0.0039 (4)	-0.0001 (4)
C4	0.0248 (6)	0.0185 (6)	0.0192 (6)	0.0016 (5)	0.0022 (5)	0.0005 (5)
C5	0.0327 (7)	0.0218 (6)	0.0196 (6)	0.0005 (5)	0.0002 (5)	-0.0043 (5)
C6	0.0321 (7)	0.0160 (6)	0.0267 (7)	-0.0025 (5)	0.0012 (5)	-0.0045 (5)
C7	0.0202 (6)	0.0161 (6)	0.0242 (6)	0.0007 (4)	0.0032 (5)	-0.0012 (5)
C8	0.0217 (6)	0.0149 (6)	0.0290 (6)	-0.0003 (4)	0.0040 (5)	0.0008 (5)
C9	0.0189 (6)	0.0199 (6)	0.0246 (6)	0.0018 (5)	0.0040 (5)	0.0058 (5)
C10	0.0175 (5)	0.0214 (6)	0.0191 (6)	0.0011 (4)	0.0030 (4)	0.0011 (5)
C11	0.0151 (5)	0.0176 (6)	0.0201 (6)	0.0016 (4)	0.0037 (4)	-0.0005 (4)
C12	0.0151 (5)	0.0170 (6)	0.0195 (6)	0.0019 (4)	0.0031 (4)	-0.0005 (4)
N13	0.0203 (5)	0.0170 (5)	0.0179 (5)	0.0013 (4)	0.0034 (4)	0.0008 (4)
N14	0.0209 (5)	0.0168 (5)	0.0180 (5)	0.0010 (4)	0.0026 (4)	0.0001 (4)

C15	0.0151 (5)	0.0176 (6)	0.0181 (5)	0.0027 (4)	0.0032 (4)	-0.0011 (4)
S16	0.02140 (16)	0.01454 (15)	0.01502 (15)	0.00047 (10)	0.00149 (11)	0.00081 (10)
C17	0.0182 (5)	0.0200 (6)	0.0177 (5)	0.0027 (4)	0.0036 (4)	0.0023 (4)
C18	0.0212 (6)	0.0193 (6)	0.0199 (6)	0.0033 (5)	0.0033 (5)	0.0020 (5)
O18	0.0460 (6)	0.0260 (5)	0.0177 (4)	0.0022 (4)	-0.0003 (4)	0.0050 (4)
N19	0.0198 (5)	0.0181 (5)	0.0163 (5)	0.0016 (4)	0.0008 (4)	-0.0006 (4)
C20	0.0222 (6)	0.0231 (6)	0.0178 (6)	0.0013 (5)	-0.0016 (4)	-0.0039 (5)
C21	0.0208 (6)	0.0249 (6)	0.0209 (6)	-0.0014 (5)	0.0038 (5)	-0.0020 (5)
C22	0.0227 (6)	0.0270 (7)	0.0280 (7)	-0.0020 (5)	0.0048 (5)	-0.0066 (5)
C23	0.0246 (6)	0.0190 (6)	0.0204 (6)	0.0008 (5)	0.0049 (5)	0.0034 (5)
C24	0.0239 (6)	0.0178 (6)	0.0245 (6)	-0.0010 (5)	0.0067 (5)	-0.0002 (5)
O24	0.0358 (5)	0.0236 (5)	0.0212 (4)	-0.0035 (4)	0.0034 (4)	0.0022 (4)
O25	0.0460 (6)	0.0177 (4)	0.0243 (5)	-0.0049 (4)	0.0049 (4)	-0.0020 (4)
C25	0.0618 (10)	0.0232 (7)	0.0272 (7)	-0.0057 (7)	0.0043 (7)	-0.0079 (6)

Table. S13. Geometric parameters (Å, º) for (**7d**)

C1—O1	1.2148 (15)	C15—N19	1.3723 (15)
C1—C11	1.4840 (16)	C15—S16	1.7645 (12)
C1—C2	1.5345 (16)	S16—C17	1.7492 (12)
C2—N13	1.2912 (16)	C17—C23	1.3344 (17)
C2—C3	1.4704 (16)	C17—C18	1.4965 (17)
C3—C4	1.3795 (17)	C18—O18	1.2100 (15)
C3—C12	1.4167 (16)	C18—N19	1.3816 (16)
C4—C5	1.4164 (18)	N19—C20	1.4722 (15)
C4—H4	0.9500	C20—C21	1.4995 (17)
C5—C6	1.3764 (19)	C20—H20A	0.9900
C5—H5	0.9500	C20—H20B	0.9900
C6—C7	1.4217 (18)	C21—C22	1.3174 (18)
C6—H6	0.9500	C21—H21	0.9500
C7—C12	1.4042 (17)	C22—H22A	0.9500
C7—C8	1.4178 (17)	C22—H22B	0.9500
C8—C9	1.3818 (18)	C23—C24	1.4709 (17)
C8—H8	0.9500	C23—H23	0.9500
C9—C10	1.4145 (17)	C24—O24	1.2079 (16)
C9—H9	0.9500	C24—O25	1.3385 (16)
C10—C11	1.3740 (17)	O25—C25	1.4518 (16)
C10—H10	0.9500	C25—H25A	0.9800

C11—C12	1.4100 (16)	C25—H25B	0.9800
N13—N14	1.3950 (14)	C25—H25C	0.9800
N14—C15	1.2892 (16)		
O1—C1—C11	128.88 (11)	N14—C15—N19	120.53 (11)
O1—C1—C2	125.78 (11)	N14—C15—S16	126.55 (9)
C11—C1—C2	105.33 (9)	N19—C15—S16	112.92 (8)
N13—C2—C3	132.65 (11)	C17—S16—C15	90.10 (6)
N13—C2—C1	119.93 (10)	C23—C17—C18	121.60 (11)
C3—C2—C1	107.39 (10)	C23—C17—S16	126.84 (10)
C4—C3—C12	118.94 (11)	C18—C17—S16	111.55 (9)
C4—C3—C2	134.69 (11)	O18—C18—N19	124.55 (12)
C12—C3—C2	106.35 (10)	O18—C18—C17	125.86 (12)
C3—C4—C5	118.59 (11)	N19—C18—C17	109.59 (10)
C3—C4—H4	120.7	C15—N19—C18	115.77 (10)
C5—C4—H4	120.7	C15—N19—C20	122.89 (10)
C6—C5—C4	122.20 (12)	C18—N19—C20	121.33 (10)
C6—C5—H5	118.9	N19—C20—C21	112.71 (10)
C4—C5—H5	118.9	N19—C20—H20A	109.0
C5—C6—C7	120.78 (11)	C21—C20—H20A	109.0
C5—C6—H6	119.6	N19—C20—H20B	109.0
C7—C6—H6	119.6	C21—C20—H20B	109.0
C12—C7—C8	116.15 (11)	H20A—C20—H20B	107.8
C12—C7—C6	116.02 (11)	C22—C21—C20	123.36 (12)
C8—C7—C6	127.83 (11)	C22—C21—H21	118.3
C9—C8—C7	120.87 (11)	C20—C21—H21	118.3
C9—C8—H8	119.6	C21—C22—H22A	120.0
C7—C8—H8	119.6	C21—C22—H22B	120.0
C8—C9—C10	121.97 (11)	H22A—C22—H22B	120.0
C8—C9—H9	119.0	C17—C23—C24	120.23 (11)
C10—C9—H9	119.0	C17—C23—H23	119.9
C11—C10—C9	118.18 (11)	C24—C23—H23	119.9
C11—C10—H10	120.9	O24—C24—O25	124.27 (12)
C9—C10—H10	120.9	O24—C24—C23	123.65 (12)
C10—C11—C12	119.97 (11)	O25—C24—C23	112.08 (11)
C10—C11—C1	132.79 (11)	C24—O25—C25	114.36 (10)
C12—C11—C1	107.24 (10)	O25—C25—H25A	109.5

C7—C12—C11	122.85 (11)	O25—C25—H25B	109.5
C7—C12—C3	123.46 (11)	H25A—C25—H25B	109.5
C11—C12—C3	113.68 (10)	O25—C25—H25C	109.5
C2—N13—N14	112.12 (10)	H25A—C25—H25C	109.5
C15—N14—N13	111.08 (10)	H25B—C25—H25C	109.5
O1—C1—C2—N13	1.25 (18)	C2—C3—C12—C7	-179.71 (11)
C11—C1—C2—N13	-177.88 (10)	C4—C3—C12—C11	-179.41 (10)
O1—C1—C2—C3	179.52 (11)	C2—C3—C12—C11	-0.30 (13)
C11—C1—C2—C3	0.39 (12)	C3—C2—N13—N14	-0.39 (18)
N13—C2—C3—C4	-3.2 (2)	C1—C2—N13—N14	177.37 (9)
C1—C2—C3—C4	178.83 (13)	C2—N13—N14—C15	178.15 (10)
N13—C2—C3—C12	177.89 (12)	N13—N14—C15—N19	178.82 (9)
C1—C2—C3—C12	-0.07 (12)	N13—N14—C15—S16	-1.00 (14)
C12—C3—C4—C5	-1.12 (18)	N14—C15—S16—C17	179.37 (11)
C2—C3—C4—C5	-179.91 (13)	N19—C15—S16—C17	-0.46 (9)
C3—C4—C5—C6	0.4 (2)	C15—S16—C17—C23	-177.73 (12)
C4—C5—C6—C7	0.3 (2)	C15—S16—C17—C18	1.78 (9)
C5—C6—C7—C12	-0.25 (18)	C23—C17—C18—O18	-2.2 (2)
C5—C6—C7—C8	179.85 (13)	S16—C17—C18—O18	178.29 (11)
C12—C7—C8—C9	-0.33 (17)	C23—C17—C18—N19	176.84 (11)
C6—C7—C8—C9	179.57 (12)	S16—C17—C18—N19	-2.71 (12)
C7—C8—C9—C10	-0.02 (19)	N14—C15—N19—C18	179.01 (11)
C8—C9—C10—C11	0.62 (18)	S16—C15—N19—C18	-1.15 (13)
C9—C10—C11—C12	-0.85 (17)	N14—C15—N19—C20	-1.19 (17)
C9—C10—C11—C1	179.87 (11)	S16—C15—N19—C20	178.65 (8)
O1—C1—C11—C10	-0.3 (2)	O18—C18—N19—C15	-178.53 (12)
C2—C1—C11—C10	178.79 (12)	C17—C18—N19—C15	2.45 (14)
O1—C1—C11—C12	-179.66 (12)	O18—C18—N19—C20	1.67 (19)
C2—C1—C11—C12	-0.56 (12)	C17—C18—N19—C20	-177.35 (10)
C8—C7—C12—C11	0.09 (17)	C15—N19—C20—C21	85.63 (14)

C6—C7—C12—C11	-179.83 (11)	C18—N19—C20—C21	-94.58 (13)
C8—C7—C12—C3	179.44 (11)	N19—C20—C21—C22	-136.68 (13)
C6—C7—C12—C3	-0.48 (17)	C18—C17—C23—C24	-178.60 (11)
C10—C11—C12—C7	0.52 (17)	S16—C17—C23—C24	0.87 (18)
C1—C11—C12—C7	179.97 (11)	C17—C23—C24—O24	0.00 (19)
C10—C11—C12—C3	-178.89 (10)	C17—C23—C24—O25	179.52 (11)
C1—C11—C12—C3	0.56 (13)	O24—C24—O25—C25	-0.42 (19)
C4—C3—C12—C7	1.19 (18)	C23—C24—O25—C25	-179.94 (12)

Table S14. Hydrogen-bond geometry (Å, º) for (**7d**).

D—H···*A*	*D*—H	H···*A*	*D*···*A*	*D*—H···*A*
C25—H25*B*···O25^{i}	0.98	2.63	3.4073 (18)	136

Symmetry code: (i) -*x*+1, -*y*, -*z*+1.

Table S15. Crystal data of **18**.

$C_{40}H_{37}N_3O_{17}S$	*Z* = 4
M_r = 863.78	*F*(000) = 1800
Triclinic, *P*-1 (no.2)	D_x = 1.434 Mg m^{-3}
a = 12.8863 (5) Å	Cu *K* radiation, = 1.54178 Å
b = 15.1023 (6) Å	Cell parameters from 9899 reflections
c = 22.3027 (8) Å	= 3.2–72.1°
= 100.800 (2)°	= 1.43 mm^{-1}
= 100.850 (2)°	*T* = 123 K
= 104.460 (2)°	Plates, orange
V = 4000.4 (3) Å^3	0.09 × 0.06 × 0.03 mm

Table S16. Data collection of **18**.

Bruker D8 VENTURE diffractometer with PhotonII CPAD detector	14020 reflections with *I* > 2 (*I*)
Radiation source: INCOATEC microfocus sealed tube	R_{int} = 0.039
rotation in and , 1°, shutterless scans	$_{max}$ = 72.2°, $_{min}$ = 2.1°
Absorption correction: multi-scan *SADABS* (Sheldrick, 2014)	*h* = -15 15
T_{min} = 0.886, T_{max} = 0.971	*k* = -18 18
71781 measured reflections	*l* = -27 27

15705 independent reflections	

Table S17. Refinement of **18**.

Refinement on F^2	Primary atom site location: dual
Least-squares matrix: full	Secondary atom site location: difference Fourier map
$R[F^2 > 2\ (F^2)] = 0.059$	Hydrogen site location: mixed
$wR(F^2) = 0.148$	H atoms treated by a mixture of independent and constrained refinement
$S = 1.10$	$w = 1/[\ ^2(F_o^2) + (0.0559P)^2 + 5.9985P]$<br>where $P = (F_o^2 + 2F_c^2)/3$
15705 reflections	$(\ /\ )_{max} = 0.001$
1113 parameters	$_{max} = 1.52$ e Å^{-3}
986 restraints	$_{min} = -0.59$ e Å^{-3}

Table S18. Fractional atomic coordinates and isotropic or equivalent isotropic displacement parameters (Å^2) for (**18**)

	x	y	z	U_{iso}*/U_{eq}	Occ. (<1)
C1	0.7350 (2)	0.41577 (18)	0.80574 (13)	0.0288 (5)	
O1	0.78799 (18)	0.45326 (16)	0.85959 (10)	0.0400 (5)	
C2	0.7716 (2)	0.36873 (19)	0.75268 (13)	0.0309 (6)	
C3	0.8727 (3)	0.3582 (2)	0.74720 (16)	0.0380 (7)	
H3	0.9365	0.3854	0.7813	0.046*	
C4	0.8781 (3)	0.3057 (2)	0.68928 (18)	0.0471 (8)	
H4	0.9475	0.2987	0.6843	0.056*	
C5	0.7861 (3)	0.2637 (2)	0.63932 (16)	0.0454 (8)	
H5	0.7934	0.2284	0.6011	0.054*	
C6	0.6813 (3)	0.2729 (2)	0.64446 (14)	0.0388 (7)	
C7	0.5780 (3)	0.2311 (2)	0.59964 (14)	0.0461 (8)	
H7	0.5743	0.1917	0.5603	0.055*	
C8	0.4841 (3)	0.2470 (2)	0.61249 (15)	0.0450 (8)	
H8	0.4164	0.2182	0.5813	0.054*	
C9	0.4826 (3)	0.3048 (2)	0.67029 (14)	0.0371 (6)	
H9	0.4158	0.3151	0.6776	0.045*	
C10	0.5806 (2)	0.34515 (19)	0.71521 (13)	0.0307 (6)	
C11	0.6780 (3)	0.32745 (19)	0.70186 (13)	0.0300 (6)	
C12	0.6132 (2)	0.40472 (18)	0.77973 (12)	0.0277 (5)	
N13	0.56180 (19)	0.44898 (15)	0.81308 (10)	0.0274 (5)	
N14	0.45440 (19)	0.43978 (16)	0.78347 (10)	0.0286 (5)	
C15	0.4186 (2)	0.50261 (17)	0.81586 (12)	0.0243 (5)	
N16	0.31878 (19)	0.51006 (16)	0.79377 (11)	0.0302 (5)	
H16	0.293 (3)	0.548 (2)	0.8160 (14)	0.036*	
C17	0.2485 (3)	0.4490 (2)	0.73265 (14)	0.0395 (7)	
H17A	0.2193	0.3840	0.7371	0.047*	
H17B	0.2933	0.4467	0.7012	0.047*	
C18	0.1553 (2)	0.4850 (2)	0.71038 (15)	0.0425 (7)	
H18	0.1065	0.4922	0.7368	0.051*	
C19	0.1363 (3)	0.5075 (3)	0.65602 (16)	0.0482 (8)	
H19A	0.1838	0.5010	0.6287	0.058*	
H19B	0.0751	0.5304	0.6441	0.058*	
S20	0.50908 (5)	0.57845 (4)	0.88757 (3)	0.02031 (13)	
C21	0.4282 (2)	0.65190 (16)	0.91330 (11)	0.0199 (5)	

C22	0.4966 (2)	0.75568 (16)	0.94255 (11)	0.0203 (5)	
C23	0.41470 (19)	0.72745 (16)	0.87911 (11)	0.0199 (5)	
H23	0.4497	0.7270	0.8427	0.024*	
C24	0.6167 (2)	0.78581 (16)	0.94344 (11)	0.0206 (5)	
C25	0.6629 (2)	0.82778 (17)	0.89187 (11)	0.0211 (5)	
C26	0.7018 (2)	0.74997 (17)	0.85221 (12)	0.0226 (5)	
C27	0.7898 (2)	0.74350 (18)	0.89196 (12)	0.0239 (5)	
C28	0.8045 (2)	0.81636 (18)	0.95407 (13)	0.0264 (5)	
C29	0.7060 (2)	0.77963 (17)	0.98260 (11)	0.0235 (5)	
C30	0.3344 (2)	0.60528 (17)	0.93978 (12)	0.0238 (5)	
O30	0.24008 (16)	0.59978 (15)	0.91816 (10)	0.0350 (4)	
O31	0.37026 (16)	0.56756 (13)	0.98543 (9)	0.0292 (4)	
C31	0.2860 (3)	0.5244 (2)	1.01506 (16)	0.0406 (7)	
H31A	0.2482	0.5702	1.0294	0.061*	
H31B	0.3208	0.5047	1.0512	0.061*	
H31C	0.2321	0.4692	0.9845	0.061*	
C32	0.4620 (2)	0.80492 (17)	0.99784 (11)	0.0246 (5)	
O32	0.39081 (18)	0.76753 (14)	1.02039 (9)	0.0365 (5)	
O33	0.51979 (17)	0.89576 (12)	1.01663 (8)	0.0296 (4)	
C33	0.4821 (3)	0.9518 (2)	1.06365 (14)	0.0401 (7)	
H33A	0.4065	0.9516	1.0457	0.060*	
H33B	0.5311	1.0167	1.0766	0.060*	
H33C	0.4834	0.9247	1.1004	0.060*	
C34	0.3112 (2)	0.75711 (18)	0.86988 (12)	0.0242 (5)	
O34	0.29467 (17)	0.82047 (14)	0.90467 (10)	0.0363 (5)	
O35	0.24324 (15)	0.70563 (14)	0.81477 (9)	0.0292 (4)	
C35	0.1378 (2)	0.7242 (3)	0.79928 (17)	0.0443 (8)	
H35A	0.0923	0.7010	0.8269	0.066*	
H35B	0.0998	0.6918	0.7553	0.066*	
H35C	0.1499	0.7922	0.8051	0.066*	
O36	0.60125 (14)	0.87677 (12)	0.86360 (8)	0.0213 (3)	
C36	0.6554 (2)	0.93036 (19)	0.82479 (13)	0.0271 (5)	
H36A	0.7277	0.9723	0.8500	0.041*	
H36B	0.6097	0.9681	0.8089	0.041*	
H36C	0.6653	0.8870	0.7892	0.041*	
C37	0.6445 (2)	0.68861 (17)	0.78851 (11)	0.0220 (5)	
O37	0.68125 (16)	0.63456 (14)	0.75833 (9)	0.0323 (4)	

O38	0.54501 (14)	0.69956 (12)	0.76898 (8)	0.0233 (4)	
C38	0.4810 (2)	0.6403 (2)	0.70711 (12)	0.0295 (6)	
H38A	0.5195	0.6572	0.6751	0.044*	
H38B	0.4078	0.6500	0.6979	0.044*	
H38C	0.4726	0.5739	0.7068	0.044*	
C39	0.8646 (2)	0.68294 (18)	0.88585 (13)	0.0266 (5)	
O39	0.90597 (16)	0.66924 (16)	0.84254 (10)	0.0378 (5)	
O40	0.88415 (16)	0.65289 (14)	0.93834 (9)	0.0327 (4)	
C40	0.9760 (3)	0.6131 (2)	0.94609 (17)	0.0436 (8)	
H40A	0.9608	0.5577	0.9113	0.065*	
H40B	0.9854	0.5945	0.9861	0.065*	
H40C	1.0438	0.6604	0.9461	0.065*	
C41	0.9217 (2)	0.86286 (19)	0.99677 (14)	0.0331 (6)	
O41	0.9406 (2)	0.91057 (19)	1.04836 (11)	0.0523 (6)	
O42	0.99568 (17)	0.84751 (16)	0.96534 (12)	0.0465 (6)	
C42	1.1117 (3)	0.8912 (3)	0.9970 (2)	0.0603 (11)	
H42A	1.1322	0.9582	0.9972	0.090*	
H42B	1.1568	0.8601	0.9748	0.090*	
H42C	1.1244	0.8847	1.0405	0.090*	
C43	0.7131 (2)	0.73571 (18)	1.03634 (12)	0.0276 (5)	
O43	0.79430 (19)	0.71795 (17)	1.06147 (10)	0.0425 (5)	
O44	0.61625 (17)	0.71654 (13)	1.05182 (9)	0.0310 (4)	
C44	0.6085 (3)	0.6681 (2)	1.10167 (14)	0.0400 (7)	
H44A	0.6255	0.6084	1.0902	0.060*	
H44B	0.5333	0.6554	1.1078	0.060*	
H44C	0.6614	0.7078	1.1408	0.060*	
O45	0.76964 (14)	0.88833 (12)	0.92931 (8)	0.0251 (4)	
C51	0.3022 (3)	-0.0510 (2)	0.3274 (2)	0.0466 (8)	
O51	0.3504 (2)	-0.0080 (2)	0.38382 (14)	0.0648 (7)	
C52	0.3512 (3)	-0.0953 (2)	0.27947 (19)	0.0476 (8)	
C53	0.4573 (4)	-0.0999 (3)	0.2826 (2)	0.0635 (12)	
H53	0.5159	-0.0689	0.3191	0.076*	
C54	0.4736 (4)	-0.1550 (3)	0.2266 (2)	0.0621 (11)	
H54	0.5455	-0.1608	0.2270	0.075*	
C55	0.3916 (4)	-0.1988 (3)	0.1736 (2)	0.0602 (11)	
H55	0.4076	-0.2335	0.1382	0.072*	
C56	0.2826 (3)	-0.1938 (3)	0.17038 (19)	0.0511 (9)	

C57	0.1950 (4)	-0.2325 (3)	0.1228 (2)	0.0660 (11)	
H57	0.2022	-0.2674	0.0844	0.079*	
C58	0.0915 (4)	-0.2231 (3)	0.1278 (2)	0.0667 (11)	
H58	0.0301	-0.2555	0.0929	0.080*	
C59	0.0715 (3)	-0.1677 (3)	0.18221 (16)	0.0505 (8)	
H59	0.0011	-0.1596	0.1835	0.061*	
C60	0.1611 (3)	-0.1286 (2)	0.23088 (17)	0.0424 (7)	
C61	0.2680 (3)	-0.1397 (2)	0.22584 (19)	0.0492 (8)	
C62	0.1810 (2)	-0.07139 (19)	0.29518 (15)	0.0340 (6)	
N63	0.1170 (2)	-0.03755 (16)	0.32509 (12)	0.0314 (5)	
N64	0.00954 (19)	-0.06235 (16)	0.28993 (11)	0.0299 (5)	
C65	-0.0454 (2)	-0.01118 (18)	0.31634 (13)	0.0278 (5)	
N66	-0.1510 (2)	-0.02492 (19)	0.28830 (13)	0.0430 (6)	
H66	-0.189 (3)	0.012 (2)	0.3016 (18)	0.052*	
C67	-0.2079 (4)	-0.0966 (3)	0.22943 (19)	0.0594 (10)*	
H67A	-0.1869	-0.1548	0.2322	0.071*	0.578 (6)
H67B	-0.2885	-0.1117	0.2256	0.071*	0.578 (6)
H67C	-0.2672	-0.1459	0.2371	0.071*	0.422 (6)
H67D	-0.1548	-0.1270	0.2141	0.071*	0.422 (6)
C68	-0.1861 (5)	-0.0718 (4)	0.1735 (3)	0.0486 (11)*	0.578 (6)
H68	-0.1167	-0.0722	0.1650	0.058*	0.578 (6)
C69	-0.2564 (9)	-0.0482 (8)	0.1330 (4)	0.082 (2)*	0.578 (6)
H69A	-0.3265	-0.0471	0.1402	0.098*	0.578 (6)
H69B	-0.2370	-0.0323	0.0965	0.098*	0.578 (6)
C68A	-0.2568 (7)	-0.0529 (6)	0.1805 (3)	0.0486 (11)*	0.422 (6)
H68A	-0.3220	-0.0380	0.1869	0.058*	0.422 (6)
C69A	-0.2275 (13)	-0.0309 (11)	0.1302 (5)	0.082 (2)*	0.422 (6)
H69C	-0.1634	-0.0430	0.1197	0.098*	0.422 (6)
H69D	-0.2706	-0.0030	0.1045	0.098*	0.422 (6)
S70	0.02115 (5)	0.07608 (4)	0.38875 (3)	0.02269 (13)	
C71	-0.08564 (19)	0.13064 (17)	0.39763 (11)	0.0202 (5)	
C72	-0.04507 (19)	0.23843 (16)	0.42191 (11)	0.0184 (4)	
C73	-0.10657 (19)	0.19321 (16)	0.35402 (11)	0.0199 (5)	
H73	-0.0586	0.1973	0.3236	0.024*	
C74	0.07537 (19)	0.28954 (16)	0.43617 (11)	0.0188 (4)	
C75	0.13447 (19)	0.33918 (16)	0.39116 (11)	0.0194 (5)	
C76	0.21167 (19)	0.28100 (17)	0.36975 (11)	0.0203 (5)	

C77	0.29016 (19)	0.29439 (17)	0.42174 (11)	0.0201 (5)	
C78	0.26199 (19)	0.36093 (17)	0.47391 (11)	0.0208 (5)	
C79	0.15561 (19)	0.30295 (16)	0.48717 (11)	0.0197 (5)	
C80	-0.1820 (2)	0.07331 (17)	0.41805 (12)	0.0239 (5)	
O80	-0.27492 (15)	0.04791 (16)	0.38606 (10)	0.0371 (5)	
O81	-0.14845 (15)	0.05064 (14)	0.47097 (9)	0.0319 (4)	
C81	-0.2354 (3)	-0.0054 (3)	0.49345 (17)	0.0453 (8)	
H81A	-0.2923	0.0267	0.4965	0.068*	
H81B	-0.2038	-0.0129	0.5351	0.068*	
H81C	-0.2685	-0.0676	0.4639	0.068*	
C82	-0.1087 (2)	0.28050 (17)	0.46453 (11)	0.0208 (5)	
O82	-0.18469 (15)	0.23600 (13)	0.48044 (9)	0.0292 (4)	
O83	-0.06786 (15)	0.37367 (12)	0.48022 (9)	0.0290 (4)	
C83	-0.1283 (3)	0.4236 (2)	0.51579 (16)	0.0393 (7)	
H83A	-0.2039	0.4102	0.4905	0.059*	
H83B	-0.0912	0.4916	0.5263	0.059*	
H83C	-0.1306	0.4027	0.5547	0.059*	
C84	-0.2200 (2)	0.20043 (17)	0.32908 (11)	0.0216 (5)	
O84	-0.25814 (15)	0.25988 (13)	0.35092 (9)	0.0290 (4)	
O85	-0.27031 (15)	0.13268 (13)	0.27584 (9)	0.0296 (4)	
C85	-0.3834 (2)	0.1275 (2)	0.24885 (14)	0.0361 (6)	
H85A	-0.4301	0.1053	0.2762	0.054*	
H85B	-0.4095	0.0835	0.2071	0.054*	
H85C	-0.3874	0.1902	0.2449	0.054*	
O86	0.06827 (14)	0.36857 (12)	0.34909 (8)	0.0222 (3)	
C86	0.1267 (2)	0.42414 (19)	0.31300 (12)	0.0269 (5)	
H86A	0.1801	0.4816	0.3415	0.040*	
H86B	0.0738	0.4413	0.2830	0.040*	
H86C	0.1660	0.3873	0.2900	0.040*	
C87	0.1926 (2)	0.21176 (17)	0.30939 (11)	0.0208 (5)	
O87	0.26036 (15)	0.17535 (13)	0.29526 (9)	0.0296 (4)	
O88	0.09078 (14)	0.19403 (12)	0.27316 (8)	0.0250 (4)	
C88	0.0648 (2)	0.12586 (19)	0.21316 (12)	0.0292 (6)	
H88A	0.1090	0.1525	0.1860	0.044*	
H88B	-0.0140	0.1107	0.1928	0.044*	
H88C	0.0817	0.0683	0.2201	0.044*	
C89	0.3714 (2)	0.24175 (17)	0.43572 (11)	0.0216 (5)	

O89	0.35787 (16)	0.18562 (13)	0.46673 (9)	0.0318 (4)	
O90	0.45757 (15)	0.26811 (16)	0.41217 (9)	0.0360 (5)	
C90	0.5424 (3)	0.2214 (4)	0.42519 (18)	0.0611 (11)	
H90A	0.5800	0.2426	0.4701	0.092*	
H90B	0.5962	0.2371	0.4002	0.092*	
H90C	0.5079	0.1529	0.4140	0.092*	
C91	0.3519 (2)	0.42540 (18)	0.53085 (12)	0.0238 (5)	
O91	0.33541 (17)	0.48578 (16)	0.56775 (10)	0.0424 (5)	
C92	0.5392 (2)	0.4695 (2)	0.58374 (13)	0.0320 (6)	
H92A	0.5477	0.5356	0.5830	0.048*	
H92B	0.6075	0.4545	0.5793	0.048*	
H92C	0.5240	0.4595	0.6238	0.048*	
O92	0.44769 (14)	0.40867 (12)	0.53188 (8)	0.0240 (4)	
C93	0.1563 (2)	0.26729 (17)	0.54444 (11)	0.0227 (5)	
O93	0.23872 (17)	0.27698 (17)	0.58340 (9)	0.0406 (5)	
O94	0.05418 (15)	0.22249 (13)	0.54620 (9)	0.0297 (4)	
C94	0.0466 (3)	0.1781 (2)	0.59768 (14)	0.0370 (7)	
H94A	0.0829	0.1283	0.5939	0.055*	
H94B	-0.0314	0.1507	0.5965	0.055*	
H94C	0.0830	0.2253	0.6377	0.055*	
O95	0.21713 (13)	0.41754 (11)	0.43749 (8)	0.0207 (3)	

Table S19. Atomic displacement parameters (Å^2) for (**18**)

	U^{11}	U^{22}	U^{33}	U^{12}	U^{13}	U^{23}
C1	0.0335 (14)	0.0251 (12)	0.0302 (13)	0.0108 (11)	0.0101 (11)	0.0077 (10)
O1	0.0376 (11)	0.0443 (12)	0.0332 (11)	0.0117 (9)	0.0063 (9)	0.0009 (9)
C2	0.0395 (15)	0.0269 (13)	0.0329 (14)	0.0128 (11)	0.0162 (12)	0.0116 (11)
C3	0.0387 (16)	0.0336 (15)	0.0492 (17)	0.0129 (12)	0.0230 (14)	0.0134 (13)
C4	0.055 (2)	0.0432 (17)	0.058 (2)	0.0213 (15)	0.0360 (16)	0.0173 (15)
C5	0.075 (2)	0.0370 (16)	0.0404 (17)	0.0279 (16)	0.0348 (16)	0.0125 (13)
C6	0.0633 (19)	0.0327 (15)	0.0310 (14)	0.0228 (14)	0.0202 (13)	0.0125 (12)
C7	0.078 (2)	0.0429 (17)	0.0231 (14)	0.0307 (16)	0.0106 (14)	0.0054 (12)
C8	0.063 (2)	0.0413 (17)	0.0285 (15)	0.0219 (15)	0.0000 (14)	0.0051 (13)
C9	0.0466 (17)	0.0349 (15)	0.0300 (14)	0.0186 (13)	0.0035 (12)	0.0052 (12)
C10	0.0429 (15)	0.0284 (13)	0.0260 (13)	0.0163 (11)	0.0103 (11)	0.0099 (10)
C11	0.0471 (16)	0.0266 (13)	0.0255 (12)	0.0187 (12)	0.0154 (11)	0.0117 (10)
C12	0.0379 (14)	0.0243 (12)	0.0262 (12)	0.0128 (11)	0.0127 (11)	0.0090 (10)

N13	0.0322 (12)	0.0263 (11)	0.0266 (11)	0.0118 (9)	0.0100 (9)	0.0068 (9)
N14	0.0349 (12)	0.0262 (11)	0.0247 (11)	0.0113 (9)	0.0072 (9)	0.0032 (9)
C15	0.0302 (13)	0.0212 (11)	0.0214 (11)	0.0071 (10)	0.0063 (10)	0.0056 (9)
N16	0.0307 (12)	0.0270 (11)	0.0283 (11)	0.0098 (9)	0.0011 (9)	0.0005 (9)
C17	0.0384 (16)	0.0350 (15)	0.0334 (15)	0.0072 (13)	-0.0046 (12)	-0.0015 (12)
C18	0.0267 (14)	0.0535 (19)	0.0396 (16)	0.0075 (13)	0.0045 (12)	0.0030 (14)
C19	0.0395 (18)	0.069 (2)	0.0389 (17)	0.0263 (17)	0.0049 (14)	0.0098 (16)
S20	0.0227 (3)	0.0193 (3)	0.0202 (3)	0.0085 (2)	0.0064 (2)	0.0038 (2)
C21	0.0230 (11)	0.0193 (11)	0.0192 (11)	0.0082 (9)	0.0071 (9)	0.0047 (9)
C22	0.0273 (12)	0.0174 (11)	0.0194 (11)	0.0082 (9)	0.0095 (9)	0.0062 (9)
C23	0.0206 (11)	0.0219 (11)	0.0199 (11)	0.0077 (9)	0.0075 (9)	0.0071 (9)
C24	0.0267 (12)	0.0159 (10)	0.0182 (11)	0.0059 (9)	0.0049 (9)	0.0026 (9)
C25	0.0198 (11)	0.0217 (11)	0.0207 (11)	0.0045 (9)	0.0033 (9)	0.0065 (9)
C26	0.0199 (11)	0.0237 (12)	0.0269 (12)	0.0069 (9)	0.0080 (9)	0.0097 (10)
C27	0.0192 (11)	0.0247 (12)	0.0284 (12)	0.0040 (9)	0.0063 (10)	0.0105 (10)
C28	0.0229 (12)	0.0244 (12)	0.0296 (13)	0.0067 (10)	-0.0004 (10)	0.0085 (10)
C29	0.0275 (12)	0.0203 (11)	0.0202 (11)	0.0061 (9)	0.0020 (9)	0.0042 (9)
C30	0.0280 (13)	0.0219 (12)	0.0259 (12)	0.0102 (10)	0.0126 (10)	0.0066 (10)
O30	0.0272 (10)	0.0456 (12)	0.0404 (11)	0.0124 (9)	0.0155 (8)	0.0206 (9)
O31	0.0334 (10)	0.0326 (10)	0.0306 (9)	0.0123 (8)	0.0162 (8)	0.0177 (8)
C31	0.0481 (18)	0.0464 (17)	0.0466 (17)	0.0208 (14)	0.0295 (15)	0.0303 (15)
C32	0.0370 (14)	0.0209 (11)	0.0197 (11)	0.0121 (10)	0.0103 (10)	0.0057 (9)
O32	0.0527 (13)	0.0302 (10)	0.0346 (10)	0.0132 (9)	0.0288 (10)	0.0077 (8)
O33	0.0471 (11)	0.0202 (8)	0.0230 (9)	0.0106 (8)	0.0133 (8)	0.0032 (7)
C33	0.072 (2)	0.0259 (14)	0.0281 (14)	0.0191 (14)	0.0226 (15)	0.0030 (11)
C34	0.0237 (12)	0.0268 (12)	0.0282 (12)	0.0102 (10)	0.0116 (10)	0.0126 (10)
O34	0.0383 (11)	0.0375 (11)	0.0414 (11)	0.0229 (9)	0.0152 (9)	0.0087 (9)
O35	0.0205 (9)	0.0389 (10)	0.0315 (10)	0.0111 (8)	0.0062 (7)	0.0135 (8)
C35	0.0223 (14)	0.067 (2)	0.0522 (19)	0.0186 (14)	0.0102 (13)	0.0277 (17)
O36	0.0224 (8)	0.0220 (8)	0.0218 (8)	0.0071 (7)	0.0062 (7)	0.0096 (7)
C36	0.0276 (13)	0.0294 (13)	0.0296 (13)	0.0090 (11)	0.0095 (11)	0.0164 (11)
C37	0.0234 (12)	0.0239 (12)	0.0236 (11)	0.0078 (9)	0.0109 (10)	0.0110 (9)
O37	0.0330 (10)	0.0360 (10)	0.0324 (10)	0.0175 (8)	0.0121 (8)	0.0053 (8)
O38	0.0224 (8)	0.0290 (9)	0.0185 (8)	0.0094 (7)	0.0050 (7)	0.0034 (7)
C38	0.0320 (14)	0.0354 (14)	0.0191 (12)	0.0116 (11)	0.0028 (10)	0.0031 (10)
C39	0.0194 (12)	0.0258 (12)	0.0333 (13)	0.0051 (10)	0.0053 (10)	0.0075 (10)
O39	0.0284 (10)	0.0519 (13)	0.0410 (11)	0.0186 (9)	0.0149 (9)	0.0147 (10)

O40	0.0330 (10)	0.0334 (10)	0.0358 (10)	0.0160 (8)	0.0054 (8)	0.0125 (8)
C40	0.0373 (16)	0.0411 (17)	0.0529 (19)	0.0219 (14)	-0.0017 (14)	0.0117 (15)
C41	0.0310 (14)	0.0265 (13)	0.0354 (14)	0.0063 (11)	-0.0050 (11)	0.0084 (11)
O41	0.0427 (13)	0.0625 (15)	0.0331 (12)	0.0049 (11)	-0.0076 (10)	-0.0015 (11)
O42	0.0210 (10)	0.0398 (12)	0.0643 (15)	0.0022 (9)	-0.0001 (10)	-0.0022 (11)
C42	0.0241 (16)	0.0429 (19)	0.091 (3)	0.0027 (14)	-0.0089 (17)	-0.0039 (19)
C43	0.0354 (14)	0.0231 (12)	0.0219 (12)	0.0076 (10)	0.0031 (10)	0.0053 (10)
O43	0.0436 (12)	0.0562 (14)	0.0375 (11)	0.0254 (11)	0.0060 (9)	0.0242 (10)
O44	0.0400 (11)	0.0316 (10)	0.0249 (9)	0.0116 (8)	0.0073 (8)	0.0146 (8)
C44	0.056 (2)	0.0410 (16)	0.0300 (14)	0.0153 (14)	0.0149 (14)	0.0202 (13)
O45	0.0227 (9)	0.0215 (8)	0.0269 (9)	0.0029 (7)	-0.0010 (7)	0.0077 (7)
C51	0.0428 (17)	0.0341 (16)	0.078 (2)	0.0194 (13)	0.0308 (16)	0.0233 (15)
O51	0.0500 (15)	0.0571 (16)	0.0707 (18)	0.0151 (13)	-0.0051 (13)	-0.0026 (14)
C52	0.0534 (19)	0.0417 (17)	0.075 (2)	0.0282 (15)	0.0417 (17)	0.0335 (16)
C53	0.073 (2)	0.058 (2)	0.115 (3)	0.0480 (19)	0.076 (2)	0.063 (2)
C54	0.067 (2)	0.077 (3)	0.093 (3)	0.052 (2)	0.062 (2)	0.060 (2)
C55	0.091 (3)	0.069 (2)	0.079 (2)	0.061 (2)	0.071 (2)	0.056 (2)
C56	0.074 (2)	0.054 (2)	0.060 (2)	0.0420 (18)	0.0418 (18)	0.0399 (17)
C57	0.105 (3)	0.070 (3)	0.051 (2)	0.056 (2)	0.038 (2)	0.0240 (19)
C58	0.081 (3)	0.075 (3)	0.048 (2)	0.044 (2)	0.0059 (19)	0.0064 (19)
C59	0.069 (2)	0.0468 (19)	0.0347 (16)	0.0160 (17)	0.0187 (16)	0.0030 (14)
C60	0.0566 (19)	0.0335 (15)	0.0538 (18)	0.0238 (14)	0.0319 (15)	0.0183 (13)
C61	0.071 (2)	0.0409 (17)	0.077 (2)	0.0402 (16)	0.0578 (18)	0.0429 (16)
C62	0.0371 (15)	0.0265 (13)	0.0488 (16)	0.0157 (11)	0.0236 (13)	0.0129 (12)
N63	0.0311 (12)	0.0279 (11)	0.0387 (13)	0.0115 (9)	0.0131 (10)	0.0086 (10)
N64	0.0307 (12)	0.0294 (11)	0.0312 (12)	0.0124 (9)	0.0095 (9)	0.0046 (9)
C65	0.0300 (13)	0.0218 (12)	0.0332 (13)	0.0081 (10)	0.0116 (11)	0.0062 (10)
N66	0.0294 (13)	0.0384 (14)	0.0497 (16)	0.0133 (11)	0.0008 (11)	-0.0124 (12)
S70	0.0196 (3)	0.0228 (3)	0.0278 (3)	0.0101 (2)	0.0062 (2)	0.0057 (2)
C71	0.0178 (11)	0.0213 (11)	0.0238 (11)	0.0089 (9)	0.0065 (9)	0.0051 (9)
C72	0.0169 (11)	0.0199 (11)	0.0199 (11)	0.0069 (9)	0.0054 (9)	0.0056 (9)
C73	0.0181 (11)	0.0219 (11)	0.0212 (11)	0.0075 (9)	0.0067 (9)	0.0051 (9)
C74	0.0192 (11)	0.0182 (11)	0.0220 (11)	0.0079 (9)	0.0091 (9)	0.0048 (9)
C75	0.0165 (10)	0.0216 (11)	0.0212 (11)	0.0066 (9)	0.0062 (9)	0.0054 (9)
C76	0.0175 (11)	0.0234 (11)	0.0225 (11)	0.0073 (9)	0.0080 (9)	0.0070 (9)
C77	0.0183 (11)	0.0214 (11)	0.0216 (11)	0.0058 (9)	0.0081 (9)	0.0045 (9)
C78	0.0176 (11)	0.0227 (11)	0.0224 (11)	0.0065 (9)	0.0066 (9)	0.0039 (9)

C79	0.0176 (11)	0.0200 (11)	0.0214 (11)	0.0062 (9)	0.0060 (9)	0.0024 (9)
C80	0.0221 (12)	0.0215 (11)	0.0307 (13)	0.0079 (9)	0.0103 (10)	0.0072 (10)
O80	0.0204 (9)	0.0505 (12)	0.0392 (11)	0.0044 (8)	0.0086 (8)	0.0155 (9)
O81	0.0263 (9)	0.0327 (10)	0.0397 (11)	0.0046 (8)	0.0097 (8)	0.0201 (8)
C81	0.0342 (16)	0.0527 (19)	0.0526 (19)	0.0027 (14)	0.0151 (14)	0.0310 (16)
C82	0.0204 (11)	0.0233 (11)	0.0209 (11)	0.0097 (9)	0.0058 (9)	0.0056 (9)
O82	0.0302 (10)	0.0307 (9)	0.0340 (10)	0.0112 (8)	0.0193 (8)	0.0112 (8)
O83	0.0262 (9)	0.0224 (8)	0.0401 (10)	0.0091 (7)	0.0156 (8)	0.0015 (8)
C83	0.0357 (15)	0.0291 (14)	0.0543 (19)	0.0135 (12)	0.0230 (14)	-0.0037 (13)
C84	0.0180 (11)	0.0265 (12)	0.0222 (11)	0.0061 (9)	0.0053 (9)	0.0107 (9)
O84	0.0244 (9)	0.0353 (10)	0.0318 (10)	0.0158 (8)	0.0080 (8)	0.0082 (8)
O85	0.0229 (9)	0.0331 (10)	0.0283 (9)	0.0087 (7)	-0.0009 (7)	0.0036 (8)
C85	0.0234 (13)	0.0477 (17)	0.0321 (14)	0.0076 (12)	-0.0025 (11)	0.0113 (13)
O86	0.0198 (8)	0.0274 (9)	0.0244 (8)	0.0097 (7)	0.0081 (7)	0.0121 (7)
C86	0.0271 (13)	0.0318 (13)	0.0271 (13)	0.0094 (11)	0.0108 (10)	0.0148 (11)
C87	0.0203 (11)	0.0233 (11)	0.0207 (11)	0.0061 (9)	0.0073 (9)	0.0082 (9)
O87	0.0267 (9)	0.0362 (10)	0.0270 (9)	0.0147 (8)	0.0082 (8)	0.0015 (8)
O88	0.0227 (9)	0.0300 (9)	0.0204 (8)	0.0082 (7)	0.0045 (7)	0.0021 (7)
C88	0.0310 (14)	0.0318 (14)	0.0200 (12)	0.0062 (11)	0.0036 (10)	0.0016 (10)
C89	0.0202 (11)	0.0253 (12)	0.0177 (11)	0.0076 (9)	0.0041 (9)	0.0011 (9)
O89	0.0353 (10)	0.0271 (9)	0.0333 (10)	0.0090 (8)	0.0065 (8)	0.0105 (8)
O90	0.0232 (9)	0.0666 (14)	0.0315 (10)	0.0240 (9)	0.0145 (8)	0.0217 (10)
C90	0.0441 (19)	0.122 (4)	0.051 (2)	0.058 (2)	0.0269 (17)	0.039 (2)
C91	0.0197 (11)	0.0263 (12)	0.0238 (12)	0.0041 (9)	0.0064 (9)	0.0046 (10)
O91	0.0286 (10)	0.0472 (12)	0.0382 (11)	0.0122 (9)	0.0021 (9)	-0.0161 (10)
C92	0.0220 (13)	0.0320 (14)	0.0322 (14)	0.0008 (11)	-0.0013 (11)	0.0020 (11)
O92	0.0172 (8)	0.0256 (9)	0.0238 (9)	0.0032 (7)	0.0011 (7)	0.0015 (7)
C93	0.0227 (12)	0.0261 (12)	0.0205 (11)	0.0095 (10)	0.0067 (9)	0.0039 (9)
O93	0.0276 (10)	0.0687 (15)	0.0287 (10)	0.0152 (10)	0.0051 (8)	0.0202 (10)
O94	0.0276 (9)	0.0352 (10)	0.0274 (9)	0.0051 (8)	0.0076 (8)	0.0148 (8)
C94	0.0443 (17)	0.0410 (16)	0.0322 (15)	0.0105 (13)	0.0168 (13)	0.0200 (13)
O95	0.0178 (8)	0.0201 (8)	0.0233 (8)	0.0050 (6)	0.0050 (7)	0.0046 (6)

Table S20. Geometric parameters (Å, º) for (**18**)

C1—O1	1.213 (3)	C52—C53	1.375 (5)
C1—C2	1.480 (4)	C53—C54	1.447 (6)
C1—C12	1.522 (4)	C53—H53	0.9500

C2—C3	1.376 (4)	C54—C55	1.356 (7)
C2—C11	1.405 (4)	C54—H54	0.9500
C3—C4	1.407 (5)	C55—C56	1.415 (5)
C3—H3	0.9500	C55—H55	0.9500
C4—C5	1.382 (5)	C56—C57	1.315 (6)
C4—H4	0.9500	C56—C61	1.421 (5)
C5—C6	1.415 (5)	C57—C58	1.399 (6)
C5—H5	0.9500	C57—H57	0.9500
C6—C11	1.401 (4)	C58—C59	1.444 (5)
C6—C7	1.419 (5)	C58—H58	0.9500
C7—C8	1.362 (5)	C59—C60	1.344 (5)
C7—H7	0.9500	C59—H59	0.9500
C8—C9	1.421 (4)	C60—C61	1.452 (5)
C8—H8	0.9500	C60—C62	1.468 (5)
C9—C10	1.372 (4)	C62—N63	1.295 (4)
C9—H9	0.9500	N63—N64	1.376 (3)
C10—C11	1.418 (4)	N64—C65	1.306 (3)
C10—C12	1.468 (4)	C65—N66	1.332 (4)
C12—N13	1.288 (3)	C65—S70	1.788 (3)
N13—N14	1.377 (3)	N66—C67	1.464 (5)
N14—C15	1.312 (3)	N66—H66	0.871 (19)
C15—N16	1.328 (4)	C67—C68	1.424 (6)
C15—S20	1.782 (3)	C67—C68A	1.498 (7)
N16—C17	1.475 (4)	C67—H67A	0.9900
N16—H16	0.857 (18)	C67—H67B	0.9900
C17—C18	1.479 (4)	C67—H67C	0.9900
C17—H17A	0.9900	C67—H67D	0.9900
C17—H17B	0.9900	C68—C69	1.319 (8)
C18—C19	1.315 (5)	C68—H68	0.9500
C18—H18	0.9500	C69—H69A	0.9500
C19—H19A	0.9500	C69—H69B	0.9500
C19—H19B	0.9500	C68A—C69A	1.322 (8)
S20—C21	1.792 (2)	C68A—H68A	0.9500
C21—C23	1.513 (3)	C69A—H69C	0.9500
C21—C30	1.518 (3)	C69A—H69D	0.9500
C21—C22	1.540 (3)	S70—C71	1.794 (2)
C22—C24	1.495 (3)	C71—C73	1.512 (3)

C22—C23	1.512 (3)	C71—C80	1.524 (3)
C22—C32	1.517 (3)	C71—C72	1.535 (3)
C23—C34	1.501 (3)	C72—C74	1.493 (3)
C23—H23	1.0000	C72—C73	1.509 (3)
C24—C29	1.343 (4)	C72—C82	1.518 (3)
C24—C25	1.556 (3)	C73—C84	1.501 (3)
C25—O36	1.362 (3)	C73—H73	1.0000
C25—O45	1.442 (3)	C74—C79	1.331 (3)
C25—C26	1.577 (3)	C74—C75	1.560 (3)
C26—C27	1.338 (4)	C75—O86	1.360 (3)
C26—C37	1.481 (4)	C75—O95	1.449 (3)
C27—C39	1.492 (4)	C75—C76	1.565 (3)
C27—C28	1.546 (4)	C76—C77	1.333 (3)
C28—O45	1.437 (3)	C76—C87	1.479 (3)
C28—C41	1.536 (4)	C77—C89	1.487 (3)
C28—C29	1.552 (4)	C77—C78	1.549 (3)
C29—C43	1.474 (3)	C78—O95	1.433 (3)
C30—O30	1.195 (3)	C78—C91	1.524 (3)
C30—O31	1.322 (3)	C78—C79	1.544 (3)
O31—C31	1.456 (3)	C79—C93	1.476 (3)
C31—H31A	0.9800	C80—O80	1.198 (3)
C31—H31B	0.9800	C80—O81	1.316 (3)
C31—H31C	0.9800	O81—C81	1.455 (3)
C32—O32	1.198 (3)	C81—H81A	0.9800
C32—O33	1.330 (3)	C81—H81B	0.9800
O33—C33	1.454 (3)	C81—H81C	0.9800
C33—H33A	0.9800	C82—O82	1.195 (3)
C33—H33B	0.9800	C82—O83	1.325 (3)
C33—H33C	0.9800	O83—C83	1.451 (3)
C34—O34	1.203 (3)	C83—H83A	0.9800
C34—O35	1.334 (3)	C83—H83B	0.9800
O35—C35	1.447 (3)	C83—H83C	0.9800
C35—H35A	0.9800	C84—O84	1.197 (3)
C35—H35B	0.9800	C84—O85	1.342 (3)
C35—H35C	0.9800	O85—C85	1.445 (3)
O36—C36	1.449 (3)	C85—H85A	0.9800
C36—H36A	0.9800	C85—H85B	0.9800

C36—H36B	0.9800	C85—H85C	0.9800
C36—H36C	0.9800	O86—C86	1.444 (3)
C37—O37	1.205 (3)	C86—H86A	0.9800
C37—O38	1.335 (3)	C86—H86B	0.9800
O38—C38	1.458 (3)	C86—H86C	0.9800
C38—H38A	0.9800	C87—O87	1.201 (3)
C38—H38B	0.9800	C87—O88	1.338 (3)
C38—H38C	0.9800	O88—C88	1.451 (3)
C39—O39	1.197 (3)	C88—H88A	0.9800
C39—O40	1.336 (3)	C88—H88B	0.9800
O40—C40	1.452 (3)	C88—H88C	0.9800
C40—H40A	0.9800	C89—O89	1.191 (3)
C40—H40B	0.9800	C89—O90	1.320 (3)
C40—H40C	0.9800	O90—C90	1.456 (4)
C41—O41	1.183 (4)	C90—H90A	0.9800
C41—O42	1.325 (4)	C90—H90B	0.9800
O42—C42	1.451 (4)	C90—H90C	0.9800
C42—H42A	0.9800	C91—O91	1.198 (3)
C42—H42B	0.9800	C91—O92	1.317 (3)
C42—H42C	0.9800	C92—O92	1.456 (3)
C43—O43	1.207 (3)	C92—H92A	0.9800
C43—O44	1.334 (3)	C92—H92B	0.9800
O44—C44	1.446 (3)	C92—H92C	0.9800
C44—H44A	0.9800	C93—O93	1.196 (3)
C44—H44B	0.9800	C93—O94	1.333 (3)
C44—H44C	0.9800	O94—C94	1.440 (3)
C51—O51	1.261 (5)	C94—H94A	0.9800
C51—C52	1.478 (5)	C94—H94B	0.9800
C51—C62	1.518 (5)	C94—H94C	0.9800
C52—C61	1.374 (6)		
O1—C1—C2	128.4 (3)	C52—C53—C54	115.6 (5)
O1—C1—C12	125.9 (2)	C52—C53—H53	122.2
C2—C1—C12	105.6 (2)	C54—C53—H53	122.2
C3—C2—C11	120.4 (3)	C55—C54—C53	123.3 (4)
C3—C2—C1	132.3 (3)	C55—C54—H54	118.3
C11—C2—C1	107.3 (2)	C53—C54—H54	118.3

C2—C3—C4	117.6 (3)	C54—C55—C56	121.0 (4)
C2—C3—H3	121.2	C54—C55—H55	119.5
C4—C3—H3	121.2	C56—C55—H55	119.5
C5—C4—C3	122.5 (3)	C57—C56—C55	128.0 (4)
C5—C4—H4	118.8	C57—C56—C61	117.2 (4)
C3—C4—H4	118.8	C55—C56—C61	114.8 (4)
C4—C5—C6	120.6 (3)	C56—C57—C58	121.0 (4)
C4—C5—H5	119.7	C56—C57—H57	119.5
C6—C5—H5	119.7	C58—C57—H57	119.5
C11—C6—C5	116.2 (3)	C57—C58—C59	124.6 (4)
C11—C6—C7	115.5 (3)	C57—C58—H58	117.7
C5—C6—C7	128.3 (3)	C59—C58—H58	117.7
C8—C7—C6	120.7 (3)	C60—C59—C58	114.2 (4)
C8—C7—H7	119.7	C60—C59—H59	122.9
C6—C7—H7	119.7	C58—C59—H59	122.9
C7—C8—C9	123.1 (3)	C59—C60—C61	121.0 (3)
C7—C8—H8	118.5	C59—C60—C62	134.3 (3)
C9—C8—H8	118.5	C61—C60—C62	104.7 (3)
C10—C9—C8	118.0 (3)	C52—C61—C56	124.1 (3)
C10—C9—H9	121.0	C52—C61—C60	113.9 (3)
C8—C9—H9	121.0	C56—C61—C60	122.0 (4)
C9—C10—C11	118.7 (3)	N63—C62—C60	132.2 (3)
C9—C10—C12	134.8 (3)	N63—C62—C51	119.9 (3)
C11—C10—C12	106.4 (2)	C60—C62—C51	107.9 (3)
C6—C11—C2	122.7 (3)	C62—N63—N64	113.2 (2)
C6—C11—C10	124.0 (3)	C65—N64—N63	110.6 (2)
C2—C11—C10	113.3 (2)	N64—C65—N66	118.9 (3)
N13—C12—C10	132.9 (3)	N64—C65—S70	120.1 (2)
N13—C12—C1	119.8 (2)	N66—C65—S70	121.0 (2)
C10—C12—C1	107.2 (2)	C65—N66—C67	122.1 (3)
C12—N13—N14	115.2 (2)	C65—N66—H66	122 (3)
C15—N14—N13	109.7 (2)	C67—N66—H66	115 (3)
N14—C15—N16	119.8 (2)	C68—C67—N66	115.7 (4)
N14—C15—S20	118.1 (2)	N66—C67—C68A	110.4 (4)
N16—C15—S20	122.07 (19)	C68—C67—H67A	108.3
C15—N16—C17	120.4 (2)	N66—C67—H67A	108.3
C15—N16—H16	120 (2)	C68—C67—H67B	108.3

C17—N16—H16	119 (2)	N66—C67—H67B	108.3
N16—C17—C18	110.7 (2)	H67A—C67—H67B	107.4
N16—C17—H17A	109.5	N66—C67—H67C	109.6
C18—C17—H17A	109.5	C68A—C67—H67C	109.6
N16—C17—H17B	109.5	N66—C67—H67D	109.6
C18—C17—H17B	109.5	C68A—C67—H67D	109.6
H17A—C17—H17B	108.1	H67C—C67—H67D	108.1
C19—C18—C17	123.5 (3)	C69—C68—C67	124.0 (7)
C19—C18—H18	118.2	C69—C68—H68	118.0
C17—C18—H18	118.2	C67—C68—H68	118.0
C18—C19—H19A	120.0	C68—C69—H69A	120.0
C18—C19—H19B	120.0	C68—C69—H69B	120.0
H19A—C19—H19B	120.0	H69A—C69—H69B	120.0
C15—S20—C21	102.30 (12)	C69A—C68A—C67	132.8 (9)
C23—C21—C30	118.9 (2)	C69A—C68A—H68A	113.6
C23—C21—C22	59.37 (15)	C67—C68A—H68A	113.6
C30—C21—C22	120.7 (2)	C68A—C69A—H69C	120.0
C23—C21—S20	117.67 (16)	C68A—C69A—H69D	120.0
C30—C21—S20	115.45 (17)	H69C—C69A—H69D	120.0
C22—C21—S20	113.17 (17)	C65—S70—C71	100.60 (12)
C24—C22—C23	117.18 (19)	C73—C71—C80	119.5 (2)
C24—C22—C32	117.0 (2)	C73—C71—C72	59.39 (15)
C23—C22—C32	116.4 (2)	C80—C71—C72	119.83 (19)
C24—C22—C21	119.42 (19)	C73—C71—S70	116.51 (17)
C23—C22—C21	59.43 (15)	C80—C71—S70	115.33 (17)
C32—C22—C21	114.9 (2)	C72—C71—S70	114.86 (16)
C34—C23—C22	121.7 (2)	C74—C72—C73	118.40 (19)
C34—C23—C21	122.2 (2)	C74—C72—C82	115.4 (2)
C22—C23—C21	61.20 (15)	C73—C72—C82	116.64 (19)
C34—C23—H23	114.0	C74—C72—C71	119.95 (19)
C22—C23—H23	114.0	C73—C72—C71	59.55 (15)
C21—C23—H23	114.0	C82—C72—C71	115.59 (19)
C29—C24—C22	131.5 (2)	C84—C73—C72	120.8 (2)
C29—C24—C25	104.4 (2)	C84—C73—C71	122.3 (2)
C22—C24—C25	124.0 (2)	C72—C73—C71	61.06 (15)
O36—C25—O45	111.97 (19)	C84—C73—H73	114.2
O36—C25—C24	114.3 (2)	C72—C73—H73	114.2

O45—C25—C24	99.92 (18)	C71—C73—H73	114.2
O36—C25—C26	121.6 (2)	C79—C74—C72	129.9 (2)
O45—C25—C26	98.79 (18)	C79—C74—C75	104.6 (2)
C24—C25—C26	107.20 (19)	C72—C74—C75	125.5 (2)
C27—C26—C37	127.6 (2)	O86—C75—O95	112.14 (19)
C27—C26—C25	104.5 (2)	O86—C75—C74	114.77 (19)
C37—C26—C25	127.4 (2)	O95—C75—C74	99.35 (17)
C26—C27—C39	133.0 (2)	O86—C75—C76	121.3 (2)
C26—C27—C28	105.2 (2)	O95—C75—C76	99.02 (17)
C39—C27—C28	121.8 (2)	C74—C75—C76	107.09 (18)
O45—C28—C41	107.3 (2)	C77—C76—C87	125.9 (2)
O45—C28—C27	100.03 (19)	C77—C76—C75	104.7 (2)
C41—C28—C27	118.2 (2)	C87—C76—C75	128.4 (2)
O45—C28—C29	99.45 (19)	C76—C77—C89	130.2 (2)
C41—C28—C29	120.4 (2)	C76—C77—C78	105.3 (2)
C27—C28—C29	107.6 (2)	C89—C77—C78	122.7 (2)
C24—C29—C43	129.6 (2)	O95—C78—C91	109.13 (19)
C24—C29—C28	105.3 (2)	O95—C78—C79	100.11 (18)
C43—C29—C28	124.5 (2)	C91—C78—C79	116.8 (2)
O30—C30—O31	126.0 (2)	O95—C78—C77	99.96 (18)
O30—C30—C21	122.5 (2)	C91—C78—C77	121.0 (2)
O31—C30—C21	111.3 (2)	C79—C78—C77	106.58 (19)
C30—O31—C31	114.8 (2)	C74—C79—C93	131.7 (2)
O31—C31—H31A	109.5	C74—C79—C78	105.6 (2)
O31—C31—H31B	109.5	C93—C79—C78	122.5 (2)
H31A—C31—H31B	109.5	O80—C80—O81	126.0 (2)
O31—C31—H31C	109.5	O80—C80—C71	122.0 (2)
H31A—C31—H31C	109.5	O81—C80—C71	111.8 (2)
H31B—C31—H31C	109.5	C80—O81—C81	115.3 (2)
O32—C32—O33	124.9 (2)	O81—C81—H81A	109.5
O32—C32—C22	124.6 (2)	O81—C81—H81B	109.5
O33—C32—C22	110.5 (2)	H81A—C81—H81B	109.5
C32—O33—C33	114.5 (2)	O81—C81—H81C	109.5
O33—C33—H33A	109.5	H81A—C81—H81C	109.5
O33—C33—H33B	109.5	H81B—C81—H81C	109.5
H33A—C33—H33B	109.5	O82—C82—O83	125.7 (2)
O33—C33—H33C	109.5	O82—C82—C72	124.9 (2)

H33A—C33—H33C	109.5	O83—C82—C72	109.4 (2)
H33B—C33—H33C	109.5	C82—O83—C83	115.2 (2)
O34—C34—O35	125.5 (2)	O83—C83—H83A	109.5
O34—C34—C23	125.7 (2)	O83—C83—H83B	109.5
O35—C34—C23	108.7 (2)	H83A—C83—H83B	109.5
C34—O35—C35	115.7 (2)	O83—C83—H83C	109.5
O35—C35—H35A	109.5	H83A—C83—H83C	109.5
O35—C35—H35B	109.5	H83B—C83—H83C	109.5
H35A—C35—H35B	109.5	O84—C84—O85	124.8 (2)
O35—C35—H35C	109.5	O84—C84—C73	125.9 (2)
H35A—C35—H35C	109.5	O85—C84—C73	109.2 (2)
H35B—C35—H35C	109.5	C84—O85—C85	115.1 (2)
C25—O36—C36	113.65 (19)	O85—C85—H85A	109.5
O36—C36—H36A	109.5	O85—C85—H85B	109.5
O36—C36—H36B	109.5	H85A—C85—H85B	109.5
H36A—C36—H36B	109.5	O85—C85—H85C	109.5
O36—C36—H36C	109.5	H85A—C85—H85C	109.5
H36A—C36—H36C	109.5	H85B—C85—H85C	109.5
H36B—C36—H36C	109.5	C75—O86—C86	114.00 (18)
O37—C37—O38	124.0 (2)	O86—C86—H86A	109.5
O37—C37—C26	124.9 (2)	O86—C86—H86B	109.5
O38—C37—C26	111.1 (2)	H86A—C86—H86B	109.5
C37—O38—C38	115.16 (19)	O86—C86—H86C	109.5
O38—C38—H38A	109.5	H86A—C86—H86C	109.5
O38—C38—H38B	109.5	H86B—C86—H86C	109.5
H38A—C38—H38B	109.5	O87—C87—O88	124.5 (2)
O38—C38—H38C	109.5	O87—C87—C76	123.7 (2)
H38A—C38—H38C	109.5	O88—C87—C76	111.8 (2)
H38B—C38—H38C	109.5	C87—O88—C88	115.38 (19)
O39—C39—O40	125.9 (2)	O88—C88—H88A	109.5
O39—C39—C27	125.0 (2)	O88—C88—H88B	109.5
O40—C39—C27	109.0 (2)	H88A—C88—H88B	109.5
C39—O40—C40	115.1 (2)	O88—C88—H88C	109.5
O40—C40—H40A	109.5	H88A—C88—H88C	109.5
O40—C40—H40B	109.5	H88B—C88—H88C	109.5
H40A—C40—H40B	109.5	O89—C89—O90	126.7 (2)
O40—C40—H40C	109.5	O89—C89—C77	121.3 (2)

H40A—C40—H40C	109.5	O90—C89—C77	112.0 (2)
H40B—C40—H40C	109.5	C89—O90—C90	115.3 (2)
O41—C41—O42	126.4 (3)	O90—C90—H90A	109.5
O41—C41—C28	123.5 (3)	O90—C90—H90B	109.5
O42—C41—C28	109.8 (2)	H90A—C90—H90B	109.5
C41—O42—C42	117.7 (3)	O90—C90—H90C	109.5
O42—C42—H42A	109.5	H90A—C90—H90C	109.5
O42—C42—H42B	109.5	H90B—C90—H90C	109.5
H42A—C42—H42B	109.5	O91—C91—O92	125.8 (2)
O42—C42—H42C	109.5	O91—C91—C78	122.4 (2)
H42A—C42—H42C	109.5	O92—C91—C78	111.7 (2)
H42B—C42—H42C	109.5	O92—C92—H92A	109.5
O43—C43—O44	125.3 (2)	O92—C92—H92B	109.5
O43—C43—C29	124.6 (3)	H92A—C92—H92B	109.5
O44—C43—C29	110.1 (2)	O92—C92—H92C	109.5
C43—O44—C44	116.5 (2)	H92A—C92—H92C	109.5
O44—C44—H44A	109.5	H92B—C92—H92C	109.5
O44—C44—H44B	109.5	C91—O92—C92	115.2 (2)
H44A—C44—H44B	109.5	O93—C93—O94	125.2 (2)
O44—C44—H44C	109.5	O93—C93—C79	123.7 (2)
H44A—C44—H44C	109.5	O94—C93—C79	111.1 (2)
H44B—C44—H44C	109.5	C93—O94—C94	115.5 (2)
C28—O45—C25	96.05 (17)	O94—C94—H94A	109.5
O51—C51—C52	127.1 (3)	O94—C94—H94B	109.5
O51—C51—C62	127.2 (3)	H94A—C94—H94B	109.5
C52—C51—C62	105.7 (3)	O94—C94—H94C	109.5
C61—C52—C53	121.2 (4)	H94A—C94—H94C	109.5
C61—C52—C51	107.7 (3)	H94B—C94—H94C	109.5
C53—C52—C51	131.1 (4)	C78—O95—C75	95.80 (16)
O1—C1—C2—C3	-4.1 (5)	C62—C51—C52—C61	-1.9 (3)
C12—C1—C2—C3	177.9 (3)	O51—C51—C52—C53	-1.5 (6)
O1—C1—C2—C11	173.7 (3)	C62—C51—C52—C53	-179.3 (3)
C12—C1—C2—C11	-4.3 (3)	C61—C52—C53—C54	-0.8 (4)
C11—C2—C3—C4	0.4 (4)	C51—C52—C53—C54	176.3 (3)
C1—C2—C3—C4	178.0 (3)	C52—C53—C54—C55	0.9 (5)
C2—C3—C4—C5	-1.3 (5)	C53—C54—C55—C56	-0.7 (5)

C3—C4—C5—C6	0.5 (5)	C54—C55—C56—C57	-179.7 (4)
C4—C5—C6—C11	1.1 (4)	C54—C55—C56—C61	0.5 (5)
C4—C5—C6—C7	-176.5 (3)	C55—C56—C57—C58	178.0 (4)
C11—C6—C7—C8	2.0 (4)	C61—C56—C57—C58	-2.2 (6)
C5—C6—C7—C8	179.5 (3)	C56—C57—C58—C59	3.6 (7)
C6—C7—C8—C9	-0.3 (5)	C57—C58—C59—C60	-3.7 (6)
C7—C8—C9—C10	-0.8 (5)	C58—C59—C60—C61	2.6 (5)
C8—C9—C10—C11	0.0 (4)	C58—C59—C60—C62	-177.1 (4)
C8—C9—C10—C12	-177.6 (3)	C53—C52—C61—C56	0.7 (5)
C5—C6—C11—C2	-2.0 (4)	C51—C52—C61—C56	-177.1 (3)
C7—C6—C11—C2	175.9 (3)	C53—C52—C61—C60	179.3 (3)
C5—C6—C11—C10	179.3 (3)	C51—C52—C61—C60	1.5 (4)
C7—C6—C11—C10	-2.8 (4)	C57—C56—C61—C52	179.7 (3)
C3—C2—C11—C6	1.3 (4)	C55—C56—C61—C52	-0.4 (4)
C1—C2—C11—C6	-176.8 (2)	C57—C56—C61—C60	1.2 (5)
C3—C2—C11—C10	-179.9 (3)	C55—C56—C61—C60	-178.9 (3)
C1—C2—C11—C10	2.0 (3)	C59—C60—C61—C52	179.8 (3)
C9—C10—C11—C6	1.9 (4)	C62—C60—C61—C52	-0.4 (3)
C12—C10—C11—C6	-179.9 (3)	C59—C60—C61—C56	-1.6 (5)
C9—C10—C11—C2	-176.9 (3)	C62—C60—C61—C56	178.2 (3)
C12—C10—C11—C2	1.3 (3)	C59—C60—C62—N63	-2.5 (6)
C9—C10—C12—N13	-10.3 (5)	C61—C60—C62—N63	177.8 (3)
C11—C10—C12—N13	171.9 (3)	C59—C60—C62—C51	178.9 (4)
C9—C10—C12—C1	173.9 (3)	C61—C60—C62—C51	-0.8 (3)
C11—C10—C12—C1	-3.9 (3)	O51—C51—C62—N63	5.1 (5)
O1—C1—C12—N13	10.6 (4)	C52—C51—C62—N63	-177.1 (3)
C2—C1—C12—N13	-171.4 (2)	O51—C51—C62—C60	-176.2 (3)
O1—C1—C12—C10	-173.0 (3)	C52—C51—C62—C60	1.6 (3)
C2—C1—C12—C10	5.0 (3)	C60—C62—N63—N64	1.8 (4)
C10—C12—N13—N14	0.0 (4)	C51—C62—N63—N64	-179.8 (2)
C1—C12—N13—N14	175.4 (2)	C62—N63—N64—C65	-166.5 (2)
C12—N13—N14—C15	-166.5 (2)	N63—N64—C65—N66	179.3 (3)
N13—N14—C15—N16	176.0 (2)	N63—N64—C65—S70	-0.4 (3)
N13—N14—C15—S20	-1.9 (3)	N64—C65—N66—C67	1.1 (5)
N14—C15—N16—C17	-0.8 (4)	S70—C65—N66—C67	-179.2 (3)
S20—C15—N16—C17	177.0 (2)	C65—N66—C67—C68	-79.4 (5)
C15—N16—C17—C18	-165.8 (3)	C65—N66—C67—C68A	-124.9 (5)

N16—C17—C18—C19	122.4 (4)	N66—C67—C68—C69	-105.0 (8)
N14—C15—S20—C21	178.6 (2)	N66—C67—C68A—C69A	104.5 (13)
N16—C15—S20—C21	0.8 (2)	N64—C65—S70—C71	174.2 (2)
C15—S20—C21—C23	-74.5 (2)	N66—C65—S70—C71	-5.5 (3)
C15—S20—C21—C30	74.2 (2)	C65—S70—C71—C73	-72.9 (2)
C15—S20—C21—C22	-140.84 (17)	C65—S70—C71—C80	74.7 (2)
C23—C21—C22—C24	-106.0 (2)	C65—S70—C71—C72	-139.58 (18)
C30—C21—C22—C24	146.4 (2)	C73—C71—C72—C74	-107.3 (2)
S20—C21—C22—C24	3.5 (3)	C80—C71—C72—C74	144.0 (2)
C30—C21—C22—C23	-107.6 (2)	S70—C71—C72—C74	0.0 (3)
S20—C21—C22—C23	109.49 (18)	C80—C71—C72—C73	-108.7 (2)
C23—C21—C22—C32	107.2 (2)	S70—C71—C72—C73	107.31 (19)
C30—C21—C22—C32	-0.4 (3)	C73—C71—C72—C82	107.2 (2)
S20—C21—C22—C32	-143.32 (18)	C80—C71—C72—C82	-1.5 (3)
C24—C22—C23—C34	-138.2 (2)	S70—C71—C72—C82	-145.52 (17)
C32—C22—C23—C34	7.3 (3)	C74—C72—C73—C84	-137.7 (2)
C21—C22—C23—C34	112.0 (2)	C82—C72—C73—C84	7.0 (3)
C24—C22—C23—C21	109.8 (2)	C71—C72—C73—C84	112.4 (2)
C32—C22—C23—C21	-104.7 (2)	C74—C72—C73—C71	109.9 (2)
C30—C21—C23—C34	-0.7 (3)	C82—C72—C73—C71	-105.4 (2)
C22—C21—C23—C34	-111.2 (3)	C80—C71—C73—C84	-0.7 (3)
S20—C21—C23—C34	146.9 (2)	C72—C71—C73—C84	-110.0 (2)
C30—C21—C23—C22	110.5 (2)	S70—C71—C73—C84	145.49 (19)
S20—C21—C23—C22	-101.9 (2)	C80—C71—C73—C72	109.2 (2)
C23—C22—C24—C29	-150.2 (3)	S70—C71—C73—C72	-104.52 (19)
C32—C22—C24—C29	64.4 (3)	C73—C72—C74—C79	-150.9 (2)
C21—C22—C24—C29	-81.7 (3)	C82—C72—C74—C79	64.0 (3)
C23—C22—C24—C25	25.3 (3)	C71—C72—C74—C79	-81.6 (3)
C32—C22—C24—C25	-120.1 (2)	C73—C72—C74—C75	28.4 (3)
C21—C22—C24—C25	93.8 (3)	C82—C72—C74—C75	-116.8 (2)
C29—C24—C25—O36	-153.3 (2)	C71—C72—C74—C75	97.7 (3)
C22—C24—C25—O36	30.1 (3)	C79—C74—C75—O86	-154.4 (2)
C29—C24—C25—O45	-33.6 (2)	C72—C74—C75—O86	26.2 (3)
C22—C24—C25—O45	149.8 (2)	C79—C74—C75—O95	-34.6 (2)
C29—C24—C25—C26	68.9 (2)	C72—C74—C75—O95	146.0 (2)
C22—C24—C25—C26	-107.7 (2)	C79—C74—C75—C76	67.9 (2)
O36—C25—C26—C27	157.5 (2)	C72—C74—C75—C76	-111.5 (2)

O45—C25—C26—C27	34.8 (2)	O86—C75—C76—C77	157.8 (2)
C24—C25—C26—C27	-68.5 (2)	O95—C75—C76—C77	34.9 (2)
O36—C25—C26—C37	-30.3 (4)	C74—C75—C76—C77	-67.9 (2)
O45—C25—C26—C37	-152.9 (2)	O86—C75—C76—C87	-32.9 (4)
C24—C25—C26—C37	103.8 (3)	O95—C75—C76—C87	-155.8 (2)
C37—C26—C27—C39	6.3 (5)	C74—C75—C76—C87	101.5 (3)
C25—C26—C27—C39	178.5 (3)	C87—C76—C77—C89	-5.4 (4)
C37—C26—C27—C28	-172.7 (2)	C75—C76—C77—C89	164.2 (2)
C25—C26—C27—C28	-0.5 (2)	C87—C76—C77—C78	-170.4 (2)
C26—C27—C28—O45	-34.2 (2)	C75—C76—C77—C78	-0.7 (2)
C39—C27—C28—O45	146.6 (2)	C76—C77—C78—O95	-34.2 (2)
C26—C27—C28—C41	-150.2 (2)	C89—C77—C78—O95	159.4 (2)
C39—C27—C28—C41	30.6 (3)	C76—C77—C78—C91	-153.7 (2)
C26—C27—C28—C29	69.1 (2)	C89—C77—C78—C91	39.9 (3)
C39—C27—C28—C29	-110.0 (2)	C76—C77—C78—C79	69.6 (2)
C22—C24—C29—C43	3.6 (4)	C89—C77—C78—C79	-96.8 (3)
C25—C24—C29—C43	-172.6 (2)	C72—C74—C79—C93	5.1 (4)
C22—C24—C29—C28	175.4 (2)	C75—C74—C79—C93	-174.2 (2)
C25—C24—C29—C28	-0.8 (2)	C72—C74—C79—C78	180.0 (2)
O45—C28—C29—C24	35.0 (2)	C75—C74—C79—C78	0.6 (2)
C41—C28—C29—C24	151.6 (2)	O95—C78—C79—C74	34.0 (2)
C27—C28—C29—C24	-68.7 (2)	C91—C78—C79—C74	151.6 (2)
O45—C28—C29—C43	-152.6 (2)	C77—C78—C79—C74	-69.7 (2)
C41—C28—C29—C43	-36.0 (4)	O95—C78—C79—C93	-150.5 (2)
C27—C28—C29—C43	103.6 (3)	C91—C78—C79—C93	-33.0 (3)
C23—C21—C30—O30	26.3 (4)	C77—C78—C79—C93	105.8 (2)
C22—C21—C30—O30	95.9 (3)	C73—C71—C80—O80	27.5 (4)
S20—C21—C30—O30	-122.0 (2)	C72—C71—C80—O80	97.1 (3)
C23—C21—C30—O31	-157.9 (2)	S70—C71—C80—O80	-119.1 (2)
C22—C21—C30—O31	-88.4 (3)	C73—C71—C80—O81	-157.3 (2)
S20—C21—C30—O31	53.8 (3)	C72—C71—C80—O81	-87.8 (3)
O30—C30—O31—C31	-6.6 (4)	S70—C71—C80—O81	56.1 (3)
C21—C30—O31—C31	177.8 (2)	O80—C80—O81—C81	-4.3 (4)
C24—C22—C32—O32	-144.3 (3)	C71—C80—O81—C81	-179.2 (2)
C23—C22—C32—O32	70.1 (3)	C74—C72—C82—O82	-145.9 (2)
C21—C22—C32—O32	3.3 (4)	C73—C72—C82—O82	68.3 (3)
C24—C22—C32—O33	37.3 (3)	C71—C72—C82—O82	1.1 (3)

C23—C22—C32—O33	-108.4 (2)	C74—C72—C82—O83	35.1 (3)
C21—C22—C32—O33	-175.1 (2)	C73—C72—C82—O83	-110.7 (2)
O32—C32—O33—C33	-6.5 (4)	C71—C72—C82—O83	-177.8 (2)
C22—C32—O33—C33	171.9 (2)	O82—C82—O83—C83	-4.9 (4)
C22—C23—C34—O34	18.1 (4)	C72—C82—O83—C83	174.0 (2)
C21—C23—C34—O34	91.8 (3)	C72—C73—C84—O84	22.8 (4)
C22—C23—C34—O35	-165.8 (2)	C71—C73—C84—O84	96.0 (3)
C21—C23—C34—O35	-92.1 (3)	C72—C73—C84—O85	-160.8 (2)
O34—C34—O35—C35	-5.2 (4)	C71—C73—C84—O85	-87.7 (3)
C23—C34—O35—C35	178.7 (2)	O84—C84—O85—C85	-7.5 (4)
O45—C25—O36—C36	56.1 (3)	C73—C84—O85—C85	176.1 (2)
C24—C25—O36—C36	168.8 (2)	O95—C75—O86—C86	60.6 (2)
C26—C25—O36—C36	-60.1 (3)	C74—C75—O86—C86	172.97 (19)
C27—C26—C37—O37	-14.8 (4)	C76—C75—O86—C86	-55.8 (3)
C25—C26—C37—O37	174.7 (2)	C77—C76—C87—O87	-18.4 (4)
C27—C26—C37—O38	164.0 (2)	C75—C76—C87—O87	174.4 (2)
C25—C26—C37—O38	-6.5 (3)	C77—C76—C87—O88	160.8 (2)
O37—C37—O38—C38	0.2 (3)	C75—C76—C87—O88	-6.4 (3)
C26—C37—O38—C38	-178.7 (2)	O87—C87—O88—C88	-0.2 (3)
C26—C27—C39—O39	47.2 (4)	C76—C87—O88—C88	-179.4 (2)
C28—C27—C39—O39	-133.9 (3)	C76—C77—C89—O89	-101.6 (3)
C26—C27—C39—O40	-137.4 (3)	C78—C77—C89—O89	61.1 (3)
C28—C27—C39—O40	41.5 (3)	C76—C77—C89—O90	80.9 (3)
O39—C39—O40—C40	9.9 (4)	C78—C77—C89—O90	-116.4 (2)
C27—C39—O40—C40	-165.4 (2)	O89—C89—O90—C90	1.2 (4)
O45—C28—C41—O41	77.4 (3)	C77—C89—O90—C90	178.6 (3)
C27—C28—C41—O41	-170.7 (3)	O95—C78—C91—O91	55.5 (3)
C29—C28—C41—O41	-35.1 (4)	C79—C78—C91—O91	-57.1 (3)
O45—C28—C41—O42	-97.6 (3)	C77—C78—C91—O91	170.4 (3)
C27—C28—C41—O42	14.3 (3)	O95—C78—C91—O92	-121.7 (2)
C29—C28—C41—O42	149.9 (2)	C79—C78—C91—O92	125.8 (2)
O41—C41—O42—C42	1.5 (5)	C77—C78—C91—O92	-6.7 (3)
C28—C41—O42—C42	176.3 (3)	O91—C91—O92—C92	1.0 (4)
C24—C29—C43—O43	165.4 (3)	C78—C91—O92—C92	178.0 (2)
C28—C29—C43—O43	-5.0 (4)	C74—C79—C93—O93	171.0 (3)
C24—C29—C43—O44	-13.0 (4)	C78—C79—C93—O93	-3.1 (4)
C28—C29—C43—O44	176.6 (2)	C74—C79—C93—O94	-8.2 (4)

O43—C43—O44—C44	-1.8 (4)	C78—C79—C93—O94	177.7 (2)
C29—C43—O44—C44	176.6 (2)	O93—C93—O94—C94	-4.1 (4)
C41—C28—O45—C25	179.2 (2)	C79—C93—O94—C94	175.1 (2)
C27—C28—O45—C25	55.2 (2)	C91—C78—O95—C75	-177.37 (18)
C29—C28—O45—C25	-54.7 (2)	C79—C78—O95—C75	-54.23 (18)
O36—C25—O45—C28	175.9 (2)	C77—C78—O95—C75	54.77 (18)
C24—C25—O45—C28	54.5 (2)	O86—C75—O95—C78	176.02 (18)
C26—C25—O45—C28	-54.8 (2)	C74—C75—O95—C78	54.32 (18)
O51—C51—C52—C61	175.9 (3)	C76—C75—O95—C78	-54.80 (18)

Table 21.Hydrogen-bond geometry (Å, º) for (**18**)

D—H···*A*	*D*—H	H···*A*	*D*···*A*	*D*—H···*A*
N16—H16···O30	0.86 (2)	2.54 (2)	3.301 (3)	148 (3)
N16—H16···O35	0.86 (2)	2.62 (3)	3.313 (3)	139 (3)
C23—H23···O38	1.00	2.27	3.248 (3)	166
C31—H31*B*···S20[i]	0.98	3.02	3.906 (3)	151
C35—H35*A*···O39[ii]	0.98	2.43	3.270 (4)	143
C36—H36*C*···O87[iii]	0.98	2.41	3.355 (3)	161
C38—H38*A*···O90[iii]	0.98	2.45	3.353 (3)	153
C40—H40*A*···O1	0.98	2.32	2.999 (4)	126
C42—H42*A*···O45[iv]	0.98	2.45	3.254 (4)	139
N66—H66···O80	0.87 (2)	2.40 (3)	3.131 (3)	141 (3)
N66—H66···O85	0.87 (2)	2.43 (3)	3.166 (3)	143 (3)
C73—H73···O88	1.00	2.41	3.382 (3)	163
C83—H83*B*···O83[v]	0.98	2.55	3.424 (4)	148
C83—H83*B*···O95[v]	0.98	2.52	3.006 (3)	110
C86—H86*C*···O37[iii]	0.98	2.48	3.389 (3)	155
C90—H90*B*···O84[vi]	0.98	2.33	3.303 (4)	170
C90—H90*C*···O51	0.98	2.62	3.554 (6)	158

Symmetry codes: (i) -*x*+1, -*y*+1, -*z*+2; (ii) *x*-1, *y*, *z*; (iii) -*x*+1, -*y*+1, -*z*+1; (iv) -*x*+2, -*y*+2, -*z*+2; (v) -*x*, -*y*+1, -*z*+1; (vi) *x*+1, *y*, *z*.

Table. S22. Crystal data of **26**.

$C_{32}H_{42}N_6S_2$·0.5(C_2H_3N)	Z = 8
M_r = 595.36	F(000) = 2552
Triclinic, *P*-1 (no.2)	D_x = 1.221 Mg m^{-3}
a = 13.7588 (6) Å	Cu *K* radiation, = 1.54178 Å

b = 23.3483 (9) Å	Cell parameters from 9126 reflections
c = 23.3963 (9) Å	= 3.7–72.1°
= 60.101 (2)°	= 1.74 mm^{-1}
= 84.894 (3)°	T = 123 K
= 84.516 (3)°	Blocks, colourless
V = 6478.3 (5) Å^3	0.40 × 0.18 × 0.06 mm

Table S23. Data collection of **26**.

Bruker D8 VENTUREdiffractometer with Photon100 detector	24776 independent reflections
Radiation source: INCOATEC microfocus sealed tube	16926 reflections with $I > 2\ (I)$
Detector resolution: 10.4167 pixels mm^{-1}	R_{int} = 0.067
rotation in and , 0.5°, shutterless scans	$_{max}$ = 72.7°, $_{min}$ = 2.2°
Absorption correction: multi-scan *SADABS* (Sheldrick, 2014)	h = -16 17
T_{min} = 0.649, T_{max} = 0.864	k = -28 23
67389 measured reflections	l = -28 28

Table. S24. Refinement of **26.**

Refinement on F^2	Primary atom site location: structure-invariant direct methods
Least-squares matrix: full	Secondary atom site location: difference Fourier map
$R[F^2 > 2\ (F^2)]$ = 0.115	Hydrogen site location: inferred from neighbouring sites
$wR(F^2)$ = 0.321	H-atom parameters constrained
S = 1.01	$w = 1/[\ ^2(F_o^2) + (0.2P)^2 + 10.P]$ where $P = (F_o^2 + 2F_c^2)/3$
24776 reflections	$(\ /\ )_{max} < 0.001$
1485 parameters	$_{max}$ = 1.33 e Å^{-3}
1254 restraints	$_{min}$ = -0.79 e Å^{-3}

Table. S25.Fractional atomic coordinates and isotropic or equivalent isotropic displacement parameters (Å^2 of **26**)

	x	y	z	U_{iso}*/U_{eq}	Occ. (<1)
C101	0.9145 (5)	1.0172 (3)	0.5724 (3)	0.0440 (14)	
H10A	0.9436	1.0563	0.5346	0.053*	
H10B	0.9340	0.9783	0.5667	0.053*	
C102	0.9556 (5)	1.0056 (3)	0.6387 (3)	0.0423 (14)	
H10C	1.0052	0.9679	0.6542	0.051*	
H10D	0.9888	1.0454	0.6293	0.051*	
C103	0.8777 (5)	0.9913 (3)	0.6938 (3)	0.0409 (13)	

C104	0.8303 (5)	0.9305 (3)	0.7271 (3)	0.0379 (12)	
C105	0.7368 (5)	0.9302 (3)	0.7557 (3)	0.0380 (12)	
H105	0.7058	0.8896	0.7800	0.046*	
C106	0.6881 (5)	0.9875 (3)	0.7496 (3)	0.0403 (13)	
C107	0.7422 (5)	1.0419 (4)	0.7288 (3)	0.0453 (14)	
H107	0.7146	1.0786	0.7329	0.054*	
C108	0.8362 (5)	1.0432 (4)	0.7020 (3)	0.0441 (14)	
H108	0.8730	1.0806	0.6889	0.053*	
C109	0.5776 (5)	0.9925 (4)	0.7513 (3)	0.0467 (15)	
H10E	0.5525	1.0181	0.7733	0.056*	
H10F	0.5531	0.9475	0.7776	0.056*	
C110	0.5374 (5)	1.0270 (4)	0.6797 (3)	0.0431 (14)	
H11A	0.4831	1.0021	0.6797	0.052*	
H11B	0.5111	1.0724	0.6676	0.052*	
C111	0.6165 (5)	1.0305 (3)	0.6281 (3)	0.0357 (12)	
C112	0.6643 (5)	1.0878 (3)	0.5916 (3)	0.0404 (13)	
H112	0.6328	1.1285	0.5850	0.049*	
C113	0.7581 (5)	1.0871 (3)	0.5643 (3)	0.0392 (13)	
H113	0.7904	1.1272	0.5398	0.047*	
C114	0.8052 (5)	1.0277 (3)	0.5725 (3)	0.0362 (12)	
C115	0.7468 (5)	0.9740 (3)	0.5954 (3)	0.0348 (12)	
H115	0.7709	0.9368	0.5911	0.042*	
C116	0.6537 (4)	0.9740 (3)	0.6245 (3)	0.0336 (11)	
C117	0.8672 (5)	0.8740 (3)	0.7184 (3)	0.0383 (12)	
H117	0.9232	0.8783	0.6900	0.046*	
N118	0.8253 (4)	0.8187 (3)	0.7485 (3)	0.0399 (11)	
N119	0.8589 (4)	0.7746 (3)	0.7273 (3)	0.0415 (11)	
H119	0.9062	0.7851	0.6965	0.050*	
C120	0.8195 (4)	0.7149 (3)	0.7538 (3)	0.0379 (12)	
S120	0.84727 (12)	0.66829 (8)	0.71680 (8)	0.0431 (4)	
N121	0.7612 (4)	0.6969 (3)	0.8074 (3)	0.0427 (12)	
H121	0.7486	0.7249	0.8223	0.051*	
C122	0.7170 (5)	0.6334 (3)	0.8428 (3)	0.0454 (14)	
H122	0.6964	0.6217	0.8101	0.054*	
C123	0.7894 (5)	0.5792 (4)	0.8879 (4)	0.0492 (15)	
H12A	0.8492	0.5777	0.8617	0.059*	
H12B	0.8082	0.5893	0.9217	0.059*	

C124	0.7446 (7)	0.5131 (4)	0.9213 (4)	0.067 (2)	
H12C	0.7917	0.4785	0.9514	0.081*	
H12D	0.7301	0.5016	0.8876	0.081*	
C125	0.6507 (7)	0.5149 (4)	0.9604 (4)	0.065 (2)	
H12E	0.6191	0.4725	0.9786	0.078*	
H12F	0.6666	0.5204	0.9978	0.078*	
C126	0.5810 (7)	0.5712 (5)	0.9171 (5)	0.077 (3)	
H12G	0.5226	0.5729	0.9443	0.093*	
H12H	0.5592	0.5627	0.8829	0.093*	
C127	0.6275 (5)	0.6383 (4)	0.8833 (4)	0.062 (2)	
H12I	0.5804	0.6738	0.8544	0.075*	
H12J	0.6464	0.6487	0.9168	0.075*	
C128	0.6055 (4)	0.9119 (3)	0.6584 (3)	0.0347 (12)	
H128	0.5568	0.9045	0.6924	0.042*	
N129	0.6293 (4)	0.8670 (3)	0.6422 (2)	0.0373 (11)	
N130	0.5834 (4)	0.8091 (3)	0.6761 (2)	0.0385 (11)	
H130	0.5458	0.8006	0.7116	0.046*	
C131	0.5970 (4)	0.7651 (3)	0.6542 (3)	0.0343 (12)	
S131	0.53925 (12)	0.69347 (8)	0.69404 (8)	0.0409 (4)	
N132	0.6572 (4)	0.7826 (3)	0.6012 (3)	0.0460 (13)	
H132	0.6830	0.8210	0.5852	0.055*	
C133	0.6855 (6)	0.7462 (4)	0.5668 (4)	0.0557 (17)	
H133	0.6854	0.6978	0.5995	0.067*	
C134	0.7891 (6)	0.7624 (5)	0.5350 (4)	0.067 (2)	
H13A	0.8343	0.7548	0.5691	0.080*	
H13B	0.8114	0.7321	0.5176	0.080*	
C135	0.7927 (9)	0.8312 (6)	0.4811 (5)	0.083 (3)	
H13C	0.8583	0.8378	0.4584	0.099*	
H13D	0.7828	0.8612	0.4999	0.099*	
C136	0.7159 (10)	0.8493 (5)	0.4311 (4)	0.093 (3)	
H13E	0.7356	0.8281	0.4040	0.111*	
H13F	0.7125	0.8978	0.4013	0.111*	
C137	0.6127 (9)	0.8273 (7)	0.4648 (5)	0.093 (3)	
H13G	0.5853	0.8574	0.4813	0.112*	
H13H	0.5689	0.8313	0.4315	0.112*	
C138	0.6146 (8)	0.7618 (6)	0.5177 (5)	0.084 (3)	
H13I	0.6289	0.7314	0.4997	0.100*	

H13J	0.5488	0.7531	0.5400	0.100*	
C201	0.4191 (6)	0.4856 (4)	0.7662 (4)	0.0585 (18)	
H20A	0.4417	0.5298	0.7334	0.070*	
H20B	0.4524	0.4529	0.7547	0.070*	
C202	0.4482 (5)	0.4681 (4)	0.8366 (4)	0.0533 (16)	
H20C	0.4796	0.4228	0.8587	0.064*	
H20D	0.4967	0.4990	0.8320	0.064*	
C203	0.3622 (5)	0.4718 (3)	0.8794 (3)	0.0402 (13)	
C204	0.3156 (5)	0.5322 (3)	0.8698 (3)	0.0362 (12)	
C205	0.2176 (5)	0.5342 (3)	0.8901 (3)	0.0372 (12)	
H205	0.1864	0.5749	0.8836	0.045*	
C206	0.1643 (5)	0.4778 (3)	0.9196 (3)	0.0382 (12)	
C207	0.2179 (5)	0.4172 (3)	0.9421 (3)	0.0430 (13)	
H207	0.1871	0.3774	0.9715	0.052*	
C208	0.3139 (5)	0.4144 (3)	0.9225 (3)	0.0451 (14)	
H208	0.3487	0.3725	0.9385	0.054*	
C209	0.0535 (5)	0.4838 (4)	0.9126 (4)	0.0505 (16)	
H20E	0.0269	0.5269	0.9075	0.061*	
H20F	0.0228	0.4484	0.9534	0.061*	
C210	0.0263 (5)	0.4782 (4)	0.8520 (4)	0.0494 (15)	
H21A	-0.0027	0.4352	0.8683	0.059*	
H21B	-0.0240	0.5136	0.8275	0.059*	
C211	0.1130 (5)	0.4836 (3)	0.8046 (3)	0.0454 (14)	
C212	0.1640 (6)	0.4269 (4)	0.8119 (3)	0.0495 (15)	
H212	0.1315	0.3868	0.8312	0.059*	
C213	0.2620 (6)	0.4271 (4)	0.7915 (4)	0.0556 (17)	
H213	0.2963	0.3874	0.7973	0.067*	
C214	0.3092 (6)	0.4856 (4)	0.7627 (3)	0.0495 (15)	
C215	0.2539 (5)	0.5447 (4)	0.7427 (3)	0.0458 (14)	
H215	0.2830	0.5856	0.7153	0.055*	
C216	0.1553 (5)	0.5440 (3)	0.7629 (3)	0.0424 (13)	
C217	0.3641 (5)	0.5944 (3)	0.8336 (3)	0.0379 (12)	
H217	0.4148	0.6003	0.8011	0.045*	
N218	0.3368 (4)	0.6408 (3)	0.8467 (3)	0.0396 (11)	
N219	0.3835 (4)	0.6992 (3)	0.8121 (3)	0.0409 (12)	
H219	0.4166	0.7092	0.7747	0.049*	
C220	0.3772 (5)	0.7408 (3)	0.8373 (3)	0.0383 (13)	

S220	0.43706 (14)	0.81057 (9)	0.79928 (8)	0.0494 (4)	
N221	0.3209 (4)	0.7214 (3)	0.8927 (2)	0.0404 (12)	
H221	0.2898	0.6850	0.9078	0.048*	
C222	0.3079 (5)	0.7578 (4)	0.9300 (4)	0.0506 (17)	
H222	0.3011	0.8064	0.8982	0.061*	
C223	0.2130 (6)	0.7363 (5)	0.9737 (4)	0.0615 (19)	
H22A	0.1945	0.7676	0.9903	0.074*	
H22B	0.1600	0.7393	0.9462	0.074*	
C224	0.2203 (8)	0.6704 (5)	1.0290 (5)	0.079 (2)	
H22C	0.2327	0.6378	1.0135	0.095*	
H22D	0.1586	0.6606	1.0565	0.095*	
C225	0.3082 (10)	0.6662 (9)	1.0709 (6)	0.143 (6)	
H22E	0.2951	0.6979	1.0876	0.171*	
H22F	0.3170	0.6211	1.1090	0.171*	
C226	0.4013 (9)	0.6840 (9)	1.0235 (7)	0.149 (6)	
H22G	0.4584	0.6807	1.0482	0.179*	
H22H	0.4135	0.6517	1.0077	0.179*	
C227	0.3927 (7)	0.7437 (7)	0.9727 (5)	0.092 (3)	
H22I	0.4537	0.7519	0.9446	0.110*	
H22J	0.3867	0.7758	0.9890	0.110*	
C228	0.0979 (5)	0.6061 (3)	0.7412 (3)	0.0432 (14)	
H228	0.0292	0.6054	0.7504	0.052*	
N229	0.1376 (4)	0.6623 (3)	0.7098 (3)	0.0426 (12)	
N230	0.0717 (4)	0.7164 (3)	0.6828 (3)	0.0451 (12)	
H230	0.0087	0.7102	0.6898	0.054*	
C231	0.1012 (5)	0.7787 (3)	0.6461 (3)	0.0387 (12)	
S231	0.01891 (11)	0.83987 (8)	0.60091 (8)	0.0413 (3)	
N232	0.1952 (4)	0.7878 (3)	0.6485 (3)	0.0397 (11)	
H232	0.2333	0.7528	0.6728	0.048*	
C233	0.2381 (5)	0.8517 (3)	0.6135 (3)	0.0379 (12)	
H233	0.2078	0.8784	0.5698	0.045*	
C234	0.3478 (5)	0.8421 (3)	0.6015 (3)	0.0426 (14)	
H23A	0.3592	0.8190	0.5755	0.051*	
H23B	0.3787	0.8142	0.6443	0.051*	
C235	0.3944 (5)	0.9094 (3)	0.5641 (3)	0.0463 (15)	
H23C	0.4661	0.9022	0.5599	0.056*	
H23D	0.3695	0.9349	0.5191	0.056*	

C236	0.3718 (6)	0.9487 (4)	0.5991 (4)	0.0576 (18)	
H23E	0.3995	0.9926	0.5723	0.069*	
H23F	0.4035	0.9257	0.6419	0.069*	
C237	0.2621 (6)	0.9583 (4)	0.6112 (5)	0.0586 (19)	
H23G	0.2302	0.9843	0.5685	0.070*	
H23H	0.2502	0.9828	0.6358	0.070*	
C238	0.2185 (5)	0.8895 (4)	0.6516 (4)	0.0500 (16)	
H23I	0.2486	0.8642	0.6949	0.060*	
H23J	0.1472	0.8950	0.6594	0.060*	
C301	0.9191 (5)	0.7501 (4)	-0.0036 (4)	0.0499 (16)	
H30A	0.9452	0.7326	-0.0330	0.060*	
H30B	0.9445	0.7203	0.0408	0.060*	
C302	0.9551 (5)	0.8219 (4)	-0.0311 (3)	0.0453 (14)	
H30C	1.0075	0.8194	-0.0033	0.054*	
H30D	0.9840	0.8375	-0.0764	0.054*	
C303	0.8771 (5)	0.8716 (3)	-0.0329 (3)	0.0373 (12)	
C304	0.8389 (4)	0.8749 (3)	0.0227 (3)	0.0335 (11)	
C305	0.7449 (5)	0.9016 (3)	0.0236 (3)	0.0328 (11)	
H305	0.7202	0.9035	0.0619	0.039*	
C306	0.6854 (5)	0.9258 (3)	-0.0302 (3)	0.0355 (12)	
C307	0.7317 (5)	0.9362 (3)	-0.0892 (3)	0.0388 (13)	
H307	0.6984	0.9616	-0.1290	0.047*	
C308	0.8261 (5)	0.9102 (3)	-0.0911 (3)	0.0423 (14)	
H308	0.8568	0.9185	-0.1323	0.051*	
C309	0.5764 (5)	0.9247 (3)	-0.0206 (3)	0.0415 (13)	
H30E	0.5465	0.9646	-0.0580	0.050*	
H30F	0.5559	0.9261	0.0204	0.050*	
C310	0.5374 (5)	0.8603 (3)	-0.0161 (3)	0.0421 (14)	
H31A	0.4862	0.8427	0.0199	0.051*	
H31B	0.5069	0.8731	-0.0579	0.051*	
C311	0.6165 (4)	0.8060 (3)	-0.0035 (3)	0.0394 (12)	
C312	0.6594 (5)	0.7990 (3)	-0.0558 (4)	0.0456 (14)	
H312	0.6226	0.8128	-0.0935	0.055*	
C313	0.7541 (5)	0.7725 (4)	-0.0548 (4)	0.0476 (15)	
H313	0.7819	0.7688	-0.0915	0.057*	
C314	0.8086 (5)	0.7511 (3)	0.0009 (4)	0.0434 (14)	
C315	0.7596 (4)	0.7444 (3)	0.0572 (3)	0.0368 (12)	

H315	0.7911	0.7205	0.0979	0.044*	
C316	0.6635 (5)	0.7721 (3)	0.0566 (3)	0.0377 (12)	
C317	0.8902 (4)	0.8408 (3)	0.0842 (3)	0.0354 (12)	
H317	0.9393	0.8068	0.0910	0.043*	
N318	0.8689 (4)	0.8569 (2)	0.1289 (2)	0.0362 (11)	
N319	0.9184 (4)	0.8226 (3)	0.1867 (2)	0.0374 (11)	
H319	0.9567	0.7872	0.1946	0.045*	
C320	0.9068 (4)	0.8443 (3)	0.2310 (3)	0.0357 (12)	
S320	0.96720 (12)	0.80372 (8)	0.30145 (7)	0.0413 (4)	
N321	0.8474 (4)	0.8988 (2)	0.2140 (2)	0.0360 (10)	
H321	0.8231	0.9178	0.1747	0.043*	
C322	0.8192 (5)	0.9298 (4)	0.2549 (3)	0.0428 (14)	
H322	0.8079	0.8941	0.3016	0.051*	
C323	0.7237 (5)	0.9717 (4)	0.2310 (4)	0.0525 (16)	
H32A	0.6753	0.9446	0.2280	0.063*	
H32B	0.6973	0.9849	0.2638	0.063*	
C324	0.7373 (6)	1.0330 (5)	0.1646 (4)	0.0626 (19)	
H32C	0.6755	1.0610	0.1538	0.075*	
H32D	0.7522	1.0199	0.1303	0.075*	
C325	0.8194 (7)	1.0730 (5)	0.1635 (5)	0.079 (3)	
H32E	0.7993	1.0930	0.1917	0.094*	
H32F	0.8316	1.1094	0.1179	0.094*	
C326	0.9142 (6)	1.0294 (5)	0.1884 (5)	0.070 (2)	
H32G	0.9393	1.0147	0.1565	0.084*	
H32H	0.9641	1.0559	0.1907	0.084*	
C327	0.8993 (6)	0.9721 (4)	0.2526 (5)	0.0627 (19)	
H32I	0.8818	0.9869	0.2854	0.075*	
H32J	0.9614	0.9446	0.2653	0.075*	
C328	0.6280 (5)	0.7797 (3)	0.1135 (3)	0.0394 (13)	
H328	0.5700	0.8067	0.1098	0.047*	
N329	0.6716 (4)	0.7518 (3)	0.1680 (3)	0.0429 (12)	
N330	0.6382 (4)	0.7732 (3)	0.2120 (3)	0.0438 (12)	
H330	0.5890	0.8030	0.2020	0.053*	
C331	0.6806 (4)	0.7487 (3)	0.2705 (3)	0.0396 (13)	
S331	0.65585 (12)	0.78898 (9)	0.31426 (9)	0.0451 (4)	
N332	0.7387 (4)	0.6949 (3)	0.2894 (3)	0.0430 (12)	
H332	0.7477	0.6779	0.2630	0.052*	

C333	0.7896 (5)	0.6614 (3)	0.3514 (3)	0.0420 (14)	
H333	0.8144	0.6958	0.3588	0.050*	
C334	0.7217 (5)	0.6207 (4)	0.4087 (3)	0.0489 (16)	
H33A	0.6975	0.5853	0.4030	0.059*	
H33B	0.6647	0.6490	0.4107	0.059*	
C335	0.7753 (6)	0.5901 (4)	0.4723 (4)	0.0597 (19)	
H33C	0.7936	0.6257	0.4799	0.072*	
H33D	0.7311	0.5616	0.5097	0.072*	
C336	0.8666 (7)	0.5492 (4)	0.4703 (4)	0.0636 (19)	
H33E	0.8475	0.5090	0.4710	0.076*	
H33F	0.9035	0.5348	0.5102	0.076*	
C337	0.9322 (6)	0.5877 (4)	0.4092 (4)	0.064 (2)	
H33G	0.9613	0.6229	0.4126	0.076*	
H33H	0.9861	0.5576	0.4068	0.076*	
C338	0.8760 (5)	0.6186 (4)	0.3466 (4)	0.0539 (17)	
H33I	0.9195	0.6459	0.3080	0.065*	
H33J	0.8529	0.5834	0.3402	0.065*	
C401	0.4462 (6)	0.5783 (4)	0.5169 (4)	0.0567 (17)	
H40A	0.4731	0.5757	0.4776	0.068*	
H40B	0.4758	0.5403	0.5562	0.068*	
C402	0.4765 (5)	0.6449 (4)	0.5109 (3)	0.0491 (15)	
H40C	0.5095	0.6343	0.5513	0.059*	
H40D	0.5240	0.6658	0.4730	0.059*	
C403	0.3913 (5)	0.6932 (3)	0.5019 (3)	0.0415 (13)	
C404	0.3485 (5)	0.7358 (3)	0.4411 (3)	0.0374 (12)	
C405	0.2484 (5)	0.7572 (3)	0.4404 (3)	0.0375 (12)	
H405	0.2195	0.7852	0.3993	0.045*	
C406	0.1927 (5)	0.7385 (3)	0.4975 (4)	0.0460 (14)	
C407	0.2413 (6)	0.7088 (4)	0.5567 (4)	0.0489 (15)	
H407	0.2069	0.7030	0.5962	0.059*	
C408	0.3393 (6)	0.6880 (4)	0.5580 (4)	0.0488 (15)	
H408	0.3718	0.6696	0.5981	0.059*	
C409	0.0834 (5)	0.7355 (4)	0.4993 (4)	0.0534 (17)	
H40E	0.0594	0.7660	0.4546	0.064*	
H40F	0.0519	0.7506	0.5296	0.064*	
C410	0.0533 (6)	0.6636 (4)	0.5227 (4)	0.0528 (16)	
H41A	0.0230	0.6448	0.5679	0.063*	

H41B	0.0038	0.6658	0.4933	0.063*	
C411	0.1395 (5)	0.6183 (3)	0.5223 (3)	0.0448 (14)	
C412	0.1865 (6)	0.5753 (4)	0.5818 (3)	0.0524 (16)	
H412	0.1507	0.5616	0.6227	0.063*	
C413	0.2819 (6)	0.5533 (4)	0.5816 (3)	0.0508 (16)	
H413	0.3111	0.5241	0.6224	0.061*	
C414	0.3373 (5)	0.5727 (3)	0.5227 (3)	0.0449 (14)	
C415	0.2866 (5)	0.6025 (3)	0.4648 (3)	0.0409 (13)	
H415	0.3188	0.6075	0.4250	0.049*	
C416	0.1882 (5)	0.6253 (3)	0.4646 (3)	0.0385 (12)	
C417	0.4045 (5)	0.7568 (3)	0.3800 (3)	0.0429 (14)	
H417	0.4727	0.7453	0.3806	0.051*	
N418	0.3638 (4)	0.7909 (3)	0.3245 (3)	0.0430 (12)	
N419	0.4288 (4)	0.8182 (3)	0.2706 (3)	0.0451 (13)	
H419	0.4920	0.8115	0.2767	0.054*	
C420	0.3972 (5)	0.8550 (3)	0.2090 (3)	0.0388 (13)	
S420	0.47706 (12)	0.90059 (8)	0.14770 (8)	0.0447 (4)	
N421	0.3031 (4)	0.8526 (3)	0.2001 (3)	0.0404 (11)	
H421	0.2664	0.8270	0.2348	0.048*	
C422	0.2576 (5)	0.8897 (3)	0.1367 (3)	0.0424 (14)	
H422	0.2866	0.9339	0.1115	0.051*	
C423	0.1480 (5)	0.9013 (3)	0.1469 (3)	0.0433 (14)	
H42A	0.1181	0.8581	0.1735	0.052*	
H42B	0.1367	0.9258	0.1715	0.052*	
C424	0.0997 (6)	0.9404 (4)	0.0811 (4)	0.0550 (17)	
H42C	0.0281	0.9436	0.0891	0.066*	
H42D	0.1228	0.9858	0.0574	0.066*	
C425	0.1222 (6)	0.9088 (4)	0.0386 (4)	0.0600 (19)	
H42E	0.0935	0.9375	-0.0049	0.072*	
H42F	0.0915	0.8658	0.0596	0.072*	
C426	0.2308 (6)	0.8976 (5)	0.0283 (4)	0.0621 (19)	
H42G	0.2613	0.9407	0.0039	0.074*	
H42H	0.2425	0.8751	0.0018	0.074*	
C427	0.2769 (5)	0.8550 (4)	0.0955 (4)	0.0491 (15)	
H42I	0.2484	0.8112	0.1192	0.059*	
H42J	0.3482	0.8481	0.0889	0.059*	
C428	0.1426 (5)	0.6627 (3)	0.4006 (3)	0.0363 (12)	

H428	0.0923	0.6961	0.3935	0.044*	
N429	0.1712 (4)	0.6498 (3)	0.3548 (3)	0.0400 (11)	
N430	0.1247 (4)	0.6860 (3)	0.2955 (3)	0.0450 (13)	
H430	0.0921	0.7238	0.2855	0.054*	
C431	0.1308 (5)	0.6615 (3)	0.2536 (3)	0.0396 (13)	
S431	0.06754 (15)	0.69941 (9)	0.18461 (9)	0.0531 (5)	
N432	0.1900 (4)	0.6067 (3)	0.2720 (3)	0.0429 (12)	
H432	0.2284	0.5956	0.3046	0.051*	
C433	0.1964 (5)	0.5635 (3)	0.2426 (3)	0.0456 (15)	
H433	0.1855	0.5908	0.1943	0.055*	
C434	0.2999 (7)	0.5300 (5)	0.2527 (4)	0.064 (2)	
H43A	0.3099	0.5072	0.2263	0.077*	
H43B	0.3478	0.5647	0.2353	0.077*	
C435	0.3210 (8)	0.4811 (5)	0.3226 (4)	0.074 (2)	
H43C	0.3856	0.4580	0.3241	0.089*	
H43D	0.3235	0.5047	0.3477	0.089*	
C436	0.2457 (10)	0.4324 (5)	0.3534 (5)	0.089 (3)	
H43E	0.2506	0.4045	0.3322	0.107*	
H43F	0.2590	0.4032	0.4006	0.107*	
C437	0.1462 (10)	0.4620 (6)	0.3483 (6)	0.098 (3)	
H43G	0.1386	0.4852	0.3743	0.118*	
H43H	0.0997	0.4265	0.3677	0.118*	
C438	0.1200 (8)	0.5122 (5)	0.2754 (5)	0.079 (2)	
H43I	0.1174	0.4883	0.2507	0.094*	
H43J	0.0549	0.5345	0.2749	0.094*	
N1A	0.9968 (8)	0.6600 (5)	0.9022 (5)	0.075 (3)*	0.729 (8)
C1A	1.0496 (10)	0.6982 (7)	0.8659 (6)	0.078 (4)*	0.729 (8)
C2A	1.0948 (10)	0.7535 (6)	0.8072 (6)	0.078 (3)*	0.729 (8)
H2A1	1.1615	0.7569	0.8163	0.116*	0.729 (8)
H2A2	1.0967	0.7454	0.7698	0.116*	0.729 (8)
H2A3	1.0563	0.7948	0.7964	0.116*	0.729 (8)
N1B	1.146 (2)	0.8242 (13)	0.7851 (14)	0.075 (3)*	0.271 (8)
C1B	1.092 (2)	0.7835 (15)	0.8065 (17)	0.078 (4)*	0.271 (8)
C2B	1.041 (3)	0.7219 (16)	0.845 (2)	0.078 (3)*	0.271 (8)
H2B1	0.9760	0.7277	0.8277	0.116*	0.271 (8)
H2B2	1.0787	0.6857	0.8422	0.116*	0.271 (8)
H2B3	1.0328	0.7111	0.8915	0.116*	0.271 (8)

N1C	0.5077 (8)	0.6036 (6)	0.3386 (5)	0.081 (3)*	0.729 (8)
C1C	0.4586 (12)	0.6387 (8)	0.2965 (7)	0.089 (4)*	0.729 (8)
C2C	0.4143 (12)	0.6919 (7)	0.2360 (6)	0.088 (4)*	0.729 (8)
H2C1	0.3453	0.6842	0.2368	0.131*	0.729 (8)
H2C2	0.4195	0.7347	0.2336	0.131*	0.729 (8)
H2C3	0.4488	0.6923	0.1973	0.131*	0.729 (8)
N1D	0.348 (2)	0.7159 (16)	0.1750 (14)	0.081 (3)*	0.271 (8)
C1D	0.407 (2)	0.693 (2)	0.2129 (16)	0.089 (4)*	0.271 (8)
C2D	0.461 (3)	0.654 (2)	0.2739 (18)	0.088 (4)*	0.271 (8)
H2D1	0.5296	0.6641	0.2648	0.131*	0.271 (8)
H2D2	0.4325	0.6646	0.3076	0.131*	0.271 (8)
H2D3	0.4554	0.6065	0.2900	0.131*	0.271 (8)

Table S26. Atomic displacement parameters (Å^2) for **26**.

	U^{11}	U^{22}	U^{33}	U^{12}	U^{13}	U^{23}
C101	0.058 (3)	0.033 (3)	0.035 (3)	-0.013 (3)	0.007 (2)	-0.011 (3)
C102	0.041 (3)	0.037 (3)	0.047 (3)	-0.004 (2)	-0.001 (2)	-0.020 (3)
C103	0.042 (3)	0.044 (3)	0.034 (3)	0.000 (2)	-0.012 (2)	-0.017 (2)
C104	0.042 (3)	0.040 (3)	0.028 (3)	0.001 (2)	-0.004 (2)	-0.015 (2)
C105	0.052 (3)	0.041 (3)	0.017 (2)	0.000 (2)	-0.004 (2)	-0.011 (2)
C106	0.052 (3)	0.050 (3)	0.025 (3)	0.003 (2)	-0.006 (2)	-0.024 (3)
C107	0.059 (3)	0.051 (4)	0.040 (3)	0.002 (3)	-0.007 (3)	-0.033 (3)
C108	0.052 (3)	0.049 (4)	0.040 (3)	-0.004 (3)	-0.014 (2)	-0.026 (3)
C109	0.052 (3)	0.053 (4)	0.042 (3)	0.003 (3)	0.003 (3)	-0.031 (3)
C110	0.043 (3)	0.045 (4)	0.044 (3)	-0.001 (3)	-0.006 (2)	-0.024 (3)
C111	0.048 (3)	0.031 (3)	0.029 (3)	-0.001 (2)	-0.014 (2)	-0.013 (2)
C112	0.052 (3)	0.034 (3)	0.033 (3)	0.003 (2)	-0.014 (2)	-0.013 (2)
C113	0.060 (3)	0.024 (3)	0.026 (3)	-0.007 (2)	-0.009 (2)	-0.004 (2)
C114	0.053 (3)	0.031 (3)	0.021 (3)	-0.011 (2)	0.003 (2)	-0.009 (2)
C115	0.051 (3)	0.028 (3)	0.025 (3)	-0.009 (2)	0.004 (2)	-0.013 (2)
C116	0.046 (3)	0.035 (3)	0.017 (2)	-0.006 (2)	-0.003 (2)	-0.009 (2)
C117	0.043 (3)	0.039 (3)	0.033 (3)	-0.004 (2)	-0.002 (2)	-0.017 (2)
N118	0.045 (3)	0.033 (2)	0.038 (3)	0.002 (2)	-0.002 (2)	-0.015 (2)
N119	0.046 (3)	0.033 (2)	0.042 (3)	-0.004 (2)	0.005 (2)	-0.016 (2)
C120	0.035 (3)	0.032 (3)	0.035 (3)	0.002 (2)	-0.003 (2)	-0.009 (2)
S120	0.0471 (8)	0.0400 (9)	0.0432 (8)	-0.0069 (7)	-0.0006 (7)	-0.0208 (7)
N121	0.047 (3)	0.038 (3)	0.039 (3)	-0.006 (2)	0.003 (2)	-0.016 (2)

C122	0.047 (3)	0.046 (3)	0.036 (3)	-0.017 (3)	-0.001 (2)	-0.012 (3)
C123	0.047 (3)	0.048 (4)	0.045 (3)	-0.006 (3)	-0.004 (3)	-0.017 (3)
C124	0.087 (5)	0.049 (4)	0.058 (4)	-0.017 (4)	0.002 (4)	-0.019 (4)
C125	0.076 (4)	0.061 (4)	0.050 (4)	-0.029 (3)	0.001 (3)	-0.017 (3)
C126	0.058 (4)	0.073 (5)	0.062 (5)	-0.028 (4)	0.006 (4)	-0.002 (4)
C127	0.043 (3)	0.061 (4)	0.054 (4)	-0.003 (3)	0.008 (3)	-0.008 (3)
C128	0.042 (3)	0.035 (3)	0.027 (3)	-0.009 (2)	0.002 (2)	-0.015 (2)
N129	0.045 (3)	0.035 (2)	0.030 (2)	-0.011 (2)	0.006 (2)	-0.014 (2)
N130	0.055 (3)	0.033 (2)	0.028 (2)	-0.013 (2)	0.011 (2)	-0.015 (2)
C131	0.040 (3)	0.030 (3)	0.027 (3)	-0.003 (2)	0.003 (2)	-0.010 (2)
S131	0.0542 (9)	0.0293 (8)	0.0346 (8)	-0.0095 (6)	0.0103 (6)	-0.0130 (6)
N132	0.061 (3)	0.035 (3)	0.038 (3)	-0.011 (2)	0.015 (2)	-0.017 (2)
C133	0.071 (4)	0.047 (4)	0.050 (4)	-0.013 (3)	0.020 (3)	-0.027 (3)
C134	0.057 (4)	0.090 (5)	0.050 (4)	0.014 (4)	0.003 (3)	-0.036 (4)
C135	0.108 (6)	0.093 (6)	0.057 (4)	-0.028 (5)	0.027 (4)	-0.045 (4)
C136	0.157 (7)	0.065 (6)	0.040 (4)	-0.009 (5)	-0.001 (4)	-0.014 (4)
C137	0.104 (6)	0.128 (7)	0.061 (5)	0.023 (5)	-0.020 (4)	-0.059 (5)
C138	0.076 (5)	0.119 (7)	0.084 (5)	-0.003 (5)	0.002 (4)	-0.072 (5)
C201	0.057 (4)	0.061 (5)	0.055 (4)	0.008 (3)	0.005 (3)	-0.029 (4)
C202	0.048 (3)	0.046 (4)	0.060 (4)	0.007 (3)	-0.005 (3)	-0.023 (3)
C203	0.041 (3)	0.036 (3)	0.041 (3)	0.007 (2)	-0.011 (2)	-0.016 (2)
C204	0.044 (3)	0.032 (3)	0.027 (3)	-0.002 (2)	-0.001 (2)	-0.011 (2)
C205	0.046 (3)	0.029 (3)	0.031 (3)	-0.003 (2)	0.002 (2)	-0.011 (2)
C206	0.048 (3)	0.031 (3)	0.030 (3)	-0.008 (2)	0.003 (2)	-0.011 (2)
C207	0.059 (3)	0.029 (3)	0.034 (3)	-0.006 (2)	-0.010 (2)	-0.009 (2)
C208	0.058 (3)	0.027 (3)	0.044 (3)	0.006 (2)	-0.018 (3)	-0.011 (3)
C209	0.045 (3)	0.042 (4)	0.057 (4)	-0.010 (3)	0.007 (3)	-0.018 (3)
C210	0.050 (3)	0.036 (4)	0.046 (3)	-0.003 (3)	-0.006 (3)	-0.007 (3)
C211	0.053 (3)	0.038 (3)	0.036 (3)	-0.005 (2)	-0.014 (2)	-0.009 (2)
C212	0.062 (3)	0.041 (3)	0.040 (3)	-0.004 (3)	-0.013 (3)	-0.014 (3)
C213	0.074 (4)	0.050 (4)	0.042 (4)	0.004 (3)	-0.011 (3)	-0.022 (3)
C214	0.062 (3)	0.051 (3)	0.031 (3)	0.007 (3)	-0.002 (3)	-0.019 (3)
C215	0.057 (3)	0.046 (3)	0.027 (3)	-0.001 (3)	-0.005 (2)	-0.013 (3)
C216	0.053 (3)	0.031 (3)	0.028 (3)	0.004 (2)	-0.009 (2)	-0.003 (2)
C217	0.044 (3)	0.034 (3)	0.029 (3)	-0.005 (2)	0.003 (2)	-0.011 (2)
N218	0.043 (3)	0.032 (2)	0.035 (3)	-0.012 (2)	0.005 (2)	-0.010 (2)
N219	0.050 (3)	0.036 (3)	0.033 (3)	-0.015 (2)	0.010 (2)	-0.014 (2)

C220	0.043 (3)	0.041 (3)	0.029 (3)	-0.007 (2)	0.005 (2)	-0.016 (2)
S220	0.0685 (11)	0.0451 (9)	0.0385 (8)	-0.0244 (8)	0.0209 (8)	-0.0238 (8)
N221	0.044 (3)	0.047 (3)	0.029 (2)	-0.013 (2)	0.005 (2)	-0.017 (2)
C222	0.045 (3)	0.077 (5)	0.042 (3)	-0.014 (3)	0.009 (3)	-0.038 (3)
C223	0.051 (4)	0.091 (5)	0.048 (4)	-0.016 (4)	0.013 (3)	-0.038 (4)
C224	0.083 (5)	0.081 (6)	0.071 (5)	-0.012 (4)	0.026 (4)	-0.038 (4)
C225	0.095 (7)	0.193 (14)	0.054 (5)	-0.043 (7)	-0.009 (5)	0.011 (6)
C226	0.065 (6)	0.183 (11)	0.099 (7)	-0.012 (6)	-0.011 (5)	0.008 (7)
C227	0.062 (4)	0.163 (9)	0.054 (4)	-0.022 (5)	0.000 (4)	-0.054 (5)
C228	0.047 (3)	0.033 (3)	0.035 (3)	0.004 (2)	-0.003 (2)	-0.006 (2)
N229	0.051 (3)	0.032 (2)	0.032 (3)	0.004 (2)	-0.002 (2)	-0.008 (2)
N230	0.043 (3)	0.032 (2)	0.044 (3)	0.001 (2)	-0.001 (2)	-0.006 (2)
C231	0.049 (3)	0.033 (3)	0.029 (3)	0.000 (2)	0.002 (2)	-0.013 (2)
S231	0.0421 (8)	0.0322 (8)	0.0359 (8)	0.0000 (6)	0.0005 (6)	-0.0074 (6)
N232	0.045 (2)	0.027 (2)	0.036 (3)	0.0009 (19)	-0.004 (2)	-0.008 (2)
C233	0.046 (3)	0.033 (3)	0.030 (3)	0.000 (2)	-0.005 (2)	-0.012 (2)
C234	0.043 (3)	0.042 (3)	0.040 (3)	-0.004 (2)	0.002 (2)	-0.018 (3)
C235	0.047 (3)	0.042 (3)	0.040 (3)	-0.008 (3)	-0.005 (3)	-0.012 (3)
C236	0.060 (4)	0.055 (4)	0.058 (4)	-0.015 (3)	-0.011 (3)	-0.025 (4)
C237	0.063 (4)	0.050 (4)	0.074 (5)	-0.007 (3)	-0.003 (3)	-0.038 (4)
C238	0.053 (4)	0.052 (4)	0.051 (4)	-0.007 (3)	0.002 (3)	-0.031 (3)
C301	0.046 (3)	0.052 (4)	0.055 (4)	0.005 (3)	-0.001 (3)	-0.031 (3)
C302	0.045 (3)	0.055 (4)	0.033 (3)	-0.006 (3)	0.005 (2)	-0.020 (3)
C303	0.044 (3)	0.036 (3)	0.027 (2)	-0.013 (2)	0.000 (2)	-0.011 (2)
C304	0.046 (3)	0.026 (3)	0.026 (2)	-0.006 (2)	-0.006 (2)	-0.009 (2)
C305	0.053 (3)	0.020 (3)	0.021 (2)	-0.002 (2)	-0.006 (2)	-0.006 (2)
C306	0.051 (3)	0.025 (3)	0.027 (2)	0.001 (2)	-0.008 (2)	-0.010 (2)
C307	0.054 (3)	0.031 (3)	0.022 (2)	-0.005 (2)	-0.007 (2)	-0.004 (2)
C308	0.053 (3)	0.043 (3)	0.025 (3)	-0.013 (3)	0.000 (2)	-0.011 (2)
C309	0.052 (3)	0.036 (3)	0.025 (3)	0.007 (2)	-0.008 (2)	-0.007 (2)
C310	0.039 (3)	0.047 (3)	0.031 (3)	-0.001 (2)	-0.005 (2)	-0.011 (3)
C311	0.037 (3)	0.032 (3)	0.043 (3)	-0.007 (2)	-0.005 (2)	-0.013 (2)
C312	0.054 (3)	0.041 (4)	0.046 (3)	-0.009 (3)	-0.006 (3)	-0.023 (3)
C313	0.057 (3)	0.044 (4)	0.051 (3)	-0.005 (3)	-0.003 (3)	-0.030 (3)
C314	0.047 (3)	0.032 (3)	0.054 (3)	0.001 (2)	-0.002 (2)	-0.025 (3)
C315	0.042 (3)	0.017 (3)	0.044 (3)	0.001 (2)	-0.005 (2)	-0.010 (2)
C316	0.043 (3)	0.025 (3)	0.036 (3)	-0.002 (2)	-0.003 (2)	-0.008 (2)

C317	0.045 (3)	0.028 (3)	0.028 (2)	-0.001 (2)	-0.009 (2)	-0.009 (2)
N318	0.048 (3)	0.029 (3)	0.026 (2)	0.000 (2)	-0.0115 (19)	-0.008 (2)
N319	0.046 (3)	0.034 (3)	0.030 (2)	0.004 (2)	-0.013 (2)	-0.014 (2)
C320	0.042 (3)	0.031 (3)	0.030 (3)	-0.005 (2)	-0.009 (2)	-0.011 (2)
S320	0.0505 (8)	0.0429 (9)	0.0290 (7)	0.0065 (7)	-0.0133 (6)	-0.0163 (7)
N321	0.045 (3)	0.032 (2)	0.030 (2)	0.0007 (19)	-0.0089 (19)	-0.014 (2)
C322	0.046 (3)	0.047 (3)	0.040 (3)	0.002 (3)	-0.005 (2)	-0.026 (3)
C323	0.049 (3)	0.051 (4)	0.059 (4)	0.003 (3)	-0.006 (3)	-0.028 (3)
C324	0.063 (4)	0.067 (5)	0.049 (4)	0.011 (3)	-0.006 (3)	-0.023 (4)
C325	0.076 (5)	0.058 (5)	0.083 (6)	-0.011 (4)	0.004 (4)	-0.019 (4)
C326	0.060 (4)	0.075 (5)	0.074 (5)	-0.014 (4)	0.012 (4)	-0.037 (4)
C327	0.061 (4)	0.064 (4)	0.076 (5)	-0.007 (3)	-0.001 (3)	-0.044 (4)
C328	0.041 (3)	0.030 (3)	0.036 (3)	0.000 (2)	-0.007 (2)	-0.007 (2)
N329	0.047 (3)	0.035 (3)	0.039 (3)	0.003 (2)	-0.005 (2)	-0.012 (2)
N330	0.042 (3)	0.043 (3)	0.037 (2)	0.005 (2)	-0.007 (2)	-0.014 (2)
C331	0.034 (3)	0.037 (3)	0.038 (3)	-0.007 (2)	-0.002 (2)	-0.010 (2)
S331	0.0439 (8)	0.0443 (9)	0.0471 (9)	-0.0023 (7)	-0.0056 (7)	-0.0221 (8)
N332	0.047 (3)	0.038 (3)	0.037 (3)	0.000 (2)	-0.004 (2)	-0.013 (2)
C333	0.046 (3)	0.038 (3)	0.037 (3)	0.000 (2)	-0.010 (2)	-0.014 (3)
C334	0.047 (3)	0.055 (4)	0.039 (3)	-0.001 (3)	-0.002 (2)	-0.020 (3)
C335	0.076 (4)	0.055 (4)	0.039 (3)	-0.008 (3)	-0.009 (3)	-0.015 (3)
C336	0.079 (5)	0.057 (4)	0.052 (4)	0.004 (4)	-0.027 (3)	-0.023 (3)
C337	0.064 (4)	0.054 (5)	0.058 (4)	0.014 (3)	-0.023 (3)	-0.016 (4)
C338	0.052 (3)	0.050 (4)	0.043 (3)	0.007 (3)	-0.005 (3)	-0.012 (3)
C401	0.064 (4)	0.055 (4)	0.045 (4)	0.014 (3)	-0.022 (3)	-0.020 (3)
C402	0.050 (3)	0.049 (4)	0.030 (3)	-0.001 (3)	-0.008 (3)	-0.006 (3)
C403	0.045 (3)	0.035 (3)	0.035 (3)	-0.012 (2)	-0.002 (2)	-0.008 (2)
C404	0.046 (3)	0.024 (3)	0.035 (3)	-0.008 (2)	0.001 (2)	-0.008 (2)
C405	0.047 (3)	0.024 (3)	0.040 (3)	-0.004 (2)	0.000 (2)	-0.015 (2)
C406	0.056 (3)	0.040 (3)	0.054 (3)	-0.008 (3)	0.008 (3)	-0.033 (3)
C407	0.064 (4)	0.045 (4)	0.045 (3)	-0.014 (3)	0.009 (3)	-0.028 (3)
C408	0.061 (3)	0.041 (4)	0.039 (3)	-0.016 (3)	0.002 (3)	-0.015 (3)
C409	0.054 (3)	0.061 (4)	0.052 (4)	-0.006 (3)	0.009 (3)	-0.034 (3)
C410	0.056 (4)	0.060 (4)	0.045 (4)	-0.018 (3)	0.011 (3)	-0.028 (3)
C411	0.060 (3)	0.038 (3)	0.037 (3)	-0.020 (3)	0.000 (2)	-0.016 (2)
C412	0.081 (4)	0.041 (4)	0.028 (3)	-0.024 (3)	0.000 (3)	-0.009 (3)
C413	0.078 (4)	0.036 (4)	0.032 (3)	-0.009 (3)	-0.013 (3)	-0.011 (3)

C414	0.064 (3)	0.033 (3)	0.033 (3)	0.002 (3)	-0.016 (2)	-0.011 (3)
C415	0.063 (3)	0.025 (3)	0.031 (3)	0.002 (2)	-0.014 (2)	-0.010 (2)
C416	0.056 (3)	0.024 (3)	0.033 (3)	-0.007 (2)	-0.009 (2)	-0.010 (2)
C417	0.047 (3)	0.037 (3)	0.034 (3)	-0.004 (3)	0.002 (2)	-0.010 (2)
N418	0.048 (3)	0.040 (3)	0.035 (2)	-0.004 (2)	0.006 (2)	-0.014 (2)
N419	0.042 (3)	0.048 (3)	0.032 (2)	-0.001 (2)	0.0015 (19)	-0.011 (2)
C420	0.044 (3)	0.030 (3)	0.036 (3)	0.003 (2)	-0.001 (2)	-0.012 (2)
S420	0.0442 (8)	0.0379 (9)	0.0370 (8)	0.0013 (6)	0.0018 (6)	-0.0085 (7)
N421	0.041 (2)	0.036 (3)	0.034 (2)	-0.001 (2)	0.0014 (19)	-0.010 (2)
C422	0.047 (3)	0.039 (3)	0.034 (3)	-0.002 (2)	-0.003 (2)	-0.012 (3)
C423	0.044 (3)	0.035 (3)	0.044 (3)	0.001 (2)	0.001 (2)	-0.016 (3)
C424	0.054 (4)	0.043 (4)	0.059 (4)	-0.006 (3)	-0.013 (3)	-0.017 (3)
C425	0.063 (4)	0.062 (5)	0.057 (4)	-0.012 (3)	-0.012 (3)	-0.028 (4)
C426	0.065 (4)	0.076 (5)	0.047 (4)	-0.019 (4)	-0.003 (3)	-0.030 (4)
C427	0.046 (3)	0.059 (4)	0.049 (3)	-0.005 (3)	-0.001 (3)	-0.032 (3)
C428	0.045 (3)	0.024 (3)	0.036 (3)	-0.001 (2)	-0.008 (2)	-0.011 (2)
N429	0.057 (3)	0.026 (3)	0.033 (2)	0.005 (2)	-0.016 (2)	-0.011 (2)
N430	0.067 (3)	0.032 (3)	0.036 (3)	0.011 (2)	-0.019 (2)	-0.016 (2)
C431	0.050 (3)	0.031 (3)	0.035 (3)	0.003 (2)	-0.008 (2)	-0.015 (2)
S431	0.0804 (12)	0.0421 (10)	0.0352 (8)	0.0173 (8)	-0.0265 (8)	-0.0177 (8)
N432	0.056 (3)	0.035 (3)	0.038 (3)	0.011 (2)	-0.020 (2)	-0.017 (2)
C433	0.065 (4)	0.039 (3)	0.036 (3)	0.012 (3)	-0.017 (3)	-0.021 (3)
C434	0.075 (4)	0.063 (5)	0.052 (4)	0.019 (4)	-0.010 (3)	-0.030 (4)
C435	0.112 (6)	0.058 (4)	0.055 (4)	0.035 (4)	-0.030 (4)	-0.032 (4)
C436	0.161 (7)	0.065 (5)	0.046 (4)	-0.001 (5)	-0.023 (5)	-0.029 (4)
C437	0.132 (7)	0.069 (6)	0.078 (6)	-0.049 (5)	0.005 (5)	-0.019 (5)
C438	0.093 (6)	0.072 (5)	0.092 (5)	-0.014 (4)	-0.013 (4)	-0.053 (4)

Table S27. Geometric parameters (Å, º) for (**26**).

C101—C114	1.500 (10)	C304—C317	1.462 (8)
C101—C102	1.584 (9)	C305—C306	1.400 (8)
C101—H10A	0.9900	C305—H305	0.9500
C101—H10B	0.9900	C306—C307	1.383 (9)
C102—C103	1.521 (9)	C306—C309	1.497 (9)
C102—H10C	0.9900	C307—C308	1.388 (10)
C102—H10D	0.9900	C307—H307	0.9500
C103—C108	1.382 (10)	C308—H308	0.9500

C103—C104	1.425 (9)	C309—C310	1.595 (10)
C104—C105	1.394 (9)	C309—H30E	0.9900
C104—C117	1.472 (9)	C309—H30F	0.9900
C105—C106	1.383 (9)	C310—C311	1.517 (9)
C105—H105	0.9500	C310—H31A	0.9900
C106—C107	1.379 (10)	C310—H31B	0.9900
C106—C109	1.513 (9)	C311—C312	1.387 (10)
C107—C108	1.383 (10)	C311—C316	1.407 (9)
C107—H107	0.9500	C312—C313	1.385 (10)
C108—H108	0.9500	C312—H312	0.9500
C109—C110	1.581 (9)	C313—C314	1.400 (10)
C109—H10E	0.9900	C313—H313	0.9500
C109—H10F	0.9900	C314—C315	1.369 (9)
C110—C111	1.527 (9)	C315—C316	1.412 (9)
C110—H11A	0.9900	C315—H315	0.9500
C110—H11B	0.9900	C316—C328	1.465 (9)
C111—C112	1.372 (9)	C317—N318	1.279 (8)
C111—C116	1.408 (9)	C317—H317	0.9500
C112—C113	1.391 (10)	N318—N319	1.384 (6)
C112—H112	0.9500	N319—C320	1.354 (8)
C113—C114	1.402 (9)	N319—H319	0.8800
C113—H113	0.9500	C320—N321	1.342 (8)
C114—C115	1.398 (8)	C320—S320	1.685 (6)
C115—C116	1.395 (9)	N321—C322	1.468 (8)
C115—H115	0.9500	N321—H321	0.8800
C116—C128	1.454 (8)	C322—C323	1.528 (9)
C117—N118	1.287 (8)	C322—C327	1.530 (10)
C117—H117	0.9500	C322—H322	1.0000
N118—N119	1.378 (7)	C323—C324	1.511 (11)
N119—C120	1.358 (8)	C323—H32A	0.9900
N119—H119	0.8800	C323—H32B	0.9900
C120—N121	1.324 (8)	C324—C325	1.525 (13)
C120—S120	1.697 (7)	C324—H32C	0.9900
N121—C122	1.455 (8)	C324—H32D	0.9900
N121—H121	0.8800	C325—C326	1.539 (13)
C122—C127	1.518 (10)	C325—H32E	0.9900
C122—C123	1.524 (10)	C325—H32F	0.9900

C122—H122	1.0000	C326—C327	1.442 (13)
C123—C124	1.507 (11)	C326—H32G	0.9900
C123—H12A	0.9900	C326—H32H	0.9900
C123—H12B	0.9900	C327—H32I	0.9900
C124—C125	1.525 (13)	C327—H32J	0.9900
C124—H12C	0.9900	C328—N329	1.283 (8)
C124—H12D	0.9900	C328—H328	0.9500
C125—C126	1.509 (13)	N329—N330	1.381 (8)
C125—H12E	0.9900	N330—C331	1.355 (8)
C125—H12F	0.9900	N330—H330	0.8800
C126—C127	1.535 (12)	C331—N332	1.315 (8)
C126—H12G	0.9900	C331—S331	1.699 (7)
C126—H12H	0.9900	N332—C333	1.466 (8)
C127—H12I	0.9900	N332—H332	0.8800
C127—H12J	0.9900	C333—C334	1.500 (10)
C128—N129	1.289 (8)	C333—C338	1.511 (10)
C128—H128	0.9500	C333—H333	1.0000
N129—N130	1.363 (7)	C334—C335	1.518 (10)
N130—C131	1.351 (8)	C334—H33A	0.9900
N130—H130	0.8800	C334—H33B	0.9900
C131—N132	1.329 (8)	C335—C336	1.516 (12)
C131—S131	1.693 (6)	C335—H33C	0.9900
N132—C133	1.443 (9)	C335—H33D	0.9900
N132—H132	0.8800	C336—C337	1.520 (12)
C133—C138	1.454 (13)	C336—H33E	0.9900
C133—C134	1.537 (11)	C336—H33F	0.9900
C133—H133	1.0000	C337—C338	1.519 (10)
C134—C135	1.469 (14)	C337—H33G	0.9900
C134—H13A	0.9900	C337—H33H	0.9900
C134—H13B	0.9900	C338—H33I	0.9900
C135—C136	1.517 (16)	C338—H33J	0.9900
C135—H13C	0.9900	C401—C414	1.506 (11)
C135—H13D	0.9900	C401—C402	1.583 (11)
C136—C137	1.562 (17)	C401—H40A	0.9900
C136—H13E	0.9900	C401—H40B	0.9900
C136—H13F	0.9900	C402—C403	1.499 (10)
C137—C138	1.407 (17)	C402—H40C	0.9900

C137—H13G	0.9900	C402—H40D	0.9900
C137—H13H	0.9900	C403—C408	1.391 (10)
C138—H13I	0.9900	C403—C404	1.410 (9)
C138—H13J	0.9900	C404—C405	1.418 (9)
C201—C214	1.522 (11)	C404—C417	1.434 (9)
C201—C202	1.567 (11)	C405—C406	1.366 (9)
C201—H20A	0.9900	C405—H405	0.9500
C201—H20B	0.9900	C406—C407	1.402 (10)
C202—C203	1.506 (10)	C406—C409	1.508 (10)
C202—H20C	0.9900	C407—C408	1.388 (11)
C202—H20D	0.9900	C407—H407	0.9500
C203—C208	1.400 (10)	C408—H408	0.9500
C203—C204	1.409 (9)	C409—C410	1.571 (11)
C204—C205	1.393 (9)	C409—H40E	0.9900
C204—C217	1.459 (9)	C409—H40F	0.9900
C205—C206	1.390 (9)	C410—C411	1.515 (11)
C205—H205	0.9500	C410—H41A	0.9900
C206—C207	1.396 (9)	C410—H41B	0.9900
C206—C209	1.533 (9)	C411—C416	1.392 (9)
C207—C208	1.365 (10)	C411—C412	1.416 (10)
C207—H207	0.9500	C412—C413	1.364 (12)
C208—H208	0.9500	C412—H412	0.9500
C209—C210	1.569 (11)	C413—C414	1.393 (10)
C209—H20E	0.9900	C413—H413	0.9500
C209—H20F	0.9900	C414—C415	1.394 (8)
C210—C211	1.523 (10)	C415—C416	1.405 (10)
C210—H21A	0.9900	C415—H415	0.9500
C210—H21B	0.9900	C416—C428	1.469 (8)
C211—C212	1.376 (10)	C417—N418	1.283 (8)
C211—C216	1.401 (9)	C417—H417	0.9500
C212—C213	1.389 (11)	N418—N419	1.378 (7)
C212—H212	0.9500	N419—C420	1.346 (8)
C213—C214	1.385 (11)	N419—H419	0.8800
C213—H213	0.9500	C420—N421	1.339 (8)
C214—C215	1.384 (10)	C420—S420	1.679 (6)
C215—C216	1.395 (10)	N421—C422	1.458 (8)
C215—H215	0.9500	N421—H421	0.8800

C216—C228	1.451 (9)	C422—C423	1.525 (9)
C217—N218	1.281 (8)	C422—C427	1.532 (9)
C217—H217	0.9500	C422—H422	1.0000
N218—N219	1.378 (7)	C423—C424	1.520 (10)
N219—C220	1.359 (8)	C423—H42A	0.9900
N219—H219	0.8800	C423—H42B	0.9900
C220—N221	1.336 (8)	C424—C425	1.504 (11)
C220—S220	1.675 (6)	C424—H42C	0.9900
N221—C222	1.484 (9)	C424—H42D	0.9900
N221—H221	0.8800	C425—C426	1.510 (12)
C222—C227	1.508 (11)	C425—H42E	0.9900
C222—C223	1.542 (9)	C425—H42F	0.9900
C222—H222	1.0000	C426—C427	1.538 (11)
C223—C224	1.435 (14)	C426—H42G	0.9900
C223—H22A	0.9900	C426—H42H	0.9900
C223—H22B	0.9900	C427—H42I	0.9900
C224—C225	1.588 (17)	C427—H42J	0.9900
C224—H22C	0.9900	C428—N429	1.273 (8)
C224—H22D	0.9900	C428—H428	0.9500
C225—C226	1.560 (18)	N429—N430	1.390 (7)
C225—H22E	0.9900	N430—C431	1.353 (8)
C225—H22F	0.9900	N430—H430	0.8800
C226—C227	1.309 (18)	C431—N432	1.341 (8)
C226—H22G	0.9900	C431—S431	1.681 (6)
C226—H22H	0.9900	N432—C433	1.472 (8)
C227—H22I	0.9900	N432—H432	0.8800
C227—H22J	0.9900	C433—C438	1.519 (12)
C228—N229	1.290 (9)	C433—C434	1.535 (10)
C228—H228	0.9500	C433—H433	1.0000
N229—N230	1.380 (7)	C434—C435	1.490 (11)
N230—C231	1.349 (8)	C434—H43A	0.9900
N230—H230	0.8800	C434—H43B	0.9900
C231—N232	1.341 (9)	C435—C436	1.470 (16)
C231—S231	1.685 (7)	C435—H43C	0.9900
N232—C233	1.452 (8)	C435—H43D	0.9900
N232—H232	0.8800	C436—C437	1.460 (17)
C233—C234	1.526 (9)	C436—H43E	0.9900

C233—C238	1.528 (9)	C436—H43F	0.9900
C233—H233	1.0000	C437—C438	1.564 (15)
C234—C235	1.540 (9)	C437—H43G	0.9900
C234—H23A	0.9900	C437—H43H	0.9900
C234—H23B	0.9900	C438—H43I	0.9900
C235—C236	1.502 (11)	C438—H43J	0.9900
C235—H23C	0.9900	N1A—C1A	1.142 (4)
C235—H23D	0.9900	C1A—C2A	1.474 (4)
C236—C237	1.527 (11)	C2A—H2A1	0.9800
C236—H23E	0.9900	C2A—H2A2	0.9800
C236—H23F	0.9900	C2A—H2A3	0.9800
C237—C238	1.551 (11)	N1B—C1B	1.145 (4)
C237—H23G	0.9900	C1B—C2B	1.473 (4)
C237—H23H	0.9900	C2B—H2B1	0.9800
C238—H23I	0.9900	C2B—H2B2	0.9800
C238—H23J	0.9900	C2B—H2B3	0.9800
C301—C314	1.514 (10)	N1C—C1C	1.143 (4)
C301—C302	1.580 (10)	C1C—C2C	1.474 (4)
C301—H30A	0.9900	C2C—H2C1	0.9800
C301—H30B	0.9900	C2C—H2C2	0.9800
C302—C303	1.491 (10)	C2C—H2C3	0.9800
C302—H30C	0.9900	N1D—C1D	1.144 (4)
C302—H30D	0.9900	C1D—C2D	1.474 (4)
C303—C304	1.395 (8)	C2D—H2D1	0.9800
C303—C308	1.408 (9)	C2D—H2D2	0.9800
C304—C305	1.383 (9)	C2D—H2D3	0.9800
C114—C101—C102	111.7 (5)	C308—C303—C302	118.8 (6)
C114—C101—H10A	109.3	C305—C304—C303	120.2 (5)
C102—C101—H10A	109.3	C305—C304—C317	118.4 (5)
C114—C101—H10B	109.3	C303—C304—C317	120.7 (6)
C102—C101—H10B	109.3	C304—C305—C306	121.9 (5)
H10A—C101—H10B	107.9	C304—C305—H305	119.1
C103—C102—C101	114.0 (5)	C306—C305—H305	119.1
C103—C102—H10C	108.8	C307—C306—C305	116.3 (6)
C101—C102—H10C	108.8	C307—C306—C309	120.9 (5)
C103—C102—H10D	108.8	C305—C306—C309	121.2 (5)

C101—C102—H10D	108.8	C306—C307—C308	120.9 (6)
H10C—C102—H10D	107.7	C306—C307—H307	119.5
C108—C103—C104	117.5 (6)	C308—C307—H307	119.5
C108—C103—C102	118.8 (6)	C307—C308—C303	121.0 (6)
C104—C103—C102	122.1 (6)	C307—C308—H308	119.5
C105—C104—C103	118.3 (6)	C303—C308—H308	119.5
C105—C104—C117	119.3 (6)	C306—C309—C310	111.9 (5)
C103—C104—C117	120.8 (6)	C306—C309—H30E	109.2
C106—C105—C104	121.7 (6)	C310—C309—H30E	109.2
C106—C105—H105	119.1	C306—C309—H30F	109.2
C104—C105—H105	119.1	C310—C309—H30F	109.2
C107—C106—C105	117.8 (6)	H30E—C309—H30F	107.9
C107—C106—C109	122.3 (6)	C311—C310—C309	113.7 (5)
C105—C106—C109	118.6 (6)	C311—C310—H31A	108.8
C106—C107—C108	120.4 (6)	C309—C310—H31A	108.8
C106—C107—H107	119.8	C311—C310—H31B	108.8
C108—C107—H107	119.8	C309—C310—H31B	108.8
C107—C108—C103	121.4 (6)	H31A—C310—H31B	107.7
C107—C108—H108	119.3	C312—C311—C316	117.3 (6)
C103—C108—H108	119.3	C312—C311—C310	119.9 (6)
C106—C109—C110	111.8 (5)	C316—C311—C310	121.3 (6)
C106—C109—H10E	109.3	C313—C312—C311	122.0 (6)
C110—C109—H10E	109.3	C313—C312—H312	119.0
C106—C109—H10F	109.3	C311—C312—H312	119.0
C110—C109—H10F	109.3	C312—C313—C314	119.6 (6)
H10E—C109—H10F	107.9	C312—C313—H313	120.2
C111—C110—C109	112.2 (5)	C314—C313—H313	120.2
C111—C110—H11A	109.2	C315—C314—C313	117.7 (6)
C109—C110—H11A	109.2	C315—C314—C301	118.9 (6)
C111—C110—H11B	109.2	C313—C314—C301	122.0 (7)
C109—C110—H11B	109.2	C314—C315—C316	121.8 (6)
H11A—C110—H11B	107.9	C314—C315—H315	119.1
C112—C111—C116	118.2 (6)	C316—C315—H315	119.1
C112—C111—C110	118.7 (6)	C311—C316—C315	118.7 (6)
C116—C111—C110	122.2 (6)	C311—C316—C328	121.6 (6)
C111—C112—C113	121.0 (6)	C315—C316—C328	117.6 (6)
C111—C112—H112	119.5	N318—C317—C304	119.8 (6)

C113—C112—H112	119.5	N318—C317—H317	120.1
C112—C113—C114	120.5 (6)	C304—C317—H317	120.1
C112—C113—H113	119.8	C317—N318—N319	117.8 (5)
C114—C113—H113	119.8	C320—N319—N318	118.8 (5)
C115—C114—C113	116.6 (6)	C320—N319—H319	120.6
C115—C114—C101	120.2 (6)	N318—N319—H319	120.6
C113—C114—C101	121.7 (6)	N321—C320—N319	115.9 (5)
C116—C115—C114	121.3 (6)	N321—C320—S320	124.5 (5)
C116—C115—H115	119.4	N319—C320—S320	119.7 (5)
C114—C115—H115	119.4	C320—N321—C322	126.2 (5)
C115—C116—C111	119.2 (6)	C320—N321—H321	116.9
C115—C116—C128	117.8 (6)	C322—N321—H321	116.9
C111—C116—C128	122.4 (5)	N321—C322—C323	109.0 (5)
N118—C117—C104	121.4 (6)	N321—C322—C327	111.4 (6)
N118—C117—H117	119.3	C323—C322—C327	110.6 (6)
C104—C117—H117	119.3	N321—C322—H322	108.6
C117—N118—N119	114.2 (5)	C323—C322—H322	108.6
C120—N119—N118	120.4 (5)	C327—C322—H322	108.6
C120—N119—H119	119.8	C324—C323—C322	112.3 (7)
N118—N119—H119	119.8	C324—C323—H32A	109.1
N121—C120—N119	117.1 (6)	C322—C323—H32A	109.1
N121—C120—S120	124.2 (5)	C324—C323—H32B	109.1
N119—C120—S120	118.7 (5)	C322—C323—H32B	109.1
C120—N121—C122	123.4 (6)	H32A—C323—H32B	107.9
C120—N121—H121	118.3	C323—C324—C325	111.9 (7)
C122—N121—H121	118.3	C323—C324—H32C	109.2
N121—C122—C127	109.5 (6)	C325—C324—H32C	109.2
N121—C122—C123	110.9 (5)	C323—C324—H32D	109.2
C127—C122—C123	109.8 (6)	C325—C324—H32D	109.2
N121—C122—H122	108.9	H32C—C324—H32D	107.9
C127—C122—H122	108.9	C324—C325—C326	111.3 (8)
C123—C122—H122	108.9	C324—C325—H32E	109.4
C124—C123—C122	110.3 (6)	C326—C325—H32E	109.4
C124—C123—H12A	109.6	C324—C325—H32F	109.4
C122—C123—H12A	109.6	C326—C325—H32F	109.4
C124—C123—H12B	109.6	H32E—C325—H32F	108.0
C122—C123—H12B	109.6	C327—C326—C325	112.2 (8)

H12A—C123—H12B	108.1	C327—C326—H32G	109.2
C123—C124—C125	110.5 (7)	C325—C326—H32G	109.2
C123—C124—H12C	109.6	C327—C326—H32H	109.2
C125—C124—H12C	109.6	C325—C326—H32H	109.2
C123—C124—H12D	109.6	H32G—C326—H32H	107.9
C125—C124—H12D	109.6	C326—C327—C322	113.1 (7)
H12C—C124—H12D	108.1	C326—C327—H32I	109.0
C126—C125—C124	110.9 (7)	C322—C327—H32I	109.0
C126—C125—H12E	109.5	C326—C327—H32J	109.0
C124—C125—H12E	109.5	C322—C327—H32J	109.0
C126—C125—H12F	109.5	H32I—C327—H32J	107.8
C124—C125—H12F	109.5	N329—C328—C316	123.1 (6)
H12E—C125—H12F	108.1	N329—C328—H328	118.5
C125—C126—C127	112.4 (7)	C316—C328—H328	118.5
C125—C126—H12G	109.1	C328—N329—N330	115.2 (5)
C127—C126—H12G	109.1	C331—N330—N329	120.6 (5)
C125—C126—H12H	109.1	C331—N330—H330	119.7
C127—C126—H12H	109.1	N329—N330—H330	119.7
H12G—C126—H12H	107.9	N332—C331—N330	117.2 (6)
C122—C127—C126	107.4 (8)	N332—C331—S331	124.4 (5)
C122—C127—H12I	110.2	N330—C331—S331	118.5 (5)
C126—C127—H12I	110.2	C331—N332—C333	124.5 (6)
C122—C127—H12J	110.2	C331—N332—H332	117.8
C126—C127—H12J	110.2	C333—N332—H332	117.8
H12I—C127—H12J	108.5	N332—C333—C334	111.2 (5)
N129—C128—C116	119.7 (5)	N332—C333—C338	110.2 (6)
N129—C128—H128	120.2	C334—C333—C338	110.6 (6)
C116—C128—H128	120.2	N332—C333—H333	108.3
C128—N129—N130	117.6 (5)	C334—C333—H333	108.3
C131—N130—N129	118.6 (5)	C338—C333—H333	108.3
C131—N130—H130	120.7	C333—C334—C335	109.6 (6)
N129—N130—H130	120.7	C333—C334—H33A	109.7
N132—C131—N130	115.4 (5)	C335—C334—H33A	109.7
N132—C131—S131	124.7 (5)	C333—C334—H33B	109.7
N130—C131—S131	119.9 (4)	C335—C334—H33B	109.7
C131—N132—C133	128.0 (6)	H33A—C334—H33B	108.2
C131—N132—H132	116.0	C336—C335—C334	111.9 (7)

C133—N132—H132	116.0	C336—C335—H33C	109.2
N132—C133—C138	109.5 (7)	C334—C335—H33C	109.2
N132—C133—C134	110.2 (7)	C336—C335—H33D	109.2
C138—C133—C134	111.4 (7)	C334—C335—H33D	109.2
N132—C133—H133	108.6	H33C—C335—H33D	107.9
C138—C133—H133	108.6	C335—C336—C337	112.0 (7)
C134—C133—H133	108.6	C335—C336—H33E	109.2
C135—C134—C133	111.8 (8)	C337—C336—H33E	109.2
C135—C134—H13A	109.3	C335—C336—H33F	109.2
C133—C134—H13A	109.3	C337—C336—H33F	109.2
C135—C134—H13B	109.3	H33E—C336—H33F	107.9
C133—C134—H13B	109.3	C338—C337—C336	111.7 (7)
H13A—C134—H13B	107.9	C338—C337—H33G	109.3
C134—C135—C136	112.5 (9)	C336—C337—H33G	109.3
C134—C135—H13C	109.1	C338—C337—H33H	109.3
C136—C135—H13C	109.1	C336—C337—H33H	109.3
C134—C135—H13D	109.1	H33G—C337—H33H	107.9
C136—C135—H13D	109.1	C333—C338—C337	109.8 (6)
H13C—C135—H13D	107.8	C333—C338—H33I	109.7
C135—C136—C137	112.2 (8)	C337—C338—H33I	109.7
C135—C136—H13E	109.2	C333—C338—H33J	109.7
C137—C136—H13E	109.2	C337—C338—H33J	109.7
C135—C136—H13F	109.2	H33I—C338—H33J	108.2
C137—C136—H13F	109.2	C414—C401—C402	113.2 (6)
H13E—C136—H13F	107.9	C414—C401—H40A	108.9
C138—C137—C136	113.0 (10)	C402—C401—H40A	108.9
C138—C137—H13G	109.0	C414—C401—H40B	108.9
C136—C137—H13G	109.0	C402—C401—H40B	108.9
C138—C137—H13H	109.0	H40A—C401—H40B	107.8
C136—C137—H13H	109.0	C403—C402—C401	113.0 (6)
H13G—C137—H13H	107.8	C403—C402—H40C	109.0
C137—C138—C133	115.2 (10)	C401—C402—H40C	109.0
C137—C138—H13I	108.5	C403—C402—H40D	109.0
C133—C138—H13I	108.5	C401—C402—H40D	109.0
C137—C138—H13J	108.5	H40C—C402—H40D	107.8
C133—C138—H13J	108.5	C408—C403—C404	116.3 (6)
H13I—C138—H13J	107.5	C408—C403—C402	118.3 (6)

C214—C201—C202	111.9 (6)	C404—C403—C402	124.4 (6)
C214—C201—H20A	109.2	C403—C404—C405	119.5 (6)
C202—C201—H20A	109.2	C403—C404—C417	120.8 (6)
C214—C201—H20B	109.2	C405—C404—C417	119.7 (6)
C202—C201—H20B	109.2	C406—C405—C404	121.5 (6)
H20A—C201—H20B	107.9	C406—C405—H405	119.3
C203—C202—C201	113.0 (6)	C404—C405—H405	119.3
C203—C202—H20C	109.0	C405—C406—C407	117.6 (7)
C201—C202—H20C	109.0	C405—C406—C409	121.3 (7)
C203—C202—H20D	109.0	C407—C406—C409	119.8 (7)
C201—C202—H20D	109.0	C408—C407—C406	120.2 (6)
H20C—C202—H20D	107.8	C408—C407—H407	119.9
C208—C203—C204	116.6 (6)	C406—C407—H407	119.9
C208—C203—C202	119.4 (6)	C407—C408—C403	121.7 (7)
C204—C203—C202	122.9 (6)	C407—C408—H408	119.1
C205—C204—C203	119.4 (6)	C403—C408—H408	119.1
C205—C204—C217	118.4 (6)	C406—C409—C410	111.8 (6)
C203—C204—C217	121.9 (6)	C406—C409—H40E	109.3
C206—C205—C204	121.5 (6)	C410—C409—H40E	109.3
C206—C205—H205	119.2	C406—C409—H40F	109.3
C204—C205—H205	119.2	C410—C409—H40F	109.3
C205—C206—C207	116.6 (6)	H40E—C409—H40F	107.9
C205—C206—C209	120.3 (6)	C411—C410—C409	112.5 (6)
C207—C206—C209	121.6 (6)	C411—C410—H41A	109.1
C208—C207—C206	120.9 (6)	C409—C410—H41A	109.1
C208—C207—H207	119.6	C411—C410—H41B	109.1
C206—C207—H207	119.6	C409—C410—H41B	109.1
C207—C208—C203	121.5 (6)	H41A—C410—H41B	107.8
C207—C208—H208	119.3	C416—C411—C412	116.2 (7)
C203—C208—H208	119.3	C416—C411—C410	123.1 (6)
C206—C209—C210	112.1 (6)	C412—C411—C410	119.4 (6)
C206—C209—H20E	109.2	C413—C412—C411	121.5 (7)
C210—C209—H20E	109.2	C413—C412—H412	119.3
C206—C209—H20F	109.2	C411—C412—H412	119.3
C210—C209—H20F	109.2	C412—C413—C414	121.2 (6)
H20E—C209—H20F	107.9	C412—C413—H413	119.4
C211—C210—C209	113.7 (6)	C414—C413—H413	119.4

C211—C210—H21A	108.8	C413—C414—C415	117.0 (7)
C209—C210—H21A	108.8	C413—C414—C401	123.2 (6)
C211—C210—H21B	108.8	C415—C414—C401	118.0 (6)
C209—C210—H21B	108.8	C414—C415—C416	120.6 (6)
H21A—C210—H21B	107.7	C414—C415—H415	119.7
C212—C211—C216	117.5 (7)	C416—C415—H415	119.7
C212—C211—C210	119.4 (6)	C411—C416—C415	120.7 (6)
C216—C211—C210	121.7 (6)	C411—C416—C428	121.4 (6)
C211—C212—C213	121.3 (7)	C415—C416—C428	117.6 (6)
C211—C212—H212	119.3	N418—C417—C404	120.9 (6)
C213—C212—H212	119.3	N418—C417—H417	119.5
C214—C213—C212	119.4 (7)	C404—C417—H417	119.5
C214—C213—H213	120.3	C417—N418—N419	114.2 (6)
C212—C213—H213	120.3	C420—N419—N418	121.1 (6)
C215—C214—C213	118.9 (7)	C420—N419—H419	119.4
C215—C214—C201	118.4 (7)	N418—N419—H419	119.4
C213—C214—C201	121.2 (7)	N421—C420—N419	117.7 (6)
C214—C215—C216	120.0 (7)	N421—C420—S420	123.6 (5)
C214—C215—H215	120.0	N419—C420—S420	118.7 (5)
C216—C215—H215	120.0	C420—N421—C422	124.4 (5)
C215—C216—C211	119.9 (6)	C420—N421—H421	117.8
C215—C216—C228	119.4 (6)	C422—N421—H421	117.8
C211—C216—C228	120.6 (6)	N421—C422—C423	110.3 (5)
N218—C217—C204	118.5 (5)	N421—C422—C427	111.9 (6)
N218—C217—H217	120.8	C423—C422—C427	110.4 (5)
C204—C217—H217	120.8	N421—C422—H422	108.0
C217—N218—N219	117.7 (5)	C423—C422—H422	108.0
C220—N219—N218	118.3 (5)	C427—C422—H422	108.0
C220—N219—H219	120.8	C424—C423—C422	110.8 (6)
N218—N219—H219	120.8	C424—C423—H42A	109.5
N221—C220—N219	115.0 (6)	C422—C423—H42A	109.5
N221—C220—S220	124.7 (5)	C424—C423—H42B	109.5
N219—C220—S220	120.3 (4)	C422—C423—H42B	109.5
C220—N221—C222	123.7 (6)	H42A—C423—H42B	108.1
C220—N221—H221	118.1	C425—C424—C423	112.0 (6)
C222—N221—H221	118.1	C425—C424—H42C	109.2
N221—C222—C227	112.0 (7)	C423—C424—H42C	109.2

N221—C222—C223	107.8 (6)	C425—C424—H42D	109.2
C227—C222—C223	109.6 (6)	C423—C424—H42D	109.2
N221—C222—H222	109.1	H42C—C424—H42D	107.9
C227—C222—H222	109.1	C424—C425—C426	112.2 (6)
C223—C222—H222	109.1	C424—C425—H42E	109.2
C224—C223—C222	114.1 (8)	C426—C425—H42E	109.2
C224—C223—H22A	108.7	C424—C425—H42F	109.2
C222—C223—H22A	108.7	C426—C425—H42F	109.2
C224—C223—H22B	108.7	H42E—C425—H42F	107.9
C222—C223—H22B	108.7	C425—C426—C427	109.7 (7)
H22A—C223—H22B	107.6	C425—C426—H42G	109.7
C223—C224—C225	107.7 (10)	C427—C426—H42G	109.7
C223—C224—H22C	110.2	C425—C426—H42H	109.7
C225—C224—H22C	110.2	C427—C426—H42H	109.7
C223—C224—H22D	110.2	H42G—C426—H42H	108.2
C225—C224—H22D	110.2	C422—C427—C426	109.5 (6)
H22C—C224—H22D	108.5	C422—C427—H42I	109.8
C226—C225—C224	106.6 (10)	C426—C427—H42I	109.8
C226—C225—H22E	110.4	C422—C427—H42J	109.8
C224—C225—H22E	110.4	C426—C427—H42J	109.8
C226—C225—H22F	110.4	H42I—C427—H42J	108.2
C224—C225—H22F	110.4	N429—C428—C416	119.2 (6)
H22E—C225—H22F	108.6	N429—C428—H428	120.4
C227—C226—C225	112.2 (13)	C416—C428—H428	120.4
C227—C226—H22G	109.2	C428—N429—N430	116.8 (5)
C225—C226—H22G	109.2	C431—N430—N429	118.3 (5)
C227—C226—H22H	109.2	C431—N430—H430	120.9
C225—C226—H22H	109.2	N429—N430—H430	120.9
H22G—C226—H22H	107.9	N432—C431—N430	114.7 (5)
C226—C227—C222	116.2 (12)	N432—C431—S431	125.0 (5)
C226—C227—H22I	108.2	N430—C431—S431	120.3 (5)
C222—C227—H22I	108.2	C431—N432—C433	125.7 (5)
C226—C227—H22J	108.2	C431—N432—H432	117.1
C222—C227—H22J	108.2	C433—N432—H432	117.1
H22I—C227—H22J	107.4	N432—C433—C438	110.2 (6)
N229—C228—C216	121.8 (6)	N432—C433—C434	107.3 (6)
N229—C228—H228	119.1	C438—C433—C434	110.9 (7)

C216—C228—H228	119.1	N432—C433—H433	109.5
C228—N229—N230	114.3 (6)	C438—C433—H433	109.5
C231—N230—N229	121.9 (6)	C434—C433—H433	109.5
C231—N230—H230	119.1	C435—C434—C433	114.6 (7)
N229—N230—H230	119.1	C435—C434—H43A	108.6
N232—C231—N230	117.2 (6)	C433—C434—H43A	108.6
N232—C231—S231	124.0 (5)	C435—C434—H43B	108.6
N230—C231—S231	118.8 (5)	C433—C434—H43B	108.6
C231—N232—C233	124.3 (5)	H43A—C434—H43B	107.6
C231—N232—H232	117.9	C436—C435—C434	111.1 (9)
C233—N232—H232	117.9	C436—C435—H43C	109.4
N232—C233—C234	109.7 (5)	C434—C435—H43C	109.4
N232—C233—C238	111.4 (5)	C436—C435—H43D	109.4
C234—C233—C238	110.5 (5)	C434—C435—H43D	109.4
N232—C233—H233	108.4	H43C—C435—H43D	108.0
C234—C233—H233	108.4	C437—C436—C435	113.8 (9)
C238—C233—H233	108.4	C437—C436—H43E	108.8
C233—C234—C235	110.4 (6)	C435—C436—H43E	108.8
C233—C234—H23A	109.6	C437—C436—H43F	108.8
C235—C234—H23A	109.6	C435—C436—H43F	108.8
C233—C234—H23B	109.6	H43E—C436—H43F	107.7
C235—C234—H23B	109.6	C436—C437—C438	112.7 (10)
H23A—C234—H23B	108.1	C436—C437—H43G	109.0
C236—C235—C234	111.5 (6)	C438—C437—H43G	109.0
C236—C235—H23C	109.3	C436—C437—H43H	109.0
C234—C235—H23C	109.3	C438—C437—H43H	109.0
C236—C235—H23D	109.3	H43G—C437—H43H	107.8
C234—C235—H23D	109.3	C433—C438—C437	109.9 (8)
H23C—C235—H23D	108.0	C433—C438—H43I	109.7
C235—C236—C237	112.3 (6)	C437—C438—H43I	109.7
C235—C236—H23E	109.1	C433—C438—H43J	109.7
C237—C236—H23E	109.1	C437—C438—H43J	109.7
C235—C236—H23F	109.1	H43I—C438—H43J	108.2
C237—C236—H23F	109.1	N1A—C1A—C2A	164.2 (15)
H23E—C236—H23F	107.9	C1A—C2A—H2A1	109.5
C236—C237—C238	109.0 (7)	C1A—C2A—H2A2	109.5
C236—C237—H23G	109.9	H2A1—C2A—H2A2	109.5

C238—C237—H23G	109.9	C1A—C2A—H2A3	109.5
C236—C237—H23H	109.9	H2A1—C2A—H2A3	109.5
C238—C237—H23H	109.9	H2A2—C2A—H2A3	109.5
H23G—C237—H23H	108.3	N1B—C1B—C2B	166 (3)
C233—C238—C237	109.5 (6)	C1B—C2B—H2B1	109.5
C233—C238—H23I	109.8	C1B—C2B—H2B2	109.5
C237—C238—H23I	109.8	H2B1—C2B—H2B2	109.5
C233—C238—H23J	109.8	C1B—C2B—H2B3	109.5
C237—C238—H23J	109.8	H2B1—C2B—H2B3	109.5
H23I—C238—H23J	108.2	H2B2—C2B—H2B3	109.5
C314—C301—C302	110.9 (6)	N1C—C1C—C2C	167.7 (18)
C314—C301—H30A	109.5	C1C—C2C—H2C1	109.5
C302—C301—H30A	109.5	C1C—C2C—H2C2	109.5
C314—C301—H30B	109.5	H2C1—C2C—H2C2	109.5
C302—C301—H30B	109.5	C1C—C2C—H2C3	109.5
H30A—C301—H30B	108.1	H2C1—C2C—H2C3	109.5
C303—C302—C301	114.5 (5)	H2C2—C2C—H2C3	109.5
C303—C302—H30C	108.6	N1D—C1D—C2D	164 (3)
C301—C302—H30C	108.6	C1D—C2D—H2D1	109.5
C303—C302—H30D	108.6	C1D—C2D—H2D2	109.5
C301—C302—H30D	108.6	H2D1—C2D—H2D2	109.5
H30C—C302—H30D	107.6	C1D—C2D—H2D3	109.5
C304—C303—C308	116.3 (6)	H2D1—C2D—H2D3	109.5
C304—C303—C302	123.9 (6)	H2D2—C2D—H2D3	109.5
C114—C101—C102—C103	13.7 (8)	C314—C301—C302—C303	12.6 (8)
C101—C102—C103—C108	-94.5 (7)	C301—C302—C303—C304	75.7 (8)
C101—C102—C103—C104	70.7 (8)	C301—C302—C303—C308	-92.5 (7)
C108—C103—C104—C105	11.5 (8)	C308—C303—C304—C305	14.8 (8)
C102—C103—C104—C105	-154.0 (6)	C302—C303—C304—C305	-153.8 (6)
C108—C103—C104—C117	177.1 (6)	C308—C303—C304—C317	-175.3 (5)
C102—C103—C104—C117	11.6 (9)	C302—C303—C304—C317	16.2 (9)
C103—C104—C105—C106	3.3 (8)	C303—C304—C305—C306	0.2 (9)
C117—C104—C105—C106	-162.6 (5)	C317—C304—C305—C306	-169.9 (5)
C104—C105—C106—C107	-15.7 (8)	C304—C305—C306—C307	-14.8 (8)
C104—C105—C106—C109	151.3 (6)	C304—C305—C306—C309	150.9 (6)
C105—C106—C107—C108	13.3 (9)	C305—C306—C307—C308	14.1 (9)

C109—C106—C107—C108	-153.2 (6)	C309—C306—C307—C308	-151.7 (6)
C106—C107—C108—C103	1.5 (10)	C306—C307—C308—C303	0.9 (10)
C104—C103—C108—C107	-14.0 (9)	C304—C303—C308—C307	-15.5 (9)
C102—C103—C108—C107	152.0 (6)	C302—C303—C308—C307	153.7 (6)
C107—C106—C109—C110	72.8 (7)	C307—C306—C309—C310	70.6 (7)
C105—C106—C109—C110	-93.5 (7)	C305—C306—C309—C310	-94.5 (6)
C106—C109—C110—C111	15.1 (8)	C306—C309—C310—C311	15.7 (7)
C109—C110—C111—C112	-97.1 (7)	C309—C310—C311—C312	-98.3 (7)
C109—C110—C111—C116	71.7 (7)	C309—C310—C311—C316	67.0 (8)
C116—C111—C112—C113	-14.7 (8)	C316—C311—C312—C313	-13.8 (10)
C110—C111—C112—C113	154.5 (6)	C310—C311—C312—C313	152.0 (6)
C111—C112—C113—C114	1.1 (9)	C311—C312—C313—C314	0.8 (10)
C112—C113—C114—C115	14.1 (8)	C312—C313—C314—C315	13.5 (10)
C112—C113—C114—C101	-151.9 (6)	C312—C313—C314—C301	-152.8 (7)
C102—C101—C114—C115	-91.5 (7)	C302—C301—C314—C315	-88.0 (8)
C102—C101—C114—C113	74.1 (7)	C302—C301—C314—C313	78.1 (8)
C113—C114—C115—C116	-16.0 (8)	C313—C314—C315—C316	-14.7 (9)
C101—C114—C115—C116	150.2 (6)	C301—C314—C315—C316	152.0 (6)
C114—C115—C116—C111	2.9 (9)	C312—C311—C316—C315	12.5 (9)
C114—C115—C116—C128	-168.2 (5)	C310—C311—C316—C315	-153.1 (6)
C112—C111—C116—C115	12.7 (8)	C312—C311—C316—C328	175.8 (6)
C110—C111—C116—C115	-156.1 (5)	C310—C311—C316—C328	10.2 (9)
C112—C111—C116—C128	-176.7 (5)	C314—C315—C316—C311	1.6 (9)
C110—C111—C116—C128	14.6 (8)	C314—C315—C316—C328	-162.3 (6)
C105—C104—C117—N118	-14.9 (9)	C305—C304—C317—N318	-27.3 (8)
C103—C104—C117—N118	179.6 (6)	C303—C304—C317—N318	162.6 (6)
C104—C117—N118—N119	170.3 (5)	C304—C317—N318—N319	179.1 (5)
C117—N118—N119—C120	-178.4 (6)	C317—N318—N319—C320	171.3 (6)
N118—N119—C120—N121	-11.5 (9)	N318—N319—C320—N321	-0.5 (8)
N118—N119—C120—S120	168.6 (4)	N318—N319—C320—S320	-179.4 (4)
N119—C120—N121—C122	-177.3 (6)	N319—C320—N321—C322	176.6 (6)
S120—C120—N121—C122	2.7 (9)	S320—C320—N321—C322	-4.5 (9)
C120—N121—C122—C127	-158.2 (6)	C320—N321—C322—C323	-157.9 (6)
C120—N121—C122—C123	80.5 (8)	C320—N321—C322—C327	79.8 (8)

N121—C122—C123—C124	-176.7 (6)	N321—C322—C323—C324	-70.6 (8)
C127—C122—C123—C124	62.2 (8)	C327—C322—C323—C324	52.3 (9)
C122—C123—C124—C125	-57.6 (9)	C322—C323—C324—C325	-52.2 (9)
C123—C124—C125—C126	54.1 (10)	C323—C324—C325—C326	51.8 (11)
C124—C125—C126—C127	-55.4 (11)	C324—C325—C326—C327	-54.0 (11)
N121—C122—C127—C126	177.4 (6)	C325—C326—C327—C322	55.8 (10)
C123—C122—C127—C126	-60.7 (8)	N321—C322—C327—C326	66.6 (9)
C125—C126—C127—C122	58.3 (10)	C323—C322—C327—C326	-54.8 (9)
C115—C116—C128—N129	-24.7 (8)	C311—C316—C328—N329	-176.9 (6)
C111—C116—C128—N129	164.5 (6)	C315—C316—C328—N329	-13.5 (9)
C116—C128—N129—N130	178.8 (5)	C316—C328—N329—N330	169.2 (6)
C128—N129—N130—C131	171.0 (6)	C328—N329—N330—C331	-178.0 (6)
N129—N130—C131—N132	1.6 (9)	N329—N330—C331—N332	-14.3 (9)
N129—N130—C131—S131	-178.6 (4)	N329—N330—C331—S331	165.6 (5)
N130—C131—N132—C133	-179.8 (7)	N330—C331—N332—C333	-179.2 (6)
S131—C131—N132—C133	0.4 (11)	S331—C331—N332—C333	0.9 (9)
C131—N132—C133—C138	86.9 (10)	C331—N332—C333—C334	78.0 (8)
C131—N132—C133—C134	-150.2 (7)	C331—N332—C333—C338	-159.1 (6)
N132—C133—C134—C135	-67.6 (9)	N332—C333—C334—C335	-176.6 (6)
C138—C133—C134—C135	54.1 (11)	C338—C333—C334—C335	60.6 (8)
C133—C134—C135—C136	-51.5 (11)	C333—C334—C335—C336	-56.2 (9)
C134—C135—C136—C137	47.5 (12)	C334—C335—C336—C337	51.9 (9)
C135—C136—C137—C138	-46.7 (13)	C335—C336—C337—C338	-51.5 (10)
C136—C137—C138—C133	51.4 (12)	N332—C333—C338—C337	176.2 (6)
N132—C133—C138—C137	67.2 (10)	C334—C333—C338—C337	-60.4 (8)
C134—C133—C138—C137	-55.0 (11)	C336—C337—C338—C333	55.4 (9)
C214—C201—C202—C203	-14.1 (10)	C414—C401—C402—C403	-8.8 (8)
C201—C202—C203—C208	94.2 (8)	C401—C402—C403—C408	86.5 (8)
C201—C202—C203—C204	-73.4 (9)	C401—C402—C403—C404	-81.2 (8)
C208—C203—C204—C205	-14.6 (9)	C408—C403—C404—C405	-15.5 (9)
C202—C203—C204—C205	153.3 (6)	C402—C403—C404—C405	152.4 (6)
C208—C203—C204—C217	171.3 (6)	C408—C403—C404—C417	165.5 (6)
C202—C203—C204—C217	-20.8 (10)	C402—C403—C404—C417	-26.6 (9)
C203—C204—C205—C206	0.0 (9)	C403—C404—C405—C406	1.2 (9)

C217—C204—C205—C206	174.2 (6)	C417—C404—C405—C406	-179.7 (6)
C204—C205—C206—C207	14.8 (9)	C404—C405—C406—C407	12.7 (9)
C204—C205—C206—C209	-151.5 (6)	C404—C405—C406—C409	-153.9 (6)
C205—C206—C207—C208	-14.8 (9)	C405—C406—C407—C408	-12.1 (10)
C209—C206—C207—C208	151.3 (6)	C409—C406—C407—C408	154.7 (7)
C206—C207—C208—C203	0.1 (10)	C406—C407—C408—C403	-2.6 (10)
C204—C203—C208—C207	14.8 (9)	C404—C403—C408—C407	16.3 (10)
C202—C203—C208—C207	-153.6 (7)	C402—C403—C408—C407	-152.4 (7)
C205—C206—C209—C210	92.1 (7)	C405—C406—C409—C410	96.2 (8)
C207—C206—C209—C210	-73.5 (8)	C407—C406—C409—C410	-70.1 (8)
C206—C209—C210—C211	-12.9 (8)	C406—C409—C410—C411	-13.6 (9)
C209—C210—C211—C212	93.8 (8)	C409—C410—C411—C416	-69.1 (8)
C209—C210—C211—C216	-72.7 (8)	C409—C410—C411—C412	97.7 (7)
C216—C211—C212—C213	14.5 (10)	C416—C411—C412—C413	13.2 (10)
C210—C211—C212—C213	-152.6 (7)	C410—C411—C412—C413	-154.4 (7)
C211—C212—C213—C214	-0.7 (11)	C411—C412—C413—C414	0.6 (11)
C212—C213—C214—C215	-12.9 (10)	C412—C413—C414—C415	-14.3 (10)
C212—C213—C214—C201	153.4 (7)	C412—C413—C414—C401	150.5 (7)
C202—C201—C214—C215	92.3 (8)	C402—C401—C414—C413	-80.2 (9)
C202—C201—C214—C213	-74.1 (9)	C402—C401—C414—C415	84.4 (8)
C213—C214—C215—C216	12.4 (10)	C413—C414—C415—C416	14.0 (9)
C201—C214—C215—C216	-154.3 (7)	C401—C414—C415—C416	-151.6 (7)
C214—C215—C216—C211	1.5 (10)	C412—C411—C416—C415	-13.2 (9)
C214—C215—C216—C228	-179.5 (6)	C410—C411—C416—C415	153.9 (6)
C212—C211—C216—C215	-14.8 (9)	C412—C411—C416—C428	173.4 (5)
C210—C211—C216—C215	151.9 (6)	C410—C411—C416—C428	-19.5 (9)
C212—C211—C216—C228	166.2 (6)	C414—C415—C416—C411	-0.2 (9)
C210—C211—C216—C228	-27.0 (10)	C414—C415—C416—C428	173.4 (6)
C205—C204—C217—N218	31.7 (9)	C403—C404—C417—N418	174.0 (6)
C203—C204—C217—N218	-154.1 (6)	C405—C404—C417—N418	-5.0 (10)
C204—C217—N218—N219	179.9 (5)	C404—C417—N418—N419	168.0 (6)
C217—N218—N219—C220	-161.0 (6)	C417—N418—N419—C420	179.6 (6)
N218—N219—C220—N221	-3.2 (9)	N418—N419—C420—N421	-14.0 (9)
N218—N219—C220—S220	177.0 (5)	N418—N419—C420—S420	166.5 (5)
N219—C220—N221—	176.9 (6)	N419—C420—N421—	179.6 (6)

C222		C422	
S220—C220—N221—C222	-3.2 (10)	S420—C420—N421—C422	-0.9 (9)
C220—N221—C222—C227	-79.7 (9)	C420—N421—C422—C423	-154.4 (6)
C220—N221—C222—C223	159.7 (7)	C420—N421—C422—C427	82.2 (8)
N221—C222—C223—C224	72.3 (9)	N421—C422—C423—C424	179.6 (6)
C227—C222—C223—C224	-49.8 (11)	C427—C422—C423—C424	-56.2 (8)
C222—C223—C224—C225	56.5 (11)	C422—C423—C424—C425	53.7 (8)
C223—C224—C225—C226	-58.5 (16)	C423—C424—C425—C426	-54.7 (9)
C224—C225—C226—C227	61 (2)	C424—C425—C426—C427	56.9 (9)
C225—C226—C227—C222	-58 (2)	N421—C422—C427—C426	-177.7 (6)
N221—C222—C227—C226	-69.4 (14)	C423—C422—C427—C426	59.0 (8)
C223—C222—C227—C226	50.2 (15)	C425—C426—C427—C422	-58.7 (8)
C215—C216—C228—N229	-7.8 (10)	C411—C416—C428—N429	-155.6 (6)
C211—C216—C228—N229	171.1 (6)	C415—C416—C428—N429	30.8 (8)
C216—C228—N229—N230	169.2 (6)	C416—C428—N429—N430	179.2 (5)
C228—N229—N230—C231	-177.2 (6)	C428—N429—N430—C431	-160.5 (6)
N229—N230—C231—N232	-14.3 (9)	N429—N430—C431—N432	-5.9 (9)
N229—N230—C231—S231	166.2 (5)	N429—N430—C431—S431	174.3 (5)
N230—C231—N232—C233	-178.2 (6)	N430—C431—N432—C433	168.3 (7)
S231—C231—N232—C233	1.2 (9)	S431—C431—N432—C433	-11.9 (10)
C231—N232—C233—C234	-154.9 (6)	C431—N432—C433—C438	-86.2 (9)
C231—N232—C233—C238	82.4 (7)	C431—N432—C433—C434	152.9 (7)
N232—C233—C234—C235	179.6 (5)	N432—C433—C434—C435	68.6 (9)
C238—C233—C234—C235	-57.2 (7)	C438—C433—C434—C435	-51.9 (10)
C233—C234—C235—C236	54.5 (7)	C433—C434—C435—C436	52.1 (10)
C234—C235—C236—C237	-55.5 (9)	C434—C435—C436—C437	-53.8 (11)
C235—C236—C237—C238	57.3 (9)	C435—C436—C437—C438	55.4 (12)
N232—C233—C238—C237	-177.8 (6)	N432—C433—C438—C437	-68.7 (10)
C234—C233—C238—C237	60.0 (8)	C434—C433—C438—C437	50.0 (10)
C236—C237—C238—C233	-58.9 (8)	C436—C437—C438—C433	-53.2 (12)

Table S28.Hydrogen-bond geometry (Å, º) for (**26**)

D—H···*A*	*D*—H	H···*A*	*D*···*A*	*D*—H···*A*
C117—H117···S231^{i}	0.95	2.83	3.659 (6)	146

N119—H119···S231^{i}	0.88	2.44	3.302 (6)	167
C123—H12*A*···S120	0.99	2.97	3.527 (8)	117
C128—H128···S220	0.95	2.87	3.707 (6)	147
N130—H130···S220	0.88	2.52	3.378 (5)	164
C134—H13*A*···S120	0.99	3.02	3.809 (9)	137
C217—H217···S131	0.95	2.91	3.736 (6)	146
N219—H219···S131	0.88	2.56	3.391 (6)	158
C227—H22*I*···S220	0.99	2.99	3.563 (9)	118
C228—H228···S120^{ii}	0.95	2.75	3.591 (7)	149
N230—H230···S120^{ii}	0.88	2.42	3.282 (6)	168
C238—H23*I*···N1*B*^{ii}	0.99	2.25	2.84 (3)	117
C317—H317···S431^{i}	0.95	2.93	3.757 (6)	147
N319—H319···S431^{i}	0.88	2.53	3.388 (6)	165
C327—H32*J*···S320	0.99	2.95	3.555 (9)	120
C328—H328···S420	0.95	2.90	3.708 (7)	143
N330—H330···S420	0.88	2.44	3.301 (6)	165
C334—H33*B*···S331	0.99	2.90	3.487 (8)	119
C417—H417···S331	0.95	2.83	3.645 (7)	144
N419—H419···S331	0.88	2.41	3.277 (6)	169
C427—H42*I*···N1*D*	0.99	2.32	2.94 (3)	120
C427—H42*J*···S420	0.99	3.01	3.559 (7)	116
C428—H428···S320^{ii}	0.95	2.89	3.729 (6)	148
N430—H430···S320^{ii}	0.88	2.56	3.386 (6)	157
C2*A*—H2*A*2···N230^{i}	0.98	2.50	3.470 (16)	170
C2*B*—H2*B*1···N119	0.98	2.66	3.55 (4)	151
C2*C*—H2*C*2···N419	0.98	2.52	3.452 (18)	160

Symmetry codes: (i) *x*+1, *y*, *z*; (ii) *x*-1, *y*, *z*.

Table. S29. Crystal data of **29**

$C_{25}H_{25}N_3O_3S{\cdot}CH_4O$	*F*(000) = 1016
M_r = 479.58	D_x = 1.305 Mg m^{-3}
Monoclinic, *P*2$_1$/*c (no.14)*	Cu *K* radiation, = 1.54178 Å
a = 19.5995 (5) Å	Cell parameters from 9340 reflections
b = 7.4181 (2) Å	= 2.4–72.1°
c = 18.2170 (5) Å	= 1.49 mm^{-1}
= 112.833 (2)°	*T* = 123 K
V = 2441.04 (12) Å^3	Plates, yellow

$Z = 4$	0.16 × 0.14 × 0.02 mm

Table. S30. Data collection of **29**.

Bruker D8 VENTURE diffractometer with PhotonII CPAD detector	4146 reflections with $I > 2 (I)$
Radiation source: INCOATEC microfocus sealed tube	$R_{int} = 0.052$
rotation in and , 1°, shutterless scans	$_{max} = 72.3°$, $_{min} = 2.5°$
Absorption correction: multi-scan *SADABS* (Sheldrick, 2014)	$h = -24$ 24
$T_{min} = 0.784$, $T_{max} = 0.942$	$k = -8$ 9
25915 measured reflections	$l = -22$ 22
4808 independent reflections	

Table. S31. Refinement of **29**.

Refinement on F^2	Primary atom site location: dual
Least-squares matrix: full	Secondary atom site location: difference Fourier map
$R[F^2 > 2 (F^2)] = 0.064$	Hydrogen site location: difference Fourier map
$wR(F^2) = 0.155$	H atoms treated by a mixture of independent and constrained refinement
$S = 1.17$	$w = 1/[^2(F_o^2) + (0.0676P)^2 + 2.1253P]$ where $P = (F_o^2 + 2F_c^2)/3$
4808 reflections	$(/)_{max} < 0.001$
315 parameters	$_{max} = 0.46$ e Å^{-3}
2 restraints	$_{min} = -0.32$ e Å^{-3}

Table. S32. Fractional atomic coordinates and isotropic or equivalent isotropic displacement parameters (Å^2) for (**29**)

	x	y	z	$U_{iso}*/U_{eq}$
C1	0.11316 (16)	0.5718 (4)	0.50023 (17)	0.0350 (6)
H1A	0.0693	0.5542	0.5137	0.042*
H1B	0.1281	0.6999	0.5100	0.042*
C2	0.17853 (19)	0.4494 (4)	0.55665 (17)	0.0403 (7)
H2A	0.2238	0.5238	0.5805	0.048*
H2B	0.1655	0.4030	0.6005	0.048*
C3	0.19461 (16)	0.2922 (4)	0.51314 (17)	0.0348 (6)
C4	0.14619 (17)	0.1459 (4)	0.48914 (18)	0.0352 (6)
H4	0.1178	0.1129	0.5191	0.042*
C5	0.13933 (16)	0.0484 (4)	0.42149 (18)	0.0348 (6)
H5	0.1052	-0.0487	0.4049	0.042*
C6	0.18160 (15)	0.0906 (4)	0.37769 (18)	0.0315 (6)

C7	0.24058 (15)	0.2091 (4)	0.41250 (18)	0.0340 (6)
H7	0.2770	0.2212	0.3903	0.041*
C8	0.24693 (16)	0.3094 (4)	0.47890 (18)	0.0342 (6)
H8	0.2872	0.3906	0.5014	0.041*
C9	0.15446 (17)	0.0417 (4)	0.29014 (19)	0.0376 (7)
H9A	0.1204	-0.0625	0.2796	0.045*
H9B	0.1973	0.0044	0.2777	0.045*
C10	0.11278 (16)	0.2031 (4)	0.23281 (18)	0.0338 (6)
H10A	0.1456	0.2546	0.2084	0.041*
H10B	0.0681	0.1557	0.1894	0.041*
C11	0.09020 (14)	0.3512 (4)	0.27624 (15)	0.0261 (5)
C12	0.03627 (14)	0.3163 (4)	0.30660 (17)	0.0297 (6)
H12	-0.0014	0.2302	0.2811	0.036*
C13	0.03700 (14)	0.4060 (4)	0.37361 (17)	0.0295 (6)
H13	-0.0008	0.3823	0.3927	0.035*
C14	0.09233 (14)	0.5304 (4)	0.41345 (16)	0.0266 (5)
C15	0.13614 (14)	0.5890 (3)	0.37308 (16)	0.0252 (5)
H15	0.1680	0.6896	0.3933	0.030*
C16	0.13462 (13)	0.5056 (3)	0.30488 (15)	0.0233 (5)
C17	0.18384 (14)	0.5713 (3)	0.26570 (15)	0.0243 (5)
C18	0.15187 (16)	0.5963 (4)	0.17650 (16)	0.0328 (6)
H18A	0.1507	0.7250	0.1640	0.049*
H18B	0.1015	0.5476	0.1541	0.049*
H18C	0.1827	0.5325	0.1535	0.049*
N18	0.25050 (12)	0.6135 (3)	0.31146 (13)	0.0255 (5)
N19	0.29197 (12)	0.6822 (3)	0.26942 (13)	0.0277 (5)
C20	0.35772 (14)	0.7156 (3)	0.31771 (16)	0.0249 (5)
S21	0.39255 (3)	0.68588 (9)	0.42227 (4)	0.02486 (17)
C22	0.47914 (14)	0.7678 (3)	0.43066 (16)	0.0247 (5)
C23	0.47906 (15)	0.8137 (4)	0.35014 (16)	0.0286 (6)
O23	0.53187 (11)	0.8740 (3)	0.33906 (12)	0.0371 (5)
N24	0.41120 (12)	0.7795 (3)	0.29274 (13)	0.0263 (5)
H24	0.4008 (17)	0.797 (4)	0.2414 (11)	0.032*
C25	0.53856 (14)	0.7914 (4)	0.49750 (16)	0.0272 (6)
H25	0.5829	0.8372	0.4952	0.033*
C26	0.53580 (14)	0.7470 (4)	0.57488 (16)	0.0270 (5)
O26	0.48278 (10)	0.6741 (3)	0.58111 (11)	0.0317 (4)

O27	0.59712 (10)	0.7935 (3)	0.63647 (11)	0.0299 (4)
C27	0.59730 (16)	0.7505 (4)	0.71532 (16)	0.0315 (6)
H27A	0.5841	0.6224	0.7176	0.038*
H27B	0.5609	0.8266	0.7264	0.038*
C28	0.67401 (17)	0.7870 (4)	0.77513 (18)	0.0377 (7)
H28A	0.6764	0.7599	0.8287	0.057*
H28B	0.7094	0.7109	0.7635	0.057*
H28C	0.6863	0.9142	0.7723	0.057*
O1M	0.36573 (12)	0.8133 (3)	0.13024 (13)	0.0415 (5)
H1M	0.4038 (16)	0.808 (6)	0.119 (2)	0.062*
C1M	0.32096 (18)	0.6661 (5)	0.0868 (2)	0.0432 (7)
H1M1	0.3521	0.5591	0.0930	0.065*
H1M2	0.2975	0.6980	0.0303	0.065*
H1M3	0.2826	0.6403	0.1074	0.065*

Table. S33. Atomic displacement parameters (Å^2) for (**29**)

	U^{11}	U^{22}	U^{33}	U^{12}	U^{13}	U^{23}
C1	0.0395 (15)	0.0400 (17)	0.0314 (15)	0.0016 (13)	0.0203 (12)	-0.0023 (12)
C2	0.0532 (18)	0.0403 (18)	0.0269 (14)	-0.0033 (14)	0.0152 (13)	0.0013 (13)
C3	0.0401 (15)	0.0336 (16)	0.0253 (14)	0.0042 (12)	0.0069 (12)	0.0081 (12)
C4	0.0416 (16)	0.0286 (15)	0.0369 (15)	0.0056 (12)	0.0167 (13)	0.0130 (12)
C5	0.0406 (15)	0.0184 (13)	0.0460 (17)	-0.0004 (11)	0.0174 (13)	0.0052 (12)
C6	0.0354 (14)	0.0196 (13)	0.0403 (16)	0.0060 (11)	0.0154 (12)	0.0030 (11)
C7	0.0279 (13)	0.0346 (16)	0.0376 (15)	0.0070 (11)	0.0108 (12)	0.0057 (13)
C8	0.0291 (13)	0.0333 (15)	0.0333 (15)	0.0014 (12)	0.0045 (11)	0.0065 (12)
C9	0.0411 (16)	0.0264 (15)	0.0482 (18)	-0.0006 (12)	0.0205 (14)	-0.0076 (13)
C10	0.0374 (15)	0.0296 (15)	0.0342 (15)	-0.0074 (12)	0.0138 (12)	-0.0105 (12)
C11	0.0246 (12)	0.0261 (14)	0.0252 (12)	-0.0015 (10)	0.0070 (10)	0.0022 (11)
C12	0.0225 (12)	0.0283 (14)	0.0360 (15)	-0.0015 (10)	0.0087 (11)	0.0019 (12)
C13	0.0219 (12)	0.0318 (15)	0.0385 (15)	0.0032 (10)	0.0156 (11)	0.0070 (12)
C14	0.0301 (13)	0.0230 (13)	0.0300 (13)	0.0074 (10)	0.0152 (11)	0.0027 (11)
C15	0.0269 (12)	0.0195 (12)	0.0305 (13)	-0.0006 (10)	0.0124 (11)	-0.0010 (10)
C16	0.0220 (11)	0.0224 (12)	0.0266 (12)	0.0033 (9)	0.0106 (10)	0.0028 (10)
C17	0.0271 (12)	0.0205 (12)	0.0275 (13)	-0.0014 (10)	0.0129 (11)	-0.0015 (10)
C18	0.0336 (14)	0.0369 (16)	0.0279 (14)	-0.0067 (12)	0.0119 (11)	-0.0011 (12)
N18	0.0291 (11)	0.0259 (11)	0.0262 (11)	-0.0024 (9)	0.0159 (9)	-0.0005 (9)
N19	0.0295 (11)	0.0304 (12)	0.0277 (11)	-0.0066 (9)	0.0160 (9)	-0.0022 (9)

C20	0.0318 (13)	0.0196 (13)	0.0283 (13)	-0.0024 (10)	0.0171 (11)	-0.0037 (10)
S21	0.0266 (3)	0.0268 (3)	0.0250 (3)	-0.0030 (2)	0.0141 (2)	-0.0023 (3)
C22	0.0282 (12)	0.0211 (12)	0.0300 (13)	-0.0014 (10)	0.0169 (11)	-0.0045 (10)
C23	0.0338 (14)	0.0266 (14)	0.0316 (14)	-0.0055 (11)	0.0194 (12)	-0.0065 (11)
O23	0.0342 (10)	0.0489 (13)	0.0350 (11)	-0.0143 (9)	0.0207 (9)	-0.0060 (9)
N24	0.0305 (11)	0.0291 (12)	0.0239 (11)	-0.0062 (9)	0.0156 (9)	-0.0033 (9)
C25	0.0265 (12)	0.0265 (14)	0.0317 (14)	-0.0014 (10)	0.0148 (11)	-0.0042 (11)
C26	0.0276 (13)	0.0233 (13)	0.0316 (14)	0.0016 (10)	0.0130 (11)	-0.0042 (11)
O26	0.0283 (9)	0.0387 (11)	0.0317 (10)	-0.0036 (8)	0.0156 (8)	-0.0039 (8)
O27	0.0305 (9)	0.0345 (11)	0.0262 (9)	-0.0049 (8)	0.0126 (8)	-0.0050 (8)
C27	0.0364 (14)	0.0319 (15)	0.0292 (14)	-0.0007 (12)	0.0159 (12)	0.0001 (12)
C28	0.0405 (16)	0.0384 (17)	0.0309 (15)	0.0008 (13)	0.0102 (13)	-0.0006 (13)
O1M	0.0361 (11)	0.0591 (15)	0.0365 (11)	-0.0045 (10)	0.0221 (9)	-0.0018 (10)
C1M	0.0399 (16)	0.051 (2)	0.0415 (17)	-0.0009 (14)	0.0187 (14)	-0.0036 (15)

Table. S34. Geometric parameters (Å, º) for (**29**)

C1—C14	1.503 (4)	C16—C17	1.487 (3)
C1—C2	1.581 (4)	C17—N18	1.286 (3)
C1—H1A	0.9900	C17—C18	1.509 (4)
C1—H1B	0.9900	C18—H18A	0.9800
C2—C3	1.511 (4)	C18—H18B	0.9800
C2—H2A	0.9900	C18—H18C	0.9800
C2—H2B	0.9900	N18—N19	1.411 (3)
C3—C4	1.395 (4)	N19—C20	1.271 (3)
C3—C8	1.398 (4)	C20—N24	1.378 (3)
C4—C5	1.390 (4)	C20—S21	1.770 (3)
C4—H4	0.9500	S21—C22	1.753 (3)
C5—C6	1.391 (4)	C22—C25	1.330 (4)
C5—H5	0.9500	C22—C23	1.505 (4)
C6—C7	1.393 (4)	C23—O23	1.214 (3)
C6—C9	1.517 (4)	C23—N24	1.359 (4)
C7—C8	1.384 (4)	N24—H24	0.887 (18)
C7—H7	0.9500	C25—C26	1.469 (4)
C8—H8	0.9500	C25—H25	0.9500
C9—C10	1.590 (4)	C26—O26	1.215 (3)
C9—H9A	0.9900	C26—O27	1.332 (3)
C9—H9B	0.9900	O27—C27	1.470 (3)

C10—C11	1.517 (4)	C27—C28	1.500 (4)
C10—H10A	0.9900	C27—H27A	0.9900
C10—H10B	0.9900	C27—H27B	0.9900
C11—C12	1.394 (4)	C28—H28A	0.9800
C11—C16	1.410 (4)	C28—H28B	0.9800
C12—C13	1.385 (4)	C28—H28C	0.9800
C12—H12	0.9500	O1M—C1M	1.432 (4)
C13—C14	1.394 (4)	O1M—H1M	0.842 (19)
C13—H13	0.9500	C1M—H1M1	0.9800
C14—C15	1.399 (4)	C1M—H1M2	0.9800
C15—C16	1.378 (4)	C1M—H1M3	0.9800
C15—H15	0.9500		
C14—C1—C2	112.8 (2)	C16—C15—H15	119.0
C14—C1—H1A	109.0	C14—C15—H15	119.0
C2—C1—H1A	109.0	C15—C16—C11	119.5 (2)
C14—C1—H1B	109.0	C15—C16—C17	119.6 (2)
C2—C1—H1B	109.0	C11—C16—C17	120.7 (2)
H1A—C1—H1B	107.8	N18—C17—C16	117.0 (2)
C3—C2—C1	112.6 (2)	N18—C17—C18	124.1 (2)
C3—C2—H2A	109.1	C16—C17—C18	118.8 (2)
C1—C2—H2A	109.1	C17—C18—H18A	109.5
C3—C2—H2B	109.1	C17—C18—H18B	109.5
C1—C2—H2B	109.1	H18A—C18—H18B	109.5
H2A—C2—H2B	107.8	C17—C18—H18C	109.5
C4—C3—C8	117.3 (3)	H18A—C18—H18C	109.5
C4—C3—C2	120.6 (3)	H18B—C18—H18C	109.5
C8—C3—C2	120.2 (3)	C17—N18—N19	113.0 (2)
C5—C4—C3	120.2 (3)	C20—N19—N18	109.7 (2)
C5—C4—H4	119.9	N19—C20—N24	122.4 (2)
C3—C4—H4	119.9	N19—C20—S21	125.76 (19)
C4—C5—C6	121.2 (3)	N24—C20—S21	111.88 (19)
C4—C5—H5	119.4	C22—S21—C20	90.38 (12)
C6—C5—H5	119.4	C25—C22—C23	122.1 (2)
C5—C6—C7	117.0 (3)	C25—C22—S21	126.9 (2)
C5—C6—C9	120.4 (3)	C23—C22—S21	111.02 (19)
C7—C6—C9	121.3 (3)	O23—C23—N24	125.7 (3)

C8—C7—C6	120.9 (3)	O23—C23—C22	124.5 (3)
C8—C7—H7	119.5	N24—C23—C22	109.8 (2)
C6—C7—H7	119.5	C23—N24—C20	116.9 (2)
C7—C8—C3	120.5 (3)	C23—N24—H24	123 (2)
C7—C8—H8	119.7	C20—N24—H24	120 (2)
C3—C8—H8	119.7	C22—C25—C26	120.2 (2)
C6—C9—C10	113.1 (2)	C22—C25—H25	119.9
C6—C9—H9A	109.0	C26—C25—H25	119.9
C10—C9—H9A	109.0	O26—C26—O27	124.1 (2)
C6—C9—H9B	109.0	O26—C26—C25	122.7 (2)
C10—C9—H9B	109.0	O27—C26—C25	113.1 (2)
H9A—C9—H9B	107.8	C26—O27—C27	115.2 (2)
C11—C10—C9	112.5 (2)	O27—C27—C28	107.0 (2)
C11—C10—H10A	109.1	O27—C27—H27A	110.3
C9—C10—H10A	109.1	C28—C27—H27A	110.3
C11—C10—H10B	109.1	O27—C27—H27B	110.3
C9—C10—H10B	109.1	C28—C27—H27B	110.3
H10A—C10—H10B	107.8	H27A—C27—H27B	108.6
C12—C11—C16	117.3 (2)	C27—C28—H28A	109.5
C12—C11—C10	119.5 (2)	C27—C28—H28B	109.5
C16—C11—C10	121.2 (2)	H28A—C28—H28B	109.5
C13—C12—C11	120.7 (3)	C27—C28—H28C	109.5
C13—C12—H12	119.7	H28A—C28—H28C	109.5
C11—C12—H12	119.7	H28B—C28—H28C	109.5
C12—C13—C14	121.1 (2)	C1M—O1M—H1M	104 (3)
C12—C13—H13	119.5	O1M—C1M—H1M1	109.5
C14—C13—H13	119.5	O1M—C1M—H1M2	109.5
C13—C14—C15	116.4 (2)	H1M1—C1M—H1M2	109.5
C13—C14—C1	121.4 (2)	O1M—C1M—H1M3	109.5
C15—C14—C1	121.2 (3)	H1M1—C1M—H1M3	109.5
C16—C15—C14	122.1 (2)	H1M2—C1M—H1M3	109.5
C14—C1—C2—C3	-12.5 (4)	C12—C11—C16—C17	-168.9 (2)
C1—C2—C3—C4	-72.9 (3)	C10—C11—C16—C17	27.5 (4)
C1—C2—C3—C8	91.0 (3)	C15—C16—C17—N18	41.6 (3)
C8—C3—C4—C5	-14.6 (4)	C11—C16—C17—N18	-134.0 (3)

C2—C3—C4—C5	149.8 (3)	C15—C16—C17—C18	-134.3 (3)
C3—C4—C5—C6	1.8 (4)	C11—C16—C17—C18	50.1 (3)
C4—C5—C6—C7	12.3 (4)	C16—C17—N18—N19	-177.2 (2)
C4—C5—C6—C9	-155.0 (3)	C18—C17—N18—N19	-1.5 (4)
C5—C6—C7—C8	-13.6 (4)	C17—N18—N19—C20	-177.9 (2)
C9—C6—C7—C8	153.6 (3)	N18—N19—C20—N24	178.1 (2)
C6—C7—C8—C3	0.8 (4)	N18—N19—C20—S21	-1.2 (3)
C4—C3—C8—C7	13.3 (4)	N19—C20—S21—C22	-178.9 (3)
C2—C3—C8—C7	-151.1 (3)	N24—C20—S21—C22	1.7 (2)
C5—C6—C9—C10	95.8 (3)	C20—S21—C22—C25	178.0 (3)
C7—C6—C9—C10	-71.0 (3)	C20—S21—C22—C23	-1.1 (2)
C6—C9—C10—C11	-16.0 (3)	C25—C22—C23—O23	0.4 (4)
C9—C10—C11—C12	-67.1 (3)	S21—C22—C23—O23	179.6 (2)
C9—C10—C11—C16	96.1 (3)	C25—C22—C23—N24	-178.9 (2)
C16—C11—C12—C13	-13.5 (4)	S21—C22—C23—N24	0.2 (3)
C10—C11—C12—C13	150.4 (3)	O23—C23—N24—C20	-178.3 (3)
C11—C12—C13—C14	-1.4 (4)	C22—C23—N24—C20	1.1 (3)
C12—C13—C14—C15	13.9 (4)	N19—C20—N24—C23	178.6 (3)
C12—C13—C14—C1	-154.8 (3)	S21—C20—N24—C23	-1.9 (3)
C2—C1—C14—C13	91.9 (3)	C23—C22—C25—C26	179.1 (2)
C2—C1—C14—C15	-76.2 (3)	S21—C22—C25—C26	0.1 (4)
C13—C14—C15—C16	-11.8 (4)	C22—C25—C26—O26	6.4 (4)
C1—C14—C15—C16	156.9 (3)	C22—C25—C26—O27	-174.1 (2)
C14—C15—C16—C11	-2.9 (4)	O26—C26—O27—C27	-0.1 (4)
C14—C15—C16—C17	-178.5 (2)	C25—C26—O27—C27	-179.6 (2)
C12—C11—C16—C15	15.5 (4)	C26—O27—C27—C28	171.7 (2)
C10—C11—C16—C15	-148.1 (3)		

Table. S35. Hydrogen-bond geometry (Å, º) for (**29**)

D—H···*A*	*D*—H	H···*A*	*D*···*A*	*D*—H···*A*
N24—H24···O1*M*	0.89 (2)	1.87 (2)	2.752 (3)	171 (3)

O1*M*—H1*M*···O26[i]	0.84 (2)	1.93 (2)	2.769 (3)	171 (4)
C1*M*—H1*M*1···O23[ii]	0.98	2.53	3.437 (4)	154

Symmetry codes: (i) *x*, -*y*+3/2, *z*-1/2; (ii) -*x*+1, *y*-1/2, -*z*+1/2.

^{1}H-NMR and ^{13}C-NMR data of starting materials

^{1}H-NMR of 5c

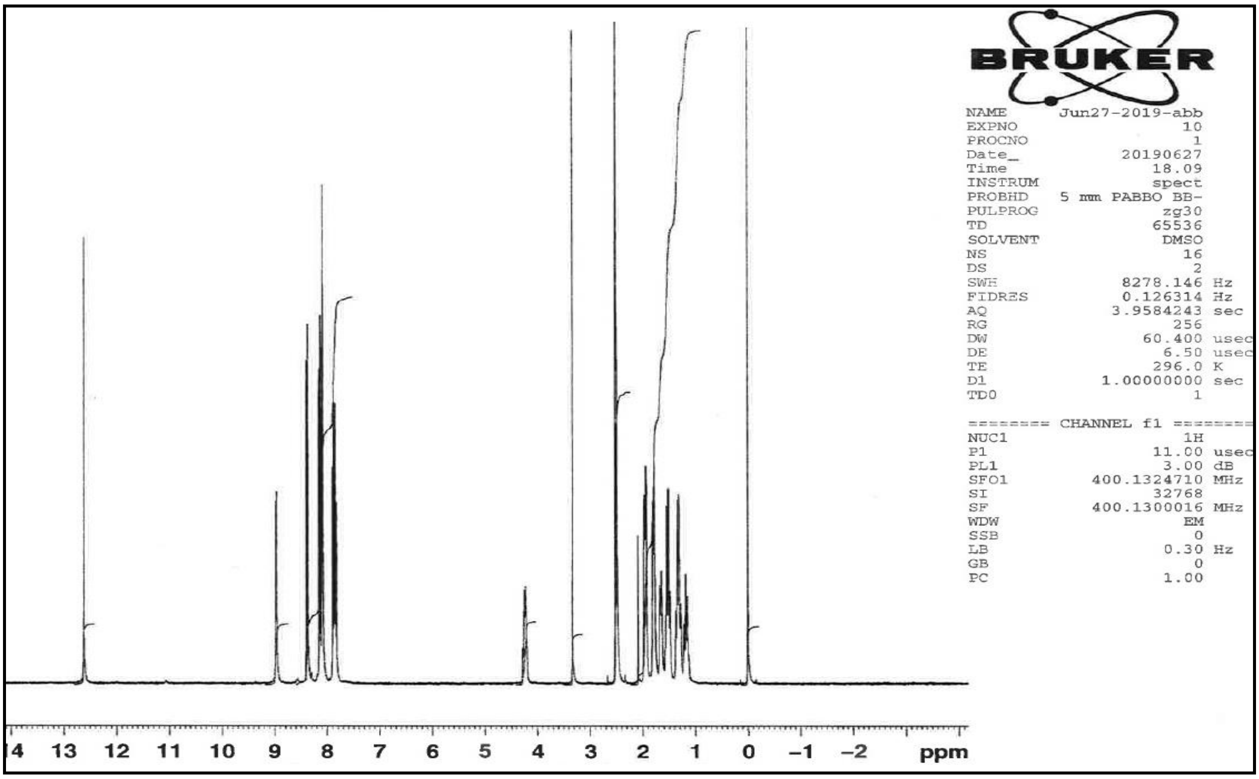

^{13}C-NMR of 5c

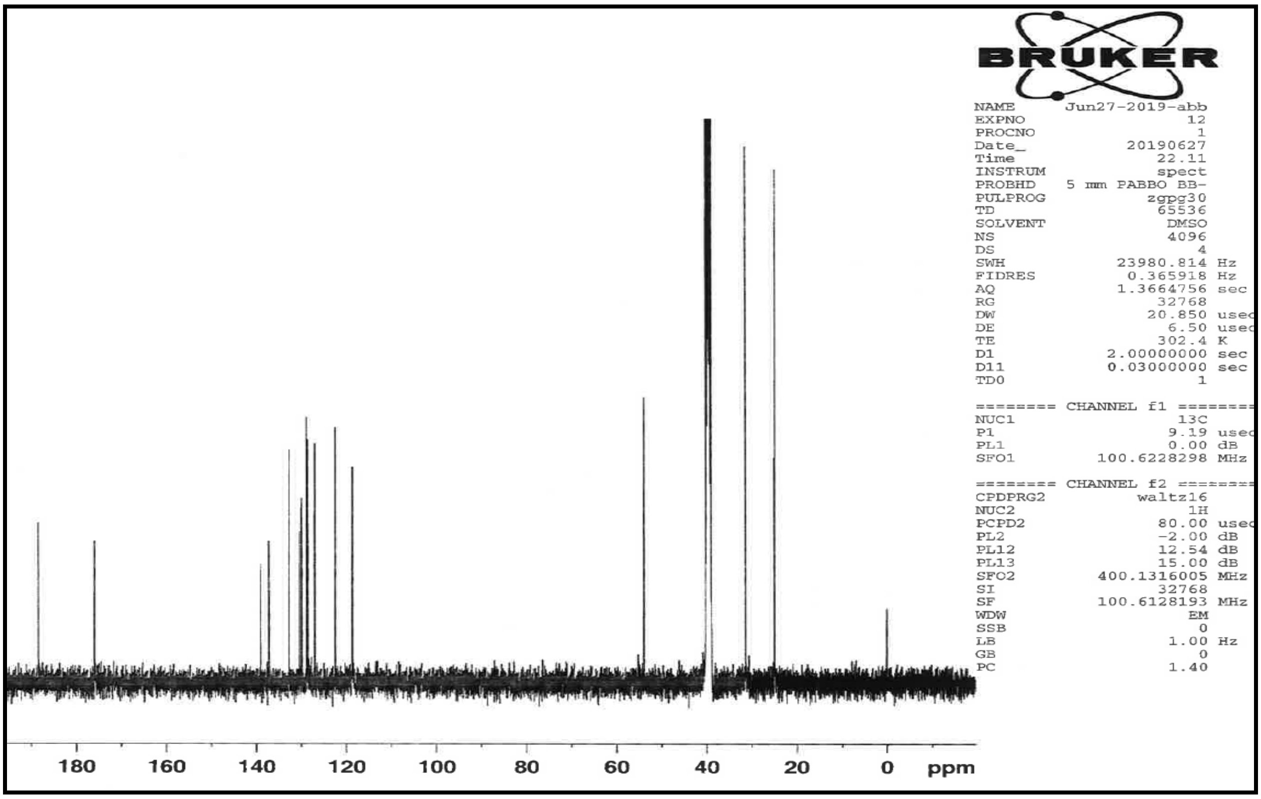

^{1}H-NMR of 22f

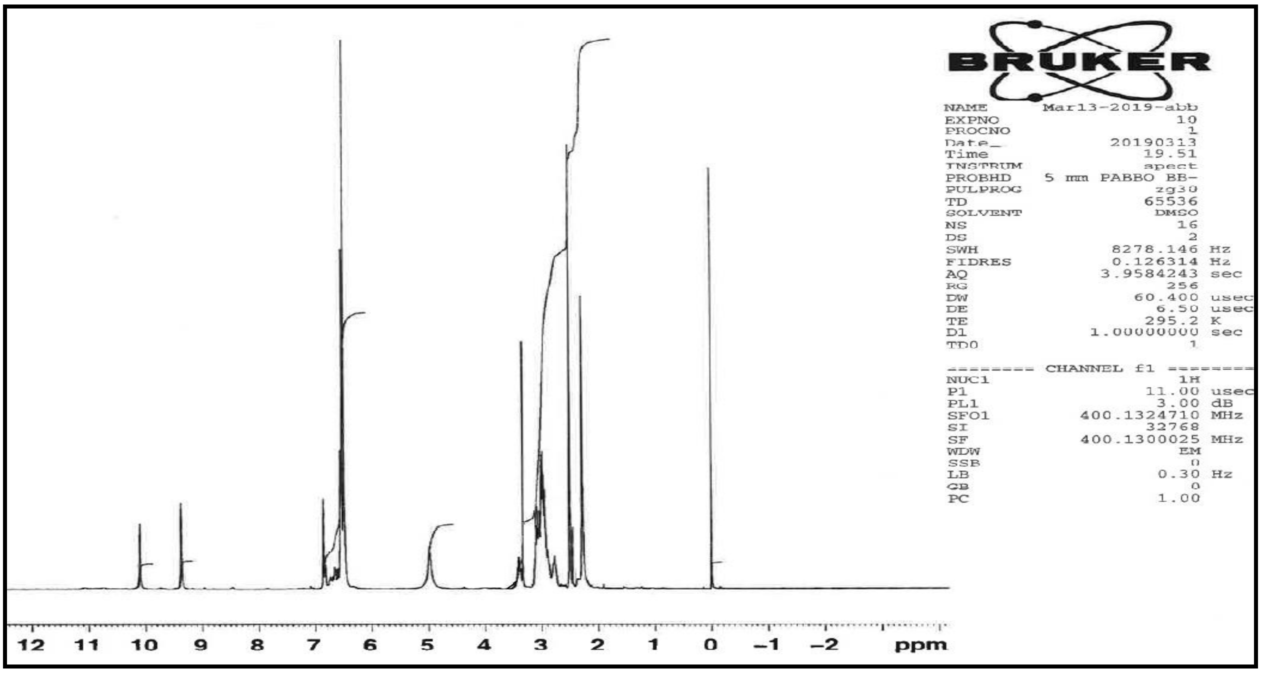

^{13}C-NMR of 22f

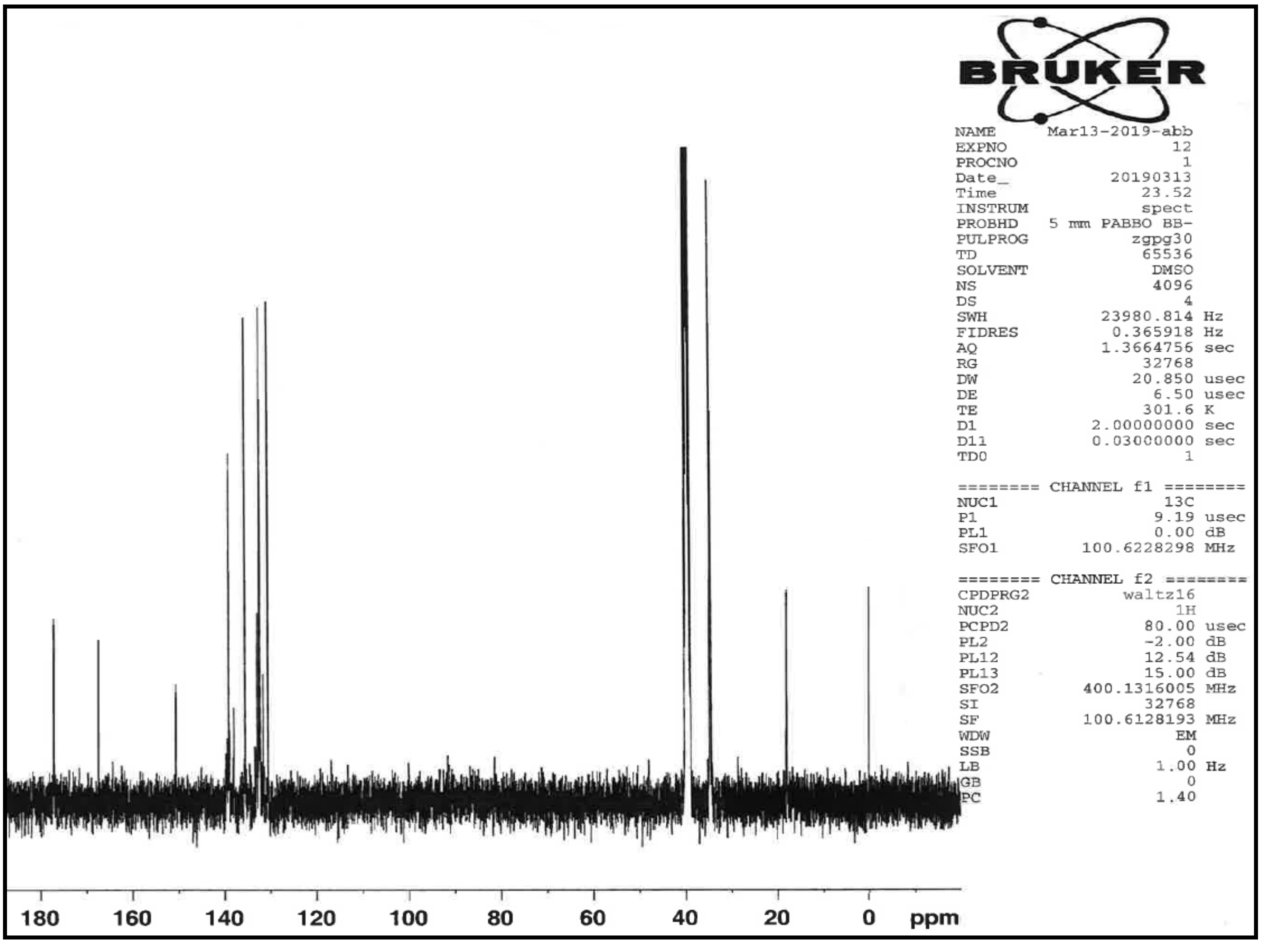

^{1}H-NMR of 23

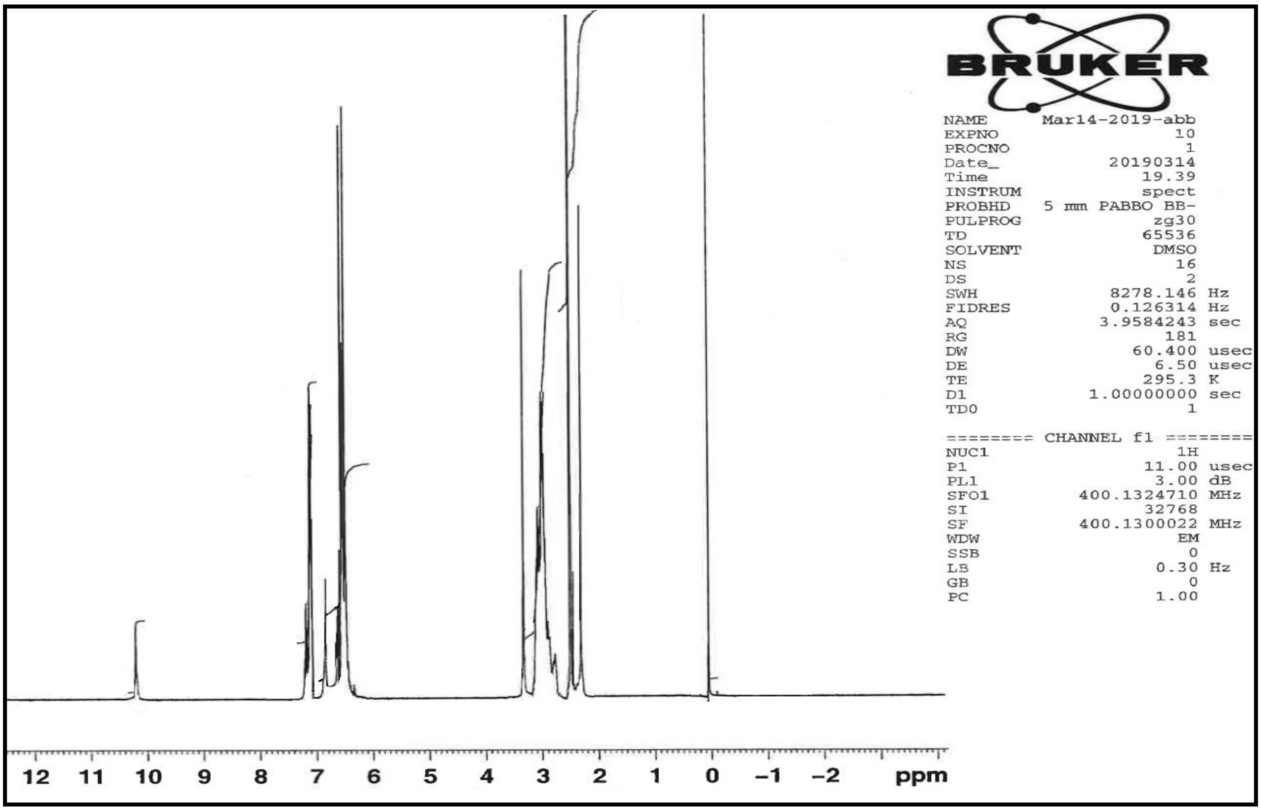

[13]2019-7-25 -C-NMR of 23

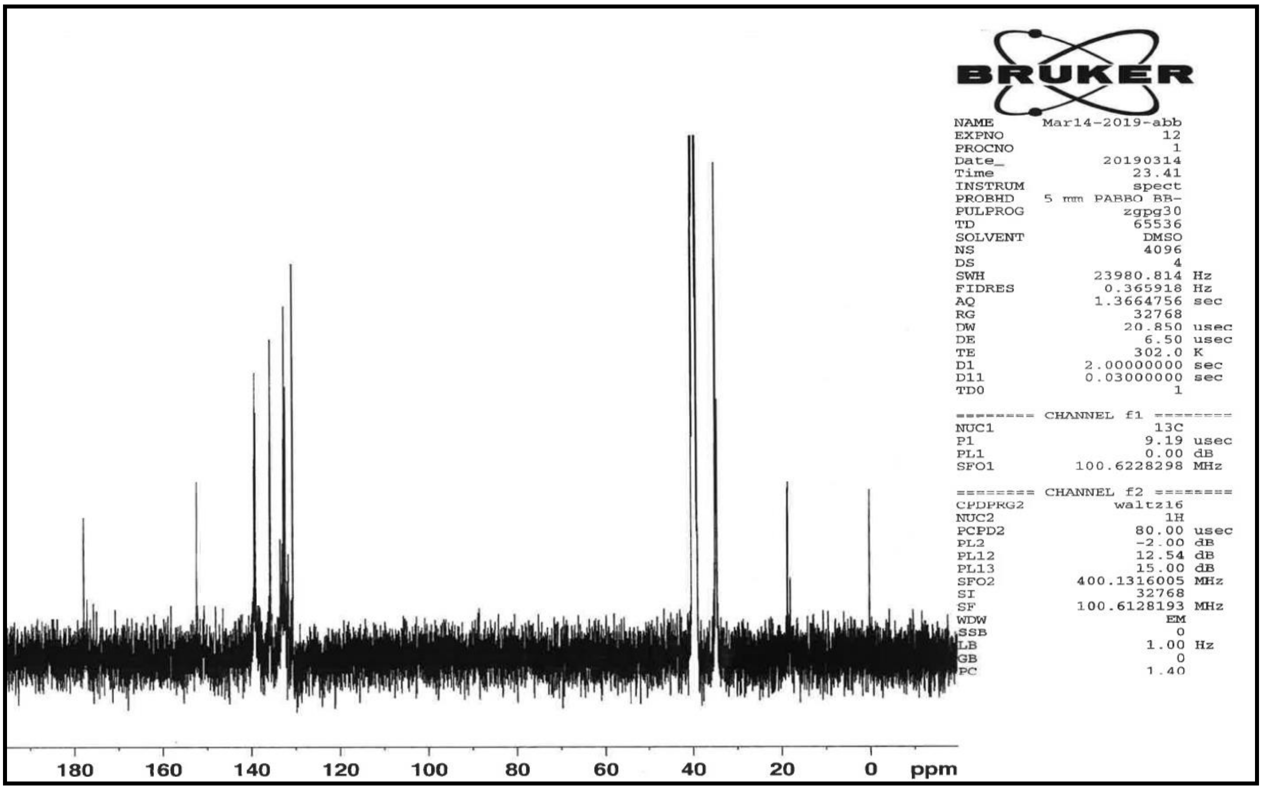

^{1}H-NMR of 25

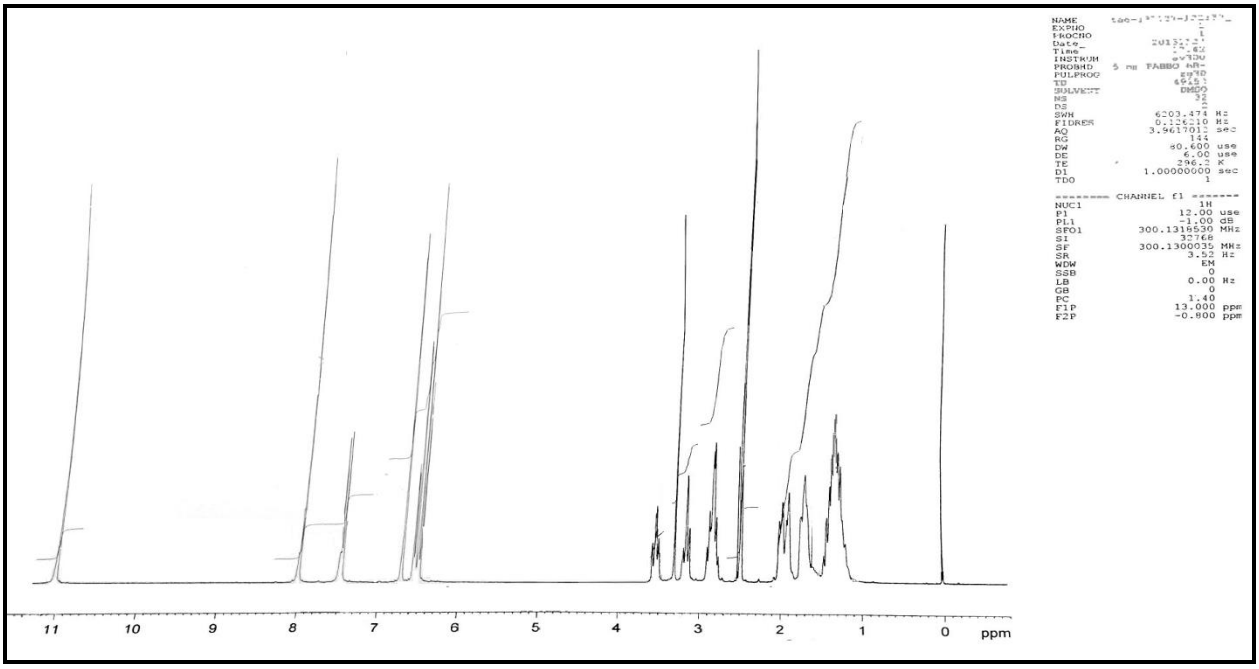

^{13}C-NMR of 25

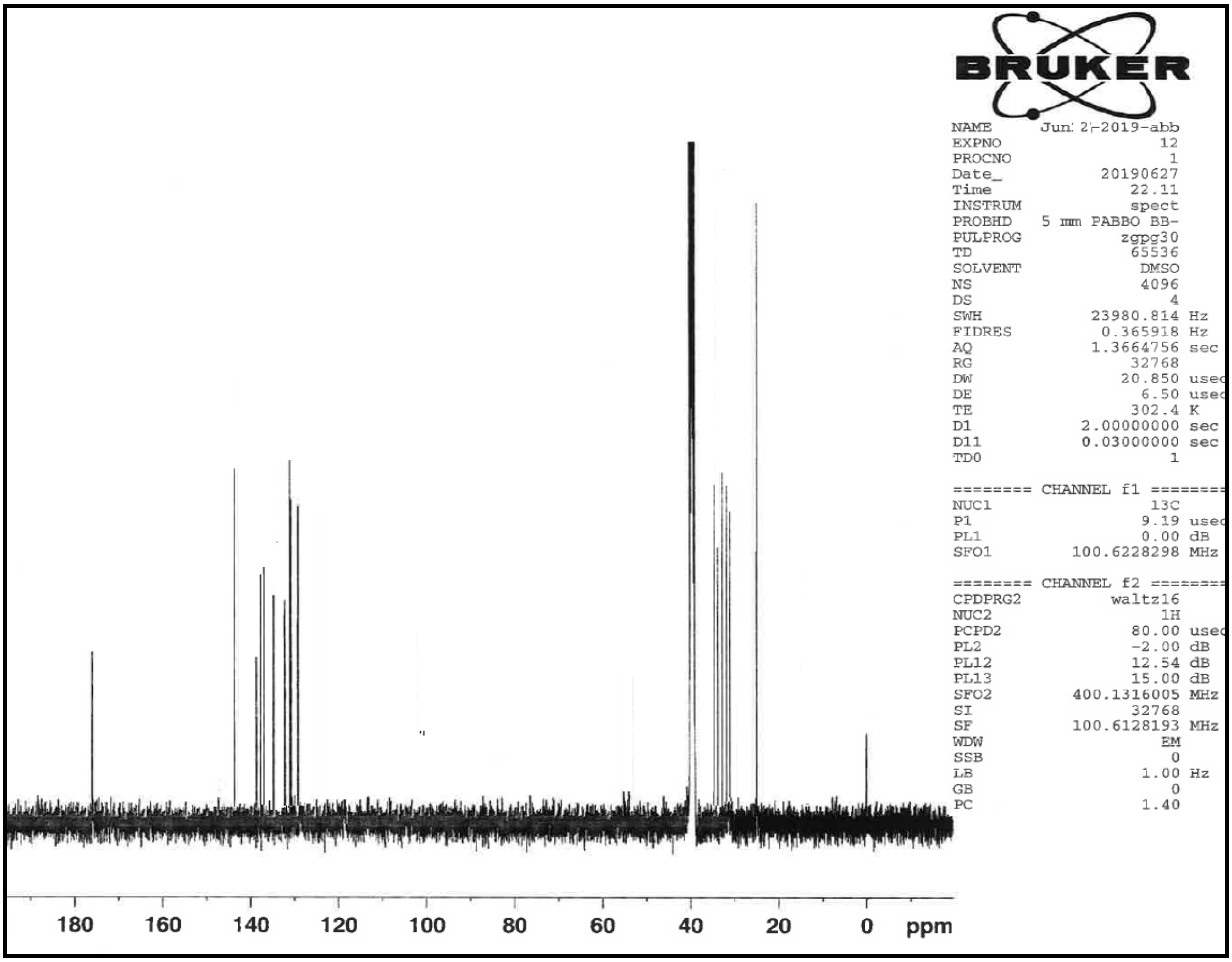

^{1}H-NMR and ^{13}C-NMR data of products

^{1}H-NMR of 7a

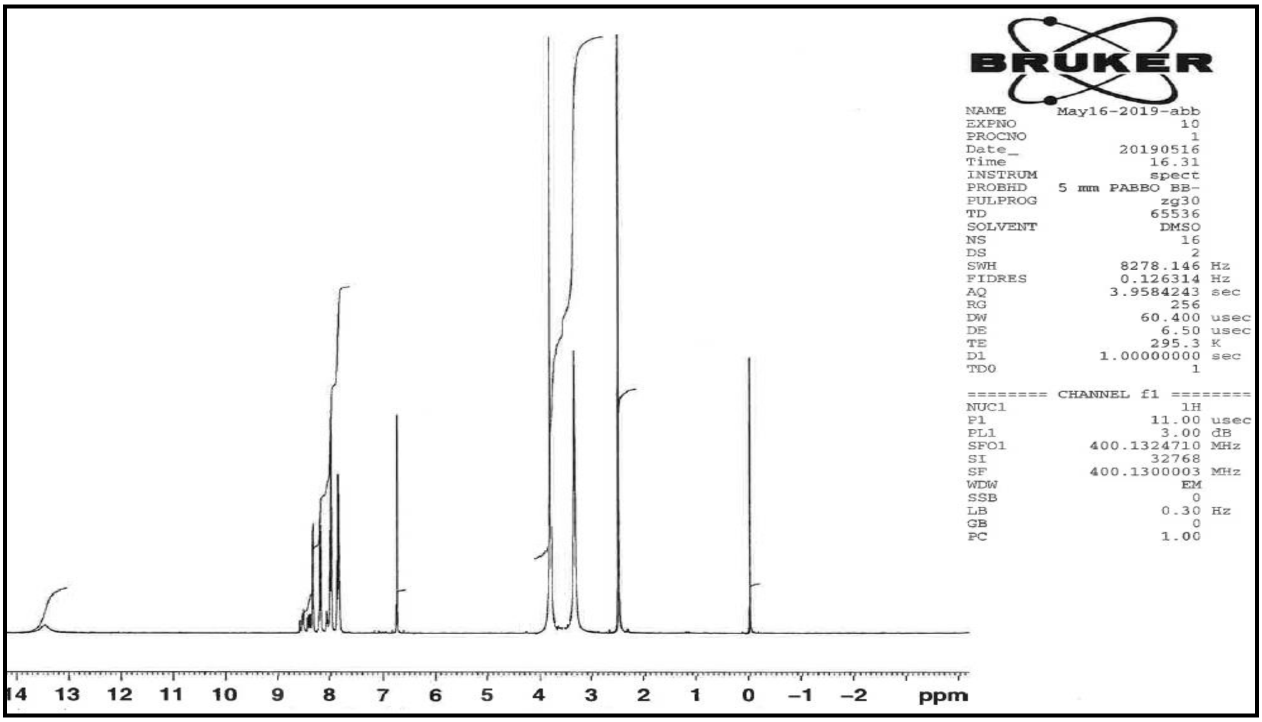

^{13}C-NMR of 7a

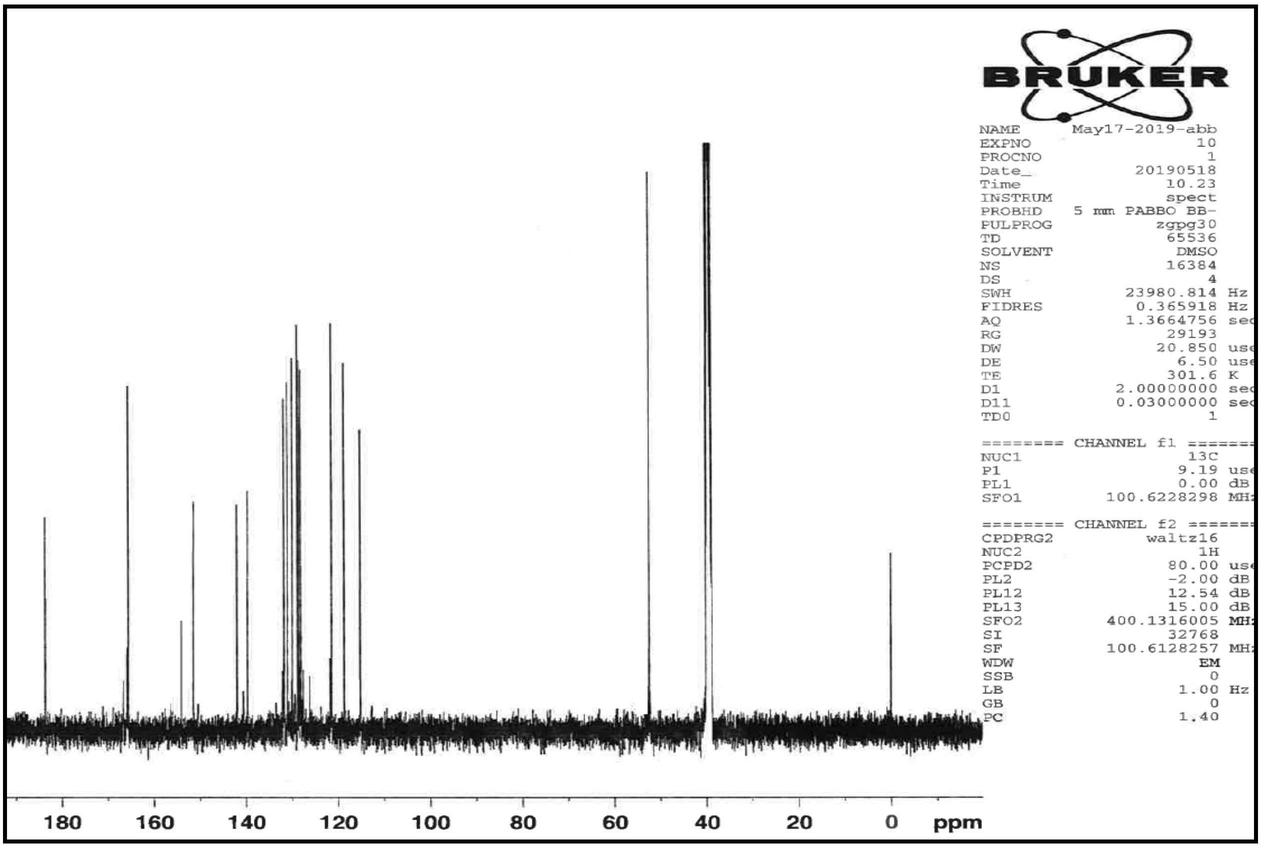

^{1}H-NMR of 7b

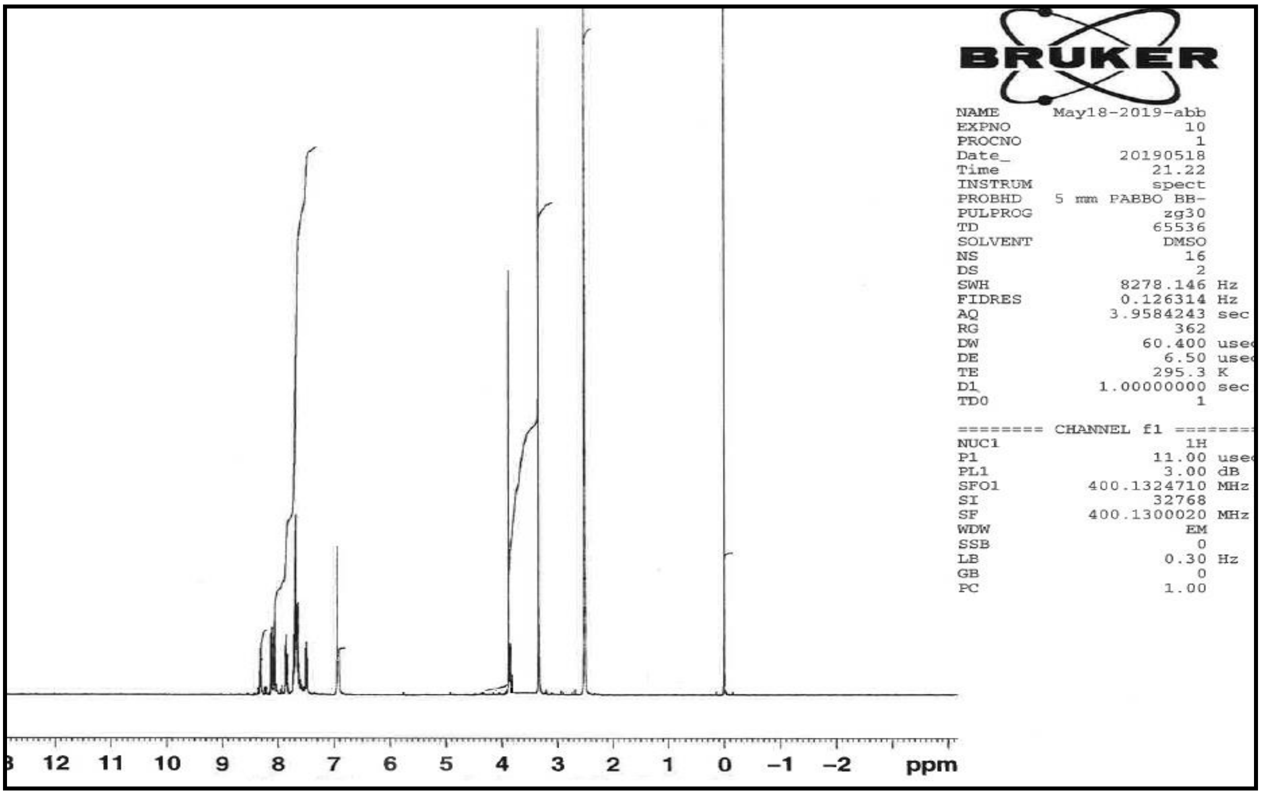

^{13}C-NMR of 7b

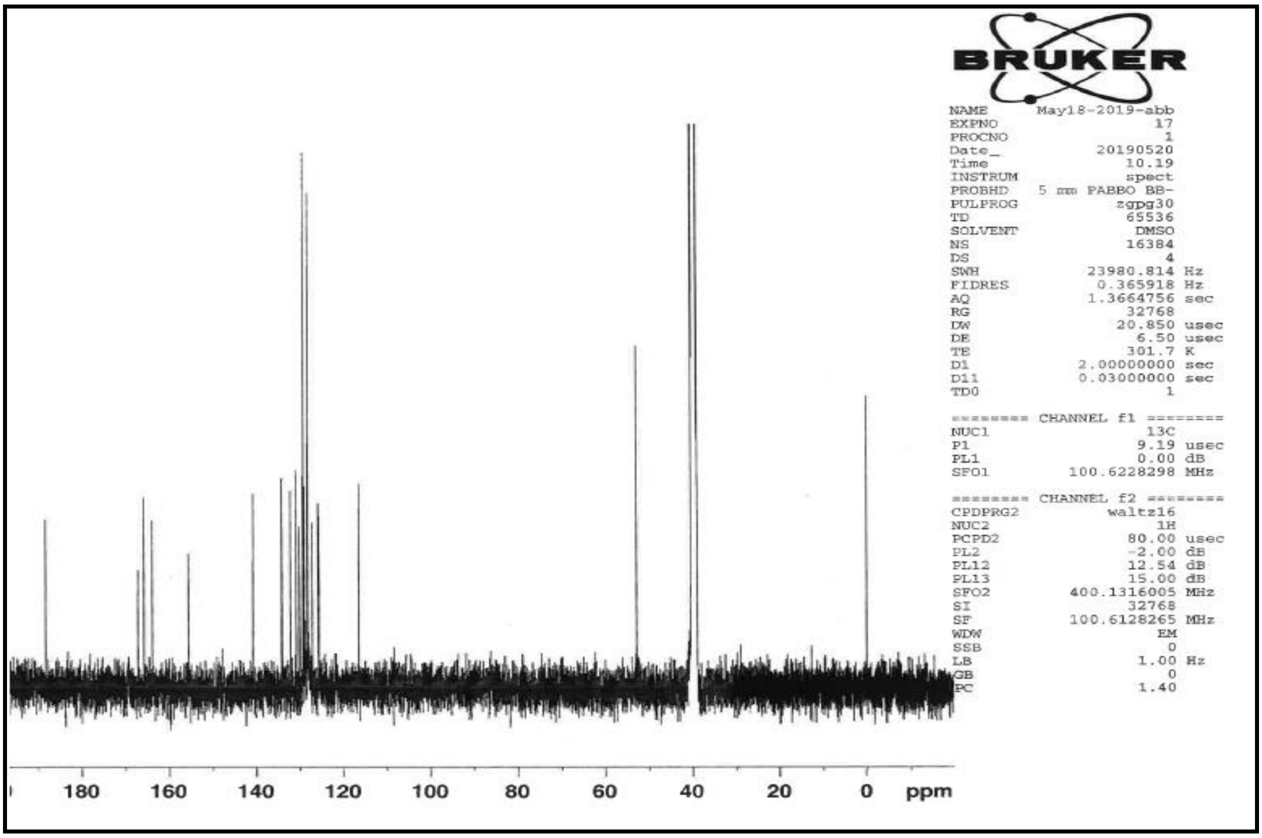

^{1}H-NMR of 7c

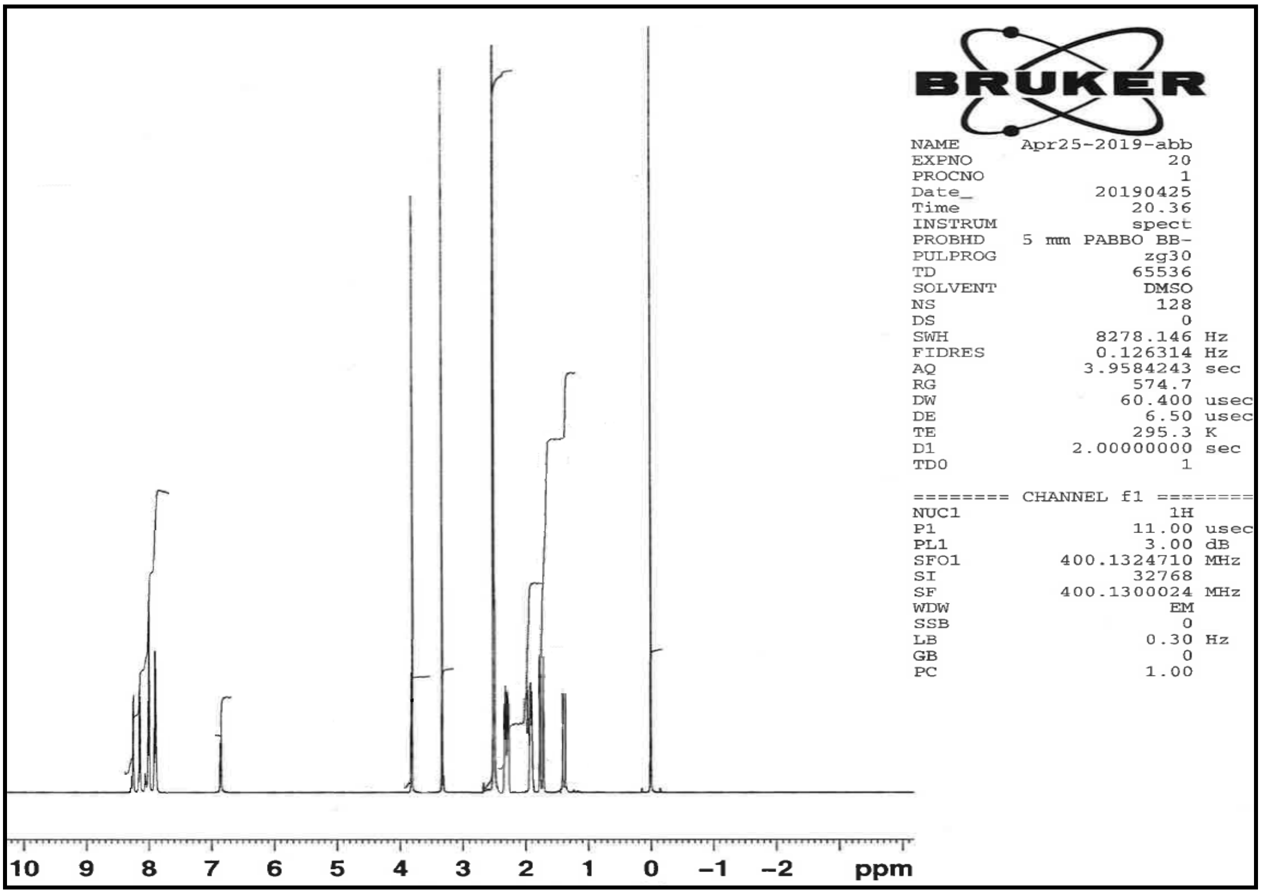

^{13}C-NMR of 7c

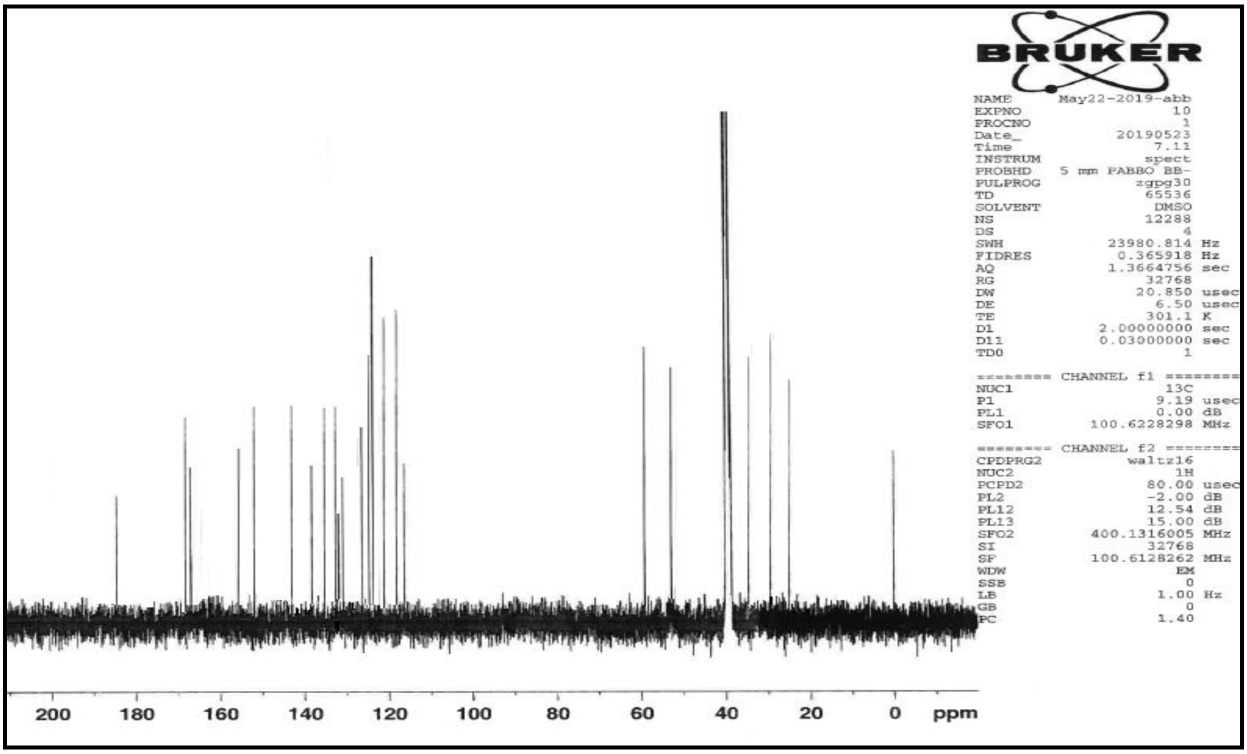

^{1}H-NMR of 7d

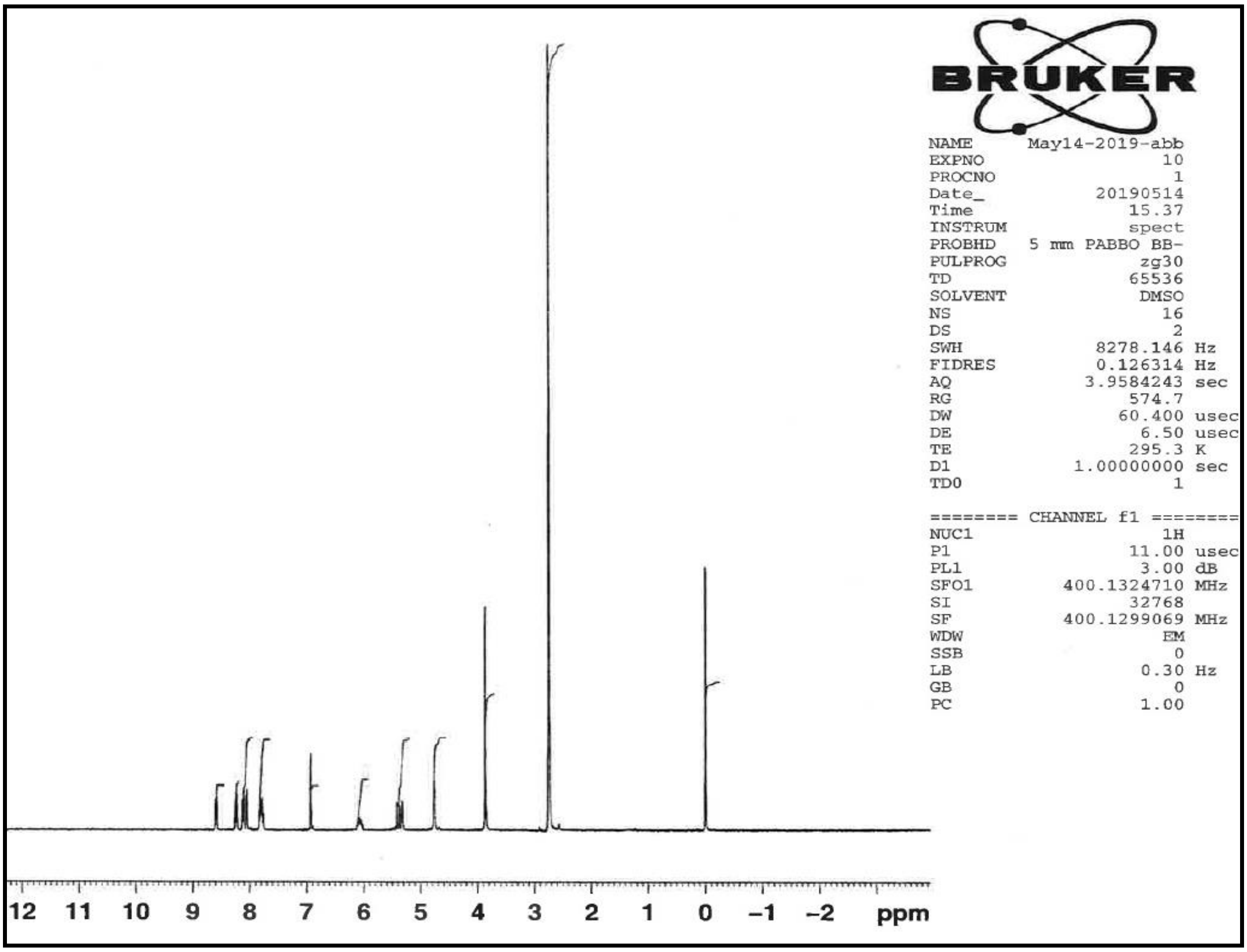

^{13}C-NMR of 7d

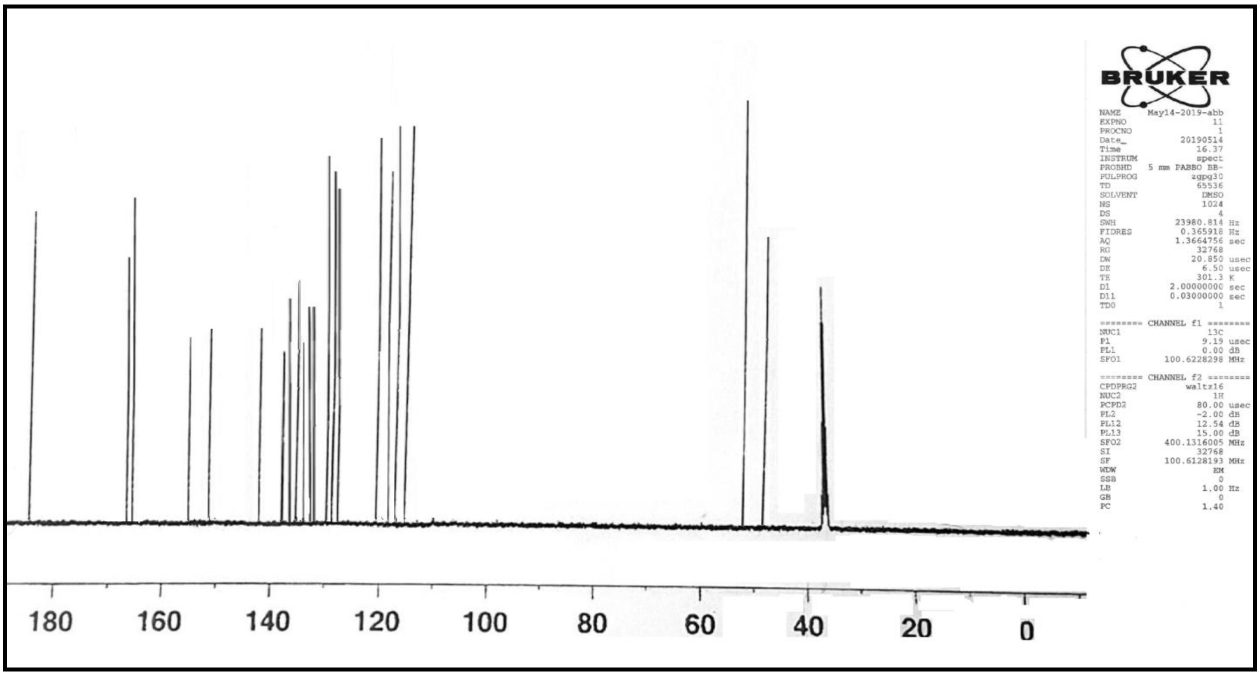

^{1}H-NMR of 7e

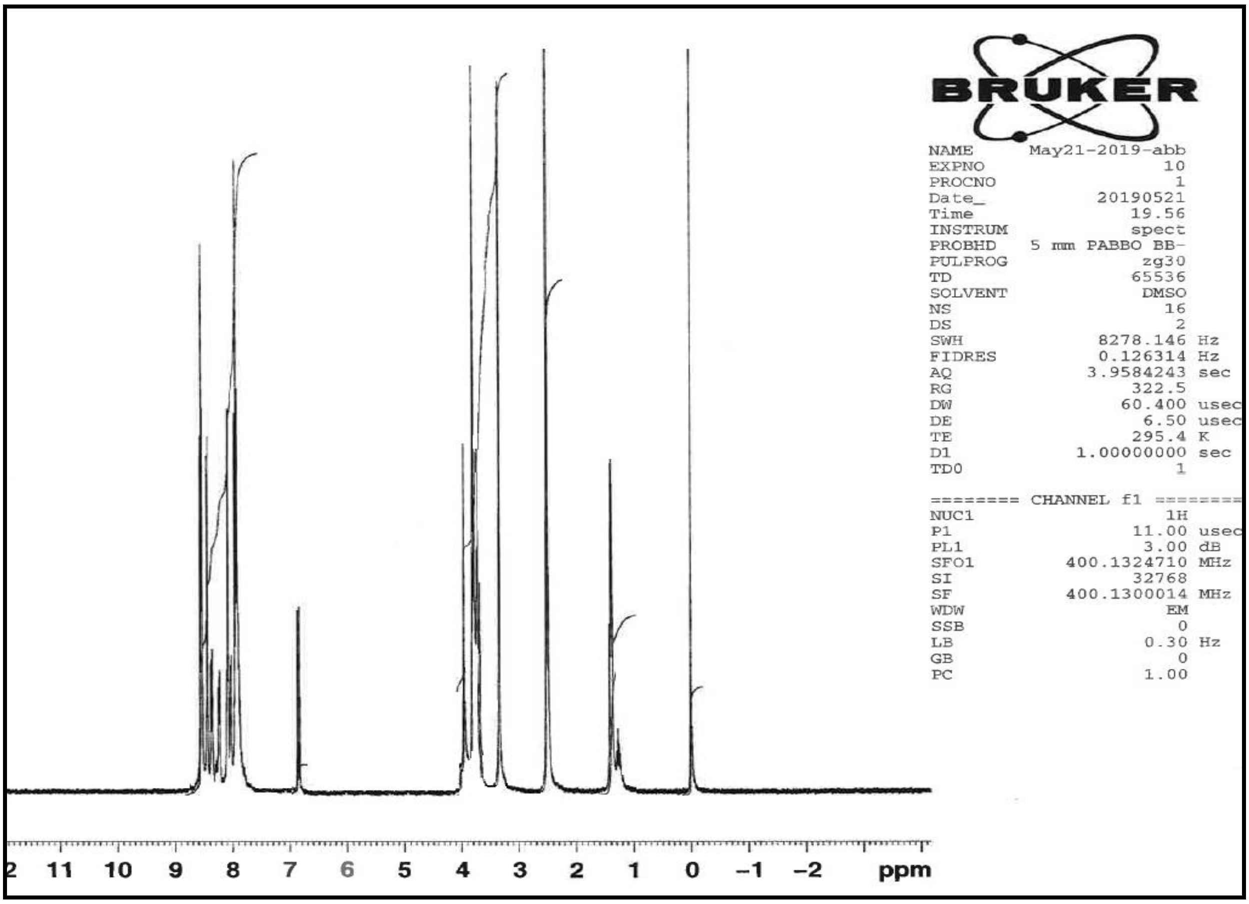

^{13}C-NMR of 7e

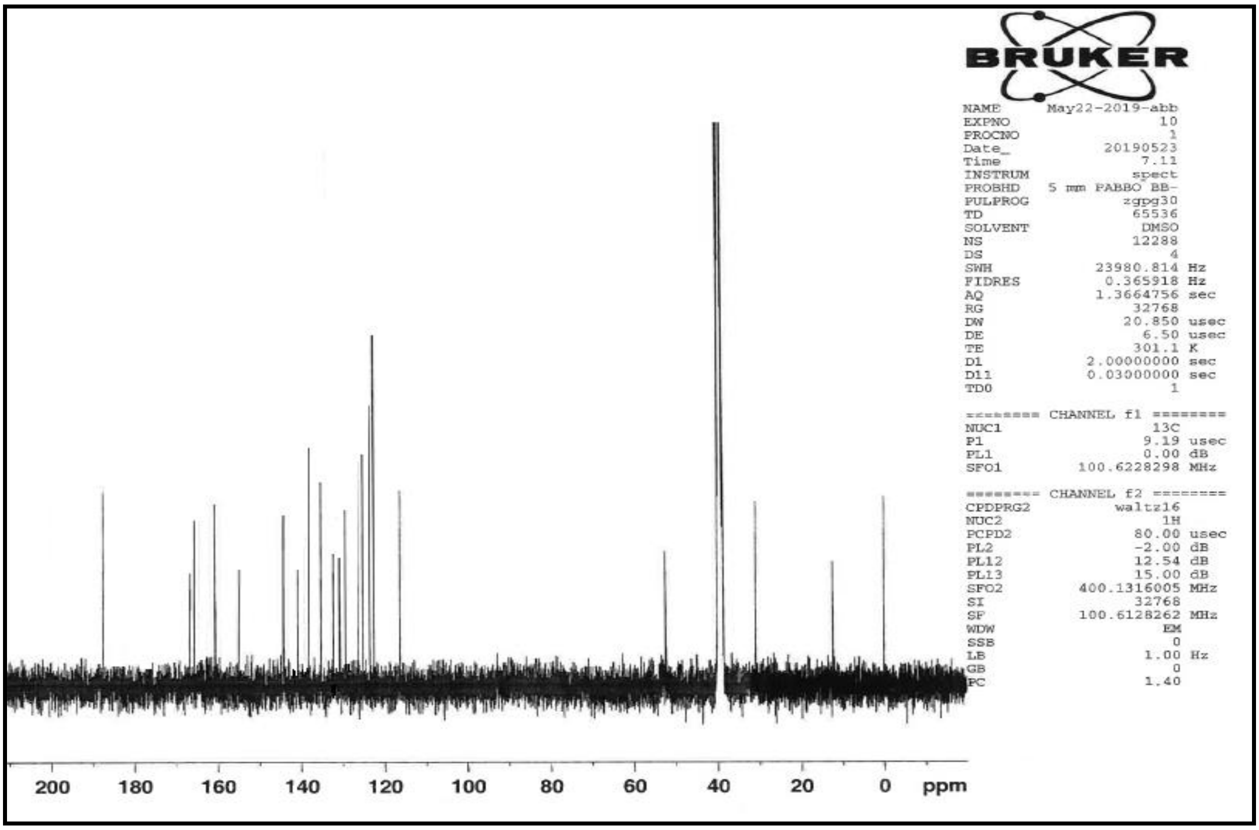

^{1}H-NMR of 8d

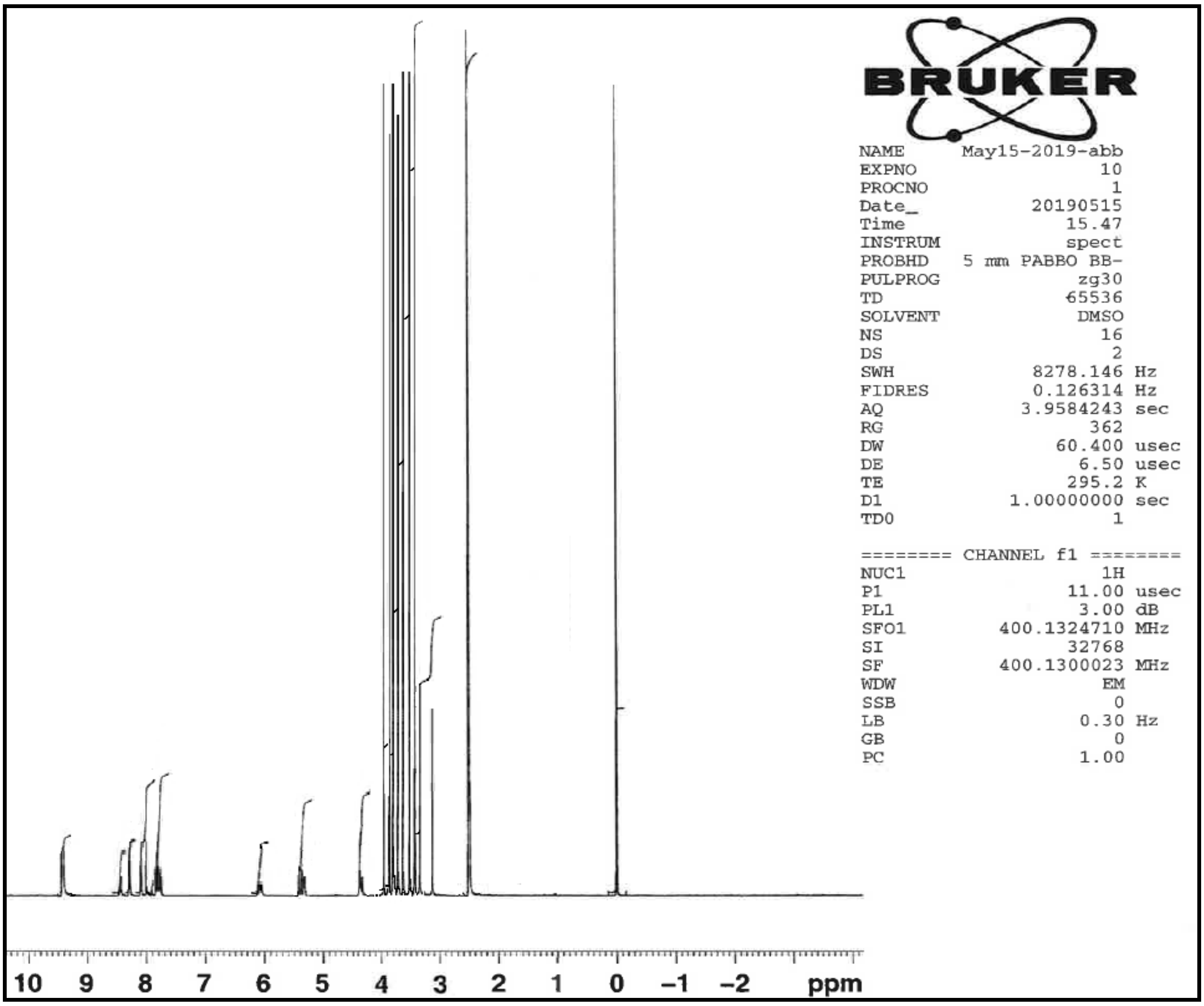

C-NMR of 8d

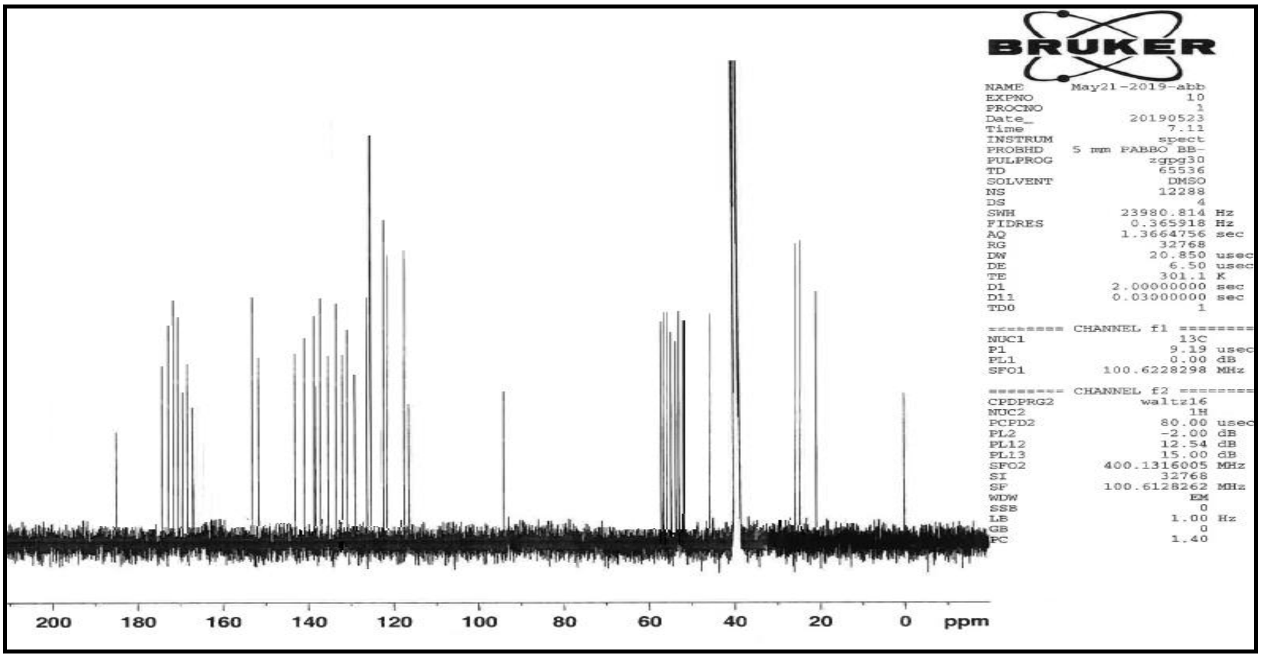

^{1}H-NMR of 26

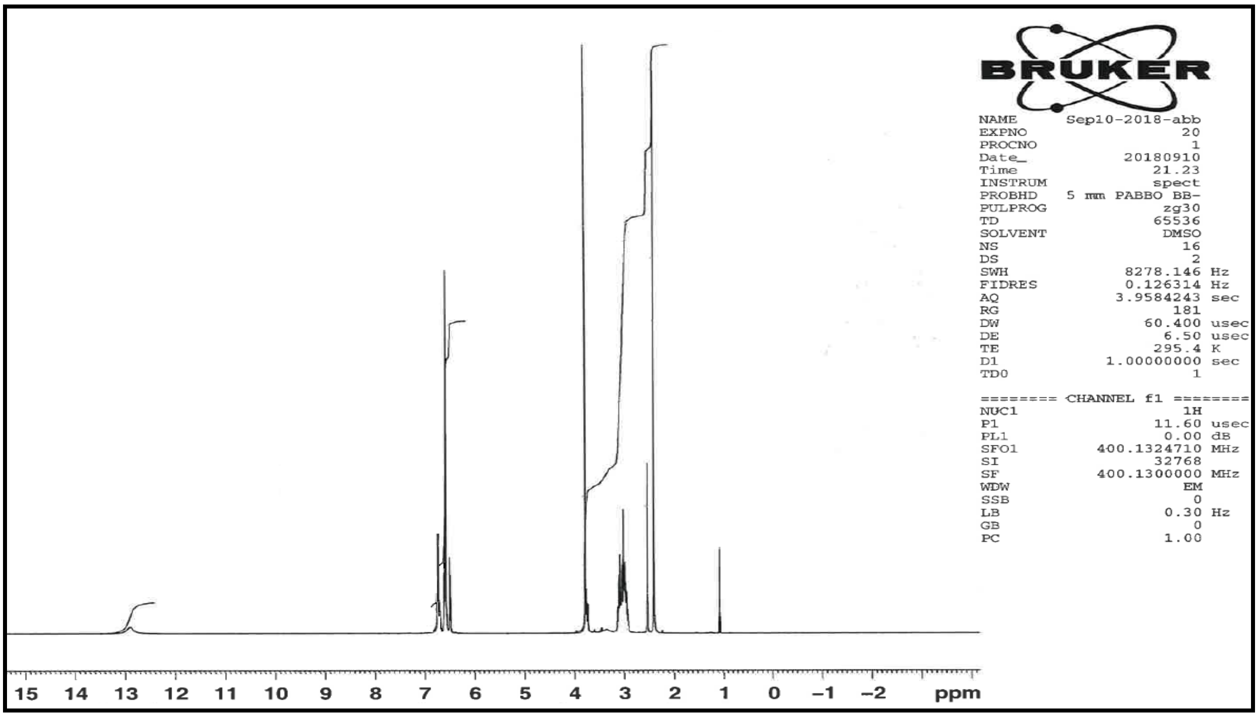

^{13}C-NMR of 26

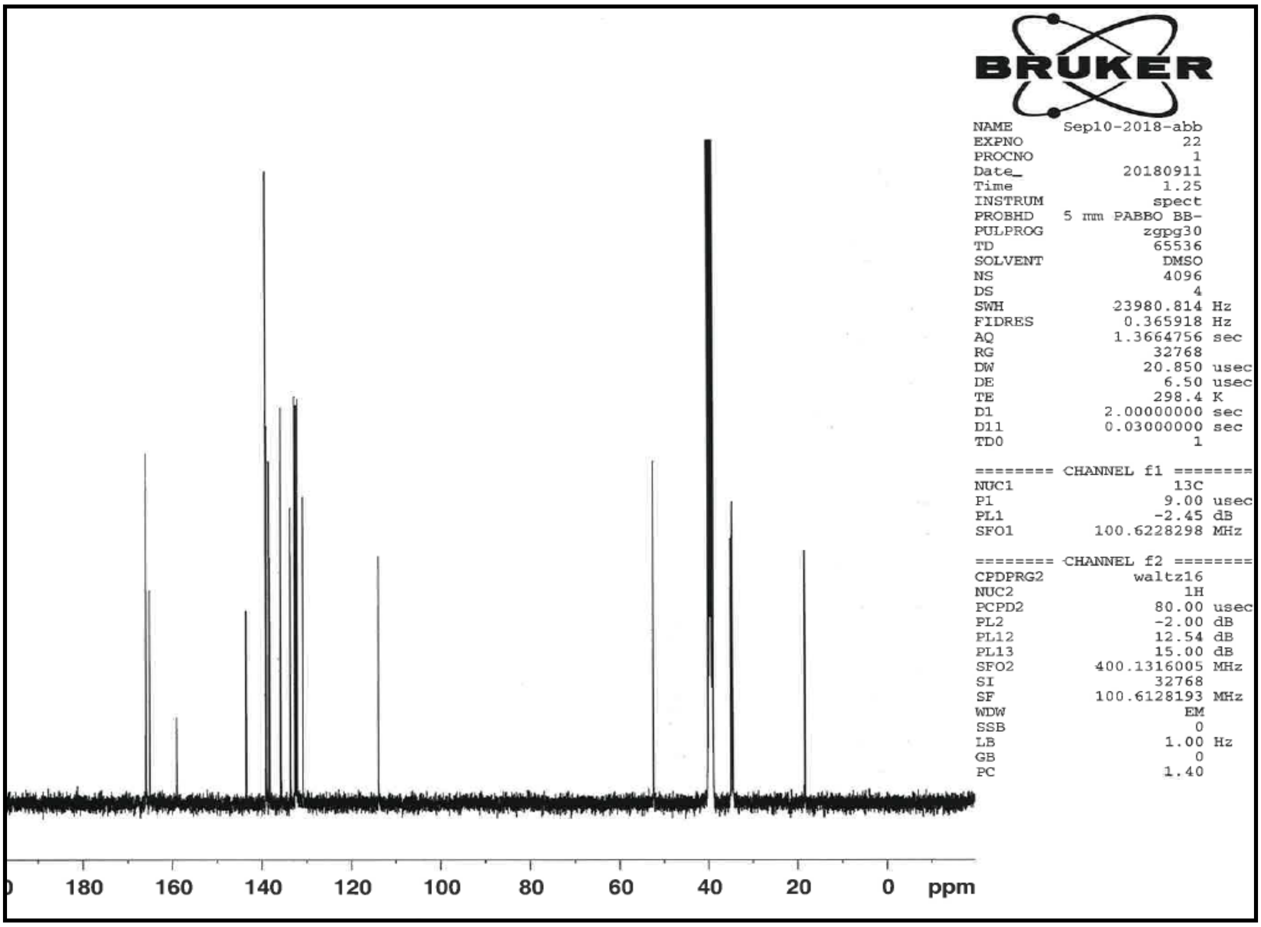

^{1}H-NMR of 27

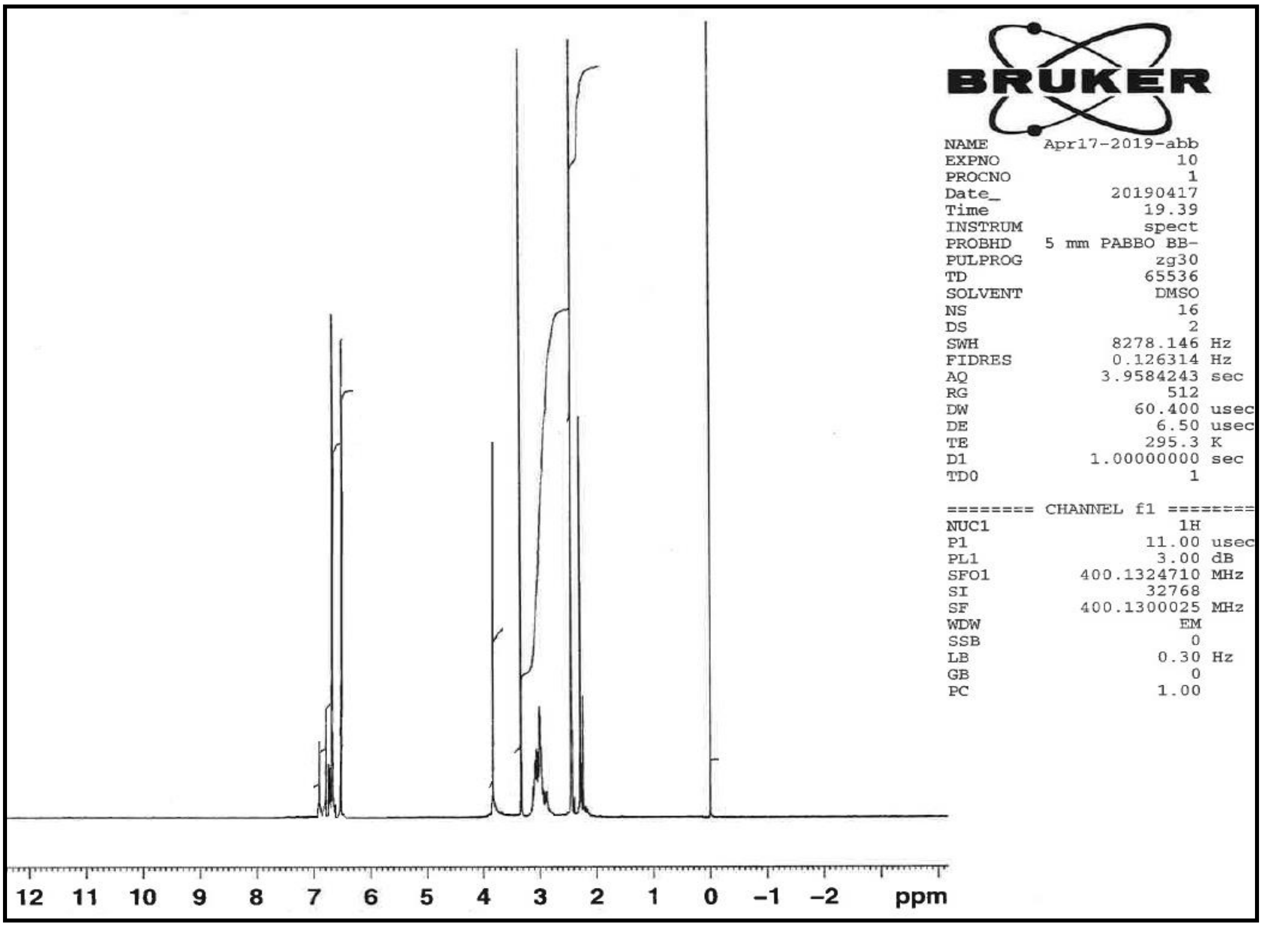

^{13}C-NMR of 27

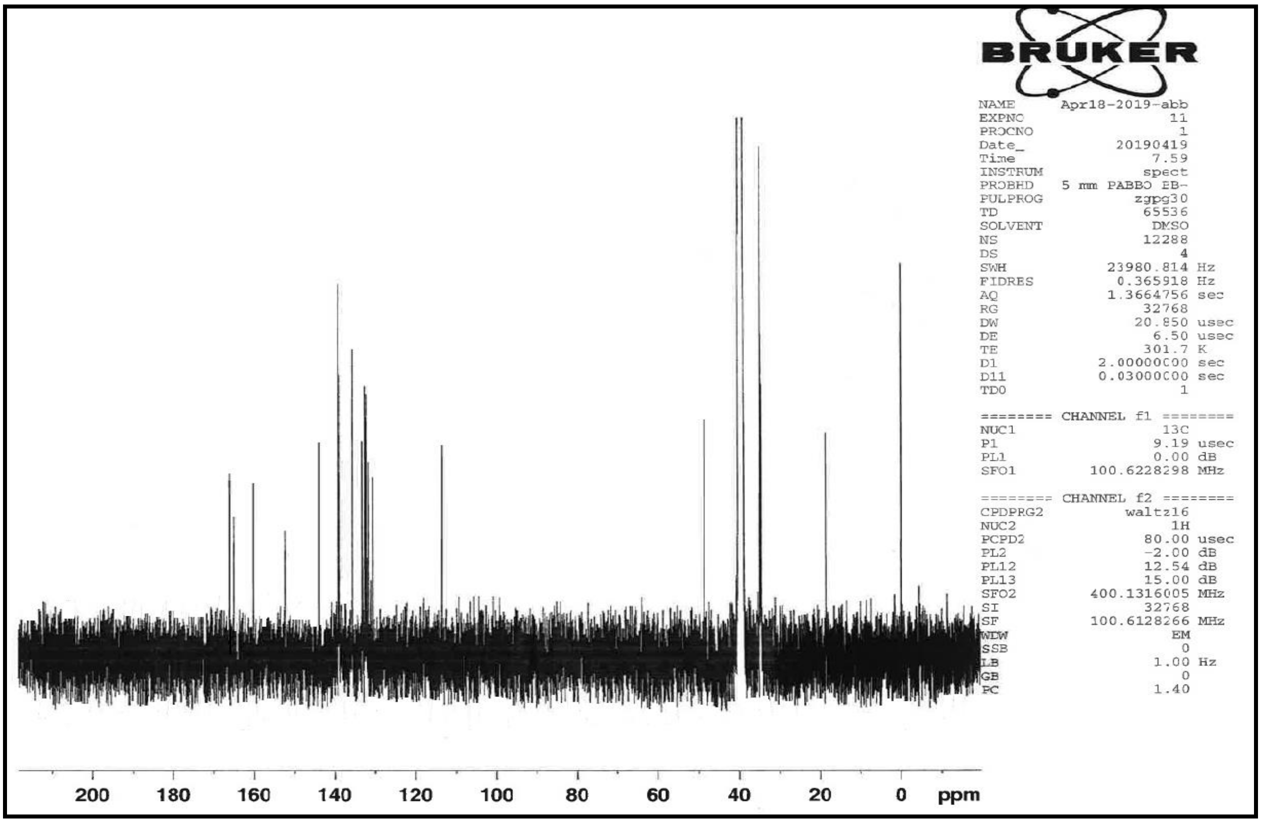

^{1}H-NMR of 28

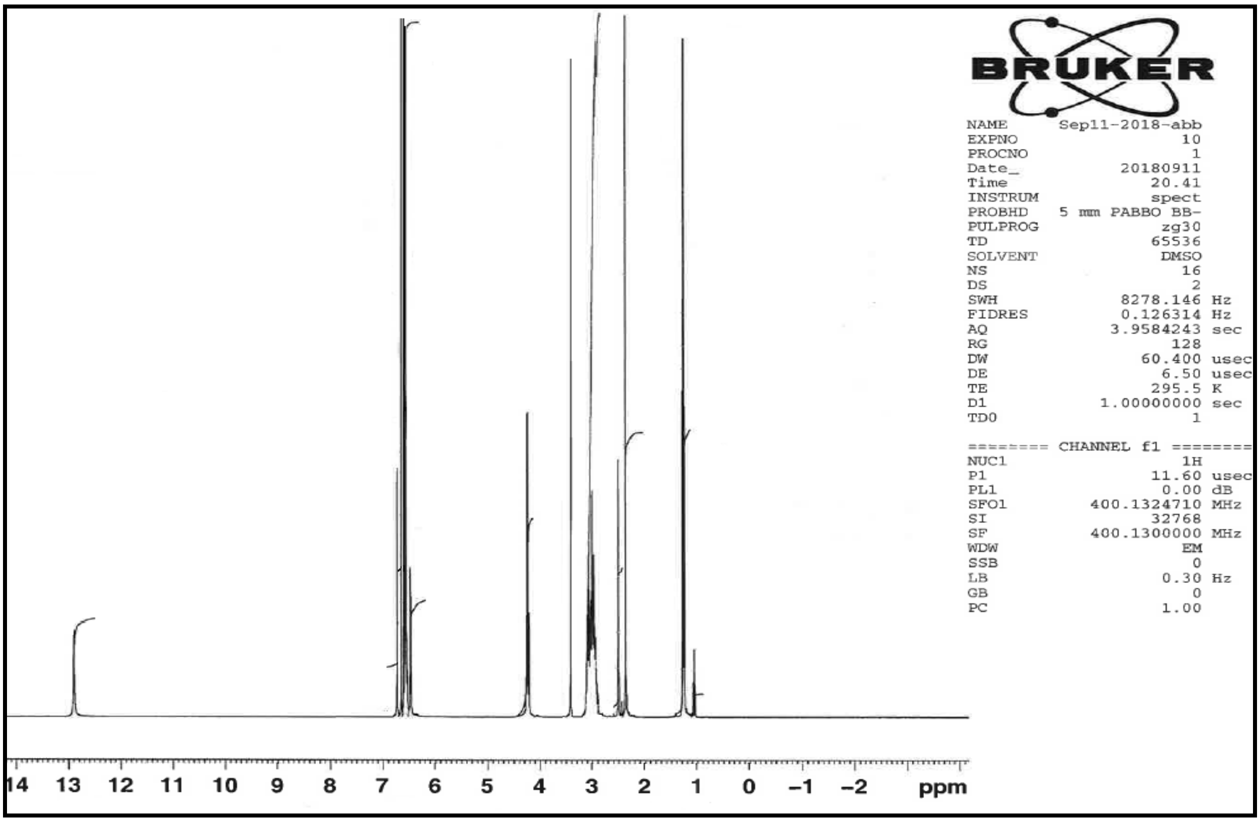

^{13}C-NMR of 28

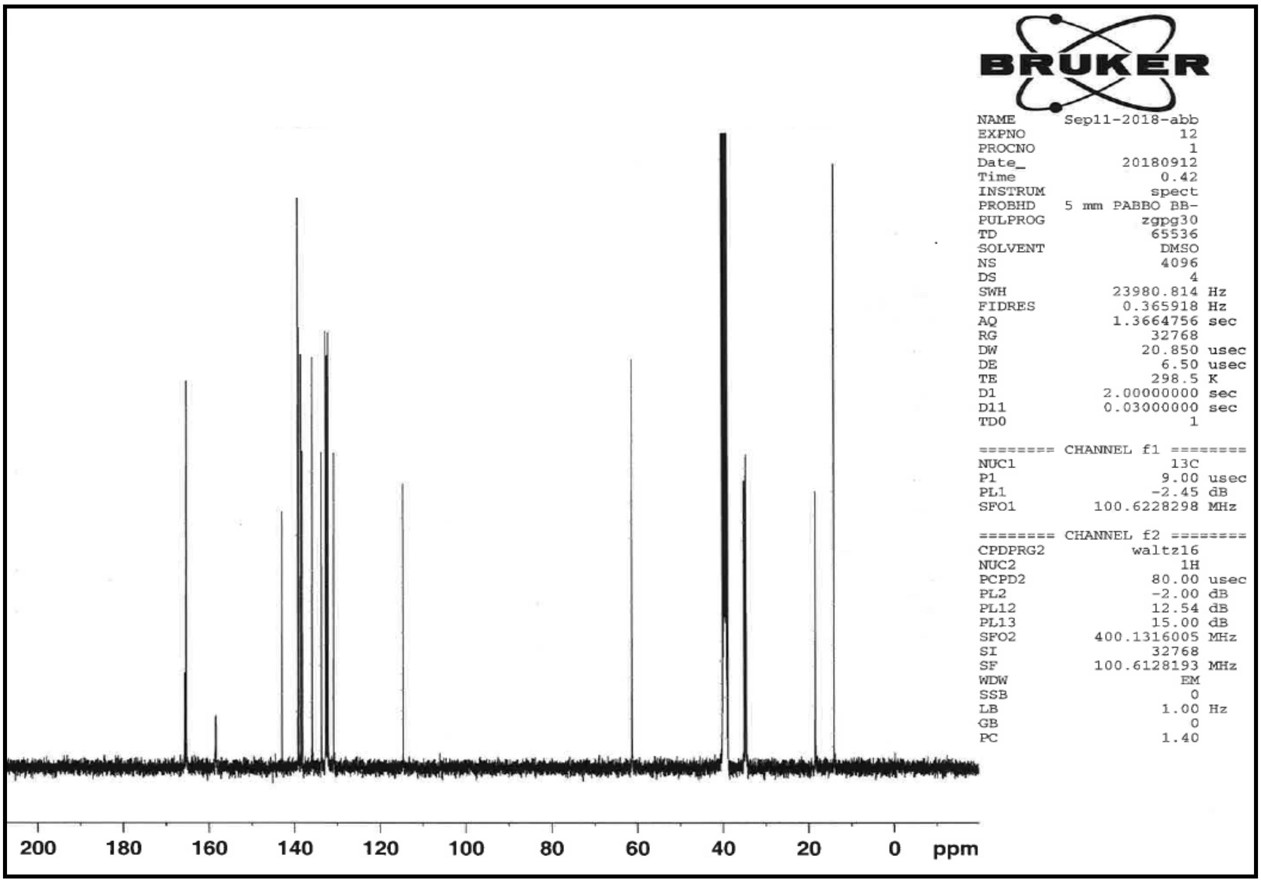

^{1}H-NMR of 29

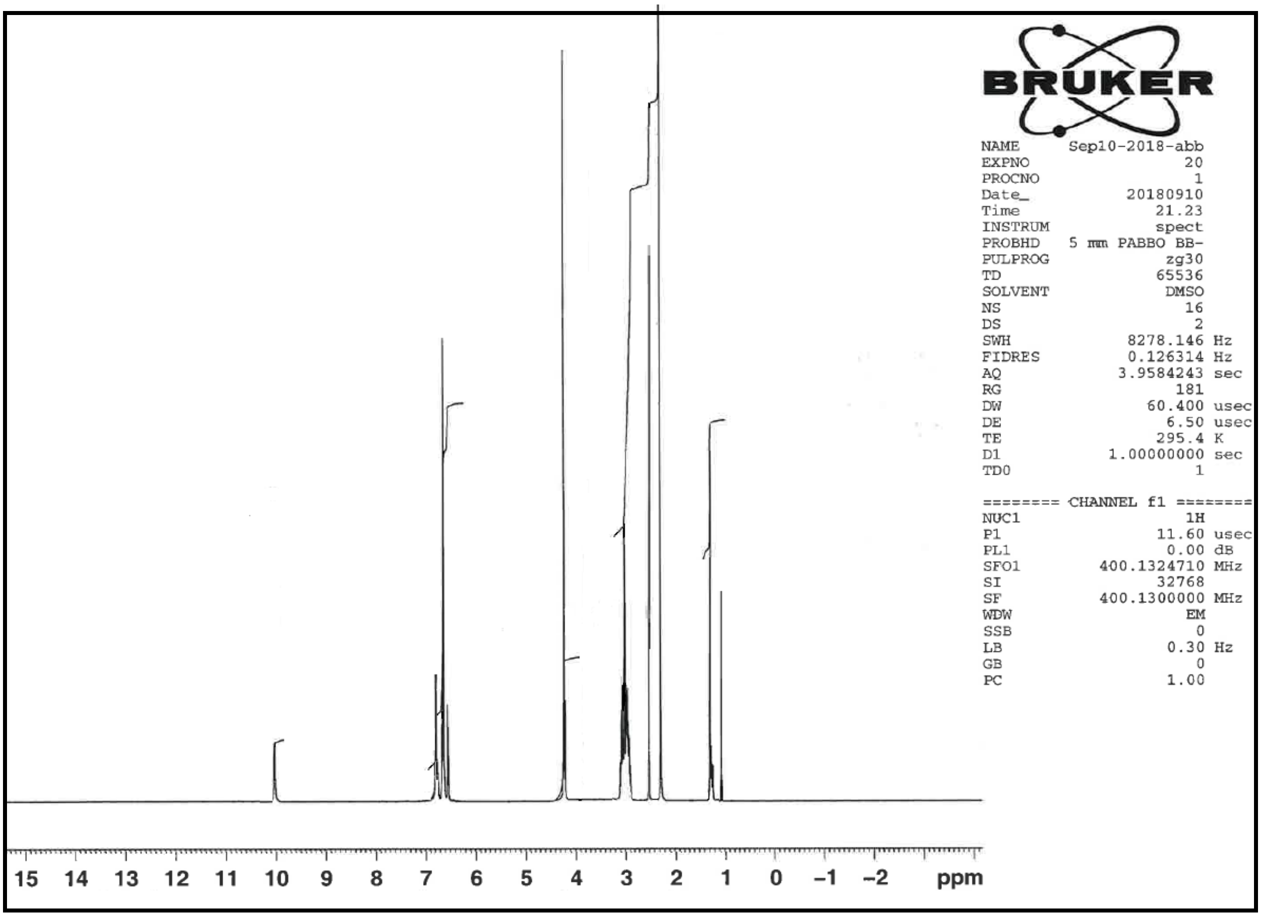
BRUKER
NAME Sep10-2018-abb
EXPNO 20
PROCNO 1
Date_ 20180910
Time 21.23
INSTRUM spect
PROBHD 5 mm PABBO BB-
PULPROG zg30
TD 65536
SOLVENT DMSO
NS 16
DS 2
SWH 8278.146 Hz
FIDRES 0.126314 Hz
AQ 3.9584243 sec
RG 181
DW 60.400 usec
DE 6.50 usec
TE 295.4 K
D1 1.00000000 sec
TD0 1
======== CHANNEL f1 ========
NUC1 1H
P1 11.60 usec
PL1 0.00 dB
SFO1 400.1324710 MHz
SI 32768
SF 400.1300000 MHz
WDW EM
SSB 0
LB 0.30 Hz
GB 0
PC 1.00
15 14 13 12 11 10 9 8 7 6 5 4 3 2 1 0 −1 −2 ppm

^{13}C-NMR of 29

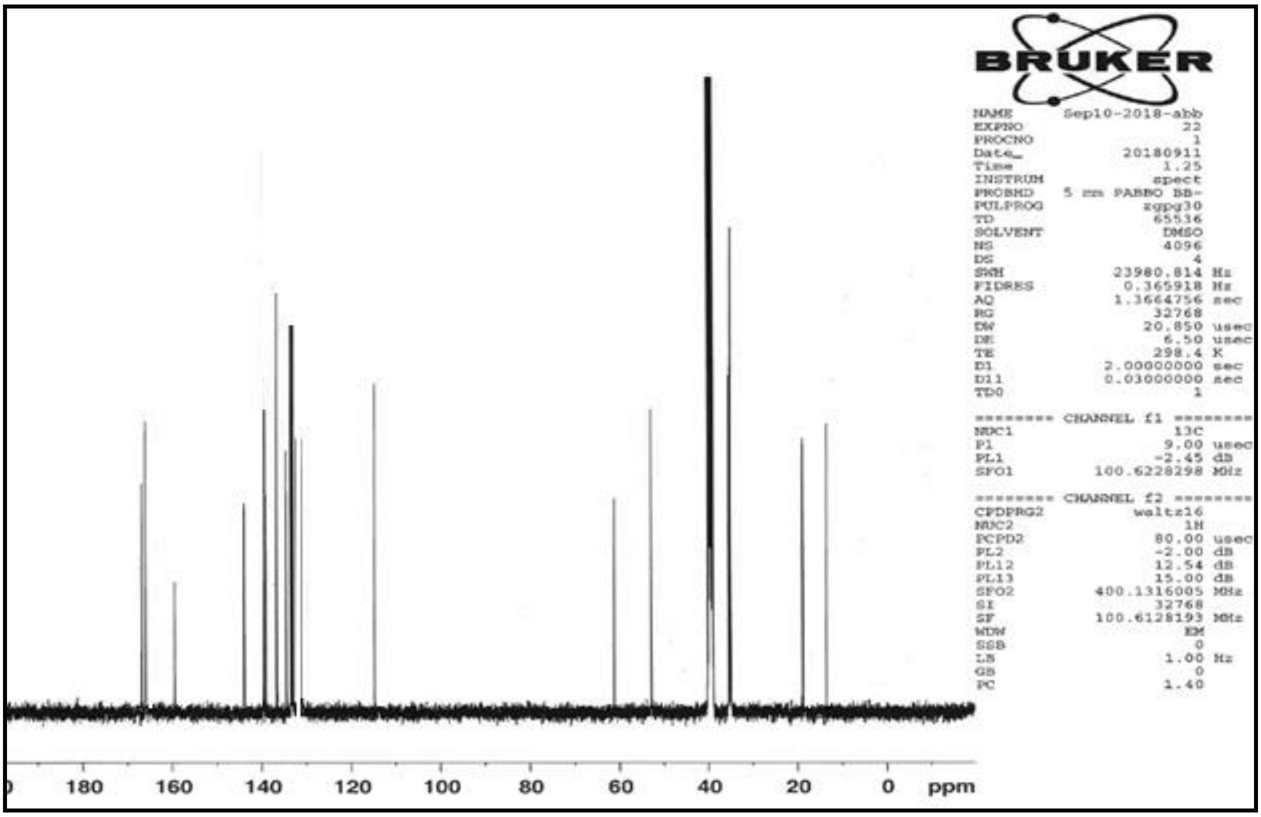
BRUKER
NAME Sep10-2018-abb
EXPNO 22
PROCNO 1
Date_ 20180911
Time 1.25
INSTRUM spect
PROBHD 5 mm PABBO BB-
PULPROG zgpg30
TD 65536
SOLVENT DMSO
NS 4096
DS 4
SWH 23980.814 Hz
FIDRES 0.365918 Hz
AQ 1.3664756 sec
RG 32768
DW 20.850 usec
DE 6.50 usec
TE 298.4 K
D1 2.00000000 sec
D11 0.03000000 sec
TD0 1
======== CHANNEL f1 ========
NUC1 13C
P1 9.00 usec
PL1 -2.45 dB
SFO1 100.6228298 MHz
======== CHANNEL f2 ========
CPDPRG2 waltz16
NUC2 1H
PCPD2 80.00 usec
PL2 -2.00 dB
PL12 12.54 dB
PL13 15.00 dB
SFO2 400.1316005 MHz
SI 32768
SF 100.6128193 MHz
WDW EM
SSB 0
LB 1.00 Hz
GB 0
PC 1.40
180 160 140 120 100 80 60 40 20 0 ppm
